# Banking records reveal characteristics of financial abuse

**DOI:** 10.1038/s41586-026-11049-7

**Published:** 2026-09-23

**Authors:** Anna Trendl, Nicola Sharp-Jeffs, Karen Perrier, Jane Rodrick, John Gathergood, David Leake

**Affiliations:** 1 Behavioural Science, Lloyds Banking Group, London, UK; 2 Warwick Business School, University of Warwick, Coventry, UK; 3 Child and Woman Abuse Studies Unit, London Metropolitan University, London, UK; 4 Global Centre for Contextual Safeguarding, University of Durham, Durham, UK; 5 Group Customer Vulnerability, Lloyds Banking Group, London, UK; 6 School of Economics, University of Nottingham, Nottingham, UK

**Keywords:** Human behaviour, Interdisciplinary studies, Economics

## Abstract

The experience of financial abuse, a form of domestic abuse involving control over money and finances, can be severe and long-lasting^1–3^, yet difficult to document^4^. Evidence on the everyday experiences of victim-survivors in the context of financial abuse is drawn primarily from small-scale, qualitative surveys^5,6^. Using anonymized banking data from a major UK retail bank, we compared the financial outcomes of a group of female victim-survivors of financial abuse (*n* = 5,428; identified from customer disclosures to the bank) with a control group (*n* = 15,602; consisting of individuals with no known disclosures). The control group matched the demographic and socioeconomic characteristics of the victim-survivor group 7 years before disclosure. A comprehensive set of 373 transactional and non-transactional financial outcomes were analysed over this period. Results indicate that, compared with controls, victim-survivors exhibited depleting savings and increasing debts, culminating in missed payments and declining credit scores. They also spent less on self-care, incurred higher transport and legal costs, increased cash withdrawals, changed addresses and passwords more frequently and were more likely to claim welfare benefits. Our results demonstrate how victim-survivors lose financial independence, experience economic instability and suffer financial distress.

## Abstract (web summary)

An analysis of UK banking data reveals that people who reported being victim-survivors of financial abuse experience worse financial outcomes compared with a control group.

## Main

Domestic abuse is increasingly recognized as an important public health concern worldwide^7^. In the United Kingdom, the Domestic Abuse Act 2021 (ref. ^8^) provides a broad definition encompassing psychological, physical, sexual and economic abuse between people with personal connections (for example, family or intimate partners). In England and Wales, an estimated 12.6 million people have experienced domestic abuse, with women disproportionately affected (30% compared with 22%; ref. ^9^). The annual cost is estimated at £66 billion (ref. ^10^) (2017 prices; £91 billion in 2026 prices^11^), largely because of harm, lost productivity and health care costs.

Although research on domestic abuse has mostly focused on physical violence^12^, many scholars stress that violence must be understood within the wider context of coercive control^13,14^, which can also encompass psychological, sexual, economic and financial abuse. The terms ‘economic’ and ‘financial’ abuse are often used interchangeably in the literature, but while economic abuse is broadly defined as control over economic resources, financial abuse specifically focuses on control over money and finances^15^. Financial abuse can include restricting access to money (for example, controlling bank accounts or rationing cash), exploiting the victim for financial gain (for example, coercing welfare claims or accruing debt in their name) or sabotaging their finances (for example, preventing work or damaging credit rating)^16^. Financial abuse is often intertwined with other aspects of coercive control (for example, emotional, sexual or physical abuse)^3^, reflecting a broader constellation of harms that may include stress, injury, reduced work capacity, social isolation and poorer financial well-being.

It is estimated that 97% of all domestic abuse cases also involve some form of economic abuse, and that in 57% of cases the victim-survivor is coerced into debt^17^. Survey data from victim-survivors show an average individual debt of £32,000 (ref. ^17^), and an overall £14.4 billion of debt in the United Kingdom is estimated to be related to economic abuse^3^ (2020 prices; £18.78 billion in 2026 prices^11^). For the victim, loss of control over their financial life often co-occurs with difficulties across non-financial domains of life, including mental and physical health, the ability to pay for personal goods and participation in leisure and well-being activities^18,19^.

Previous studies based on interviews and small-scale surveys show that even years after being subjected to abuse, victim-survivors often remain burdened by substantial debt, damaged credit scores^1^ and the less visible loss of financial skills and confidence^2^, all of which hinder regaining financial independence and stability^3^. Yet, signs of financial abuse are often overlooked by the criminal justice process, as it is harder to evidence than physical abuse^4,20^. Because it often begins early in relationships^15^ and precedes around a third of domestic homicide cases^21^, addressing this gap could inform harm prevention efforts.

Although financial abuse leaves no physical marks, it creates clear, measurable traces in financial records. Financial institutions, as custodians of these data, are well placed to help bridge this evidence gap. Reflecting this potential, the principal financial trade association of the United Kingdom, UK Finance, has recently called on its members to identify how financial abuse manifests in financial records^22^. Despite this, research has relied predominantly on cross-sectional surveys and qualitative studies, with relatively few investigations using administrative data. Consequently, the potential of financial records to improve our understanding of victim-survivors’ experiences of financial abuse remains largely unexplored.

Previous studies have demonstrated the value of financial transaction data for examining a wide range of societal issues, including the association between gambling, financial and lifestyle outcomes^23^, the financial markers of loss of financial capacity^24^ and the carbon emissions associated with household spending^25^. Apart from the insights revealed by transaction patterns, banking data contain a broader spectrum of behavioural markers, including bank account management activity (for example, online logins, password changes), offering additional insight into the experiences of victim-survivors.

For understanding financial abuse, banking data offer distinct advantages over commonly used survey data sources. Static, cross-sectional survey data on finances can be costly to collect per participant, narrow in focus and prone to recall bias, particularly in the context of trauma, in which autobiographical recall may be fragmented^26^. By contrast, banking data offer continuous, objectively inferred, complete, timestamped records of financial behaviours over time at scale. Moreover, financial data allow us to follow the same individual over time, capturing how financial abuse is associated with changes in victim-survivors’ financial lives.

Here we conduct a large-scale retrospective analysis of anonymized, individual-level banking records from the largest retail bank of the United Kingdom linked to victim-survivors of financial abuse. The bank serves about 51% of UK adults and is representative by age, gender, region and income (Supplementary Table 1). Female victim-survivors self-disclosed their experiences to the specialist Domestic and Financial Abuse Team of the bank (VS group; *n* = 5,428). Bank staff are trained to identify potential vulnerabilities, including financial and domestic abuse, during routine interactions (for example, querying transactions, clarifying charges). When staff judge that a customer may have experienced domestic or financial abuse and might benefit from further support, they offer a referral to the specialist team of the bank. With consent from the customer, the specially trained team contacts the customer to understand more about the nature and experience of abuse and, where possible, provide personalized support. The most common forms of support provided are separation of joint accounts, temporary suppression of charges and signposting to relevant charities for support.

Using a matched case–control design with 15,602 control individuals (control group), we examine 373 financial behavioural indicators over the 7 years preceding the disclosure of financial abuse. Our analysis focuses on self-disclosing victim-survivors with ongoing banking relationships, allowing us to track outcomes over time. The matched design controls for socioeconomic confounders, enabling the comparison of victim-survivors’ financial well-being with controls over time. The outcomes analysed here span a wide range of behaviours, including financial balances, spending patterns and account management. To our knowledge, this is one of the first empirical illustrations of the experience of financial abuse in victim-survivors’ everyday lives using banking data.

## Behavioural indicators of financial abuse

**Fig. 1:**
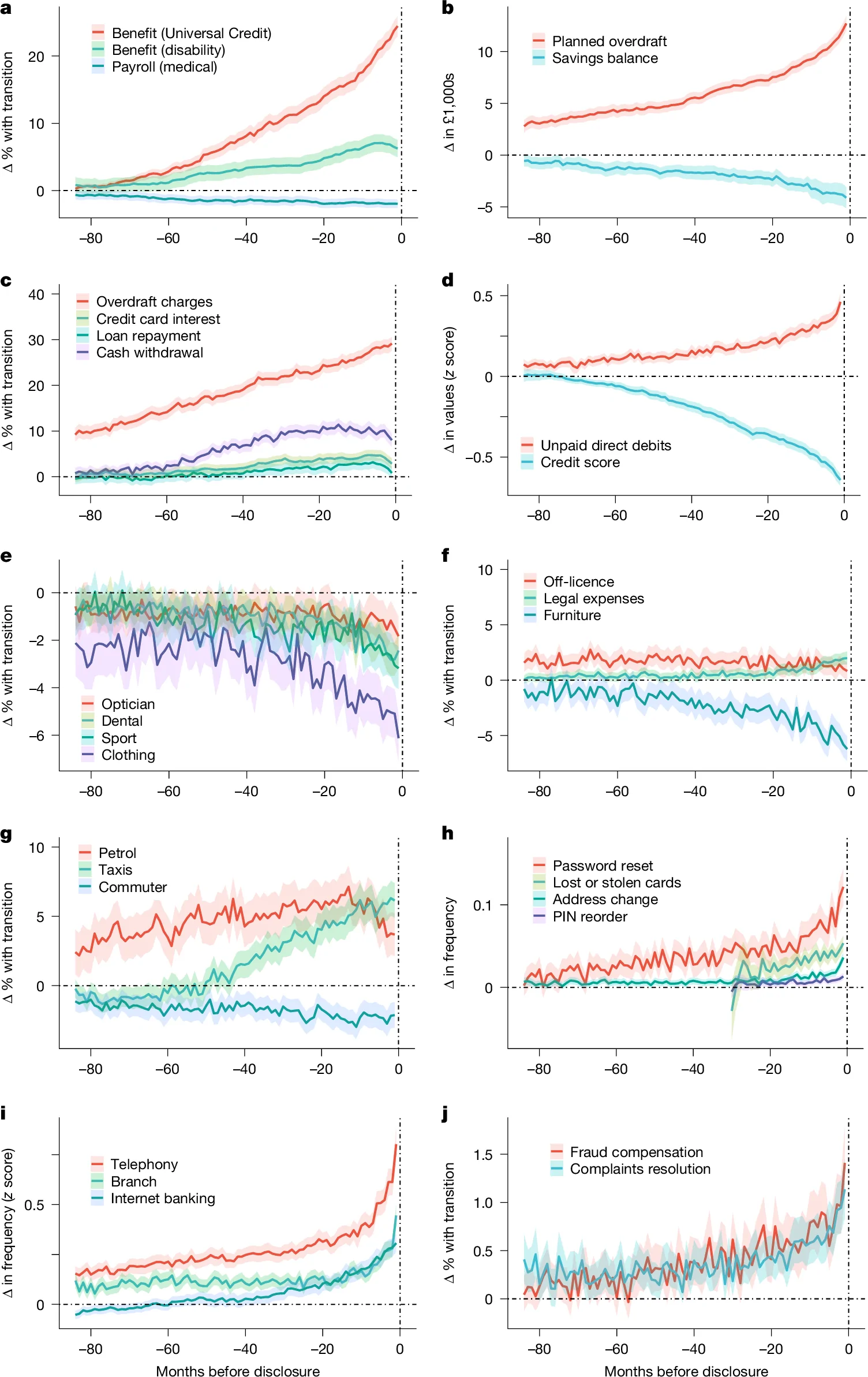
Monthly VS–control group differences in selected financial and non-financial indicators in the primary matched sample. Line plots show the monthly difference between the VS and control groups (VS − control) relative to disclosure (month = 0). Shaded ribbons indicate 95% confidence intervals around the estimated group differences. Sample sizes were *n* = 5,428 victim-survivors and *n* = 15,602 matched controls. Data on lost or stolen cards and PIN reorder were available from June 2022 onwards. Financial indicators (**a**–**d**). **a**, Sources of income: VS–control difference in monthly group proportion with transactions. Exact variables used are as follows: benefits (disability living allowance, other); benefits (Universal Credit, other); and payroll (medical, NHS). **b**, Financial balances: VS–control difference in monthly group averages (measured in thousands of pounds). Exact (non-transactional) variables used: planned overdraft and savings balance. **c**, Borrowing and cash usage: VS–control difference in monthly group proportion with transactions. Exact variables used are as follows: credit card interest (derived transactional), financial services (loans, other; transactional), overdraft charges (derived transactional), ATM withdrawal (derived transactional). **d**, Financial distress. VS–control differences in standardized monthly values (*z* scores). Exact variables are as follows: credit score (non-transactional) and monthly counts of unpaid direct debits (derived transactional). Non-financial indicators (**e**–**h**). **e**, Self-care: VS–control difference in monthly group proportion with transactions. Exact variables used are as follows: retail (clothing, women); services (health, dental); services (health, optician); hobbies and interests (sport, other). **f**, Personal spend: VS–control difference in monthly group proportion with transactions. Exact variables used are as follows: retail (furniture, other); services (professional, legal); retail (food and drink, alcohol). **g**, Transport: VS–control difference in monthly group proportion with transactions. Exact variables used as follows: travel (commuter, other); motor (petrol, other); travel (taxis, other). **h**, Access and security: VS–control difference in monthly frequency. Exact (non-transactional) variables used are as follows: address change, lost or stolen card, password reset and PIN reorder. **i**, Interactions. VS–control differences in standardized monthly frequencies (*z* scores). Exact (non-transactional) variables used are as follows: branch visits, internet banking logins, and telephone visits. **h**, Remedial payments: VS–control difference in monthly group proportion with transactions. Exact variables used are as follows: financial services (fraud, write off); financial services (other, complaints resolution).

Supplementary Table 2 shows the descriptive statistics of the VS and control groups (primary matched sample). To define the subset of 373 indicators reflecting differences associated with financial abuse, we first identified 161 transactional and non-transactional outcomes in which the VS–control difference was statistically significant (*P* < 0.01) in the year before disclosure. From this subset of 161 outcomes, we then selected 30 representative variables to illustrate the temporal dynamics of the financial markers of financial abuse (Fig. 1). Below, we report the results of statistical tests of VS–control group differences in the period 1  year before disclosure. Unless otherwise specified, all statistical tests reported below are based on *n* = 5,428 victim-survivors and *n* = 15,602 matched controls.

Supplementary Table 3 reports statistical test results for VS–control differences across all 373 outcome variables over three pre-disclosure periods: 7–3.5 years, 3.5–0 years and 1 year before disclosure. To provide further context to the VS–control differences, we also report corresponding descriptive estimates of variable means 1 year before disclosure for a wider group of randomly selected bank customers (bank population benchmark; *n* = 19,059) and a group of randomly selected female bank customers (female bank population benchmark; *n* = 18,906). Below, we report the bank population benchmark estimates. Estimates for both benchmark samples are provided in Supplementary Table 4.

## Financial indicators

Figure 1a shows that the VS group is less likely to receive payroll from the National Health Service (NHS; the largest employer in the United Kingdom^27^; difference −1.8 percentage points (pp), [95% confidence interval (CI) −2.6 to −1.1], *χ*^2^ = 21.9; adjusted *P* < 0.001; bank population benchmark: 3.9%, [95% CI 3.6–4.2]), and more likely to transition to Universal Credit (the largest welfare benefit in the United Kingdom^28^; difference 28.4 pp, [95% CI 26.9–29.9]; *χ*^2^ = 1,542.5, adjusted *P* < 0.001; bank population benchmark: 9.8%, [95% CI 9.4–10.2]) and disability benefit (difference 7.9 pp, [95% CI 6.6–9.3], *χ*^2^ = 150.9, adjusted *P* < 0.001; bank population benchmark: 9.1%, [95% CI 8.7–9.5]) compared with the control group. Welfare benefits provide a vital safety net for victim-survivors, particularly when abusers restrict their ability to work^29^. However, the current design of the Universal Credit system, which requires a single household account, has been criticized as it may make it harder for victim-survivors to protect payments from an abuser^30^.

Figure 1b,c shows the increased borrowing activity and reduced financial inclusion of the VS group relative to the control group illustrated by higher average planned overdrafts (difference £10,389, [95% CI £9,768–11,010]; *t* = 32.8, adjusted *P* < 0.001; bank population benchmark: £2,488, [95% CI £2,445–2,532]), lower savings balances (*n* = 4,149 victim-survivors; *n* = 12,181 matched controls; difference −£3,501, [95% CI −£4,319 to −£2,683]; *t* = −8.4, adjusted *P* < 0.001; bank population benchmark: £14,215, [95% CI £13,738–14,693]), higher activity in transaction categories relating to credit card interest (difference 5 pp, [95% CI 3.6–6.3]; *χ*^2^ = 59.8, adjusted *P* < 0.001; bank population benchmark: 15.9%, [95% CI 15.4–16.5]), loan repayments (difference 9.6 pp, [95% CI 8–11.1]; *χ*^2^ = 155.4, adjusted *P* < 0.001; bank population benchmark: 25.5%, [95% CI 24.9–26.1]), overdraft charges (difference 34.6 pp, [95% CI 33.2–36]; *χ*^2^ = 1,924.5, adjusted *P* < 0.001; bank population benchmark: 27.1%, [95% CI 26.5–27.7]) and cash withdrawals (difference 4 pp, [95% CI 3.3–4.7]; *χ*^2^ = 92.8, adjusted *P* < 0.001; bank population benchmark: 85.9%, [95% CI 85.4–86.4]). Additional analyses showed that victim-survivors were more likely than controls to submit credit card applications (*χ*^2^(1, *n* = 21,030) = 148.1; adjusted *P* < 0.001) and loan applications (*χ*^2^(1, *n* = 21,030) = 104.7; adjusted *P* < 0.001) during the pre-disclosure period. We found no evidence of group differences in successful credit card applications (*χ*^2^(1, *n* = 21,030) = 0.8, adjusted *P* = 0.399) or successful loan applications (*χ*^2^(1, *n* = 21,030) = 1.6, adjusted *P* = 0.399). These findings suggest that higher debt levels among victim-survivors may be driven, at least partly, by increased demand for credit (Supplementary Fig. 1). The increased financial burden from escalating debt repayments and depleting savings observed here is consistent with earlier research describing financial exploitation in contexts in which the victim-survivor is relatively advantaged in terms of assets or access to credit^5^. The pattern of increased cash usage is consistent with a range of experiences reported in the literature, including financial exploitation through unauthorized or forced cash withdrawals aiming to deplete the victim-survivor’s resources^31^ as well as patterns in which multiple small cash withdrawals are used by the victim-survivor to retain greater discretion over spending in contexts of heightened monitoring^32^.

Figure 1d shows that indicators of financial distress, including reduced credit scores (difference −109 points, [95% CI, −116 to −101]; *t* = −30.2, adjusted *P* < 0.001; bank population benchmark: 960, [95% CI 957–962]) and increased unpaid direct debit events (ACH debit; difference 31.9 pp, [95% CI 30.4–33.4]; *χ*^2^ = 1,726.4, adjusted *P* < 0.001; bank population benchmark: 16.5%, [95% CI 16–17.1]) are more prevalent in the VS group compared with the control group. Credit score measures individual creditworthiness using data from all bank accounts and financial products, including those beyond the bank used in our analysis and summarizes the increasing financial strain experienced by the VS group relative to the control group. Missed automated direct debit payments across key spending categories, including household bills, loans, mortgages and subscriptions, can substantially harm credit scores, as shown here^33^. Damaged credit scores hinder victim-survivors’ ability to gain financial stability and rebuild their lives^3^.

## Non-financial indicators

Figure 1e,f shows that relative to the control group, transactional activity of the VS group is lower towards self-care spend categories such as dental (difference −8.8 pp, [95% CI −10.2 to −7.4]; *χ*^2^ = 136.6, adjusted *P* < 0.001; bank population benchmark: 34.8%, [95% CI 34.2–35.5]), optician (difference −2.9 pp, [95% CI −4.3 to −1.5]; *χ*^2^ = 15.4, adjusted *P* < 0.001; bank population benchmark: 27.6%, [95% CI 27–28.2]), and sport (difference −5.3 pp, [95% CI −6.9 to −3.8]; *χ*^2^ = 46.5, adjusted *P* < 0.001; bank population benchmark: 36.5%, [95% CI 35.9–37.2]). Decreased spending on self beyond basic necessities aligns with patterns of financial control documented in previous studies, including restricted access to funds and financial exploitation, both of which have been linked to reduced financial autonomy among victim-survivors^3,34^.

We also observe that the VS group show higher spending activity towards off-licence stores (liquor store; difference 4.8 pp, [95% CI 3.4–6.3]; *χ*^2^ = 44.3, adjusted *P* < 0.001; bank population benchmark: 23.5%, [95% CI 22.9–24.1]) and legal expenses (difference 5.9 pp, [95% CI 4.8–6.9]; *χ*^2^ = 141.9, adjusted *P* < 0.001; bank population benchmark: 8.1%, [95% CI 7.7–8.5]), but lower activity towards furniture (difference −8 pp, [95% CI −9.5 to −6.5]; *χ*^2^ = 1,20.3, adjusted *P* < 0.001; bank population benchmark: 55.6%, [95% CI 54.9–56.3]), compared with the control group. Higher spending in off-licence stores is consistent with previous evidence showing that victim-survivors may use alcohol as a coping strategy^35^. Increased legal expenditures probably reflect efforts by victim-survivors to pursue legal separation or protective measures. The expense of legal representation can make up over half the overall estimated cost of escaping an abuser^36^, which, for many, presents a substantial barrier to accessing justice and securing their safety.

Figure 1g shows that the VS group has higher spending activity towards petrol (difference 5 pp, [95% CI 3.8–6.3]; *χ*^2^ = 60.8, adjusted *P* < 0.001; bank population benchmark: 66.7%, [95% CI 66–67.4]) and taxis (difference 12.3 pp, [95% CI 10.8–13.9]; *χ*^2^ = 246.2, adjusted *P* < 0.001; bank population benchmark: 31.9%, [95% CI 31.2–32.6]), but lower activity in commuting-related spend (consisting of spend on urban public transport companies, for example, transport for London; difference −5.5 pp, [95% CI −6.9 to −4.1]; *χ*^2^ = 54.8, adjusted *P* < 0.001; bank population benchmark: 28.1%, [95% CI 27.5–28.7]) compared with the control group. Lower commuting-related spending in the VS group aligns with earlier findings of lower employment prevalence among victim-survivors^3^. Higher spending on flexible transport options, including cars and taxis, has been reported previously and may reflect the complex, constrained circumstances in which victim-survivors meet everyday needs, including limited alternatives, time pressures and safety considerations^37^.

Figure 1h,i shows increased VS–control difference in account management activity related to PIN reorders (*n* = 4,514 victim-survivors; *n* = 12,981 matched controls; difference 0.008, [95% CI 0.007–0.01]; *t* = 10.7, adjusted *P* < 0.001; bank population benchmark: 0.003, [95% CI 0.002–0.003]), password resets (difference 0.079, [95% CI 0.07–0.088]; *t* = 18.1, adjusted *P* < 0.001; bank population benchmark: 0.044, [95% CI 0.042–0.045]), reporting lost or stolen cards (*n* = 4,514 victim-survivors; *n* = 12,981 matched controls; difference 0.041, [95% CI 0.037–0.045]; *t* = 18.4, adjusted *P* < 0.001; bank population benchmark: 0.019, [95% CI 0.018–0.02]), address changes (difference 0.017, [95% CI 0.016– 0.019]; *t* = 22, adjusted *P* < 0.001; bank population benchmark: 0.008, [95% CI 0.007–0.008]) and interactions with the bank across different channels (branch, difference 0.114, [95% CI 0.104–0.123]; *t* = 23.5, adjusted *P* < 0.001; bank population benchmark: 0.08, [95% CI 0.078–0.082]; internet banking, difference 10.1, [95% CI 8.3–11.9]; *t* = 11, adjusted *P* < 0.001; bank population benchmark: 25.2, [95% CI 25–25.4]; telephony, difference 0.567, [95% CI 0.518–0.616]; *t* = 22.7, adjusted *P* < 0.001; bank population benchmark: 0.109, [95% CI 0.106–0.112]). All of these activities reflect changes in account access and management consistent with those reported in situations involving financial abuse, including both shifts in control by an abuser and increased engagement by victim-survivors post-separation, when it may be safe to do so^38^. Frequent changes of address align with the residential instability often associated with separation.

Finally, Fig. 1j shows that the VS group is relatively more likely to receive remedial payments for fraud (difference 7.5 pp, [95% CI 6.6–8.4]; *χ*^2^ = 425.4, adjusted *P* < 0.001; bank population benchmark: 2.4%, [95% CI 2.2–2.6]) and complaints (difference 6.1 pp, [95% CI 5.4–6.8]; *χ*^2^ = 593.2, adjusted *P* < 0.001; bank population benchmark: 1%, [95% CI 0.9–1.1]). Apart from arrears management, fraud and complaint-related interactions are potential points of disclosure to the bank, which could explain their prevalence in our sample. Past research has found that victim-survivors are more likely to disclose financial abuse to the bank than the police or a domestic abuse service^15^. One survey revealed that whereas 23% of victims sought support from their bank, only 13% spoke to the police^39^.

## Alternative comparison groups

To test for the sensitivity of the results, we repeated the previous analyses using two alternative samples.

First, we created a distress-matched sample, matching victim-survivors (*n* = 5,183) with controls (*n* = 13,430) who experienced similar levels of financial distress in the year before disclosure. This allows us to separate patterns associated with abuse from those related to financial distress. Second, we created a smaller relationship-end-matched sample that included victim-survivors for which information on relationship end date was available (*n* = 1,149) and corresponding matched controls (*n* = 3,236; 21% of the primary matched control sample). By comparing VS–control differences at relationship end, rather than disclosure date, we can examine the extent to which the patterns reported are specific to the pre-separation period. Apart from defining the individual analysis period using the relationship end date, we followed the same matching procedure as in the primary sample. Supplementary Table 2 presents descriptive statistics for the VS and control groups. All statistical tests reported below for the distress-matched sample are based on *n* = 5,183 victim-survivors and *n* = 13,430 matched controls.

**Fig. 2:**
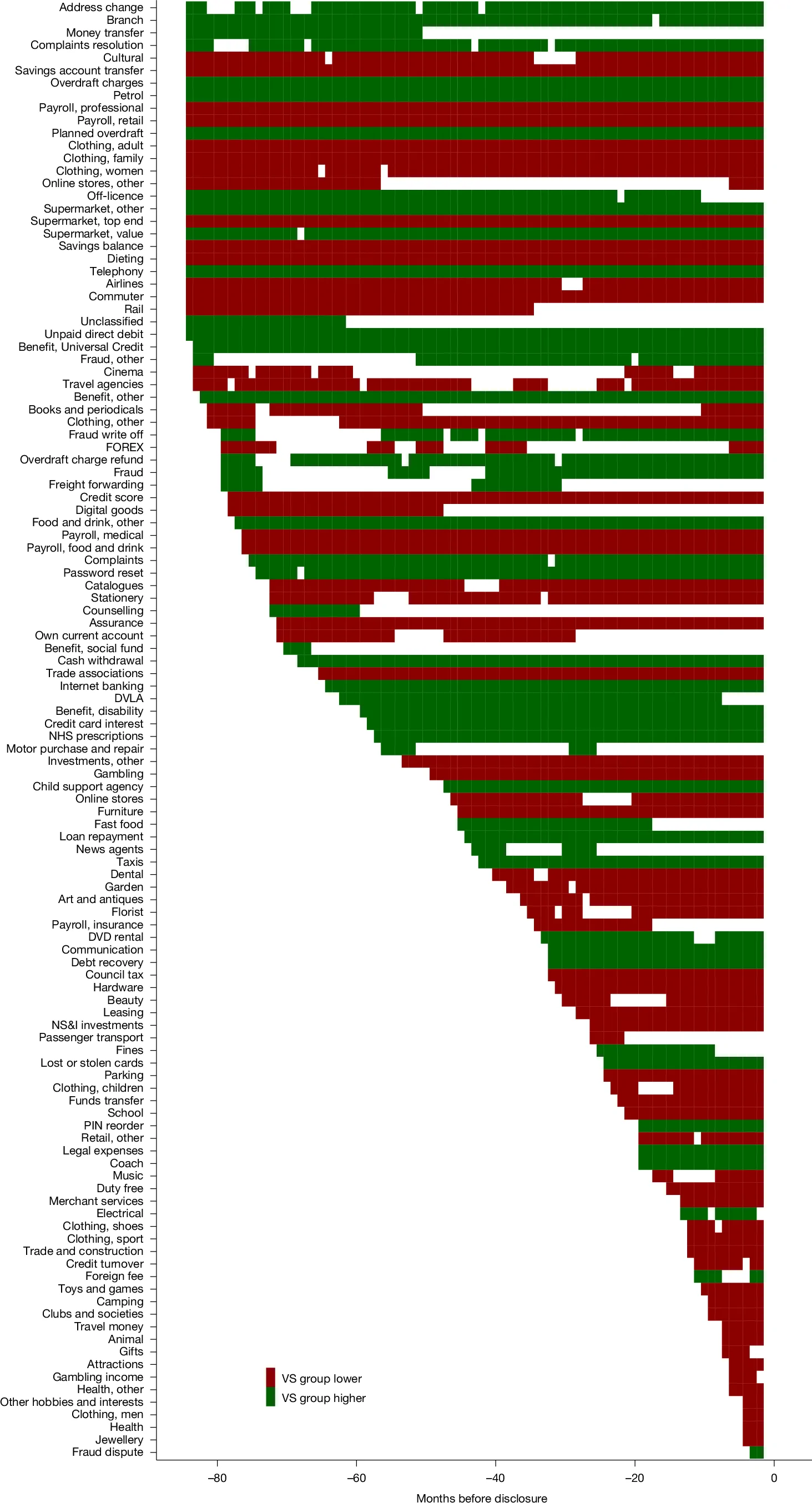
Individual cluster profiles of temporal VS–control group differences in the primary matched sample. Tile plot showing significant temporal clusters of VS–control differences for 116 financial indicators. Sample sizes: VS (*n* = 5,428), control (*n* = 15,602). For each financial indicator (*n* = 373) and time point (*n* = 84), we estimated an ordinary least squares regression with group membership (VS compared with control) as the predictor of interest and recorded the corresponding *t*-statistic. Temporal cluster candidates were identified where the *t*-value exceeded 1.96 (*P* = 0.05, two-sided). We then retained only those clusters whose summed *t*-values were significant at *P*  < 0.001 (two-sided), as determined by a 1,000-iteration permutation test. The horizontal axis shows the number of months relative to disclosure date (month = 0). Colours denote the direction of the difference (green, VS group higher; red, control group higher). Financial indicators on the vertical axis are ordered by the onset of significant cluster differences (earliest first). Abbreviated variable labels are used for readability; the correspondence between abbreviated and original financial indicator names is provided in Supplementary Table 7.

Results from the distress-matched sample (Supplementary Fig. 2 and Supplementary Table 5) show that a close match is achieved for financial-distress related variables (Fig. 2b–d). The VS group shows distinctive patterns compared with the distress-matched control sample, replicating the main comparison for variables, including benefit receipt, cash withdrawals, account access and security, and bank interactions.

The VS–control difference observed in disability benefit receipt (difference, 14.3 pp, [95% CI 13–15.7]; *χ*^2^ = 539.7, adjusted *P* < 0.001) is almost double of that observed in the primary matched sample (difference 7.9 pp, [95% CI 6.6–9.3]; *χ*^2^ = 150.9, adjusted *P* < 0.001). This amplified difference highlights health-related vulnerabilities as a potentially important pathway into financial distress among victim-survivors, beyond that observed in financially distressed women without victim-survivor status. In the United Kingdom, it is estimated that disabled adults—defined as those living with a long-standing illness, condition or impairment—are 2.6 times more likely to experience domestic abuse compared with non-disabled adults^40^.

No evidence for VS–control difference in optician spend was found (difference −0.5 pp, [95% CI −2 to 1]; *χ*^2^ = 0.4, adjusted *P* = 0.645), and estimated differences were smaller for dental (difference −3.4 pp, [95% CI −4.9 to −1.9]; *χ*^2^ = 20.2, adjusted *P* < 0.001, compared with difference −8.8 pp, [95% CI −10.2 to −7.4]; *χ*^2^ = 136.6, adjusted *P* < 0.001, in the primary matched sample) and furniture spend (difference −4.5 pp, [95% CI −6 to −2.9]; *χ*^2^ = 33.2, adjusted *P* < 0.001, compared with difference −8 pp, [95% CI −9.5 to −6.5]; *χ*^2^ = 120.3, adjusted *P* < 0.001, in the primary matched sample). Attenuated differences in self-care spend indicate that the experience of victim-survivors of financial abuse in these dimensions are not distinctive from women in financial distress without a victim-survivor status.

In contrast to results from the primary matched sample, no evidence of VS–control differences in the distress-matched sample were found for petrol (difference 0.8 pp, [95% CI −0.5 to 2]; *χ*^2^ = 1.6, adjusted *P* = 0.344, compared with difference 5 pp, [95% CI 3.8–6.3]; *χ*^2^ = 60.8, adjusted *P* < 0.001 in the primary matched sample), whereas differences in taxi spend were markedly attenuated (difference 2.5 pp, [95% CI 0.9–4.1]; *χ*^2^ = 9.5, adjusted *P* = 0.003, compared with difference 12.3 pp, [95% CI 10.8–13.9]; *χ*^2^ = 246.2, adjusted *P* < 0.001 in the primary matched sample). These findings indicate that the previously observed differences in these spending categories may not be unique to women experiencing financial abuse and may also be present among women experiencing financial distress. These financial-distress-related travel pressures may include irregular income, reduced flexibility and time constraints^41^.

Finally, the distress-matched results reveal distinctive bank interaction patterns for victim-survivors. The direction of the VS–control difference was reversed for internet banking logins (difference −3.4, [95% CI −5.3 to −1.5]; *t* = −3.5, adjusted *P* < 0.001, compared with difference 10.1, [95% CI 8.3–11.9]; *t* = 11, adjusted *P* < 0.001, in the primary matched sample). This indicates that, although increased internet banking logins are associated with financial distress, victim-survivors tend to rely more on human banking channels. This may reflect barriers to accessing internet banking, or the greater complexity and sensitivity of their needs, which are less easily addressed through digital channels.

Results from the relationship-end-matched sample (Supplementary Fig. 3 and Supplementary Table 6) were consistent with those observed in the primary matched sample, although estimated VS–control differences were generally smaller and several estimates were centred near zero, consistent with the reduced statistical power expected from the smaller sample size. All statistical tests reported below for the relationship-end-matched sample are based on *n* = 1,149 victim-survivors and *n* = 3,236 matched controls.

Specifically, in the relationship-end-matched sample, no evidence for VS–control difference was found for payroll (difference −1.1 pp, [95% CI −2.6 to 0.5]; *χ*^2^ = 1.8, adjusted *P* = 0.377, compared with difference −1.8 pp, [95% CI −2.6 to −1.1]; *χ*^2^ = 21.9, adjusted *P* < 0.001, in the primary matched sample), optician spend (difference −2.3 pp, [95% CI −5.3 to 0.7]; *χ*^2^ = 2.3, adjusted *P* = 0.390, compared with difference −2.9 pp, [95% CI −4.3 to −1.5]; *χ*^2^ = 15.4, adjusted *P* < 0.001, in the primary matched sample) and sport spend (difference 1.3 pp, [95% CI −2 to 4.7]; *χ*^2^ = 0.6, adjusted *P* = 0.667, compared with difference −5.3 pp, [95% CI −6.9 to −3.8]; *χ*^2^ = 46.5, adjusted *P* < 0.001, in the primary matched sample).

Observed VS–control differences were larger for cash withdrawal (difference 7 pp, [95% CI 5.4–8.5]; *χ*^2^ = 52.7, adjusted *P* < 0.001, compared with difference 4 pp, [95% CI 3.3–4.7]; *χ*^2^ = 92.8, adjusted *P* < 0.001, in the primary matched sample) and petrol spend (difference 10.1 pp, [95% CI 7.4–12.7]; *χ*^2^ = 46.7, adjusted *P* < 0.001, compared with difference 5 pp, [95% CI 3.8–6.3]; *χ*^2^ = 60.8, adjusted *P* < 0.001, in the primary matched sample). The larger differences in cash withdrawals and petrol spending around relationship end compared with disclosure date may reflect practical demands associated with separation, including relocation or increased travel.

## Temporal patterns of differences

**Fig. 3:**
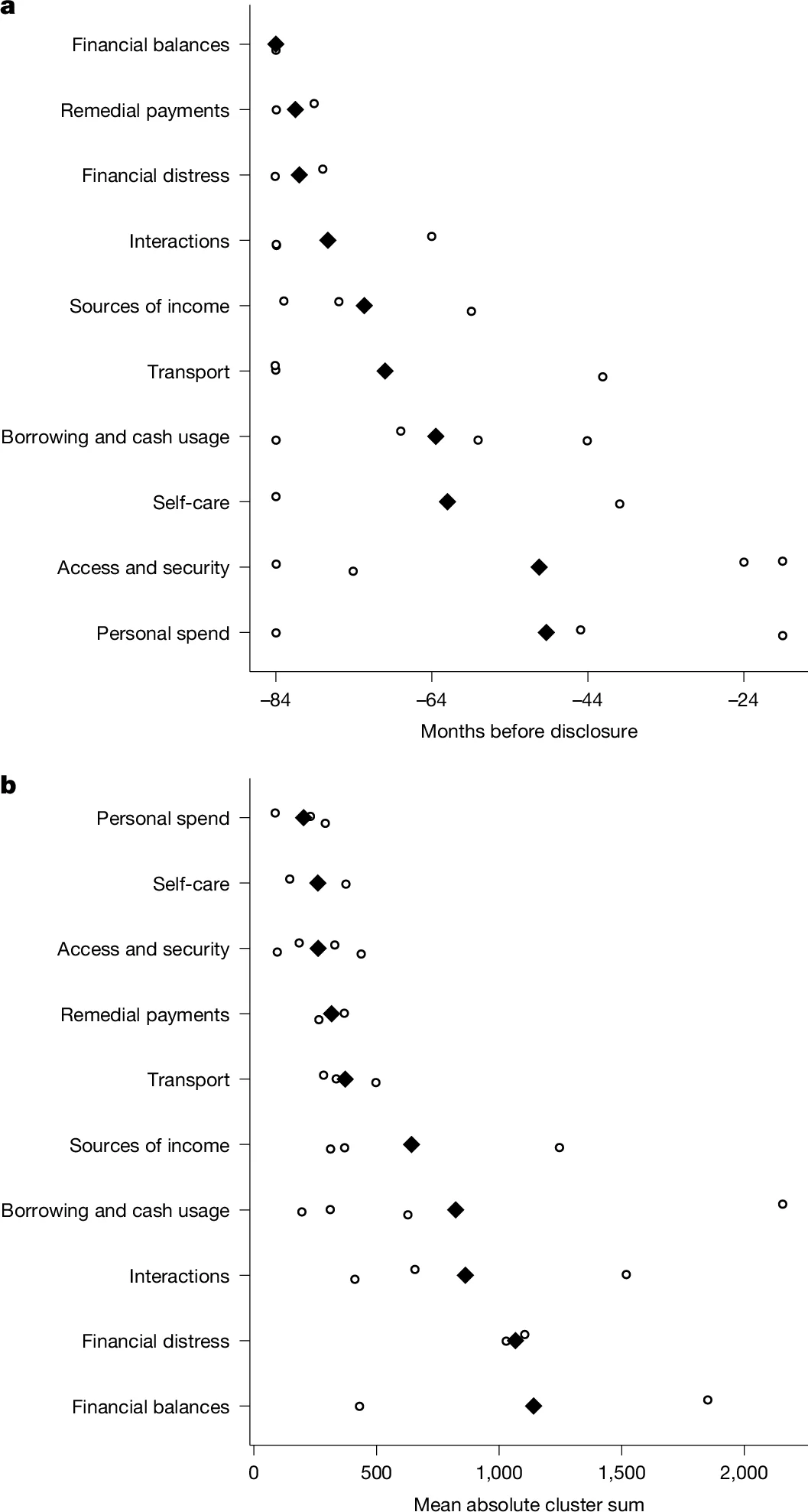
Aggregated temporal clustering results by financial indicator group in the primary matched sample. Financial indicator groups correspond to those shown in Fig. 1 (30 indicators). **a**, The earliest significant temporal cluster for each individual variable (open circles) together with the group mean (filled diamond), ordered by the average onset of differences. **b**, The absolute temporal cluster sum for each individual variable (open circles) together with the group mean (filled diamond), ordered by the average cluster size.

Figure 2 shows the individual cluster profiles for the 116 variables, in which a significant temporal cluster was identified, whereas Fig. 3 shows the aggregated results of the clustering analysis using the variable classification presented in Fig. 1. In line with the patterns shown in Fig. 1, Fig. 3a shows that group differences in financial balances, remedial payments and financial distress are present at the start of the analysis period. In outcomes related to interactions, income sources and transport, differences on average first appear 6years before disclosure. Differences in debt-related costs appear on average 5 years before disclosure, whereas differences in personal spend, self-care and account access appear the latest, about 4 years before disclosure. Figure 3b shows the average size of the differences, revealing that group differences are largest in categories related to financial balances and smallest in remedial payments.

## Discussion

To our knowledge, this is one of the first studies using administrative, objective data to describe how being subject to financial abuse manifests in victim-survivors’ lives. Our results show depleted savings, over-indebtedness and worsening financial strain, culminating in lower credit scores compared with a matched control group over the 7 years before disclosure.

The findings presented in this paper highlight considerations for measuring the socioeconomic costs of financial abuse. In the year preceding disclosure, compared with the broader bank population, victim-survivors were more likely to experience adverse financial outcomes (Supplementary Tables 3 and 4): the percentage with at least one missed direct debit was 279% higher (63% compared with 17%), whereas those relying on Universal Credit welfare benefit was 421% higher (51% compared with 10%). Similarly, compared with the averages of the bank population, victim-survivors’ credit scores were 20% lower (767 compared with 960), and their savings balances were 78% lower (£3,088 compared with £14,215).

Victim-survivors’ financial experiences vary in severity, with bank disclosures probably reflecting more adverse cases. However, given the magnitude of the harms identified—including damaged credit scores—and the estimated prevalence of financial abuse, these costs are likely to be economically significant. Previous estimates place the annual cost of domestic abuse in the United Kingdom at £91 billion (2026 prices)^4,5^, equivalent to approximately 7% of expected public expenditure in the United Kingdom in 2025–26 (ref. ^42^). Owing to data limitations, this estimate excludes costs associated with financial abuse. By quantifying these financial harms, our results could inform extensions of national cost models to capture the socioeconomic burden of domestic abuse more comprehensively.

The findings presented in this paper also have implications for the policy discourse on how to improve support for victim-survivors. For example, estimated group differences in average credit scores could inform the design of credit rating system reforms^43,44^, aiming to support victim-survivors regain financial independence and stability. Subsequently, estimated differences in selected financial indicators reported here could be used as a benchmark for assessing victim-survivors’ financial well-being in the context of these reforms.

Financial institutions have a direct interest in supporting vulnerable customers experiencing financial abuse^45,46^. Observed changes in cash usage, transport spending and account management activity are consistent with patterns reported around periods of safety-seeking or separation among victim-survivors. Many UK banks, including Lloyds Banking Group, are signatories of the Financial Abuse Code of UK Finance^47^, which sets industry standards for enabling safe disclosure and improving the financial outcomes of victim-survivors. In a recent report^22^, UK Finance proposed commissioning a project exploring how banking data could be leveraged to identify the risk indicators of financial abuse. Further research should identify the most effective set of indicators, evaluate their predictive validity and explore whether data coverage could be improved by combining data from multiple financial institutions.

Our study has several limitations. First, the victim-survivor group, comprising of women in the United Kingdom with bank accounts who disclosed their victim-survivor status to the bank and have met our sample selection criteria, is not necessarily representative of the average victim-survivor, especially if disclosure is associated with experiencing significant financial distress. The findings reported here are most directly applicable to contexts with high levels of financial inclusion and individualized banking systems, such as the United Kingdom and may not generalize to international contexts, in which financial abuse is shaped by different social and cultural norms^48^, nor to settings in which gendered and unpaid labour constitute central forms of economic control^49^. Further work is needed to understand the extent to which the results reported in this specific group are generalizable to other populations.

Second, there are limitations in the extent to which we can measure financial abuse. Given estimated levels of underreporting of financial abuse and our reliance on self-disclosure^50^, the control group may also include victim-survivors who did not disclose. This could result in an underestimation of true group differences. Financial abuse often co-occurs with other forms of abuse, and we cannot isolate indicators specific to financial abuse from these overlapping experiences, such as psychological control or restrictions on mobility. Owing to data availability restrictions, our analysis only looks at a maximum 7-year window and draws on data from one bank. As domestic abuse can span decades^6^, affecting all bank accounts held by the individual, this timeframe and data view may not reflect all of an individual’s financial patterns associated with financial abuse. Moreover, disclosure records do not include information on the duration of abuse before disclosure, which limits our ability to relate observed financial behaviours to the duration or timing of abuse.

Third, our methodological approach does not allow us to establish causality between financial abuse and observed outcomes. For example, there may be two-way relationship between financial abuse and financial distress, or factors jointly driving abuse and distress. Furthermore, administrative data do not allow us to observe individual motivations or decision-making processes underlying these patterns; qualitative research, such as life-history interviews, would be valuable for exploring these mechanisms in greater depth.

Despite these limitations, this study shows that financial data provide a valuable lens to understand the experience of financial abuse. The results presented in this study indicate that banking data could guide future efforts to develop vulnerability indicators and intervention strategies designed to support victim-survivors.

## Methods

### Ethical approval

Before commencement of the analysis, approval was obtained following review by the Privacy Risk and Impact Assessment (PRIA) governance body of the bank. Following an initial review by a Data Privacy Manager and a Data Privacy Risk Specialist, the application underwent an additional review by a Group Data Protection Officer, in accordance with the governance procedures of the bank for projects involving data relating to vulnerable customers. The reviewers were assigned according to the standard PRIA governance procedures of the bank and were independent of the project team. The PRIA application for this project was submitted in March 2024, and approval was granted in August 2024. The body approved use of the data in line with the project proposal for the publication of research entering the public domain. Customers were informed through the privacy notice of the bank^51^ at account opening that their personal data may be used for behavioural segmentation to better understand customer needs and that aggregated research findings and insights may be shared beyond the bank. Although the data privacy notice of the bank covers the use of customer data for research purposes, customers retained their existing rights to request the erasure of, or object to the processing of, their personal data, and use of their data for research was not a requirement of holding an account.

### Sample selection

#### Primary matched sample

We created two samples for this analysis, victim-survivor (VS) and control, constructed from the customer base of one of the largest retail banks in the United Kingdom, with 28 million customers in 2025 (ref. ^52^).

The filtering criteria applied to both groups were as follows: (1) women at least 18 years old at the start of the individual-specific 7-year period; (2) who have a current (checking) account; (3) who have not held a joint current (checking) account with another individual in the sample (4) who had at least 6 average monthly transactions within each year across their current (checking) and credit card accounts. The sample is restricted to women, as 87% of VS disclosing financial abuse to the bank identify as female.

First, we identified a sample of women who, between 2020 and 2024, disclosed to the specialist domestic and financial abuse team of the bank that they have been a VS of domestic or financial abuse (*n* = 9,728). The VS candidate sample is defined as the subset of this sample who have met the above filtering criteria (*n* = 5,444).

At the time of this analysis (May 2025), transactional data histories span 10 years (May 2015). Therefore, for those with a disclosure date in or after June 2022 (85% of the VS sample), the individual-specific analysis period is defined as the 7-year period preceding the disclosure date. For the remaining sample, for which we do not have access to the full 7 years’ worth of history, the earliest analysis month is May 2015. For this subsample, the shortest history available is 73 months, or just over 6 years.

Next, we constructed a group of control candidates using the following approach. From all female bank customers, we randomly selected an initial pool of 334,642 individuals. Applying the second and third restrictions reduced this control candidate sample to 263,638. We then randomly assigned a ‘disclosure date’ to all control candidates, sampled from the distribution of VS disclosure dates. With the exact analysis period now defined for all control candidates, we then restricted this sample to individuals who met our full inclusion criteria specified above (*n* = 45,702).

Finally, we performed propensity score matching, using the following matching variables: year and month of the individual-specific analysis period, age, median monthly credit turnover (sum of all credit to the individual’s account, excluding inter-account transfers and refunds; proxy for income), median monthly number of transactions, deprivation rank of the geography where the individual lived^53^, average credit score, flags for the receipt of the following benefits: child benefit, employment support allowance, disability living allowance and flags for the individual holding the following internal bank products: mortgage, credit cards and loans.

Except for age and deprivation rank, which were calculated in the first month, we used the first year of the analysis period to construct these variables. Propensity scores were estimated using the MatchIt package (v.4.5.5) in R (v.4.3.0) with a generalized linear model with a logistic link function. Matching was performed using nearest-neighbour matching with a 1:3 treatment-to-control ratio, a calliper of 0.1 and without replacement.

This process resulted in a final sample of 5,428 VS and 15,602 control individuals, and a combined sample of 21,030 individuals. Matching weights from the propensity score matching have been applied in all subsequent analyses reported. The analysis periods range between May 2015 and December 2024.

Supplementary Figs. 4 and 5 show the distribution of the matching variables listed above across samples, and Supplementary Table 8 shows the output of the propensity score matching.

### Distress-matched and relationship-end-matched samples

We applied the same filtering criteria as described above to create the distress-matched and relationship-end-date samples. Owing to changes in the data retention policy of the bank, at the time these analyses were conducted (January 2026), transaction histories used in the analyses below were restricted to 7 years. The analysis periods range between January 2019 and December 2024. Matching in the following analyses was performed using nearest-neighbour matching with a 1:3 treatment-to-control ratio, a calliper of 0.01 and without replacement.

We created the distress-matched VS (*n* = 5,387) and control candidate (*n* = 76,083) samples by applying the same filtering criteria as described above. To create a matched sample, we used variables capturing financial distress and matched the two samples at the end of the analysis period (12 months before disclosure date), to create a control sample that experienced similar levels of financial distress as the VS sample in the period preceding disclosure. We used the following matching variables: year and month of the individual-specific analysis period, age, median monthly credit turnover, median monthly number of transactions, deprivation rank of the geography where the individual lived, average credit score, credit score 12 months before disclosure, average monthly number of rejected direct debits and overdraft charges, average loan, savings and planned overdraft balances, binary flags for the following: savings, loan accounts, usage of planned overdraft, unpaid direct debit and overdraft charges.

The propensity score matching process resulted in a final sample of 5,183 VS and 13,430 control individuals. Supplementary Table 9 shows the output of the propensity score matching.

To create the relationship-end-date samples, we extracted relationship-end date information from the VS candidate sample. Applying the same criteria as described above, we retrieved notes associated with 5,303 victim-survivors. During appointments, with the customer’s consent, bank staff record key details of each case in the form of notes. The richness of the information recorded varies by note, but some include temporal markers, most commonly the relationship end date. We identified 3,559 notes that contained words indicative of a relationship end date. These records either included month names (for example, ‘jan’) or any of the following search words: fled, split, ended, ago, left. We then manually reviewed these notes and extracted relationship end dates with month-level precision. For example, ‘relationship ended 3 years ago’ was not accurate enough, but ‘fled 2 weeks ago’ would be included with a relationship end date 2 weeks before disclosure. We retrieved the latest known contact date with the abuser at the time of the disclosure, and only if it was within our analysis period (after January 2019). Therefore, relationship end could be missing if it was not mentioned in the note, was too long ago, was not specific enough or if the victim-survivor was still in the relationship at the time of disclosure. Manual review resulted in a sample of 1,189 VS sample candidates. On average, relationships ended 7.7 months (s.d. 9.3) before the disclosure date.

The matching variables were the same as those used in the primary matched sample, the only difference being that the individual-specific analysis period was now defined by the relationship end date as opposed to the disclosure date. The propensity score matching process resulted in a final sample of 1,149 VS and 3,236 control individuals. Supplementary Table 10 shows the output of the propensity score matching.

#### Bank population prevalence estimates

To contextualize the VS–control differences reported in the year before disclosure, we created two alternative samples, a random sample of all bank customers (bank population benchmark) and a random sample of female customers (female bank population benchmark). We have randomly selected an initial pool of 48,758 individuals for the bank population benchmark sample and 48,400 women for the female bank population benchmark sample. Applying the second and third restrictions reduced these control candidate samples to 38,492 and 38,449, respectively. We then randomly assigned a ‘disclosure date’ to all benchmark candidates, sampled from the distribution of VS disclosure dates. With the exact analysis period now defined for all benchmark candidates, we then restricted this sample to individuals who met our full inclusion criteria specified above, with the exception of the gender restriction for the bank population benchmark sample (19,059 and 18,906, respectively). Finally, we then retrieved the corresponding estimates for the 373 outcome variables analysed and 15 demographic variables. The estimates are reported in Supplementary Table 4. This analysis took place in January 2026. Supplementary Fig. 6 shows the distribution of VS and female bank population benchmark average credit scores in the year before the disclosure month.

#### Transactional and non-transactional outcomes

We retrieved the current account and credit card holdings of VS and control individuals in the matched samples within the individual-specific analysis period. Sole and joint accounts associated with an individual were both included. Next, transactions associated with these accounts in the relevant analysis period were retrieved. A transaction was defined as any credit or debit that occurred on a personal current checking or credit card account, including electronic transfers, online transactions and cash withdrawals using an ATM. Overall, in the largest, primary matched sample, 99% of the 162 million debit and credit card transactions retrieved were classified by the internal transaction classification system of the banks into 455 transaction categories. Using information on the nature of the transaction, we created seven additional derived transactional categories: ATM withdrawal, internal overdraft, internal credit card interest charges, cash advance (credit-card-specific), unpaid direct debit, unpaid cheque, and unclassified for any other remaining transactions. Credit scores range between 0 and 1,344 in the primary matched sample.

From these 462 categories, we kept only those in which at least 1% of the overall, primary matched sample had a transaction during our analysis period, resulting in 353 transaction categories. For our subsequent analyses involving transaction data, we converted monthly transaction count data into a binary format to capture the presence or absence of a transaction in a specific month.

Apart from the 353 transactional categories, 20 additional, non-transactional variables were retrieved. We used the same approach when retrieving transactional and non-transactional variables for the distress- and relationship-end-matched samples and focused on the 353 transactional and 20 non-transactional categories identified in the main analysis. These are a mix of binary and frequency indicators, including, but not limited to, product holdings, overdraft use, interactions with the bank, password and address changes. Unlike transactional variables, not all of these variables were available for the entire analysis period: unplanned, planned overdraft, credit score, internet banking, branch, telephone visits and reported fraud are only available from June 2015 (January 2019 in the distress- and relationship-end-matched samples), complaints frequency are only available from March 2017 (January 2019 in the distress- and relationship-end-matched samples), and PIN reorders and lost or stolen cards are only available from June 2022 (January 2023 in the distress- and relationship-end-matched samples). The full list of 373 financial variables constructed can be found in Supplementary Table 3. Across selected transactional and non-transactional variables, VS–control differences are shown in Fig. 1 (primary matched sample), Supplementary Fig. 2 (distress-matched sample) and Supplementary Fig. 3 (relationship-end-matched sample).

#### Testing group differences

The primary analysis was conducted between September 2024 and July 2025, whereas the additional analyses, including the distress- and relationship-end-matched samples, were conducted between December 2025 and January 2026. The analysis considered three pre-disclosure periods: 7–3.5 years, 3.5–0 years, and 1 year before disclosure. For transactional outcomes, weighted proportions of individuals with at least one transaction in each category during each period were compared between groups using weighted *χ*^2^ statistics. For non-transactional outcomes, an individual-specific average value was first calculated within each period, and weighted group means were compared using *t*-statistics. Statistical significance was assessed using two-sided permutation tests^54^ by randomly permuting group labels (1,000 permutations for transactional outcomes and 500 permutations for non-transactional outcomes). Because statistical inference was based on permutation tests rather than reference distributions, degrees of freedom are not reported. *P*-values were adjusted for multiple comparisons using the Benjamini–Hochberg false discovery rate procedure^55^. Analyses were conducted in R (v.4.3.0) using the survey package (v.4.4-2).

#### Analysis of the dynamics of group differences

To identify persistent group differences over time in the primary matched sample, for each outcome (*n* = 373) and time point (*n* = 84) pair, we ran a series of linear regressions with ordinary least squares estimation using group membership as a predictor. The analysis generated a two-dimensional matrix of *t*-statistics, representing the temporal evolution of group differences relative to the disclosure date for each outcome.

Next, we selected temporal *t*-statistics cluster candidates that exceeded 1.96 (corresponding to *P* = 0.05 for a two-sided test). To control for multiple comparisons through the familywise error rate^56,57^, we then generated joint null distributions of the largest absolute cluster sums through 1,000 permutation iterations and retained only candidate clusters significant at *P* < 0.001 (two-sided). Figure 2 shows the significant temporal clusters identified for individual financial indicators, and Fig. 3 summarizes the timing and magnitude of these clusters by financial indicator group. These analyses were conducted in Python (v.3.12.2) using the glmtools (v.0.2.1) and MNE packages^58^ (v.1.7.1).

#### Representativeness of the Lloyds Banking Group customer base

We identified those bank customers who had at least 12 transactions per month across their current (checking) and credit card accounts in the year 2024 (*n* = 15,177,227). For this sample, we then retrieved the following information in December 2024: gender, age, deprivation rank, region and joint account status. Population statistics by age, gender, and region and median household disposable income estimates for the United Kingdom were obtained from the Office for National Statistics (ONS)^59,60^. Median annual credit turnover (bank proxy for income) is approximated using monthly median credit turnover calculated for the period between October and December 2024 using data from individuals holding joint accounts (*n* = 5,615,574) to provide a suitable comparison with household-level median disposable income in the United Kingdom in 2024. The comparison can be found in Supplementary Table 1.

#### Representativeness of the VS sample

We assessed the representativeness of the VS sample by comparing the distribution of key demographic characteristics—age group, region of residence and disability status—with corresponding estimates from a nationally representative survey. Comparisons were restricted to victim-survivors residing in England and Wales to align with the geographic coverage of the survey benchmarks, which together comprise about 89% of the UK population. Age in the Lloyds Banking Group (LBG) sample was calculated as of December 2024. Variables were selected based on the availability of comparable measures across data sources. Supplementary Table 10 presents the percentage distribution of these characteristics for the VS sample restricted to England and Wales (*n* = 4,875) alongside the corresponding ONS estimates. The LBG sample includes women aged older than 18 years at the start of the 7-year analysis period. Consequently, the youngest participant was 25 years old in 2024. In the LBG sample, disability is inferred from disability benefit receipt.

#### Financial product applications and application outcomes

For the VS (*n* = 5,428) and control samples (*n* = 15,602), we retrieved information on applications for financial products and their outcomes during the analysis period. Application data were available for credit cards, current (checking) accounts, loans, mortgages and savings products. Monthly weighted differences in the proportion of individuals submitting applications and the proportion with successful applications were calculated between the VS and control groups relative to disclosure. The resulting time series are shown in Supplementary Fig. 1.

#### Measuring the impact of the filtering criteria

To assess the impact of the four filtering criteria of the VS sample, we examined whether age at the start of the analysis period and changes in deprivation rank and average credit score over the analysis period differed by inclusion status. We compared the final VS candidate sample (*n* = 5,444) with those who were filtered out (*n* = 4,284). These characteristics were selected because they are independent of account activity. The comparison is shown in Supplementary Table 12.

#### Measuring the degree of self-selection by financial distress

To investigate whether the VS group (*n* = 5,428) was composed predominantly of individuals experiencing financial distress, we compared the distribution of average credit scores during the year before disclosure between the female bank population benchmark (see ‘Female bank population prevalence estimates’; *n* = 18,906) and the VS sample. Supplementary Fig. 6 shows the resulting kernel density estimates.

### Reporting summary

Further information on research design is available in the Nature Portfolio Reporting Summary linked to this article.

## Online content

Any methods, additional references, Nature Portfolio reporting summaries, source data, extended data, supplementary information, acknowledgements, peer review information; details of author contributions and competing interests; and statements of data and code availability are available at https://doi.org/10.1038/s41586-026-11049-7.

## Supplementary information

Supplementary Information This file contains Supplementary Figs. 1–6 and Supplementary Tables 1, 2, 11 and 12.

Reporting Summary

Supplementary Tables 3–10 Supplementary Table 3. Primary matched sample: group differences across all 373 outcome variables. Supplementary Table 4. Bank population benchmark and female bank population benchmark estimates. Supplementary Table 5. Distress-matched sample: group differences across all 373 outcome variables. Supplementary Table 6. Relationship-end-matched sample: group differences across all 373 outcome variables. Supplementary Table 7. Mapping of original and abbreviated financial indicator names. Supplementary Table 8. Propensity score matching diagnostics (primary matched sample). Supplementary Table 9. Propensity score matching diagnostics (distress-matched sample). Supplementary Table 10. Propensity score matching diagnostics (relationship-end-matched sample).

Peer Review File

## Data Availability

Access to the raw data is restricted owing to confidentiality, contractual and regulatory obligations associated with customer banking records. Researchers wishing to enquire about access should contact the corresponding author. Any access to the underlying data is subject to approval in line with the internal governance, data privacy and security processes of the Lloyds Banking Group. To facilitate reproducibility, the accompanying code generates a model-derived synthetic dataset reproducing the aggregate statistical properties of the original dataset used in the study.

## Code availability

Data were extracted from the internal secure systems of the bank using Teradata SQL Assistant (v.16.20.0.12); the SQL code used for data extraction is commercially sensitive and therefore not publicly available. Requests regarding access to the extraction code should be directed to the corresponding author and are subject to approval by Lloyds Banking Group in accordance with its internal governance and information security requirements. The analysis presented in the study was conducted using R (v.4.3.0) and Python (v.3.12.2). The full analytical workflow of this analysis is provided in the accompanying code (https://osf.io/8emyq/overview?view_only=fc3d9c489eb34b878c2da214d24e4388), which generates a model-derived synthetic dataset that preserves the aggregate statistical properties of the original data and replicates the analysis steps used to obtain the main results presented in the paper.
