# Supplementary material for: Banking records reveal characteristics of financial abuse
Source: Nature. 2026 Sep 23;658(8138):1200–7. doi: 10.1038/s41586-026-11049-7 (PMC13716816; doi:10.1038/s41586-026-11049-7)
Supplement: Supplementary file 1 [file 41586_2026_11049_MOESM1_ESM.pdf]

nature portfolio

https://doi.org/10.1038/s41586-026-11049-7

Supplementary information

Banking records reveal characteristics of financial abuse

In the format provided by the authors and unedited

measure_type	value	ONS (UK)	LBG	unit
age	20 to 24	0.078	0.064	proportion
age	25 to 29	0.085	0.081	proportion
age	30 to 34	0.09	0.097	proportion
age	35 to 39	0.089	0.099	proportion
age	40 to 44	0.085	0.091	proportion
age	45 to 49	0.077	0.082	proportion
age	50 to 54	0.083	0.086	proportion
age	55 to 59	0.086	0.088	proportion
age	60 to 64	0.08	0.081	proportion
age	65 to 69	0.067	0.065	proportion
age	70 to 74	0.058	0.055	proportion
age	75 to 79	0.054	0.051	proportion
age	80 to 84	0.034	0.032	proportion
age	85 to 89	0.021	0.019	proportion
age	90 and over	0.012	0.009	proportion
gender	female	0.515	0.516	proportion
gender	male	0.485	0.484	proportion
region	North West	0.11	0.11	proportion
region	Yorkshire and The Humber	0.09	0.08	proportion
region	East Midlands	0.07	0.07	proportion
region	West Midlands	0.1	0.09	proportion
region	East of England	0.09	0.09	proportion
region	London	0.1	0.13	proportion
region	South East	0.13	0.14	proportion
region	South West	0.1	0.09	proportion
region	Northern Ireland	0.01	0.03	proportion
region	Scotland	0.1	0.08	proportion
region	Wales	0.05	0.05	proportion
region	North East	0.05	0.04	proportion
income	Median Credit Turnover	36,700	36,355	pounds

Supplementary Table 1. Comparison of the LBG customer population with the UK population. Demographic distributions of age, gender, region and median income for the Lloyds Banking Group (LBG; N = 15,177,227) customer population compared with UK population estimates from the Office for National Statistics (ONS). The LBG customer population closely approximates the UK population across these characteristics. Methodological details are provided in the section “Representativeness of the LBG customer base”.

variable	unit	VS	Control	VS_distress	Control_distress	VS_rltnshp_end	Control_rltnshp_end
Sample size	count	5428	15602	5183	13430	1149	3236
Mean Period Start Date	date	19/07/2016	23/07/2016	01/01/2019	01/01/2019	01/01/2019	01/01/2019
Mean Period End Date	date	30/06/2023	04/07/2023	30/06/2023	07/07/2023	24/08/2022	20/08/2022
Mean Age	years	35.26	34.9	37.77	37.86	33.96	33.76
SD Age	years	11.84	11.91	11.97	10.67	10.5	11.04
Mean Credit Turnover	£	1845.54	1861.44	2736.1	2739.35	2248.04	2162.36
SD Credit Turnover	£	1486.68	1364.34	2064.94	1734.92	1807.91	2134.62
Mean Transactions	count	56.31	56.83	85.27	85	67.17	68.02
SD Transactions	count	34.35	35.66	53.89	46.43	41.84	45.71
Mean Deprivation Rank	rank	12333.38	12287.73	12329.93	12147.72	11881.93	11632.93
SD Deprivation Rank	rank	9606.06	9729.56	9658.96	9690.44	9528.64	9413.52
Mean Credit Score	score	940.12	940.97	777.71	768.15	911.04	910.5
SD Credit Score	score	114.47	117.55	216.22	227.89	127.12	135.83
CB*	count	2510	7326			539	1551
CB	%	46	47			47	48
DB**	count	861	2070			180	444
DB	%	16	15			16	15
ESA***	count	803	1727			95	221
ESA	%	15	14			8	8
Loan	count	1405	4054			237	627
Loan	%	26	26			21	21
Mortgage	count	863	2677			137	410
Mortgage	%	16	17			12	13
Credit Card	count	2057	6114			383	1111
Credit Card	%	38	38			33	34

Supplementary Table 2. Descriptive statistics for the victim-survivor (VS) and Control groups in the primary, distress-matched, and relationship-end matched samples. Weighted means and standard deviations are reported for all variables included in the propensity score matching procedure. Weighted means for the primary and relationship-end matched samples were calculated using data from the first year of the

observation period, whereas those for the distress-matched sample were calculated using data from the final year. Age and deprivation rank were calculated from the first month of the observation period for the primary and relationship-end matched samples and from the final month for the distress-matched sample. Credit scores range from 0 to 1,344, and deprivation rank ranges from 1 (most deprived) to 32,844 (least deprived). *Child benefit; **Disability benefit; ***Employment and Support Allowance. Methodological details are provided in the section "Sample selection".

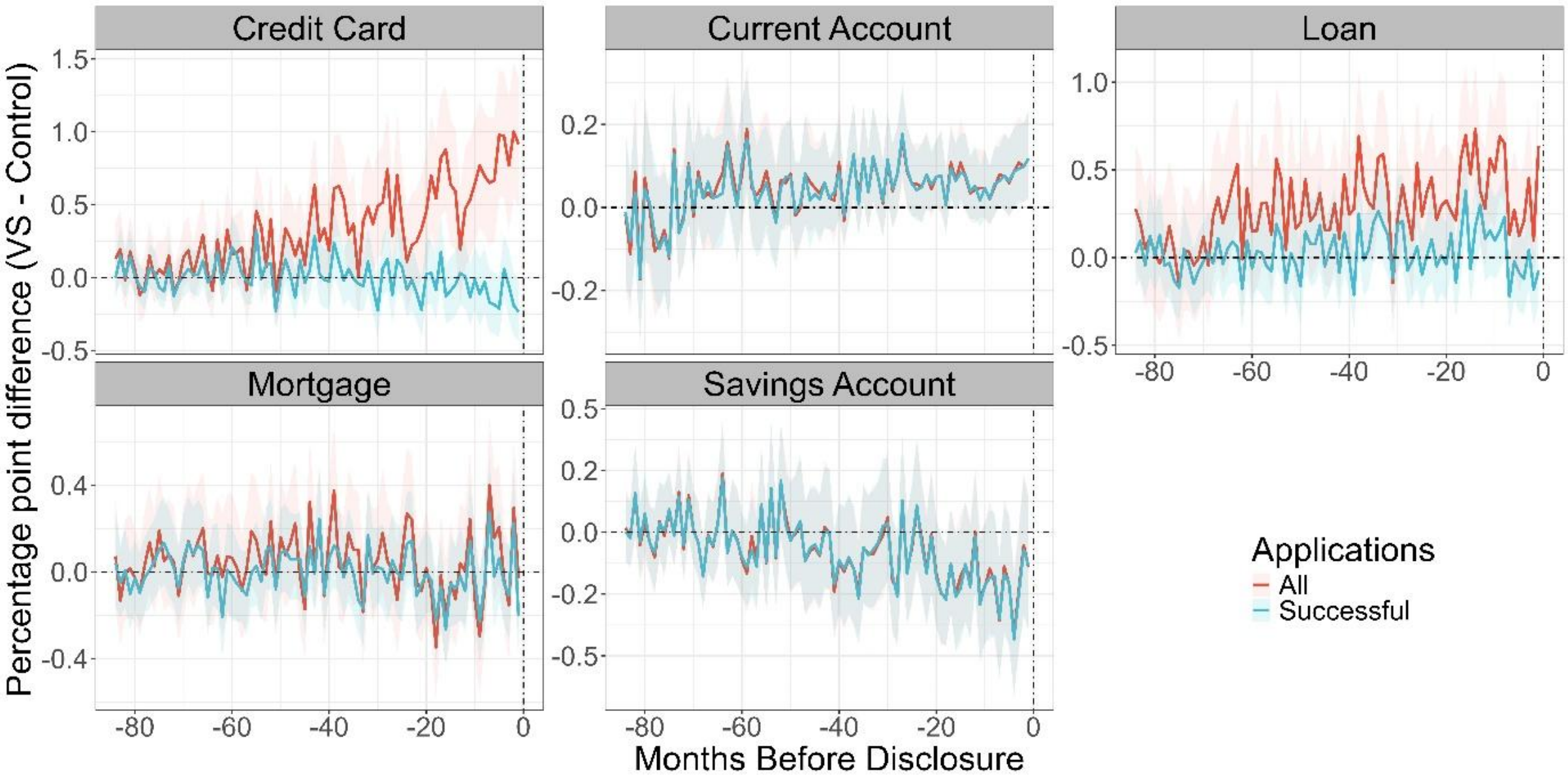

Supplementary Figure 1. Monthly VS–Control differences in financial product applications (primary matched sample). Line plots show the monthly difference between the VS and Control groups (VS–Control) in the proportion of individuals submitting financial product applications (All) and successful applications (Successful) relative to disclosure (month = 0). Shaded ribbons indicate 95% confidence intervals around the estimated group differences. Sample sizes were N = 5,428 victim-survivors and N = 15,602 matched controls. Panels show credit card, current account, loan, mortgage and savings product applications. Formal statistical comparisons are reported in the main text. Methodological details are provided in the section "Financial product applications and application outcomes".

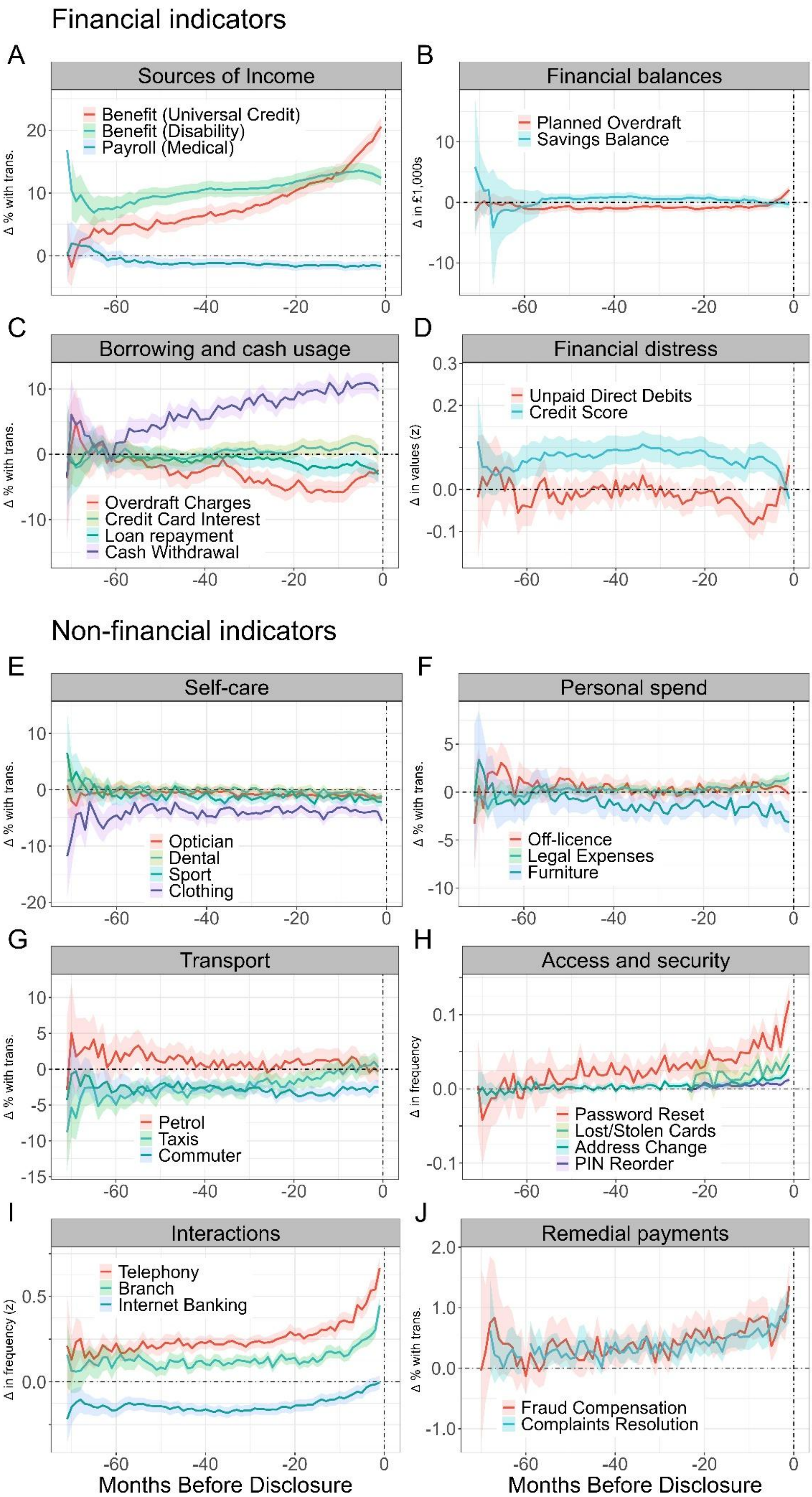
Financial indicators
A
Sources of Income
Benefit (Universal Credit)
Benefit (Disability)
Payroll (Medical)
Δ % with trans.
B
Financial balances
Planned Overdraft
Savings Balance
Δ in £1,000s
C
Borrowing and cash usage
Overdraft Charges
Credit Card Interest
Loan repayment
Cash Withdrawal
Δ % with trans.
D
Financial distress
Unpaid Direct Debits
Credit Score
Δ in values (z)
Non-financial indicators
E
Self-care
Optician
Dental
Sport
Clothing
Δ % with trans.
F
Personal spend
Off-licence
Legal Expenses
Furniture
Δ % with trans.
G
Transport
Petrol
Taxis
Commuter
Δ % with trans.
H
Access and security
Password Reset
Lost/Stolen Cards
Address Change
PIN Reorder
Δ in frequency
I
Interactions
Telephony
Branch
Internet Banking
Δ in frequency (z)
Months Before Disclosure
J
Remedial payments
Fraud Compensation
Complaints Resolution
Δ % with trans.
Months Before Disclosure

Supplementary Figure 2. Monthly VS–Control group differences in selected financial and non-financial indicators (distress-matched sample). Line plots show the monthly difference between the VS and Control groups (VS–Control) relative to disclosure (month = 0). Shaded ribbons indicate 95% confidence intervals around the estimated group differences. Sample sizes were N = 5,183 victim-survivors and N = 13,430 matched controls. Data on Lost/Stolen Cards and PIN Reorder were available from January 2023 onwards. **Financial indicators. Panel A. Sources of Income**. VS-Control difference in monthly group proportion with transactions. Exact variables used: Benefits/Disability Living Allowance/Other, Benefits/Universal Credit/Other, Payroll/Medical/NHS. **Panel B. Financial balances**. VS-Control difference in monthly group averages (measured in thousands of pounds). Exact (non-transactional) variables used: Planned Overdraft, Savings Balance. **Panel C. Borrowing and cash usage.** VS-Control difference in monthly group proportion with transactions. Exact variables used: Credit Card Interest (derived transactional), Financial Services/Loans/Other (transactional), Overdraft charges (derived transactional), ATM Withdrawal (derived transactional). **Panel D. Financial distress.** VS–Control differences in standardized monthly values (z scores). Exact variables: Credit Score (non-transactional) and monthly counts of Unpaid Direct Debits (derived transactional). **Non-financial indicators. Panel E. Self-care.** VS-Control difference in monthly group proportion with transactions. Exact variables used: Retail/Clothing/Women, Services/Health/Dental, Services/Health/Optician, Hobbies & Interests/Sport/Other. **Panel F. Personal spend.** VS-Control difference in monthly group proportion with transactions. Exact variables used: Retail/Furniture/Other, Services/Professional/Legal, Retail/Food & Drink/Alcohol. **Panel G. Transport.** VS-Control difference in monthly group proportion with transactions. Exact variables used: Travel/Commuter/Other, Motor/Petrol/Other, Travel/Taxis/Other. **Panel H. Access and security.** VS-Control difference in monthly frequency. Exact (non-transactional) variables used: Address Change, Lost/Stolen card, Password Reset, PIN Reorder. **Panel I. Interactions.** VS–Control differences in standardized monthly frequencies (z scores). Exact (non-transactional) variables used: Branch visits, Internet Banking logins, Telephone visits. **Panel J.** Remedial payments. VS-Control difference in monthly group proportion with transactions. Exact variables used: Financial Services/Fraud/Write Off, Financial Services/Other/Complaints Resolution.

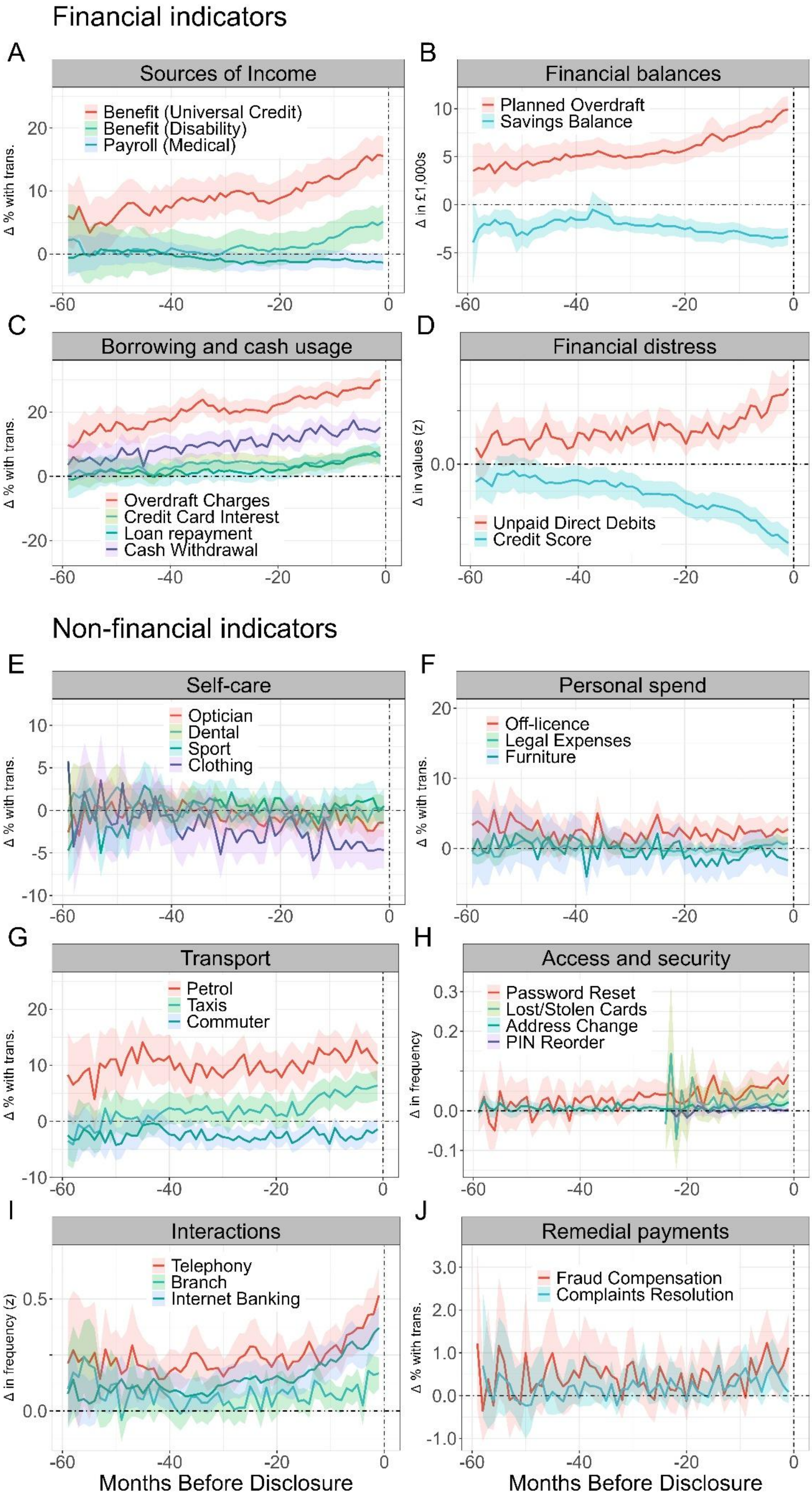
Financial indicators
A
Sources of Income
Benefit (Universal Credit)
Benefit (Disability)
Payroll (Medical)
Δ % with trans.
B
Financial balances
Planned Overdraft
Savings Balance
Δ in £1,000s
C
Borrowing and cash usage
Overdraft Charges
Credit Card Interest
Loan repayment
Cash Withdrawal
Δ % with trans.
D
Financial distress
Unpaid Direct Debits
Credit Score
Δ in values (z)
Non-financial indicators
E
Self-care
Optician
Dental
Sport
Clothing
Δ % with trans.
F
Personal spend
Off-licence
Legal Expenses
Furniture
Δ % with trans.
G
Transport
Petrol
Taxis
Commuter
Δ % with trans.
H
Access and security
Password Reset
Lost/Stolen Cards
Address Change
PIN Reorder
Δ in frequency
I
Interactions
Telephony
Branch
Internet Banking
Δ in frequency (z)
J
Remedial payments
Fraud Compensation
Complaints Resolution
Δ % with trans.
Months Before Disclosure
Months Before Disclosure

Supplementary Figure 3. Monthly VS–Control group differences in selected financial and non-financial indicators (relationship-end matched sample). Line plots show the monthly difference between the VS and Control groups (VS–Control) relative to disclosure (month = 0). Shaded ribbons indicate 95% confidence intervals around the estimated group differences. Sample sizes were N = 1,149 victim-survivors and N = 3,236 matched controls. Data on Lost/Stolen Cards and PIN Reorder were available from January 2023 onwards. **Financial indicators. Panel A. Sources of Income**. VS-Control difference in monthly group proportion with transactions. Exact variables used: Benefits/Disability Living Allowance/Other, Benefits/Universal Credit/Other, Payroll/Medical/NHS. **Panel B. Financial balances**. VS-Control difference in monthly group averages (measured in thousands of pounds). Exact (non-transactional) variables used: Planned Overdraft, Savings Balance. **Panel C. Borrowing and cash usage.** VS-Control difference in monthly group proportion with transactions. Exact variables used: Credit Card Interest (derived transactional), Financial Services/Loans/Other (transactional), Overdraft charges (derived transactional), ATM Withdrawal (derived transactional). **Panel D. Financial distress.** VS–Control differences in standardized monthly values (z scores). Exact variables: Credit Score (non-transactional) and monthly counts of Unpaid Direct Debits (derived transactional). **Non-financial indicators. Panel E. Self-care.** VS-Control difference in monthly group proportion with transactions. Exact variables used: Retail/Clothing/Women, Services/Health/Dental, Services/Health/Optician, Hobbies & Interests/Sport/Other. **Panel F. Personal spend.** VS-Control difference in monthly group proportion with transactions. Exact variables used: Retail/Furniture/Other, Services/Professional/Legal, Retail/Food & Drink/Alcohol. **Panel G. Transport.** VS-Control difference in monthly group proportion with transactions. Exact variables used: Travel/Commuter/Other, Motor/Petrol/Other, Travel/Taxis/Other. **Panel H. Access and security.** VS-Control difference in monthly frequency. Exact (non-transactional) variables used: Address Change, Lost/Stolen card, Password Reset, PIN Reorder. **Panel I. Interactions.** VS–Control differences in standardized monthly frequencies (z scores). Exact (non-transactional) variables used: Branch visits, Internet Banking logins, Telephone visits. **Panel J.** Remedial payments. VS-Control difference in monthly group proportion with transactions. Exact variables used: Financial Services/Fraud/Write Off, Financial Services/Other/Complaints Resolution.

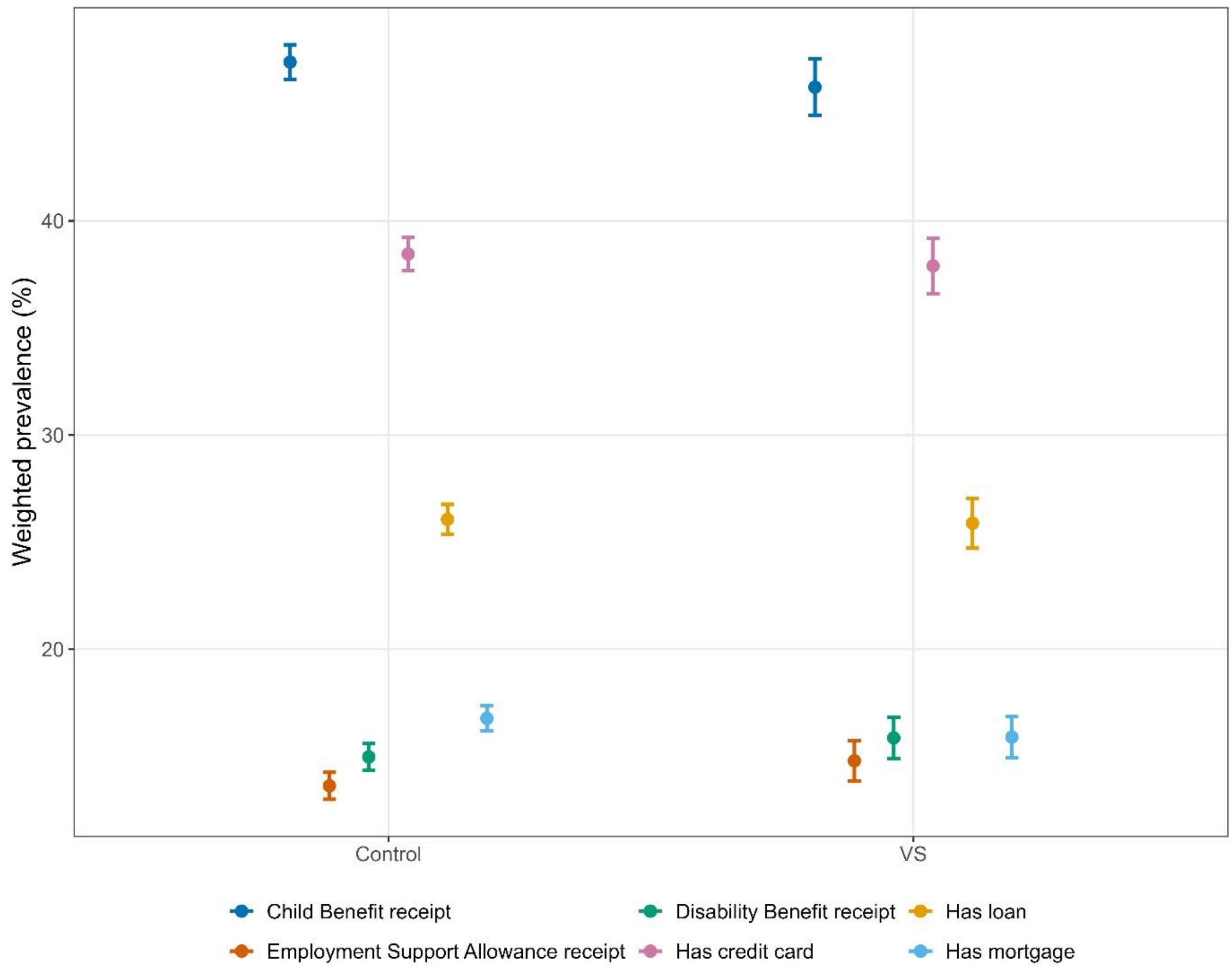

Supplementary Figure 4. Distribution of binary matching variables in the propensity score matched sample (primary matched sample). Weighted prevalence estimates for the six binary matching variables used in the propensity score matching are shown for the Control (N = 15,602) and victim-survivor (VS; N = 5,428) groups. Points represent weighted prevalence estimates and error bars denote 95% confidence intervals. Variables comprise receipt of Child Benefit, Employment Support Allowance and Disability Benefit, together with indicators for holding a credit card, loan and mortgage at the start of the analysis period.

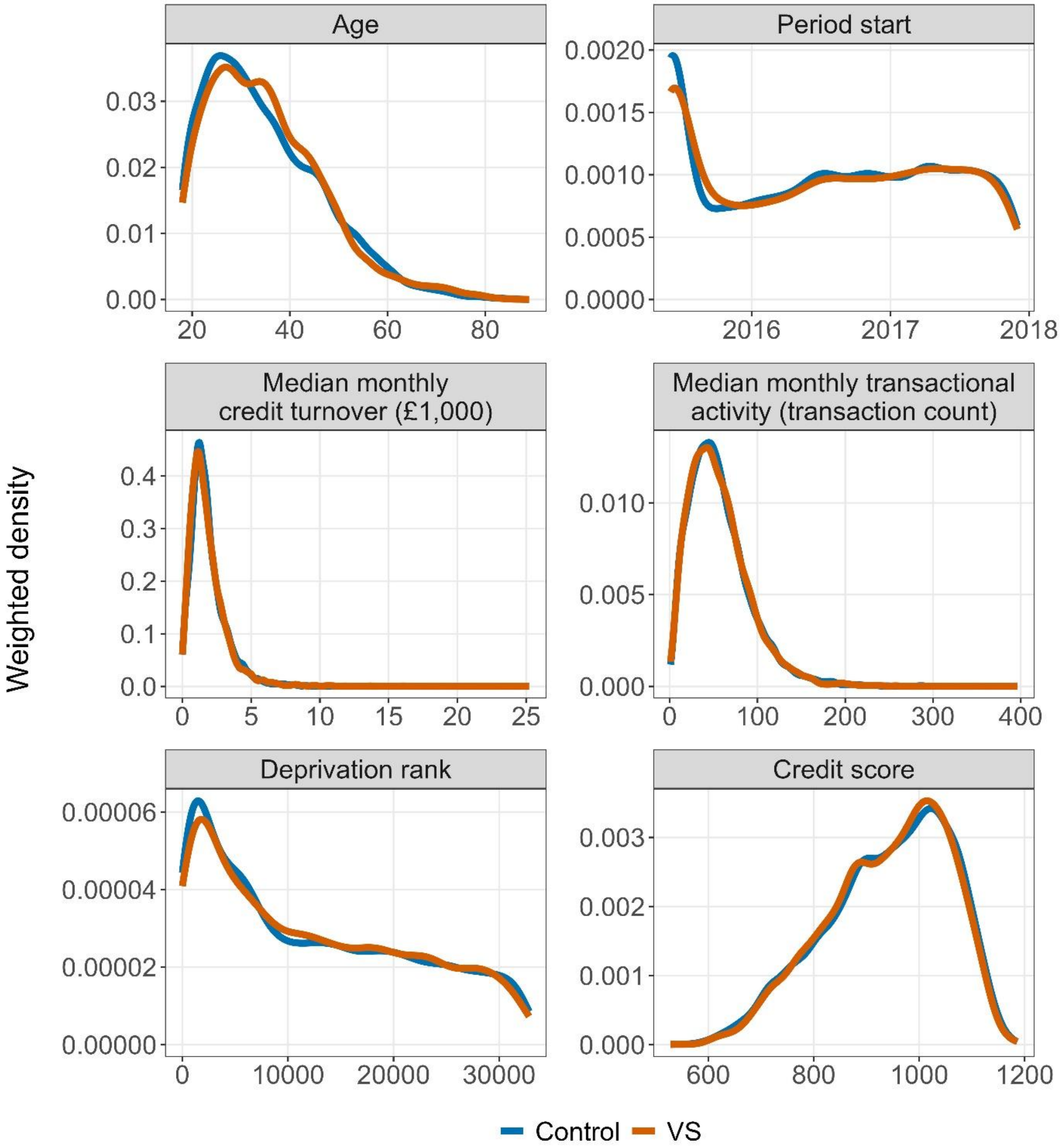

Supplementary Figure 5. Distribution of continuous matching variables in the propensity score matched sample (primary matched sample). Weighted kernel density estimates are shown for the six continuous matching variables used in the propensity score matching for the Control (N = 15,602) and victim-survivor (VS; N = 5,428) groups. Variables comprise age, analysis period start date, median monthly credit turnover, median monthly transactional activity, deprivation rank and average credit score, measured at the start of the analysis period.

measure_type	value	ONS (UK)	LBG	unit
age	16 to 19	0.09	0.00	proportion
age	20 to 24	0.15	0.00	proportion
age	25 to 34	0.18	0.24	proportion
age	35 to 44	0.17	0.34	proportion
age	45 to 54	0.13	0.25	proportion
age	55 to 59	0.08	0.08	proportion
age	60 to 74	0.13	0.07	proportion
age	75 years and over	0.06	0.02	proportion
region	North East	0.04	0.05	proportion
region	North West	0.16	0.13	proportion
region	Yorkshire and the Humber	0.10	0.10	proportion
region	East Midlands	0.09	0.08	proportion
region	West Midlands	0.10	0.12	proportion
region	East	0.13	0.10	proportion
region	London	0.09	0.09	proportion
region	South East	0.13	0.15	proportion
region	South West	0.10	0.11	proportion
region	Wales	0.06	0.06	proportion
disability	Disabled	0.35	0.30	proportion

Supplementary Table 11. Comparison of victim-survivor characteristics between the LBG sample and Office for National Statistics (ONS) estimates. Percentage distributions of age group, region of residence, and disability status for the victim-survivor sample residing in England and Wales (LBG; N = 4,875) and corresponding ONS estimates. Disability in the LBG sample was inferred from disability benefit receipt. For methodological details, see "Representativeness of the VS sample".

variable	group_1	group_2	mean_diff	ci_lower	ci_upper	conf_level	method
Age at start	Final	Filtered	4.85	4.50	5.20	0.95	Bootstrap percentile CI (B=5000)
Credit score difference (end-start)	Final	Filtered	-13.28	-24.18	-2.01	0.95	Bootstrap percentile CI (B=5000)
Deprivation rank difference (end-start)	Final	Filtered	295.89	-46.17	632.63	0.95	Bootstrap percentile CI (B=5000)

Supplementary Table 12. Comparison of included and excluded victim-survivor candidates following application of the filtering criteria. Mean differences between the final victim-survivor sample (N = 5,444) and excluded victim-survivor candidates (N = 4,284) are shown for age at the start of the analysis period, change in average credit score between the start and end of the analysis period, and change in deprivation rank over the same period. Mean differences are accompanied by two-sided 95% bootstrap percentile confidence intervals (5,000 bootstrap resamples). For methodological details, see "Measuring the impact of the filtering criteria".

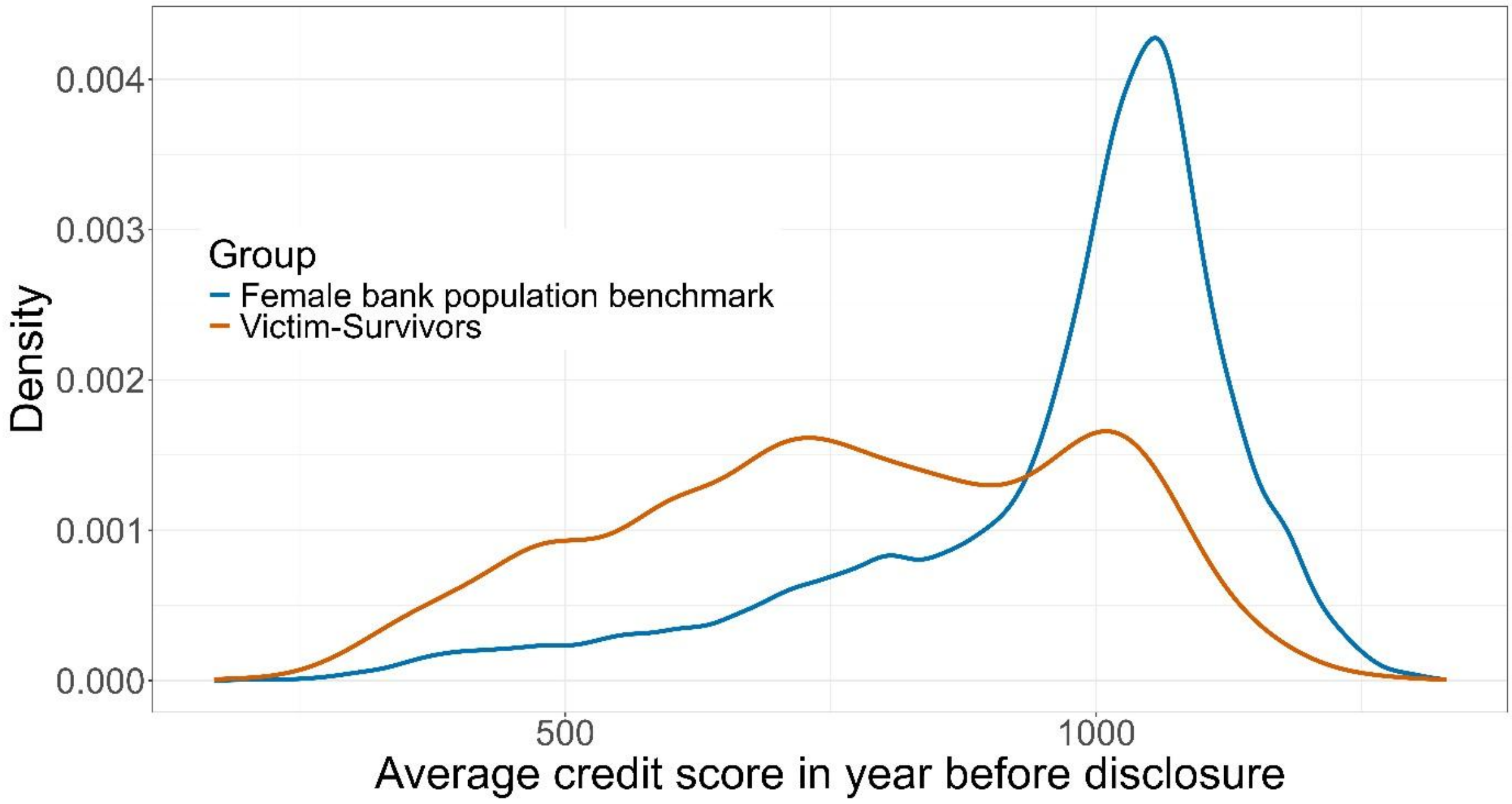

Supplementary Figure 6. Distribution of average credit scores in the year before disclosure for the victim-survivor and Female bank population benchmark samples. Kernel density estimates of average credit scores during the year before disclosure are shown for the victim-survivor sample (N = 5,428) and the Female bank population benchmark (N = 18,906). Credit scores were averaged over the 12 months preceding disclosure (or the corresponding reference date for the benchmark sample). For methodological details, see "Measuring the degree of self-selection by financial distress".
