# Supplementary material for: Banking records reveal characteristics of financial abuse
Source: Nature. 2026 Sep 23;658(8138):1200–7. doi: 10.1038/s41586-026-11049-7 (PMC13716816; doi:10.1038/s41586-026-11049-7)
Supplement: Supplementary file 2 [file 41586_2026_11049_MOESM2_ESM.pdf]

nature portfolio

Corresponding author(s): Anna Trendl

Last updated by author(s): Jul 29, 2026

Reporting Summary

Nature Portfolio wishes to improve the reproducibility of the work that we publish. This form provides structure for consistency and transparency in reporting. For further information on Nature Portfolio policies, see our Editorial Policies and the Editorial Policy Checklist.

Statistics

For all statistical analyses, confirm that the following items are present in the figure legend, table legend, main text, or Methods section.

n/a	Confirmed	
☐	☒	The exact sample size (n) for each experimental group/condition, given as a discrete number and unit of measurement
☒	☐	A statement on whether measurements were taken from distinct samples or whether the same sample was measured repeatedly
☐	☒	The statistical test(s) used AND whether they are one- or two-sided<br>*Only common tests should be described solely by name; describe more complex techniques in the Methods section.*
☐	☒	A description of all covariates tested
☐	☒	A description of any assumptions or corrections, such as tests of normality and adjustment for multiple comparisons
☐	☒	A full description of the statistical parameters including central tendency (e.g. means) or other basic estimates (e.g. regression coefficient) AND variation (e.g. standard deviation) or associated estimates of uncertainty (e.g. confidence intervals)
☐	☒	For null hypothesis testing, the test statistic (e.g. F, t, r) with confidence intervals, effect sizes, degrees of freedom and P value noted<br>*Give P values as exact values whenever suitable.*
☒	☐	For Bayesian analysis, information on the choice of priors and Markov chain Monte Carlo settings
☒	☐	For hierarchical and complex designs, identification of the appropriate level for tests and full reporting of outcomes
☒	☐	Estimates of effect sizes (e.g. Cohen's d, Pearson's r), indicating how they were calculated

Our web collection on statistics for biologists contains articles on many of the points above.

Software and code

Policy information about availability of computer code

Data collection	Data were extracted from the bank's internal secure systems using Teradata SQL Assistant (v.16.20.0.12); the SQL code used for data extraction is commercially sensitive and therefore not publicly available. Requests regarding access to the extraction code should be directed to the corresponding author and are subject to approval by Lloyds Banking Group in accordance with its internal governance and information security requirements. The analysis presented in the study was conducted using R (4.3.0) and Python (3.12.2). The full analytical workflow of this analysis is provided in the accompanying code (https://osf.io/8emyq/overview?view_only=fc3d9c489eb34b878c2da214d24e4388), which generates a model-derived synthetic dataset that preserves the aggregate statistical properties of the original data and replicates the analysis steps used to obtain the main results presented in the paper.
Data analysis	Data analyses were performed using R (v4.3.0) and Python (v3.12.2). Propensity score matching was conducted using the MatchIt package (v4.5.5), and weighted statistical analyses were performed using the survey package (v4.4-2). Temporal cluster analyses were conducted using the glmtools package (v0.2.1) and MNE (v1.7.1). The complete analytical workflow, including code that generates a model-derived synthetic dataset reproducing the aggregate statistical properties of the original data and replicates the main analyses, is available at https://osf.io/8emyq/?view_only=fc3d9c489eb34b878c2da214d24e4388

For manuscripts utilizing custom algorithms or software that are central to the research but not yet described in published literature, software must be made available to editors and reviewers. We strongly encourage code deposition in a community repository (e.g. GitHub). See the Nature Portfolio guidelines for submitting code & software for further information.

Data

Policy information about availability of data

All manuscripts must include a data availability statement. This statement should provide the following information, where applicable:

- Accession codes, unique identifiers, or web links for publicly available datasets
- A description of any restrictions on data availability
- For clinical datasets or third party data, please ensure that the statement adheres to our policy

Access to the raw data is restricted owing to confidentiality, contractual, and regulatory obligations associated with customer banking records. Researchers wishing to enquire about access should contact the corresponding author in the first instance. Any access to the underlying data is subject to approval in line with Lloyds Banking Group's internal governance, data privacy and security processes. To facilitate reproducibility, the accompanying code generates a model-derived synthetic dataset reproducing the aggregate statistical properties of the original dataset used in the study. The code that generates this dataset and reproduces the main results of the study is available here: https://osf.io/8emyq/overview?view_only=fc3d9c489eb34b878c2da214d24e4388.

Research involving human participants, their data, or biological material

Policy information about studies with human participants or human data. See also policy information about sex, gender (identity/presentation), and sexual orientation and race, ethnicity and racism.

Reporting on sex and gender

We used bank records of gender (self-reported by the customer). The sample was restricted to women to due the fact that the overwhelming majority (87%) of financial abuse disclosures were made by women.

Reporting on race, ethnicity, or other socially relevant groupings

The study compared two groups of women: (1) customers who had disclosed domestic or financial abuse to the bank's specialist domestic and financial abuse team (Victim-Survivor group), and (2) propensity score–matched female bank customers with no such disclosure (Control group). Participants were restricted to women because approximately 87% of customers disclosing financial abuse to the bank identify as female. No analyses were performed based on race, ethnicity, or other socially relevant groupings.

Population characteristics

The primary matched sample comprised 5,428 victim-survivors and 15,602 matched controls (total n = 21,030). Participants were women aged 18 years or older at the start of the individual-specific analysis period (mean age 35.3 years in the victim-survivor group and 34.9 years in the control group). Controls were propensity score matched on age, calendar time, credit turnover, transaction volume, deprivation rank, credit score, receipt of selected benefits, and financial product holdings.

Recruitment

We did not recruit participants.

Ethics oversight

Prior to analysis commencing, approval was obtained following review by the bank's Privacy Risk and Impact Assessment (PRIA) governance body. Following an initial review by a Data Privacy Manager and a Data Privacy Risk Specialist, the application underwent an additional review by a Group Data Protection Officer, in accordance with the bank's governance procedures for projects involving data relating to vulnerable customers. The reviewers were assigned according to the bank's standard PRIA governance procedures and were independent of the project team. The PRIA application for this project was submitted in March 2024, and approval was granted in August 2024. The body approved use of the data in line with the project proposal for the publication of research entering the public domain. Customers were informed through the bank's privacy notice51 at account opening that their personal data may be used for behavioral segmentation to better understand customer needs, and that aggregated research findings and insights may be shared beyond the bank. Although the bank's data privacy notice covers the use of customer data for research purposes, customers retained their existing rights to request the erasure of, or object to the processing of, their personal data, and use of their data for research was not a requirement of holding an account.

Note that full information on the approval of the study protocol must also be provided in the manuscript.

Field-specific reporting

Please select the one below that is the best fit for your research. If you are not sure, read the appropriate sections before making your selection.

☐ Life sciences ☒ Behavioural & social sciences ☐ Ecological, evolutionary & environmental sciences

For a reference copy of the document with all sections, see nature.com/documents/nr-reporting-summary-flat.pdf

Behavioural & social sciences study design

All studies must disclose on these points even when the disclosure is negative.

Study description

In this article we perform quantitative analysis on transactional data from a retail bank.

Research sample

Research sample. The primary study sample comprised 5,428 women who had disclosed domestic or financial abuse to Lloyds Banking Group's specialist support service (victim-survivor group) and 15,602 propensity score–matched female bank customers with no such disclosure (control group). Participants were aged 18 years or older at the start of the individual-specific analysis period (mean age 35.3 and 34.9 years in the victim-survivor and control groups, respectively). The sample was restricted to women because

	approximately 87% of customers disclosing financial abuse to the bank identify as female. The control group was selected to match the victim-survivor group on key socioeconomic and financial characteristics measured at the start of the analysis period, enabling comparison of financial trajectories while minimizing baseline differences. The broader Lloyds Banking Group customer base is broadly representative of the UK population across key demographic characteristics (Supplementary Table 1), and the victim-survivor sample is broadly representative of victim-survivors in England and Wales with respect to available demographic characteristics when compared with nationally representative survey estimates (Supplementary Table 11).
Sampling strategy	The victim-survivor sample comprised all eligible women who disclosed domestic or financial abuse to Lloyds Banking Group's specialist domestic and financial abuse team between 2020 and 2024 and met the prespecified inclusion criteria. The control sample was selected by randomly sampling female customers from the wider Lloyds Banking Group customer base, applying the same eligibility criteria, assigning pseudo-disclosure dates sampled from the victim-survivor distribution, and then performing propensity score matching to create a 1:3 matched control group. No formal sample size calculation was performed because this was a retrospective observational study using existing banking records. The sample size was determined by the number of eligible victim-survivors available within the study period, and all eligible individuals were included to maximize statistical precision. A 1:3 matching ratio was selected to increase statistical power while maintaining close covariate balance between groups.
Data collection	The data were provided by LBG.
Timing	Not applicable - the data were not collected by the authors.
Data exclusions	Exclusion criteria were pre-established before analysis. The study was restricted to women because approximately 87% of customers disclosing financial abuse to the bank identify as female. Additional eligibility criteria required participants to be aged 18 years or older at the start of the analysis period, hold a current (checking) account, not hold a joint current account with another individual in the study sample, and have at least six average monthly transactions during each year of the analysis period. Of 9,728 customers who disclosed domestic or financial abuse between 2020 and 2024, 5,444 met these eligibility criteria. Following propensity score matching, the final victim-survivor sample comprised 5,428 individuals. For the control group, an initial random sample of 334,642 female customers was reduced to 45,702 after applying the same eligibility criteria, from which 15,602 matched controls were selected.
Non-participation	Not applicable - we did not recruit participants, which means that no participants dropped out.
Randomization	Participants were not allocated to experimental groups.

Reporting for specific materials, systems and methods

We require information from authors about some types of materials, experimental systems and methods used in many studies. Here, indicate whether each material, system or method listed is relevant to your study. If you are not sure if a list item applies to your research, read the appropriate section before selecting a response.

Materials & experimental systems

n/a	Involved in the study
☒	☐ Antibodies
☒	☐ Eukaryotic cell lines
☒	☐ Palaeontology and archaeology
☒	☐ Animals and other organisms
☒	☐ Clinical data
☒	☐ Dual use research of concern
☒	☐ Plants

Methods

n/a	Involved in the study
☒	☐ ChIP-seq
☒	☐ Flow cytometry
☒	☐ MRI-based neuroimaging

Plants

Seed stocks	Not applicable
Novel plant genotypes	Not applicable
Authentication	Not applicable
