# Supplementary material for: Banking records reveal characteristics of financial abuse
Source: Nature. 2026 Sep 23;658(8138):1200–7. doi: 10.1038/s41586-026-11049-7 (PMC13716816; doi:10.1038/s41586-026-11049-7)
Supplement: Supplementary file 3 [file 41586_2026_11049_MOESM4_ESM.pdf]

nature portfolio

Peer Review File

Banking records reveal characteristics of financial abuse

Corresponding Author: Dr Anna Trendl

Any redactions in this file are there to maintain patient confidentiality, the confidentiality of unpublished data, or to remove third-party material.

This file contains all reviewer reports in order by version, followed by all author rebuttals in order by version.

Version 0:

Reviewer comments:

Referee #1

(Remarks to the Author)
Thank you for the opportunity to review the manuscript "Victim-survivors' banking records reveal the characteristics of financial abuse." It is a privilege to be one of the first to read this critical scholarship.

Summary of key results: This study uses administrative data from a major UK bank to look at patterns in financial behaviors among victim-survivors compared to a matched control group with no abuse disclosure. Findings from this study show that bank records provide a unique opportunity to examine the financial outcomes of victim-survivors and that victim-survivors experience increased debt, depleted savings, more frequent overdrafts, and a range of other financial indicators reflective of intimate partner violence at statistically significantly different rates compared to the control group.

Originality and significance: This study is novel and makes an important contribution to the financial abuse field. Once published, it will be cited frequently.

Data & methodology: The authors use a rigorous strategy for identifying outcomes to focus on, developing a control group, and conducting analyses to look at group differences and longitudinal patterns. Not only is the data quality high, it is also unique. The presentation of findings is thoughtfully done, especially considering the page limits and the amount of data they examine.

Appropriate use of statistics and treatment of uncertainties: Analyses appear appropriate and limitations are documented.

Conclusions: Conclusions are appropriate given the scope of the paper.

Suggested improvements: This is very minor, but could "commuting-related expenses" be clarified? I think these may look different across international contexts and it would be helpful to better understand how it was operationalized here.

References: Appropriately credit previous works.

Clarity and context: The manuscript (including abstract, introduction, and conclusion) are clearly written.

Laura Johnson

(Remarks on code availability)
The link did not seem to open properly for me when I clicked it.

Referee #2

(Remarks to the Author)

A. Summary of the key results

The paper examines retail bank data on customers which have self reported as victims of financial abuse. Using propensity score matching, a control group of similar customers is created using customer details as of 7 years prior to the reporting. The paper then compares trends in the two groups and demonstrates a decline in a number of characteristic outcomes of socioeconomic welfare as well as a set of broad characteristics that appear to characterize financial abuse victims leading up to disclosure that could potentially be used as predictive early warnings for financial institutions.

B. Originality and significance: if not novel, please include reference

The paper reads as reasonably novel and significant to me.

C. Data & methodology: validity of approach, quality of data, quality of presentation

The data quality and presentation seem reasonable to me.

The implementation of the methodology reads as basically correct to me, but I question whether it is appropriate to the stated or intended goals of the paper. The paper reads as having two goals:

1. Predicting the occurrence of financial abuse
2. Demonstrating the socioeconomic impact of financial abuse

If the goal is prediction, which I think is the more important goal as per the introduction, then creating a control sample via propensity score matching followed by monthly outcome comparisons seems inappropriate. It would be much more natural to want to predict the outcome out of the entire population. Some sort of a-priori category sorting may be warranted if the vast majority of eventual victims are from a certain monthly credit turnover bin or geographic deprivation rank, but in the context of prediction, a propensity score match reads as an ill-defined step in prediction. An explicit prediction model would also want to demonstrate the most important characteristics or combination of characteristics for identifying a likely victim from available bank data.

If the goal is demonstrating the socioeconomic impact, then the methodology is okay given the data limitations. However in this case many of the outcomes don't seem relevant to the question of socioeconomic welfare costs. More importantly, the paper should provide some minimal guidance on how to interpret the magnitude of the mean differences.

It seems reasonable that financial abuse would lead to things like overdraft charges, declining consumption, or an increase in unpaid debt. However it is difficult to get a sense of the scale of the problems without comparing them to values from existing research. Alternatively, a comparison to the distribution of outcomes within the sample of all bank customers could provide context to the values. This is important not just for qualifying the overall economic magnitude of any given effect, but in characterizing which specific negative outcomes are the most extreme and therefore most in need of attention by support organizations.

As per the direction of Nature, I have inspected and run the sample code and with small modification it appears to execute as intended, and I don't see any obvious errors with it. Some small suggestions vis-a-vis the code. It's usually best practice to either include the packages in the code repository itself, or to request them from CRAN and PyPi with a specific version so that package updates do not change replicability. The data and output paths should probably also just be specified as a relative reference to the execution directory rather than an absolute path to the host's computer, which would make the code auto-execute without edits. Alternatively, if absolute paths are important, they could be parceled out to an explicit configuration file.

D. Appropriate use of statistics and treatment of uncertainties

The raw statistical treatment seems basically correct to me, modulo the above comments regarding the methodology.

E. Conclusions: robustness, validity, reliability

The nature of the data creates obvious limitations, but they are acknowledged in the text. Given the limited research into this question, and the preliminary nature of the findings, I don't view this as an issue.

F. Suggested improvements: experiments, data for possible revision

Suggestions for improvement primarily relate to my comments on Methodology (C) and Clarity and context (H). I think the research is interesting and valuable, but that the paper should emphasize whether the goal is pre-preemptive prediction of abuse or quantification of the effects of abuse, and clarify the testing and results as giving more specific context to either one.

G. References: appropriate credit to previous work?

I am not well versed in this area, but the reference read as reasonably complete to me.

H. Clarity and context: lucidity of abstract/summary, appropriateness of abstract, introduction and conclusions

The abstract and introduction are okay, but I had a fair amount of confusion about exactly what was meant by a victim-survivor of financial abuse. A lot of text is dedicated to explaining the problems associated with financial abuse, but I felt unclear as to exactly what is being measured. This is critical because it's the actual identifier of the the treatment and control group. How the central identifier comes to exist should be made clear earlier and more directly in the introduction.

Specifically, how does this self-disclosure come to be? Is it voluntary, or is it elicited by the bank? If it's voluntary, what motivates the victim to self-disclose? If it's elicited by the bank, what prompts the bank to ask about financial abuse? How and why does the bank classify a disclosure as financial abuse and record it as such?

Additionally, the structure of the introduction and abstract make it unclear whether the primary takeaway should be one of predicting financial abuse or examining the socioeconomic impact of financial abuse. I understand that the results are meant to be preliminary, but by not committing to one idea or another, or at least cleanly distinguishing the two, it was hard for me as a reader to get a handle on how I should be evaluating the empirical test. It seems from my reading that the primary purpose of the research is to predict abuse from financial data, but the tests lean more towards examining outcomes. Clarifying this would make interpreting the results easier for the reader.

(Remarks on code availability)
I have inspected and run the sample code and with small modification it appears to execute as intended, and I don't see any obvious errors with it. Some small suggestions vis-a-vis the code. It's usually best practice to either include the packages in the code repository itself, or to request them from CRAN and PyPi with a specific version so that package updates do not change replicability. The data and output paths should probably also just be specified as a relative reference to the execution directory rather than an absolute path to the host's computer, which would make the code auto-execute without edits. Alternatively, if absolute paths are important, they could be parceled out to an explicit configuration file.

Referee #3

(Remarks to the Author)
Referee Report for "Victim-survivors’ banking records reveal the characteristics of financial abuse" submitted to Nature

Summary and general assessment of the paper

This paper studies an interesting question, the possible impact of financial abuse on a range of individual financial and non-financial outcomes. Using data from a major UK retail lending institution, the authors investigate the association between individuals’ reported financial abuse and the occurrence of a wide set of outcomes, which range from individual borrowing and expenditures patterns, to credit scores changes, to travel expenses, to reliance on the UK safety net.
This is a policy-relevant issue. While the analysis is potentially interesting and overall presented with care, I have a number of fundamental concerns about the analysis, the methods and the interpretation of the results. As I explain below, because of these relevant limits, in my opinion it is hard to learn sufficiently robust and insightful lessons from the paper.

Comments

1) My first concern regards the possibility of making claims of causal direction based on the descriptive analysis. A reader can be naturally concerned that the indicators of financial distress, impaired (or excess) use of resources, reliance on safety net, etc. are themselves driving conflicts and vulnerability to financial abuse inside households and, hence, causing financial abuse, rather than being induced by financial abuse. The reason we observe an association between credit access (i.e. a low credit score) and financial abuse is that the motivation and incentive to engage in financial abuse inside a household could be more pronounced when an individual is financially weaker inside the household. Similarly, the reliance of an individual on the external safety net could exacerbate or moderate the likelihood that financial abuse occurs.
In a different but related vein, there could be other factors jointly driving a situation of distress and financial abuse, for example.
For all these reasons, while it is interesting to observe associations and descriptive patterns, it's hard to make a step further claiming causality. The analysis is instead presented with the goal of reaching conclusions about the impact of financial abuse on individual outcomes.

2) In many cases the outcomes can be driven by other forms of physical and psychological abuse occurring at the same time as the financial abuse that is the object of interest here. I could not see an attempt to really isolate financial abuse from other forms of abuse, and the reader is then left wondering what is happening in the background. Indeed, in the presentation of the analysis the authors stress how various forms of abuse very often occur simultaneously.
Moreover, in various cases the explanations offered for the effects appear to indeed relate to other forms of abuse, rather

than specifically to financial abuse. It would be important to control as accurately as possible for the possible simultaneous occurrence of other forms of abuse. A change in the use of some type of transportation, or in the reliance on borrowing, can be driven by physical and psychological abuse occurring at the same time as financial abuse, and the resulting need of individuals to resort to external care and support, or to finance medical expenses.

3) I liked the richness of the outcomes considered. At the same time, it's hard to make sense of all the possible effects. In fact, some of the explanations offered are somewhat stretched, and refer somewhat loosely to previous studies. In light of this and also of point 2 above, my suggestion would be to focus the attention on a more limited set of outcomes for which the mechanisms of influence of financial abuse can be clearer and that (point 2) are more tightly related to financial abuse specifically.

4) It would be important to discuss and address much more carefully the possible issues of sample bias. The sample is based on data from a banking institution and, more specifically, a large retail bank. A reader can then wonder to what extent clients of this large retail bank are representative of the population (this problem is briefly acknowledged in the conclusions). An issue of sample selection can also stem from the permanence in the sample. The borrowers who continue to maintain an account rather than exiting the sample or switching to a different bank can be different, for example more or less resilient to financial abuse. This could lead to underestimate or overestimate the association between financial abuse and individual outcomes.

5) Among the various outcomes, I was relatively more intrigued by the financial outcomes, as they might be somewhat more closely connected to financial abuse specifically rather than to other forms of abuse. However, I was somewhat confused by the interpretation of the results for financial outcomes. It appears that borrowers more exposed to financial abuse tend to borrow more but also that they use more cash. Moreover, borrowing and, plausibly, borrowing capacity rise but credit scores tend to decline. Are these interpreted as supply side or demand side effects in the credit market? In most cases there is an attempt to provide a demand-driven rationale, but the actual extension of loans (e.g., credit conditions, costs, rationing) will also likely reflect the status of the individual borrowers. So I would expect quite complex interactions between supply side and demand side effects.

6) It would be interesting to see a more detailed discussion about the generalizability of the results beyond the specific setting considered here. Is there any concern that the structure of families in the UK might affect the link between financial abuse and outcomes? Further analysis of the characteristics of the UK financial system would also be useful, as this structure can affect the link between financial abuse and financial outcomes.

7) What are the possible policy implications of the observed associations, including the possible estimates of the costs of financial abuse for the society at large? What could a financial institution or a policymaker do in order to break down the observed links? I think it would be interesting to at least give a sense of the implications for policymakers and the social costs of the observed mechanisms.

8) The results are sufficiently well presented and described. The abstract and introduction of the paper are sufficiently clear. I have no particular concerns on these fronts. Perhaps, I would suggest highlighting the nature of the data used and their limits earlier in the introduction, and with more detail. Moreover, the paper makes reference to the previous literature in a clear way.

(Remarks on code availability)
The code files appear to be in good order and the instructions well written

Referee #4

(Remarks to the Author)
A. Summary of the Key Results
This paper seeks to demonstrate that financial data can be used to construct risk indicators for financial abuse—an issue that accompanies domestic abuse in approximately 95% of cases.
Drawing on data provided by one of the United Kingdom’s largest financial institutions (Lloyds Banking Group), the authors compare the financial behavior of a general sample with that of a cohort of women who have self-identified as victim-survivors of financial abuse, over a seven-year period. From an initial set of more than 400 indicators, 171 variables showing statistically significant differences were retained for analysis.
The results reveal significant disparities between the two groups across multiple domains: higher levels of debt and overdraft use among victim-survivors, a decline in savings, distinctive spending and cash withdrawal patterns, and increased interactions with banking services.
The paper concludes by arguing that banking data can serve as a valuable tool for the prevention of domestic abuse, as key behavioral differences can be systematically observed. Such data could be used not only to detect financial abuse but also to inform policy interventions designed to help victim-survivors regain financial stability—for instance, through improved credit scoring methodologies.

B. Originality and Significance
The topic addressed in this article is of considerable importance. It highlights the financial dimension of domestic violence—crucial both for identifying early warning signs and for understanding the economic dynamics that entrap women in abusive relationships, as well as the long-term economic consequences they face.

A further contribution of the paper lies in demonstrating that banking data represent an invaluable resource for detecting situations of risk.
While the notion that banks should engage with the issue of financial abuse is not entirely new—see, for example, Ayesha Scott's Financial Abuse in a Banking Context: Why and How Financial Institutions Can Respond (Journal of Business Ethics, 2023)—Scott's work adopts an ethical perspective and relies primarily on qualitative data.
The originality of this research lies in its use of quantitative data and in its effort to design empirical indicators. Moreover, thanks to the longitudinal character of the banking data, the authors are able to reconstruct seven years of financial history for the participants, which allows for a temporally grounded analysis.

C. Data and Methodology: Validity of Approach, Data Quality, and Presentation
A central methodological issue concerns the data on victim-survivors, which originate from within the bank itself. Although the authors acknowledge limitations regarding their sample, the implications of these limitations for the validity of the findings are not sufficiently examined.
In particular, the first limitation—that only women who contacted the bank's Specialist Domestic and Financial Abuse Team are included—deserves closer scrutiny. It would be essential to assess the representativeness of this group: what proportion of the bank's female clientele does it represent, and how does that compare to the estimated prevalence of financial abuse in the wider population? Are there demographic, geographic, or socioeconomic specificities within the sample?
Given the centrality of this issue, a more detailed description of the Domestic and Financial Abuse Team should be integrated into the main text, not confined to the methodological appendix. How do potential victims become aware of this service? What forms of assistance does it provide? Are individuals encouraged to contact it in cases of domestic abuse even when financial abuse is not explicitly present?
A major concern—one acknowledged but not fully explored by the authors—is the potential selection bias whereby only individuals experiencing severe financial consequences are likely to contact this service, thereby skewing the sample.
Furthermore, the analysis would benefit from considering whether, in some cases, financial distress may have preceded the abusive relationship and contributed to victims' vulnerability, or conversely, whether financial hardship triggered recognition of the abuse and disclosure to the bank.
Another limitation concerns the use of the disclosure date as a pivotal temporal marker. This date likely encompasses a variety of situations: some women may have long been separated and are seeking to rebuild financial stability, while others may still be cohabiting with their abuser and under ongoing coercive control. Their financial behaviors are therefore not directly comparable.
Finally, the treatment of banking data appears to assume that each participant holds a single account with Lloyds. Yet many may hold multiple accounts or loans with other institutions. These factors—central to financial life—are not accounted for in the analysis.

D. Appropriate Use of Statistics and Treatment of Uncertainties
The study's approach—testing a large number of indicators independently—is useful as an exploratory step. However, the authors refrain from identifying broader behavioral patterns or developmental stages that could be operationalized by banks. Each variable is tested separately, and the paper does not propose an analytical model linking these dimensions, which limits the robustness and interpretability of the findings. A cluster or typology analysis could have revealed recurrent combinations of behaviors among victim-survivors, potentially forming the basis for automated detection systems.
As it stands, this remains an initial stage of inquiry. There is an implicit suggestion that the study could directly yield predictive indicators, whereas substantial further work would be required to achieve this goal. Moreover, no correlation analyses are performed; as a result, the explanations of observed behaviors remain tentative and largely hypothetical.

E. Conclusions: Robustness, Validity, and Reliability
The article's conclusions are appropriately cautious: it emphasizes that banking data hold promise for addressing violence against women and should be more systematically leveraged in the future. These conclusions are robust, albeit modest in scope.
Beyond these general claims, however, the paper also presents more specific results whose reliability is weaker, due to the methodological limitations outlined above.

F. Suggested Improvements: Additional Data and Possible Revisions
To strengthen the findings, it would be valuable to include a comparison group of women experiencing financial hardship—manifested in high debt levels and reduced savings—but without exposure to domestic or financial abuse. This would help isolate the behaviors specifically associated with financial abuse.
In addition, alternative temporal markers beyond the "disclosure date" should be explored to identify key stages in financial exploitation and to test their regularity.
Finally, the question of causality warrants deeper investigation. Qualitative research, particularly life-history interviews, would be indispensable for understanding the differentiated financial behaviors highlighted in the article and the motivations underlying them.
At a minimum, the article suggests that financial distress may stem from two sources—first, the direct exploitation by an abusive partner, and second, the costly and complex processes involved in leaving the abusive situation. Distinguishing between these mechanisms is crucial for designing appropriate support measures.

G. References: Adequate Acknowledgment of Previous Work
The article could engage more extensively with existing qualitative research on financial abuse, including for instance the work of Sundari Anitha, E. Branigan, and S. Singh.
Moreover, while the study focuses on the UK context, it would benefit from greater international perspective. Relevant research has been conducted in other countries, such as Australia and India, and should be cited to situate the contribution

within the broader field.

H. Clarity and Context
The manuscript is well-written, and the abstract, introduction, and conclusion accurately reflect the content and objectives of the study. Overall, the paper is clearly structured and accessible, even when dealing with complex methodological issues.

(Remarks on code availability)

Referee #5

(Remarks to the Author)
Using a matched case-control design, this study examined the evolution of a set of financial behavioral indicators (e.g. financial balances, spending patterns, account management) derived from banking data in the seven years prior to disclosure of financial abuse to bank personnel.
Compared to participants in the control group, victim-survivors had depleting savings and increasing debts over the seven years before reporting financial abuse to the bank, leading to missed payments and reduced credit scores. Participants in the victim-survivor group also showed relatively reduced expenditure on items related to self-care, transportation, and legal costs, increased cash withdrawals, more frequently changed addresses and passwords, and a higher likelihood of claiming welfare benefits. The authors conclude that results demonstrate how victim-survivors lose financial independence and stability. They also point out that banking data represents a fruitful new avenue for early identification of financial abuse.

I believe that the results presented in this manuscript will be of wide interest to an interdisciplinary group of scholars of intimate partner violence generally and economic abuse in particular. Exploring financial abuse through banking data is a creative and ambitious idea and the data set is unique in allowing for the investigation of survivors' evolving finances through objectively inferred data collected in a large sample. The authors controlled for socio-economic confounds, enabling a sharp comparison between the control and comparison group; and they were able to analyze a very large number of financial variables, creating a broad and deep picture of survivors' finances over time. As far as I know, this is the first quantitative and objective study of financial abuse's impact on survivors' everyday lives and represents an important leap in the study of this topic. The manuscript itself is well-written and the data is, for the most part, well presented.
Nonetheless, the manuscript could be improved, and here I offer several suggestions for doing so, most notably related to the questions of causality and disclosure process. (Note that none of these suggestions touch on the statistical analyses used, as this is not my area of expertise.)

Abstract:

The abstract is clear and direct. However, as I will elaborate below, the study design does not allow for causal inferences and this should be made crystal clear upfront in the abstract.

Introduction/Methods:

The authors provide appropriate citations throughout this section but I would suggest adding some of the key references in this area, including the following:

Stylianou, A. M. (2018). Economic abuse within intimate partner violence: A review of the literature.
Violence & Victims, 33(6), 983–1001.

Johnson, L., Chen, Y., Stylianou, A., & Arnold, A. (2022). Examining the impact of economic abuse on survivors of intimate partner violence: A scoping review. BMC Public Health, 22, 1262.
Postmus, J. L., Hoge, G. L., Breckenridge, J., Sharp-Jeffs, N., & Chung, D. (2020). Economic abuse as an invisible form of domestic violence: A multicountry review. Trauma, Violence, & Abuse, 21(2), 261–283.

Adams AE, Littwin AK, Javorka M. (2020). The frequency, nature, and effects of coerced debta among a national sample of women seeking help for intimate partner violence. Violence Against Women, 26(11):1324-1342.

Line 38: "Financial abuse is a subcategory of economic abuse…." It would be useful to define and elaborate a bit on economic abuse as well as on how economic abuse often fits within a broader pattern of coercive control. This would allow the reader to understand financial abuse in a more comprehensive way.

Line 80: Here, the authors introduce the idea of survivor disclosures. Although they describe the disclosure process in the Methods section, without a fuller description here, I was confused as I continued to read.

Also, in this first description of disclosure, it would be useful to make explicit that disclosure recipients did not document length of time since the start of the abuse. This should also be named in the Limitations section.

In the Methods section, Line 284, the authors provide more detailed information on the disclosure process, but this information, in turn, raised concerns that overlap with the question of causality, described next: In my reading, it sounds like participants disclosed the financial abuse in the hopes of receiving some sort of support. How might a potential reward for

disclosure affect who is in the victim-survivor group? At the margin, does financial insecurity raise the likelihood of reporting abuse to the bank? Is it possible, for example, that only those with the most severe financial abuse reported it? (Conversely, it is possible that those with higher abuse were more afraid to disclose the abuse). These interpretations should be discussed more deeply in the Limitations section.

Line 83: This is the first place the authors discuss their use of a retrospective design to examine the “impact of financial abuse on victim-survivors’ financial wellbeing over time.” As I began to note in the above comment about the disclosure process, here and elsewhere, the authors imply a causality they cannot establish empirically. Their retrospective design allows for various interpretations of the findings. For example, bad financial luck or poor financial decision-making can set in motion a woman’s entry into a relationship where she has relatively little power simply out of financial desperation, setting the stage for abuse. In this interpretation, it is the financial downturn that predicts the abuse, not the other way around. In other words, the authors sometimes make leaps in interpreting their findings and should offer clear alternative explanations, probably in the Limitations section.

Figures:

Figure 1: I like these panels as a way to visualize complex data. It might be useful to add labels or units along the vertical axis for easier comprehension

Figure 3: It would be easier to compare Panels A and B if the categories along the vertical axis were in the same order.

Discussion:

The limitations section lays out well some of the challenges in interpreting the results of the study. I think these should be elaborated – particularly the two I have focused on in this review - so that the authors have a chance to explore alternative interpretations of the data more fully.

(Remarks on code availability)

Version 1:

Reviewer comments:

Referee #1

(Remarks to the Author)
Thank you for the opportunity to review the revised draft of this manuscript, focused on victim-survivors banking records. As I noted in my original review, this study provides a unique and valuable insight into survivors’ financial abuse experiences viewed through their banking patterns.

I think the authors did an excellent job of addressing reviewer feedback. While this type of statistical analyses is not my expertise, the the findings and implications are clear.

The authors removed causal language, instead emphasizing associations found within the data. The addition of the sensitivity analysis, particularly by repeating the analysis with the distress-matched sample, helps to isolate the behaviors associated with intimate partner violence survivorship, as the authors note in their response.

With regard to the comment “seemingly conflicting finding that victim-survivors simultaneously increase their borrowing and experience declining credit scores” – I appreciate that the authors used credit application data to provide further context. However, these findings also seem reasonable to me since increasing borrowing does have the potential to decrease credit score.

In my reading of the revised draft, it occurred to me that the limitations section might be the first time that the manuscript really explicitly states the gender of victim-survivors in the sample (“First, the victim-survivor group, comprising of women in the UK with bank accounts…”) Because of this, the use of the female bank population benchmark also seemed to be missing context. Perhaps the gender of the sample could be noted more clearly earlier in the paper. Also, was this a specific sampling choice or did only women utilize the financial and domestic abuse team?

I appreciate the authors’ decision to include both financial and non-financial outcomes and agree they are relevant to providing context to the various domains impacted by abuse.

Overall, the discussion of study limitations is much more robust.

(Remarks on code availability)
I was able to access the code, however, I did not attempt to open it because I do not use R.

Referee #2

(Remarks to the Author)
The authors have been responsive to my concerns. The reframing of the paper from a predictive exercise to a descriptive characterization of financial abuse in banking data resolves the methodological tension I identified in Round 1 between the stated goals and the PSM design. The bank population benchmarks now provide the effect-magnitude context I requested, and the disclosure process is adequately explained. Code reproducibility improvements have been implemented. I am satisfied that my comments have been addressed.

Malcolm Wardlaw

(Remarks on code availability)

Referee #3

(Remarks to the Author)
I appreciated the revision and I would like to thank the authors for their work. While the paper cannot establish causality, the authors now make clear the scope of this work and more cautiously refer to associations. This is useful. I also appreciated the careful work in documenting the external validity of the analysis and in defending the representativeness of the sample.

I have one remaining comment that bundles together two comments in my previous report. The authors argue that the various forms of abuse occur more or less at the same time, so it is indeed very difficult to separate the specific effects of financial abuse. At the same time, in the revised version they keep on focusing on the same very wide range of outcomes, with no particular priority in their discussion in the text. Given the difficulty of properly controlling for forms of non-financial abuse, I find this strategy of presenting such a wide range of outcomes not particularly convincing. It can be natural for a reader to tie, say, the use of particular types of transportation to forms of physical or psychological abuse, rather than primarily to financial abuse. In the absence of a clear distinction among the effects of various types of abuse, presenting on the same level such a wide range of outcomes can weaken the message and raise a reader's concerns about omitted variables issues.

It's of course up to the Editor's judgement, but my recommendation is then (in a minor revision) to focus more on financial and economic outcomes that can be more closely linked to financial abuse and reduce the emphasis on outcomes that are far away from financial abuse. Such additional outcomes could be presented and discussed in a somewhat shorter way in the text, as additional outcomes, with related figures given less priority. This would also allow to better clarify the comments on more salient outcomes and instead shorten the comments on outcomes and mechanisms that are less clearly related to financial abuse and that currently appear to be disconnected from the core of the paper.

(Remarks on code availability)
The code appears to be in good order.

Referee #4

(Remarks to the Author)
The authors have carried out a thorough and rigorous revision in this second version and have addressed the comments I previously raised, in particular regarding the representativeness of the sample, the possibility of constructing a cluster of variables, and the comparison with individuals experiencing financial distress who are not victims of violence. The inclusion of the suggested references should also be noted.

I am therefore in favor of the publication of this article in its current form, and I would like to reiterate the importance of the topic and the originality of the approach, which I hope will inspire further research.

Jeanne Lazarus

(Remarks on code availability)

Referee #5

(Remarks to the Author)
Thank you for the opportunity to review the revised version of this important manuscript. I believe that the authors did a tremendous job responding to the many concerns expressed by the five reviewers. They have certainly addressed my own suggestions.

The only thing I want to suggest now is that the authors review the manuscript one more time for language that implies causation. For example, this revised line still implies causality with the word “shapes.” Current evidence on how financial abuse shapes the everyday experiences of its impact on victim-survivors’ lives survivors is drawn primarily from small-scale, qualitative surveys.” It would be good to shift to words like “is related to” here and possibly elsewhere.

Otherwise, I think this will make a terrific contribution.

Best, Lisa Goodman

(Remarks on code availability)
--

Version 2:

Reviewer comments:

Referee #2

(Remarks to the Author)
==
FIELD 1 — "Remarks on Code Availability" (transmitted to authors)
==

I reviewed the code and supporting materials deposited in the OSF repository associated with this submission, and I ran the analysis pipeline end-to-end on the simulated data. The data underlying the paper is confidential banking data and cannot be shared, which is appropriate; the authors instead provide a self-contained pipeline that generates simulated data preserving the relevant statistical properties (means and standard deviations) of the original, together with the parameter files needed to do so. The analysis is therefore fully demonstrable without access to the confidential source data.

The renv environment restored cleanly, the dependencies installed without incident, and all five steps executed in the documented order and ran to completion. They produce the full set of outputs mapped in the README: Figure 1 from the main analysis, the propensity-score balance tables and figures (Supplementary Tables 7-9, Figures 4-6), the temporal-clustering results, and Figures 2-3. The propensity-score matching achieves good covariate balance on the simulated sample, with all post-match standardized mean differences falling below roughly 0.06. I am satisfied that the analysis is reproducible from the materials provided. Because the demonstration runs on simulated data and at reduced permutation and subsample settings, the figures reproduce the structure and qualitative behavior of the published results rather than their exact magnitudes, which is the appropriate and expected standard for a study built on confidential data.

The code is clearly organized and well documented. The five-step structure is logical, the README maps each output to its corresponding manuscript figure or table, and pseudocode.md gives a readable account of the analytical logic. I note in particular that the two concerns I raised in the first round have been resolved: the absolute file paths have been removed in favor of paths relative to the execution directory, and the R package environment is now fully version-locked via renv.lock. Both changes materially improve reproducibility.

I have a small number of minor packaging issues that the authors should correct, none of which affects the validity of the analysis:

1. The README "Quick Start" instructs reviewers to run setup.sh and then run_analysis.sh, but neither script is present in the repository; only the individual step scripts are included. The authors should either add the two wrapper scripts or update the README to point solely to the step-by-step instructions.

2. The README refers to the final visualization script as step_5_visualise_temporal_pattern.R, but the file in the repository is named step_5_temporal_pattern_viz.R. The names should be reconciled so the documented sequence can be run as written.

3. The simulated demonstration dataset distributed with the submission (sample_data_2_pct.csv.gz) does not appear to be read by any script in the pipeline, which generates its own simulated data internally from the parameter files in inputs/. The authors may wish to either wire this file into the demo or clarify its intended role to avoid confusion.

4. A compiled Python artifact (__pycache__/functions.cpython-313.pyc, built under Python 3.13) is included although the stated requirement is Python 3.10 or later. This is harmless but should be removed from the distribution.

5. The R environment is pinned via renv.lock, but the Python dependencies in requirements.txt are specified only with lower bounds (>=). Because the clustering step imports a non-public function from MNE (mne.stats.cluster_level._find_clusters), which is more likely than the public API to change across releases, I would encourage pinning the Python dependencies to exact versions as well to protect long-term replicability.

6. A global seed is set, but several stages draw fresh stochastic samples during execution, so the demonstration is not fully deterministic run-to-run (for example, the number of significant clusters varies slightly between executions). This does not affect the validity of the demonstration, but seeding each stochastic stage would make the example exactly reproducible and is a small, worthwhile addition.

Overall, the data availability statement is appropriate and the code is in good order. Having run the pipeline to completion and confirmed it reproduces the documented outputs, I am satisfied that the reproducibility concerns from the previous round have been adequately addressed. The remaining items above are minor packaging fixes that can be handled at the copy-editing stage.

(Remarks on code availability)
Regarding the execution shell scripts setup.sh, and run_analysis.sh. I have written a working version of them in trying to assemble the code flow. This worked for me on MacOS GNU bash, version 3.2.57.

```
#!/usr/bin/env bash
#
# setup.sh
#
# Restores the locked R environment (renv) and builds a Python virtual
#
# Usage:
# bash setup.sh
#
# Notes:
# - The R restore can be slow the first time: renv builds pinned package
#
set -euo pipefail
cd "$(dirname "$0")"

# --- R environment -------------------------------------------------------
command -v Rscript >/dev/null 2>&1 || { echo "ERROR: Rscript not found on PATH." >&2; exit 1; }
echo ">>> Restoring R environment via renv (this may take a while on first run)..."
Rscript -e 'if (!requireNamespace("renv", quietly = TRUE)) install.packages("renv", repos = "https://cloud.r-project.org");
renv::restore(prompt = FALSE)'

# --- Python environment --------------------------------------------------
echo ">>> Building Python environment from requirements.txt..."
if command -v uv >/dev/null 2>&1; then
uv venv .venv
uv pip install --python .venv -r requirements.txt
else
command -v python3 >/dev/null 2>&1 || { echo "ERROR: python3 not found on PATH." >&2; exit 1; }
python3 -m venv .venv
# shellcheck disable=SC1091
source .venv/bin/activate
python -m pip install --upgrade pip
python -m pip install -r requirements.txt
deactivate
fi

echo ">>> Setup complete. Python venv is at ./.venv"
echo ">>> Next: bash run_analysis.sh"
```

```
#!/usr/bin/env bash
#
# run_analysis.sh — execute the full analysis pipeline in order.
#
# Runs steps 1–5 against the simulated data and writes all figures and
# tables
#
# Usage:
# bash run_analysis.sh
#
set -euo pipefail

# Always run from the directory this script lives in, so the scripts'
# relative paths (inputs/, helpers/, outputs/) resolve correctly.
cd "$(dirname "$0")"
```

```
# Required tooling.
command -v Rscript >/dev/null 2>&1 || { echo "ERROR: Rscript not found on PATH." >&2; exit 1; }
command -v jupyter >/dev/null 2>&1 || { echo "ERROR: jupyter not found on PATH." >&2; exit 1; }

run_r () {
echo ">>> [$1/5] Rscript $2"
Rscript "$2"
}

echo "=== Pipeline start: $(date) ==="

run_r 1 step_1_setup.R
run_r 2 step_2_psm.R
run_r 3 step_3_main_analysis.R

echo ">>> [4/5] jupyter nbconvert --execute step_4_clustering.ipynb"
jupyter nbconvert --to notebook --execute --inplace --ExecutePreprocessor.timeout=-1 step_4_clustering.ipynb

run_r 5 step_5_temporal_pattern_viz.R

echo "=== Pipeline complete: $(date) ==="
echo "Outputs written to: $(pwd)/outputs/"
```

Referee #3

(Remarks to the Author)
The authors have substantially satisfied my recommendations. Thank you for your careful work

(Remarks on code availability)
The code looks satisfactory to me

Open Access This Peer Review File is licensed under a Creative Commons Attribution 4.0 International License, which permits use, sharing, adaptation, distribution and reproduction in any medium or format, as long as you give appropriate credit to the original author(s) and the source, provide a link to the Creative Commons license, and indicate if changes were made.
In cases where reviewers are anonymous, credit should be given to 'Anonymous Referee' and the source.
The images or other third party material in this Peer Review File are included in the article's Creative Commons license, unless indicated otherwise in a credit line to the material. If material is not included in the article's Creative Commons license and your intended use is not permitted by statutory regulation or exceeds the permitted use, you will need to obtain permission directly from the copyright holder.

To view a copy of this license, visit https://creativecommons.org/licenses/by/4.0/

Revision of Nature manuscript 2025-09-23824

“Victim-survivors’ banking records reveal the characteristics of financial abuse”

Thank you for offering us the opportunity to undertake a revision of this paper for publication in Nature. Please see the original decision letter and reviewer comments enclosed below in italics, with our responses shown in plain type. Please also find enclosed our revised manuscript with tracked changes and line numbers added, which we reference below.

Editor Letter

Dear Dr Trendl,

Your manuscript, "Victim-survivors’ banking records reveal the characteristics of financial abuse", has now been seen by five referees, whose comments are attached below. While they find your work of potential interest, as do we, they have raised important concerns that in our view need to be addressed before we can consider publication in Nature.

In particular, reviewers raise important concerns in relation to the causal claims and associations done, the potential bias of the data, and their limitations. We are interested in a new version of the paper that clearly demonstrates that the data is valid internally and externally and that the methods are appropriate to threat them.

Thank you for your guidance on these points. Please see our in-line responses to the six points you detail below. Please note that references to line numbers in this document refer to the revised version of the manuscript with tracked changes switched on (manuscript_revised_tracked.docx). In our responses, strikethrough text refers to text that has been removed and text in bold refers to text added in the revised version.

We therefore invite you to revise your manuscript so that it:

-Clarifies the methodological decisions that would impact goal attainment and suitability of the methods (R2).

Thank you for this important feedback. Reviewer 2 requested that we clarify whether the goal of the paper is 1) predicting the occurrence of financial abuse, or 2) demonstrating the socioeconomic impact of financial abuse. We have revised the manuscript to clarify that the primary goal of the analysis is to illustrate victim-survivors experience of financial abuse using banking data.

Specifically, we have made the following changes:

Abstract:

- *“The ~~effects~~* ***experience*** *of financial abuse, a form of domestic abuse involving control over money and finances, can be severe and long-lasting, yet difficult to document. Current evidence* ***on how financial abuse shapes the everyday experiences*** *of ~~its impact on~~ victim-~~survivors’ lives~~* ***survivors*** *is drawn primarily from small-scale, qualitative surveys.”* Lines 30-33.

- *"Our results ~~show~~ **demonstrate** how victim-survivors lose financial independence, **experience economic instability**, and ~~stability, and provide preliminary evidence that banking data could underpin a data-driven approach to mitigating foreseeable harm through early identification~~ suffer financial distress."* lines 43-44.

Introduction:

- *"However, the potential of financial data for understanding ~~the impact~~ **victim-survivors experience** of financial abuse remains largely untapped."* lines 133-134.
- *"In addition, financial data allows us to follow the same individual over time, capturing ~~the evolving impact of economic abuse on~~ **how financial abuse is associated with changes in** victim-survivors' financial lives."* Lines 146-148.
- *"The matched-design approach ~~controlled~~ **controls** for socio-economic confounds, ~~allowing us to characterize the impact~~ **, enabling the comparison** of ~~financial abuse on~~ victim-survivors' financial wellbeing **with controls** over time. The outcomes analyzed here span a wide range of behaviors, including financial balances, spending patterns and account management~~,~~**. To our knowledge, this is** ~~providing~~ the first empirical illustration of **the experience of** financial ~~abuse's impact~~ **abuse** ~~on~~ **in** victim-survivors' everyday lives **using banking data**.* Lines 166-172

Discussion

- *"To our knowledge, ~~the present study~~ **this** is the first ~~to draw on~~ **study using** administrative, objective data ~~sources~~ to ~~document the impact of~~ **describe how** financial abuse ~~on~~ **manifests in** victim-survivors' lives"* Lines 458-459
- *"Despite these limitations, this study shows that financial data provides a valuable lens to understand the ~~impact~~ **experience** of financial abuse. The results presented in this study indicate that banking data ~~has the potential to underpin the~~ **data could guide future efforts to** develop~~ment of risk~~ **vulnerability** indicators and intervention strategies **designed** to ~~prevent harm and~~ support victim-survivors."* lines 550-554

-Refines the causal analyses, or, if the advance is correlational, makes the necessary adjustments to the claims (R2-5).

We are grateful to Reviewers 2, 3, 4, and 5 for highlighting where our original language has implied causal links. We recognize that while our methodology identifies robust associations, it does not establish causality. Consequently, we have revised the manuscript to ensure our results are framed as associative and descriptive rather than causal or predictive.

Beyond clarifying the paper's scope, we have systematically updated the terminology throughout the manuscript – as detailed below – to ensure our analysis is presented as a descriptive characterization of the data rather than a causal claim.

Results:

- *"To ~~identify~~* ***define*** *the subset of 373 indicators ~~characterizing the effects of~~* ***reflecting differences associated with*** *financial abuse, we first identified"* lines 175-177
- *"~~Decreased spend~~* ***Decreased spending*** *on self beyond basic necessities ~~may reflect the impact of~~* ***aligns with patterns of*** *financial control ~~– either through restriction tactics or exploitation leaving victim-survivors with less money via increased borrowing~~* ***documented in prior studies, including restricted access to funds and financial exploitation, both of which have been linked to reduced financial autonomy among victim-survivors"*** lines 273-276
- *"Higher spending in off-licence stores is consistent with prior evidence showing that victim-survivors may use alcohol as a coping strategy~~, which may even be encouraged by the abuser to increase control~~"*-lines 2812-284
- *"The pattern of increased cash usage ~~might reflect~~* ***is consistent with a range of experiences reported in the literature, including*** *financial exploitation through unauthorized or forced cash withdrawals aiming to deplete the victim-survivor's resources"* lines 307-310
- *"Lower commuting-related spending in the VS group ~~is in line with earlier results showing lower likelihood of employment and could result from sabotaging employment, while increased petrol spend may signal the victim-survivor's attempts to maintain safety and access support, which may involve longer journeys. Access to taxis can provide a quick and discreet emergency escape option particularly when shared vehicles are being tracked by the abuser, and an analysis of escape journeys of women found that taxis were the second most common option after transport provided by friends and family.~~* ***aligns with earlier findings of lower employment prevalence among victim-survivors. Higher spending on flexible transport options, including cars and taxis, has been reported previously and may reflect the complex, constrained circumstances in which victim-survivors meet everyday needs, including limited alternatives, time pressures and safety considerations."*** lines 319-323
- *"All of these activities ~~may be linked to the abuser taking control of bank accounts or victim-survivors seeking to take back control~~* ***reflect changes in account access and management consistent with those reported in situations involving financial abuse, including both shifts in control by an abuser and increased engagement by victim-survivors*** *post-separation, when it may be safe to do so. Frequent ~~address changes are likely to reflect separation and the victim-survivors' efforts to prevent the abuser from discovering their new location~~* ***changes of address align with the residential instability often associated with separation"*** lines 333-337

Discussion

- *"**Observed** changes in cash usage, transport spend**ing**, and account management activity ~~may reflect victim-survivors' efforts to escape their abuser~~* ***are consistent with patterns reported around periods of safety-seeking or separation among victim-survivors.****"* Lines 500-502

- *"~~The results presented here, while descriptive, offer preliminary evidence for the feasibility of developing risk indicators that could underpin proactive support strategies and minimize the risk of foreseeable harm.~~"*
- We also added the following limitation: "***our methodological approach does not allow us to establish causality between financial abuse and observed outcomes. For example, there may be two-way relationship between financial abuse and financial distress, or factors jointly driving abuse and distress.***" lines 544-546

-Addresses the limitations of the data given the sensitivity of the topic (R4-5).

In response to the requests from Reviewers 2, 3, 4 and 5 to provide greater clarity about the construction of the victim-survivor sample, in the revised version we provide a more detailed description of how victim-survivors come to disclose their experiences to the bank in the introduction (lines 152-161): *"**Victim-survivors self-disclosed their experiences to the bank's specialist financial and domestic abuse team (VS group; N = 5,428). Bank staff are trained to identify potential vulnerabilities, including financial and domestic abuse, during routine interactions (e.g., querying transactions, clarifying charges). When staff judge that a customer may have experienced domestic or financial abuse and might benefit from further support, they offer a referral to the bank's specialist team. With consent from the customer, the specially trained team contacts the customer to understand more about the nature and experience of abuse and, where possible, provide personalized support. The most common forms of support provided are separation of joint accounts, temporary suppression of charges, and signposting to relevant charities.**"*

In response to Reviewer 4's suggestions, we have conducted two additional robustness analyses to address concerns regarding financial distress and separation timing.

First, by repeating the analysis using a distress-matched sample, we isolate the behaviours specifically associated with being a victim-survivor, controlling for the broader experience of financial hardship. Second, incorporating a relationship-end date sample allows us to determine the extent to which our primary findings are specific to the post-separation period.

These findings are now detailed in a new subsection within the Results section (lines 354–416):

"Replications for alternative temporal markers and comparison groups. To test for the sensitivity of the results, we repeated the previous analyses using two alternative samples. First, we created a distress-matched sample, pairing victim-survivors (N = 5,183) with controls (N = 13,430) who experienced similar levels of financial distress in the year before disclosure. This allows us to separate patterns associated with abuse from those related to financial distress. Second, we created a smaller relationship-end matched sample that included victim-survivors where information on relationship end date was available (N = 1,149) and corresponding matched controls (N = 3,236; 21% of the primary matched control sample). By comparing VS-Control differences at relationship end, rather than disclosure date, we can examine the extent to which the patterns reported are specific to the pre-separation period. Aside from defining the individual analysis period using the relationship end date, we followed the same matching procedure as in the primary sample. Supplementary Table 2 presents descriptive statistics for the VS and Control groups.

Results from the distress-matched sample (Supplementary Figure 2, Supplementary Table 5) show that a close match is achieved for financial-distress related variables (Panels B-D). The VS group show distinctive patterns compared with the distress-matched control sample, replicating the main comparison for variables including benefit receipt, cash withdrawals, account access and security, and bank interactions.
The VS-Control difference observed in disability benefit receipt (difference, 14.3pp, 95% CI 13 to 15.7) is almost double of that observed in the primary matched sample (difference 7.9pp, 95% CI, 6.6 to 9.3). This amplified difference highlights health-related vulnerabilities as a potentially important pathway into financial distress among victim-survivors, beyond that observed in financially distressed women without victim-survivor status. In the UK, it is estimated that disabled adults - defined as those living with a long-standing illness, condition, or impairment - are 2.6 times more likely to experience domestic abuse compared to non-disabled adults[1].
No evidence for VS-Control difference in optician spend was found (difference -0.5pp, 95% CI, -2 to 1), and estimated differences were smaller for dental (difference -3.4pp, 95% CI, -4.9 to -1.9 vs difference -8.8pp, 95% CI, -10.2 to -7.4 in the primary matched sample), and furniture spend (difference -4.5, 95% CI, -6 to -2.9 vs difference -8pp, 95% CI, -9.5 to -6.5 in the primary matched sample). Attenuated differences in self-care spend suggest that the experience of victim-survivors of financial abuse in these dimensions are not distinctive from women in financial distress without a victim-survivor status.
Interestingly, in contrast to results from the primary matched sample, no strong evidence of VS-Control differences in the distress-matched sample were found for petrol (difference 0.8pp, 95% CI, -0.5 to 2 vs difference 5pp, 95% CI, 3.8 to 6.3 in the primary matched sample) and taxi spend (difference 2.5pp, 95% CI, 0.9 to 4.1 vs difference 12.3pp, 95% CI, 10.8 to13.9 in the primary matched sample). This suggests that previously observed differences in these spending categories are not distinct from those seen in women who experience financial distress but not financial abuse. Such financial distress-related travel pressures may include irregular income, reduced flexibility, and time constraints[2].
Finally, the distress-matched results reveal distinctive bank interaction patterns for victim-survivors. The direction of the VS-Control difference was reversed for internet banking logins (difference -3.4, 95% CI, -5.3 to -1.5 vs difference 10.1, 95% CI, 8.3 to 11.9 in the primary matched sample). This suggests that, although increased internet banking logins are associated with financial distress, victim-survivors tend to rely more on human banking channels. This may reflect barriers to accessing internet banking, or the greater complexity and sensitivity of their needs, which are less easily addressed through digital channels.
Results from the relationship-end matched sample (Supplementary Figure 3, Supplementary Table 6) were consistent with those observed in the primary matched sample, although estimated VS-Control differences were generally smaller and several estimates were centered near zero, reflecting reduced statistical power given the smaller sample size.
Specifically, in the relationship-end matched sample, no evidence for VS-Control difference was found for payroll (difference -1.1pp, 95% CI, -2.6 to 0.5 vs difference -1.8pp, 95% CI, -2.6 to -1.1 in the primary matched sample), optician (difference -2.3pp, 95% CI, -5.3 to 0.7 vs difference -2.9pp, 95% CI, -4.3 to -1.5 in the primary matched sample), and sport (difference 1.3pp, 95% CI, -2 to 4.7 vs difference -5.3pp, 95% CI, -6.9 to -3.8 in the primary matched sample) spend.
Observed VS-Control differences were larger for cash withdrawal (difference 7pp, 95% CI, 5.4 to 8.5 vs difference 4pp, 95% CI, 3.3 to 4.7 in the primary matched sample), and petrol spend (difference 10.1pp, 95% CI, 7.4 to 12.7 vs difference 5pp, 95% CI, 3.8 to 6.3 in the primary matched sample). The larger differences in cash withdrawals and petrol spending

around relationship end compared to disclosure date may reflect practical demands associated with separation, including relocation or increased travel.”

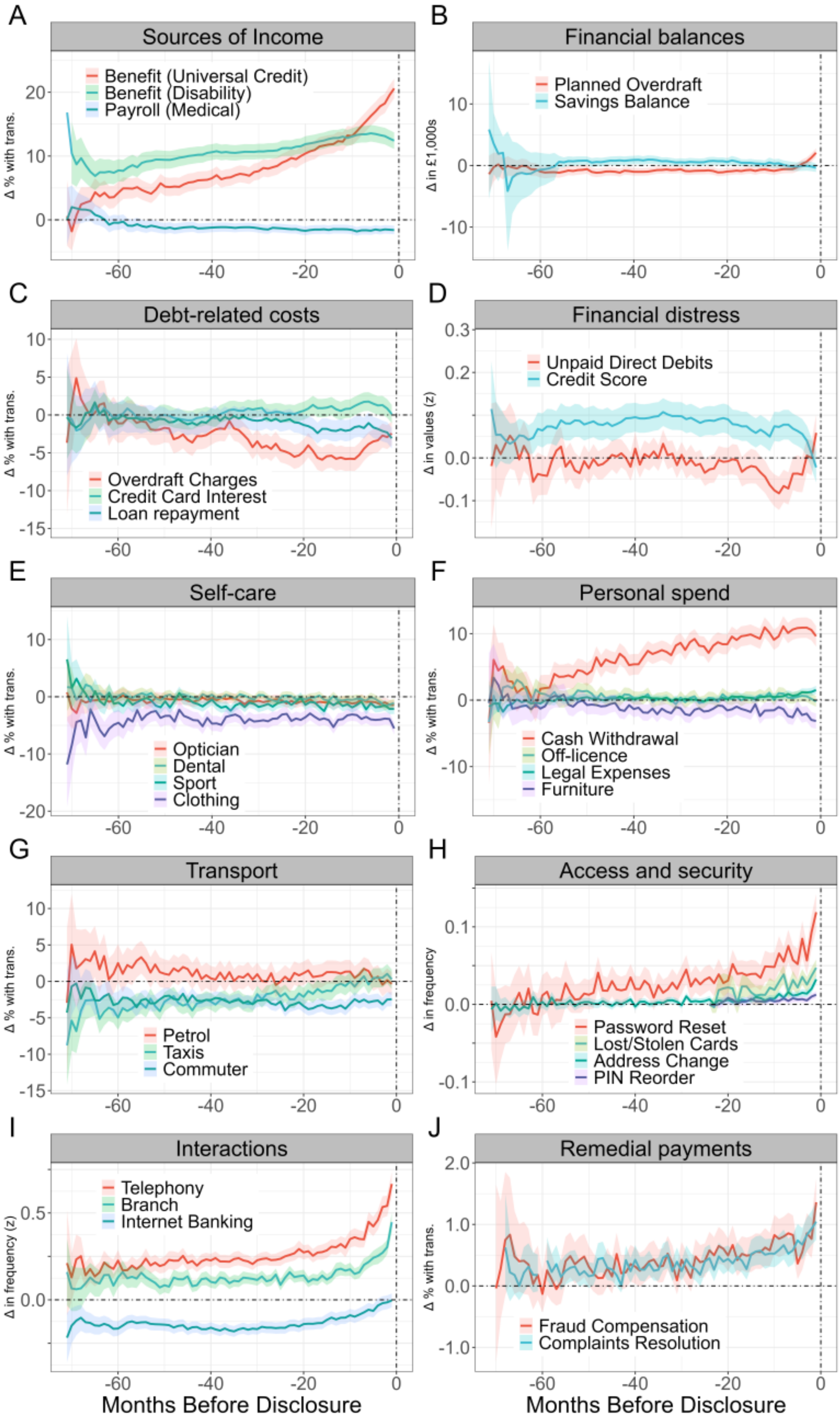

Supplementary Figure 2. Monthly VS-Control Group differences in selected financial indicators (distress-matched sample). Line plots showing monthly VS-Control group differences (vertical

axis). The horizontal axis shows the number of months relative to disclosure date (month = 0). Shading denotes 95% CI. Sample sizes: VS (N =5,183), Control (N =13,430). Data on Lost/Stolen cards and PIN reorder are only available from January, 2023. **Panel A. Sources of Income**. VS-Control difference in monthly group proportion with transactions. Exact (transactional) variables used: Benefits/Disability Living Allowance/Other, Benefits/Universal Credit/Other, Payroll/Medical/NHS. **Panel B. Financial balances**. VS-Control difference in monthly group averages (measured in thousand pounds). Exact (non-transactional) variables used: Planned Overdraft, Savings Balance. **Panel C. Debt-related costs.** VS-Control difference in monthly group proportion with transactions. Exact variables used: Credit Card Interest (derived transactional), Financial Services/Loans/Other (transactional), Overdraft charges (derived transactional). **Panel D. Financial distress.** VS-Control difference in scaled monthly group averages (measured in monthly credit scores, and monthly counts of missed direct debits). Exact variables used: Credit score (non-transactional), Unpaid direct debit (derived transactional). **Panel E. Self-care.** VS-Control difference in monthly group proportion with transactions. Exact (transactional) variables used: Retail/Clothing/Women, Services/Health/Dental, Services/Health/Optician, Hobbies & Interests/Sport/Other. **Panel F. Personal spend.** VS-Control difference in monthly group proportion with transactions. Exact variables used: ATM Withdrawal (derived transactional), Retail/Furniture/Other (transactional), Services/Professional/Legal (transactional), Retail/Food & Drink/Alcohol (transactional). **Panel G. Transport.** VS-Control difference in monthly group proportion with transactions. Exact (transactional) variables used: Travel/Commuter/Other, Motor/Petrol/Other, Travel/Taxis/Other. **Panel H. Access and security.** VS-Control difference in monthly frequency. Exact (non-transactional) variables used: Address Change, Lost/Stolen card, Password Reset, PIN Reorder. **Panel I. Interactions.** VS-Control difference in monthly scaled frequency. Exact (non-transactional) variables used: Branch visits, Internet Banking logins, Telephone visits. **Panel J.** Remedial payments. VS-Control difference in monthly group proportion with transactions. Exact (transactional) variables used: Financial Services/Fraud/Write Off, Financial Services/Other/Complaints Resolution.

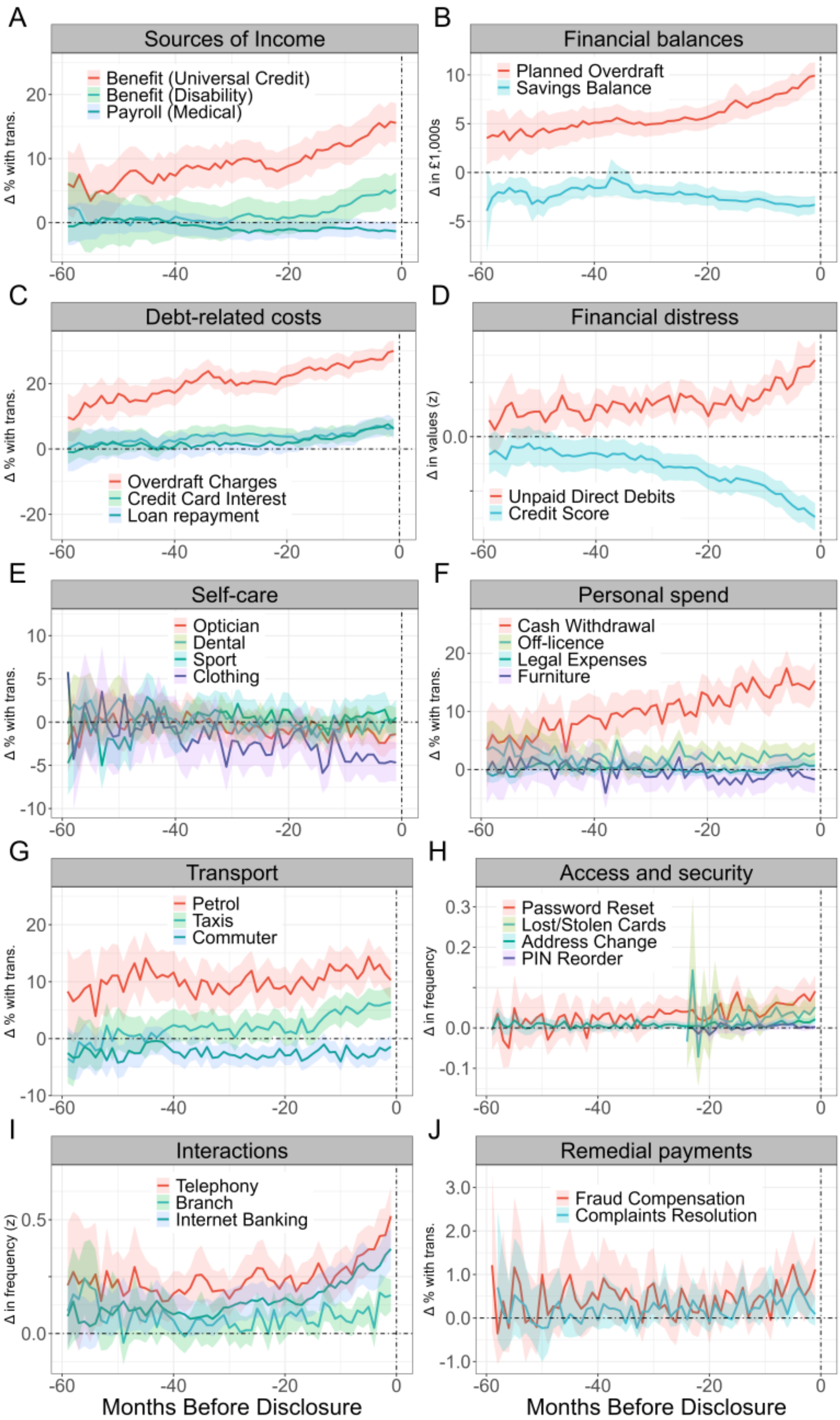

Supplementary Figure 3. Monthly VS-Control Group differences in selected financial indicators (relationship-end matched sample). Line plots showing monthly VS-Control group differences (vertical axis). The horizontal axis shows the number of months relative to relationship end date (month = 0). Shading denotes 95% CI. Sample sizes: VS (N =1,149), Control (N =3,236). Data on Lost/Stolen cards and PIN reorder are only available from January, 2023. **Panel A. Sources of**

Income. VS-Control difference in monthly group proportion with transactions. Exact (transactional) variables used: Benefits/Disability Living Allowance/Other, Benefits/Universal Credit/Other, Payroll/Medical/NHS. **Panel B. Financial balances**. VS-Control difference in monthly group averages (measured in thousand pounds). Exact (non-transactional) variables used: Planned Overdraft, Savings Balance. **Panel C. Debt-related costs.** VS-Control difference in monthly group proportion with transactions. Exact variables used: Credit Card Interest (derived transactional), Financial Services/Loans/Other (transactional), Overdraft charges (derived transactional). **Panel D. Financial distress.** VS-Control difference in scaled monthly group averages (measured in monthly credit scores, and monthly counts of missed direct debits). Exact variables used: Credit score (non-transactional), Unpaid direct debit (derived transactional). **Panel E. Self-care.** VS-Control difference in monthly group proportion with transactions. Exact (transactional) variables used: Retail/Clothing/Women, Services/Health/Dental, Services/Health/Optician, Hobbies & Interests/Sport/Other. **Panel F. Personal spend.** VS-Control difference in monthly group proportion with transactions. Exact variables used: ATM Withdrawal (derived transactional), Retail/Furniture/Other (transactional), Services/Professional/Legal (transactional), Retail/Food & Drink/Alcohol (transactional). **Panel G. Transport.** VS-Control difference in monthly group proportion with transactions. Exact (transactional) variables used: Travel/Commuter/Other, Motor/Petrol/Other, Travel/Taxis/Other. **Panel H. Access and security.** VS-Control difference in monthly frequency. Exact (non-transactional) variables used: Address Change, Lost/Stolen card, Password Reset, PIN Reorder. **Panel I. Interactions.** VS-Control difference in monthly scaled frequency. Exact (non-transactional) variables used: Branch visits, Internet Banking logins, Telephone visits. **Panel J.** Remedial payments. VS-Control difference in monthly group proportion with transactions. Exact (transactional) variables used: Financial Services/Fraud/Write Off, Financial Services/Other/Complaints Resolution.

Details on the construction of these samples can be found in the Methods (lines 614-654):
"Distress-matched and relationship-end matched samples. We applied the same filtering criteria as described above to create the distress-matched and relationship end date samples. Due to changes in the bank's data retention policy, at the time these analyses were conducted (January 2026), transaction histories used in the below analyses were restricted to 7 years. The analysis periods range between January 2019 and December 2024. Matching in the following analyses was performed using nearest-neighbor matching with a 3:1 treatment-to-control ratio, a caliper of 0.01, and without replacement.
We created the distress-matched VS (N = 5,387) and control candidate (N = 76,083) samples by applying the same filtering criteria as described above. To create a matched sample, we used variables capturing financial distress and matched the two samples at the end of the analysis period (12 months prior to disclosure date), to create a control sample that experienced similar levels of financial distress as the VS sample in the period preceding disclosure. We used the following matching variables: year and month of the individual-specific analysis period, age, median monthly credit turnover, median monthly number of transactions, deprivation rank of the geography where the individual lived, average credit score, credit score 12 months prior to disclosure, average monthly number of rejected direct debits and overdraft charges, average loan, savings, and planned overdraft balances, binary flags for the following: savings, loan accounts, usage of planned overdraft, unpaid direct debit and overdraft charges.
The propensity matching process resulted in a final sample of 5,183 VS and 13,430 Control individuals. Supplementary Table 8 shows the output of the propensity score matching.
To create the relationship-end date samples, we extracted relationship-end date information from the VS candidate sample. Applying the same criteria as described above, we retrieved notes associated with 5,303 victim-survivors. During appointments, with the customer's consent, bank staff records key details of each case in the form of notes. The richness of the information recorded vary by note, but some includes temporal markers, most commonly the relationship end date. We identified 3,559 notes that contained words

indicative of a relationship end date. These records either included month names (e.g., "jan") or any the following search words: fled, split, ended, ago, left. We then manually reviewed these notes and extracted relationship end dates with month-level precision. For example, "relationship ended 3 years ago" was not accurate enough, but "fled 2 weeks ago" would be included with a relationship end date two weeks prior to disclosure. We retrieved the latest known contact date with the abuser at the time of the disclosure, and only if it was within our analysis period (after January 2019). Therefore, relationship end could be missing if it was not mentioned in the note, was too long ago, was not specific enough or if the victim-survivor was still in the relationship at the time of disclosure. The manual review resulted in a sample with 1,189 VS sample candidates. On average, relationships ended 7.7 months (s.d. 9.3) prior to disclosure date.
The matching variables were the same as those used in the primary matched sample, the only difference being that the individual-specific analysis period was now defined by the relationship-end date as opposed to disclosure date. The propensity matching process resulted in a final sample of 1,149 VS and 3,236 Control individuals. Supplementary Table 9 shows the output of the propensity score matching"

We believe these sensitivity analyses provide valuable additional insights into the experience of victim-survivors and we are grateful to Reviewer 4 for suggesting these supplementary analyses.

-Addresses the self selection bias (R3-4) and carries out robustness checks and sensitivity analyses.

We have conducted a number of additional sensitivity and robustness checks in response to Reviewer comments.

1) **Contextualizing differences**: In response to comments from Reviewer 2 on the contextualization of economic impact and policy implications, we have created two additional benchmark samples (Bank population benchmark sample consisting of 19,059 randomly selected bank customers and Female Bank population benchmark sample comprising of 18,906 randomly selected female customers) to provide context to the findings reported. We report the 373 outcomes analysed previously for these samples in the year prior to disclosure in Supplementary Table 4. These estimates are directly comparable to the VS and Control means reported in the same period in Supplementary Table 3. More details about sample construction can be found in the Methods (lines 655-669).

To help the reader put the differences in context, when reporting VS-Control differences seen in the primary matched sample, we also report the population estimate of the mean of the sample of all bank customers, together with the 95% confidence interval around that estimate e.g. at line 224 "*difference -1.8pp, 95% CI, -2.6 to -1.1****; Bank pop. benchmark: 3.9%, 95% CI, 3.6 to 4.2***".

In the discussion, we call out examples associated with financial harm where we observe the largest differences (lines 464-479):

*"**The findings presented in this paper highlight considerations for measuring the socioeconomic costs of financial abuse. In the year preceding disclosure, compared to the broader bank population (Supplementary Tables 2 and 4), victim-survivors were more likely to experience adverse financial outcomes: the percentage with at least one missed***

direct debit was 279% higher (63% vs 17%), while those relying on Universal Credit welfare benefit was 421% higher (51% vs 10%). Similarly, compared to the bank population's averages, victim-survivors' credit scores were 20% lower (767 vs 960), and their savings balances were 78% lower (£3,088 vs £14,215).

Victim-survivors' financial experiences vary in severity, with bank disclosures likely reflecting more adverse cases. However, given the magnitude of the harms identified - including damaged credit scores - and the estimated prevalence of financial abuse, these costs are likely to be economically significant. Previous estimates place the annual cost of domestic abuse in the UK at £89 billion (2025 prices), equivalent to ~7% of expected public expenditure in the UK in 2024-25. Owing to data limitations, this estimate excludes costs associated with financial abuse. By quantifying these financial harms, our results could inform extensions of national cost models to capture the socioeconomic burden of domestic abuse more comprehensively."

2) **LBG representativeness:** In response to a comment from Reviewer 3 with regards to the representativeness of the bank sample, we have conducted an additional analysis to compare the characteristics of the bank sample compared to UK-level survey data. In Supplementary Table 1, we provide comparisons of the characteristics of the Lloyds Banking Group (LBG) population with the UK population (using UK population data[3] provided by the Office for National Statistics[4]; ONS). These tables compare the two populations by age, gender, region and income. They demonstrate that the LBG population is representative of the UK population, closely matching population distributions for each variable. This is as expected, given that Lloyds bank is one of the UK's largest retail bank with 28m customers, serving approximately 51% of the UK adult population. We have added a sentence at lines 151-152 of the revised version of the paper "*The retail bank serves approximately 51% of the UK adult population, and is representative of the population by age, gender, region and income (see Supplementary Table 1)"*. More details about the samples used for the comparison can be found in the Methods at lines 738-747.

3) **Impact of the filtering criteria:** In response to a comment from Reviewer 3 regarding the potential bias arising from permanence in the sample, we have compared the characteristics of the victim-survivor group by inclusion status.

Specifically, to examine the impact of the filtering criteria applied, we compared the characteristics of the VS candidate sample (N = 5,444) with those who were filtered out (N = 4,284) when applying criteria (1)-(4) at lines 566-570 in the Methods. This comparison focuses exclusively on characteristics unaffected by account activity: age, credit score, and deprivation rank. Other metrics, such as income, are less accurately measured for inactive bank customers who did not meet out transaction criteria, and therefore the comparison would not be informative. These results are shown in Supplementary Table 11.

First, we compared the mean age at the start of the individual-specific period. We found that compared to VS individuals included in the final sample, excluded VS candidates were on average 5 years younger at the start of the individual-specific period (difference 4.9 years, 95% CI, 4.5 to 5.2), reflecting the minimum age threshold (18) imposed at the start of the individual-specific time period.

Second, we compared the mean difference in credit scores and deprivation rank between the first and last year of the individual-specific analysis period. The decline in average credit scores between the first and last year of the individual-specific period was slightly larger amongst included VS individuals compared to excluded VS candidates (difference -13.3

points, 95% CI, -24.3 to -2.5). In contrast, we found no evidence for differences in deprivation rank change between the start and the end of the analysis period (difference 296 ranks, 95% CI, -37 to 636) by inclusion status.

Overall, while the included VS sample is slightly older than excluded candidates, we find no evidence suggesting that permanence in the sample is systematically associated with differential changes in credit scores or deprivation rank. This analysis is included in the Methods at lines 765-569.

4) **Increased demand for borrowing:** In response to a comment from Reviewer 3 regarding the seemingly conflicting finding that victim-survivors simultaneously increase their borrowing and experience declining credit scores, we were able to analyse credit application data to provide further context.

We compare application rates and access rates for the primary matched victim-survivor and control groups, with results shown in Supplementary Figure 1. Results show that victim-survivors have higher application rates for credit cards and loans, but do not have higher success rates. For other financial products there are no differences in application and success rates between the groups.

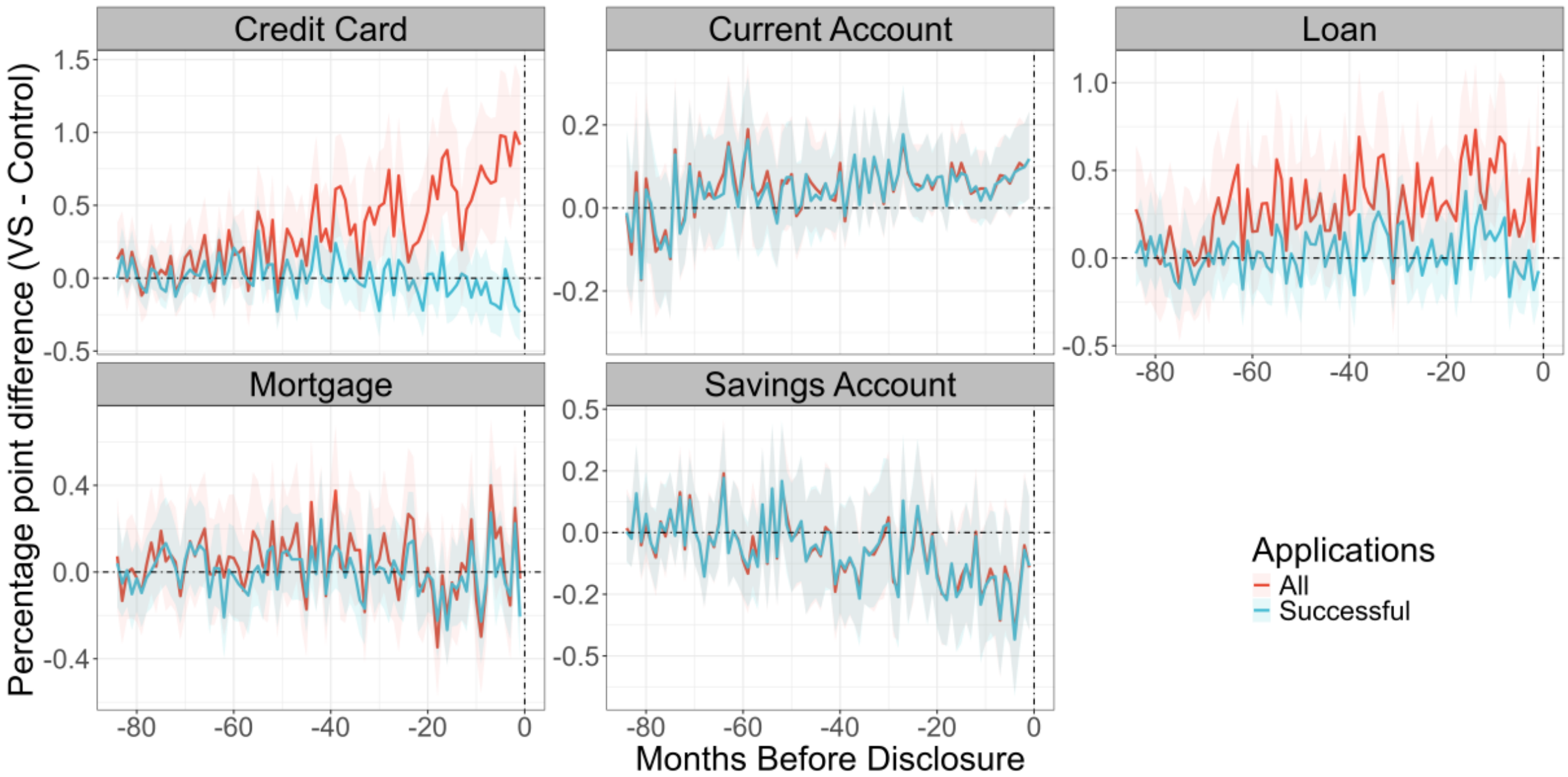

Supplementary Figure 1 Victim-survivor and Control financial product applications (primary matched sample). VS-Control percentage point differences in the number of total, and successful financial product applications (credit card, current account, loan, mortgage, savings account) in the 7 year period prior to disclosure month. Results show that victim-survivors have higher application rates for credit cards and loans, but do not have higher success rates. There are no differences in application and success rates between the groups for other financial products. For methodological details, see "Financial product applications and application outcomes".

We conclude that this provides evidence against a hypothesis a supply-side explanation for our results whereby victim-survivors are more successful in accessing credit. We have added at lines 244-246: *"**Additional analysis shows that compared to Control, the VS group increases the number of applications submitted for credit (credit cards and personal loans) over time, with no corresponding difference in success rates, indicating that higher debt***

levels are at least partly a result of higher demand for credit (see Supplementary Figure 1).”

5) **Measuring the degree of self-selection by financial distress**: Reviewers 4 and 5 both raised points regarding the possibility of the victim-survivor sample exclusively comprising of individuals in severe financial distress. To better understand how victim-survivor’s financial distress compare to the financial distress experienced a comparable bank population, we compared the average credit score distributions in the VS sample to that of the Female bank population benchmark sample (a sample of 18,906 randomly selected female bank customers).

Details on the construction of this sample can be found in the Methods section (lines 655-669), while Supplementary Figure 7 shows the comparison.

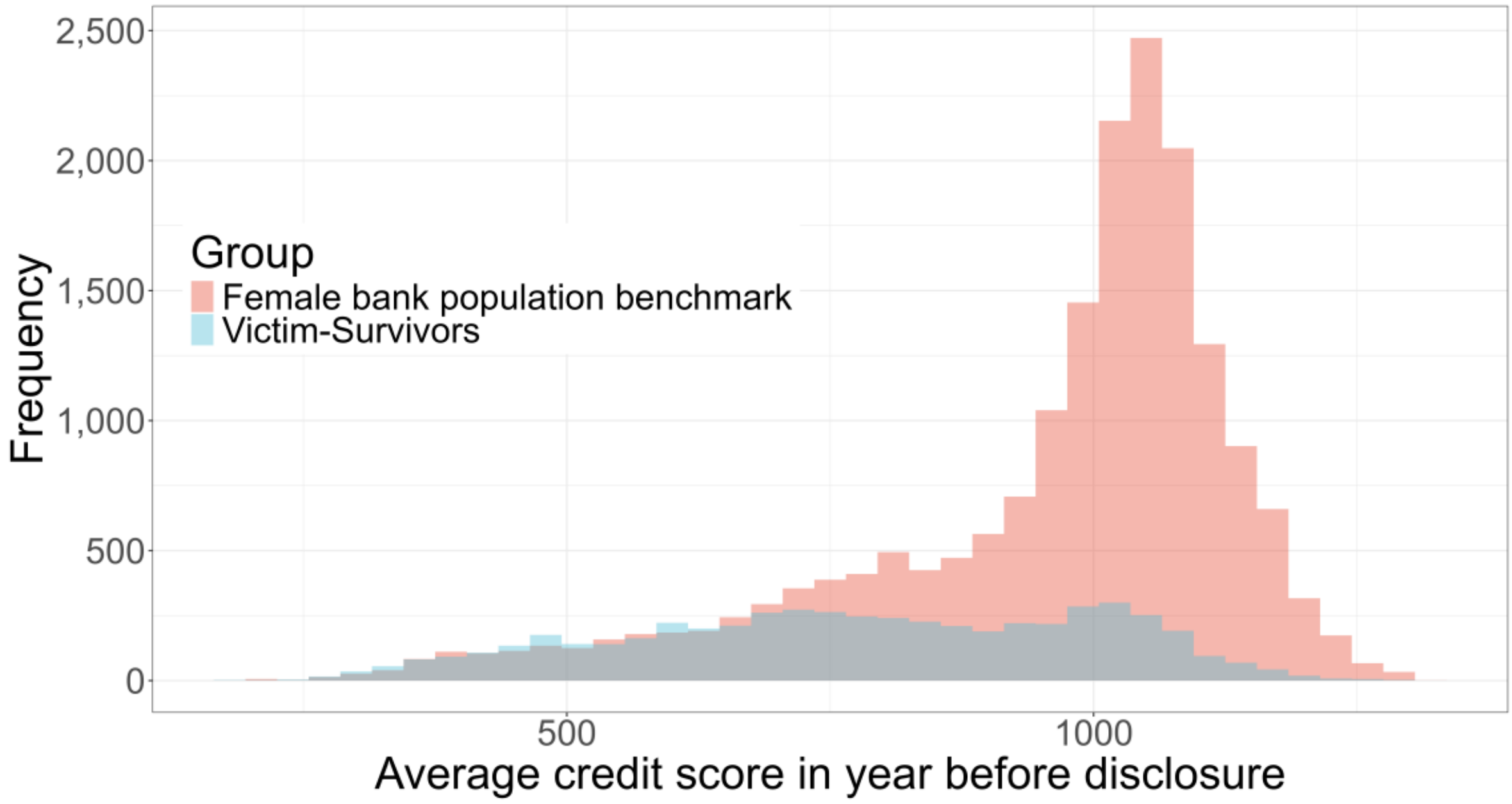

Supplementary Figure 7. Victim-survivor and Female bank population credit score distributions in the year prior to disclosure. The figure shows that while on average, credit scores are significantly lower in the VS group (as expected), we see a wide distribution spanning the entire interval of Control credit score values, suggesting that that the VS sample does not only include individuals in severe financial distress. For methodological details, see "Measuring the degree of self-selection by financial distress".

This reveals two patterns. First, as expected, the average credit score among victim-survivors is lower than that among the Female bank population benchmark group. Second, the plot illustrates that the victim-survivor group does not only contain individuals with severely low credit scores, but instead contains individuals with credit scores across the distribution of the Female bank population benchmark group. Overall, 35% of individuals in the victim-survivor group have credit scores that sit above the 25th percentile of credit score (887) for the Female bank population benchmark group. We agree with the reviewer comment that one might be concerned in particular if the victim-survivor group were heaped at very low credit scores and/or the plot illustrated a clear bi-modality between the two groups. We are reassured by the overlapping distributions that, while the credit score for victim-survivors is lower than that for the Female bank population benchmark group, our victim-survivor sample is not only limited to individuals experiencing severe financial situations.

-Addresses the remaining comments from the reviewers.

Below we address the remaining comments from the reviewers that were not included in the revisions discussed above. Here we only provide a summary; more detailed, point-by-point responses to these items are provided in the reviewer-specific sections.

1) Reviewer 1 asked for clarification regarding 'commuting-related expenses'. At lines 316 in the revised version of the manuscript we have added in parenthesis: *"lower activity in commuting-related spend (**consisting of spend on urban public transport companies e.g., Transport for London**; difference -5.5pp, 95% CI, -6.9 to -4.1;"*.

2) Reviewer 1 raised an issue with the code link not opening. We hope the revised code link will work for the reviewer, alternatively we are happy to provide the code via an alternative method.

3) Reviewer 2 suggested improvements to the code provided, specifically 1) ensuring that the specific package versions are used automatically in the code script and 2) using a path relative to the execution directory. To support reproducibility of the code we have revised the code package and made the following changes: 1) the script now uses a path relative to the execution directory and 2) specific versions of packages are automatically installed and loaded (in R we use the renv package used for creating reproducible environments and in Python packages are installed via a requirements.txt).

4) Reviewer 3 suggested controlling for the simultaneous occurrence of different types of abuse and restricting the set of outcomes examined. Reviewer 2 also raised a point regarding the relevance of non-financial outcomes.

   Available research evidence suggests that different aspects of coercive control, including emotional, physical, psychological, and financial abuse tend to co-occur substantially[5]. Furthermore, as financial stability is a foundational determinant of overall wellbeing, financial hardship often co-occurs with broader instability and disadvantage across multiple domains of a victim-survivor's life.

   In line with these considerations and the clarified goal of the paper, that is, to describe the experience of victim-survivors using banking data, we feel that the broad set of outcomes considered in the paper is relevant for illustrating the multi-domain experiences associated with financial abuse. Beyond financial outcomes such as balances and debts, transaction data reveals distinct patterns across different areas of life (e.g., employment, welfare, well-being, travel).

   We have revised the manuscript to position financial abuse within the wider context of coercive control, and to highlight the co-occurrence of different types of abuse, as well as the multidimensional nature of victim-survivors' experience:

- *"**While research on domestic abuse has mostly focused on physical violence, many scholars stress that violence must be understood within the wider context of***

coercive control, which can also encompass psychological, sexual, economic and financial abuse*."* Lines 101-103

- *"**Financial abuse is often intertwined with other aspects of coercive control (e.g., emotional, sexual or physical abuse)**[5]**, reflecting a broader constellation of harms that may include stress, injury, reduced work capacity, social isolation and poorer financial wellbeing.**"* Lines 109-112
- *"**For the victim, loss of control over their financial life often co-occurs with difficulties across non-financial domains of life, including mental and physical health, the ability to pay for personal goods, and participation in leisure and well-being activities."*** Lines 117-120

5) Reviewer 3 mentioned that some of the explanations in the original version of the manuscript seem somewhat stretched. In line with the removal of language implying causality, we have revised the narrative provided for the findings we describe in the Results section to make it more theoretically conservative and aligned with the descriptive scope of the study:

- *"~~Decreased spend~~ **Decreased spending** on self beyond basic necessities ~~may reflect the impact of~~ **aligns with patterns of** financial control ~~– either through restriction tactics or exploitation leaving victim-survivors with less money via increased borrowing~~ **documented in prior studies, including restricted access to funds and financial exploitation, both of which have been linked to reduced financial autonomy among victim-survivors"*** lines 273-276
- *"Higher spending in off-licence stores is consistent with prior evidence showing that victim-survivors may use alcohol as a coping strategy~~, which may even be encouraged by the abuser to increase control~~"*-lines 282-284
- *"The pattern of increased cash usage ~~might reflect~~ **is consistent with a range of experiences reported in the literature, including** financial exploitation through unauthorized or forced cash withdrawals aiming to deplete the victim-survivor's resources"* lines 307-312
- *"Lower commuting-related spending in the VS group ~~is in line with earlier results showing lower likelihood of employment and could result from sabotaging employment, while increased petrol spend may signal the victim-survivor's attempts to maintain safety and access support, which may involve longer journeys. Access to taxis can provide a quick and discreet emergency escape option particularly when shared vehicles are being tracked by the abuser, and an analysis of escape journeys of women found that taxis were the second most common option after transport provided by friends and family.~~ **aligns with earlier findings of lower employment prevalence among victim-survivors. Higher spending on flexible transport options, including cars and taxis, has been reported previously and may reflect the complex, constrained circumstances in which victim-survivors meet everyday needs, including limited alternatives, time pressures and safety considerations."*** lines 319-323

- *"All of these activities ~~may be linked to the abuser taking control of bank accounts or victim-survivors seeking to take back control~~* ***reflect changes in account access and management consistent with those reported in situations involving financial abuse, including both shifts in control by an abuser and increased engagement by victim-survivors*** *post-separation, when it may be safe to do so. Frequent ~~address changes are likely to reflect separation and the victim-survivors' efforts to prevent the abuser from discovering their new location~~* ***changes of address align with the residential instability often associated with separation"*** lines 333-337
- *"**Observed** changes in cash usage, transport spend**ing**, and account management activity ~~may reflect victim-survivors' efforts to escape their abuser~~* ***are consistent with patterns reported around periods of safety-seeking or separation among victim-survivors.****"* Lines 500-502

Furthermore, the two supplementary analyses allow for a more granular investigation of the robustness of our findings. While the results from these analyses largely replicate the findings reported in the primary matched sample, the observed points of divergence provide additional valuable insights into the experiences of victim-survivors (lines 367-416):

- ***"Results from the distress-matched sample (Supplementary Figure 2, Supplementary Table 5) show that a close match is achieved for financial-distress related variables (Panels B-D). The VS group show distinctive patterns compared with the distress-matched control sample, replicating the main comparison for variables including benefit receipt, cash withdrawals, account access and security, and bank interactions.***
- ***The VS-Control difference observed in disability benefit receipt (difference, 14.3pp, 95% CI 13 to 15.7) is almost double of that observed in the primary matched sample (difference 7.9pp, 95% CI, 6.6 to 9.3). This amplified difference highlights health-related vulnerabilities as a potentially important pathway into financial distress among victim-survivors, beyond that observed in financially distressed women without victim-survivor status. In the UK, it is estimated that disabled adults - defined as those living with a long-standing illness, condition, or impairment - are 2.6 times more likely to experience domestic abuse compared to non-disabled adults.***
- ***No evidence for VS-Control difference in optician spend was found (difference -0.5pp, 95% CI, -2 to 1), and estimated differences were smaller for dental (difference -3.4pp, 95% CI, -4.9 to -1.9 vs difference -8.8pp, 95% CI, -10.2 to -7.4 in the primary matched sample), and furniture spend (difference -4.5, 95% CI, -6 to -2.9 vs difference -8pp, 95% CI, -9.5 to -6.5 in the primary matched sample). Attenuated differences in self-care spend suggest that the experience of victim-survivors of financial abuse in these dimensions are not distinctive from women in financial distress without a victim-survivor status.***
- ***Interestingly, in contrast to results from the primary matched sample, no strong evidence of VS-Control differences in the distress-matched sample were found for***

petrol (difference 0.8pp, 95% CI, -0.5 to 2 vs difference 5pp, 95% CI, 3.8 to 6.3 in the primary matched sample) and taxi spend (difference 2.5pp, 95% CI, 0.9 to 4.1 vs difference 12.3pp, 95% CI, 10.8 to13.9 in the primary matched sample). This suggests that previously observed differences in these spending categories are not distinct from those seen in women who experience financial distress but not financial abuse. Such financial distress-related travel pressures may include irregular income, reduced flexibility, and time constraints.

- ***Finally, the distress-matched results reveal distinctive bank interaction patterns for victim-survivors. The direction of the VS-Control difference was reversed for internet banking logins (difference -3.4, 95% CI, -5.3 to -1.5 vs difference 10.1, 95% CI, 8.3 to 11.9 in the primary matched sample). This suggests that, although increased internet banking logins are associated with financial distress, victim-survivors tend to rely more on human banking channels. This may reflect barriers to accessing internet banking, or the greater complexity and sensitivity of their needs, which are less easily addressed through digital channels.***
- ***Results from the relationship-end matched sample (Supplementary Figure 3, Supplementary Table 6) were consistent with those observed in the primary matched sample, although estimated VS-Control differences were generally smaller and several estimates were centered near zero, reflecting reduced statistical power given the smaller sample size.***
- ***Specifically, in the relationship-end matched sample, no evidence for VS-Control difference was found for payroll (difference -1.1pp, 95% CI, -2.6 to 0.5 vs difference -1.8pp, 95% CI, -2.6 to -1.1 in the primary matched sample), optician (difference -2.3pp, 95% CI, -5.3 to 0.7 vs difference -2.9pp, 95% CI, -4.3 to -1.5 in the primary matched sample), and sport (difference 1.3pp, 95% CI, -2 to 4.7 vs difference -5.3pp, 95% CI, -6.9 to -3.8 in the primary matched sample) spend.***
- ***Observed VS-Control differences were larger for cash withdrawal (difference 7pp, 95% CI, 5.4 to 8.5 vs difference 4pp, 95% CI, 3.3 to 4.7 in the primary matched sample), and petrol spend (difference 10.1pp, 95% CI, 7.4 to 12.7 vs difference 5pp, 95% CI, 3.8 to 6.3 in the primary matched sample). The larger differences in cash withdrawals and petrol spending around relationship end compared to disclosure date may reflect practical demands associated with separation, including relocation or increased travel."***

6) Reviewers 3 and 4 suggested discussing the generalizability of the findings reported outside the UK. We agree that this is an important point, and therefore we addressed it in the Limitations section:

- *"Our study has several limitations.* ***First, the victim-survivor group, comprising of women in the UK with bank accounts who disclosed their victim-survivor status to the bank, and have met our sample selection criteria is not necessarily representative of the average victim-survivor, especially if disclosure is associated with experiencing significant financial distress. The findings reported here are most directly applicable to contexts with high levels of financial inclusion and***

***individualized banking systems, such as the UK and may not generalize to international contexts, where financial abuse is shaped by different social and cultural norms, nor to settings where gendered and unpaid labor constitute central forms of economic control. Further work is needed to understand the extent to which the results reported in this specific group are generalizable to other populations.*"** Lines 522-531

7) Reviewer 4 raised an important point regarding the use of data from a single financial institution. To mitigate this, we applied a filtering criterion based on transactional activity over a seven-year period; this ensured that individuals in the final sample likely used LBG as their primary bank.

   Furthermore, the credit scores employed in our analysis reflect financial activity across all institutions, providing a more holistic view of the individual's position beyond their activity with LBG. At lines 255–257, we explicitly note that credit scores capture an individual's broader financial health: "***Credit score measures individual creditworthiness using data from all bank accounts and financial products, including those beyond the bank used in our analysis, and summarizes the increasing financial strain experienced by the VS group relative to Control."***

   In addition, we have addressed this constraint within the Limitations section:

   "First, the victim-survivor group, comprising of women in the UK with bank accounts who disclosed their victim-survivor status to the bank, and have met our sample selection criteria is not necessarily representative of the average victim-survivor, especially if disclosure is associated with experiencing significant financial distress. (…) Due to data availability restrictions, our analysis only looks at a maximum 7-year window ***and draws on data from one bank.*** *Since domestic abuse can span decades****, impacting all bank accounts held by the individual,*** *this timeframe* ***and data view*** *may ~~not fully capture the long-term financial markers of~~* ***reflect all of an individual's financial patterns associated with financial abuse.*** *"*

8) Reviewer 4 highlighted the importance of qualitative research (life history interviews) for understanding the motivations underlying the differentiated financial behaviours seen in the data.

   While the present study is intended as a quantitative characterization of financial behaviors rather than a direct investigation of motivations, we agree with the reviewer that qualitative work is invaluable to better understand the drivers underlying the observed patterns. We now explicitly acknowledge the need for complementary qualitative research to explore these mechanisms in greater depth, and we view such work as an important direction for future research beyond the scope of the current study (Discussion, lines 544–549):

   "Third, our methodological approach does not allow us to establish causality between financial abuse and observed outcomes. For example, there may be two-way relationship between financial abuse and financial distress, or factors jointly driving abuse and distress. In addition, administrative data does not allow us to

observe individual motivations or decision-making processes underlying these patterns; qualitative research, such as life-history interviews, would be valuable for exploring these mechanisms in greater depth."

9) Reviewer 4 suggested that the manuscript could engage more extensively with existing qualitative literature

We have now included the work of Sundari Anitha and S. Singh, in the context of generalizability to non-UK settings (lines 522-531) as suggested by the reviewer:

"Our study has several limitations. First, the victim-survivor group, comprising of women in the UK with bank accounts who disclosed their victim-survivor status to the bank, and have met our sample selection criteria is not necessarily representative of the average victim-survivor, especially if disclosure is associated with experiencing significant financial distress. The findings reported here are most directly applicable to contexts with high levels of financial inclusion and individualized banking systems, such as the UK and may not generalize to international contexts, where financial abuse is shaped by different social and cultural norms[57], nor to settings where gendered and unpaid labor constitute central forms of economic control[58]. Further work is needed to understand the extent to which the results reported in this specific group are generalizable to other populations."

57. Singh, S. Domestic Economic Abuse: The Violence of Money. (Routledge, London, 2021). doi:10.4324/9781003178606.
58. Anitha, S. Understanding Economic Abuse Through an Intersectional Lens: Financial Abuse, Control, and Exploitation of Women's Productive and Reproductive Labor. Violence Women 25, 1854–1877 (2019).

10) Reviewer 5 requested 1) adding a number of key references to the Introduction section 2) positioning financial abuse in relation to economic abuse and 3) elaborating on how financial abuse fits within the broader pattern of coercive control.

We have revised the Introduction as follows:

"While research on domestic abuse has mostly focused on physical violence[12], many scholars stress that violence must be understood within the wider context of coercive control[13,14], which can also encompass psychological, sexual, economic and financial abuse. The terms 'economic' and 'financial' abuse are often used interchangeably in the literature, but while economic abuse is broadly defined as control over economic resources, financial abuse specifically focuses on control over money and finances[15]." Lines 101-106

"For the victim, loss of control over their financial life often co-occurs with difficulties across non-financial domains of life, including mental and physical health, the ability to pay for personal goods, and participation in leisure and well-being activities[19,20]." Lines 117-120

"Previous studies based on interviews and small-scale surveys reveal that even years after being subjected to abuse, victim-survivors often remain burdened by

substantial debt, damaged credit scores[1], and the less visible loss of financial skills and confidence[2], all of which hinder regaining financial independence and stability[3]." Lines 121-124

1. Adams, A. E., Littwin, A. K. & Javorka, M. The Frequency, Nature, and Effects of Coerced Debt Among a National Sample of Women Seeking Help for Intimate Partner Violence. Violence Women ***26****, 1324–1342 (2020).*
12. Postmus, J. L., Hoge, G. L., Breckenridge, J., Sharp-Jeffs, N. & Chung, D. Economic Abuse as an Invisible Form of Domestic Violence: A Multicountry Review. Trauma Violence Abuse ***21****, 261–283 (2020).*
19. Johnson, L., Chen, Y., Stylianou, A. & Arnold, A. Examining the impact of economic abuse on survivors of intimate partner violence: a scoping review. BMC Public Health ***22****, 1014 (2022).*
20. Stylianou, A. M. Economic Abuse Within Intimate Partner Violence: A Review of the Literature. Violence Vict. ***33****, 3–22 (2018).*

11) Reviewer 5 requested that the lack of data on the time since the start of the abuse is explicitly acknowledged in the limitations

We have revised the limitations as follows (lines 537-543):

"*(..)Due to data availability restrictions, our analysis only looks at a maximum 7-year window* ***and draws on data from one bank.*** *Since domestic abuse can span decades****, impacting all bank accounts held by the individual,*** *this timeframe* ***and data view*** *may ~~not fully capture the long-term financial markers of~~* ***reflect all of an individual's financial patterns associated with financial abuse. In addition, disclosure records do not include information on the duration of abuse prior to disclosure, which limits our ability to relate observed financial behaviors to the duration or timing of abuse."***

12) Reviewer 5 suggested adding labels or units along the vertical axis of Figure 1 to aid comprehension; and providing a shared category order for Panels A and B on Figure 2 to facilitate comparison

We have revised Figure 1 to include vertical axis labels as requested. Regarding the category ordering in Figure 2, we have chosen to retain the current independent ordering for each panel. As we detailed in our response to the Reviewer, we believe this allows the reader to immediately infer the relative ranking of categories within each perspective (size and onset of differences) and that forcing a uniform order across both panels would obscure these rankings and make the primary findings less

intuitive to infer.

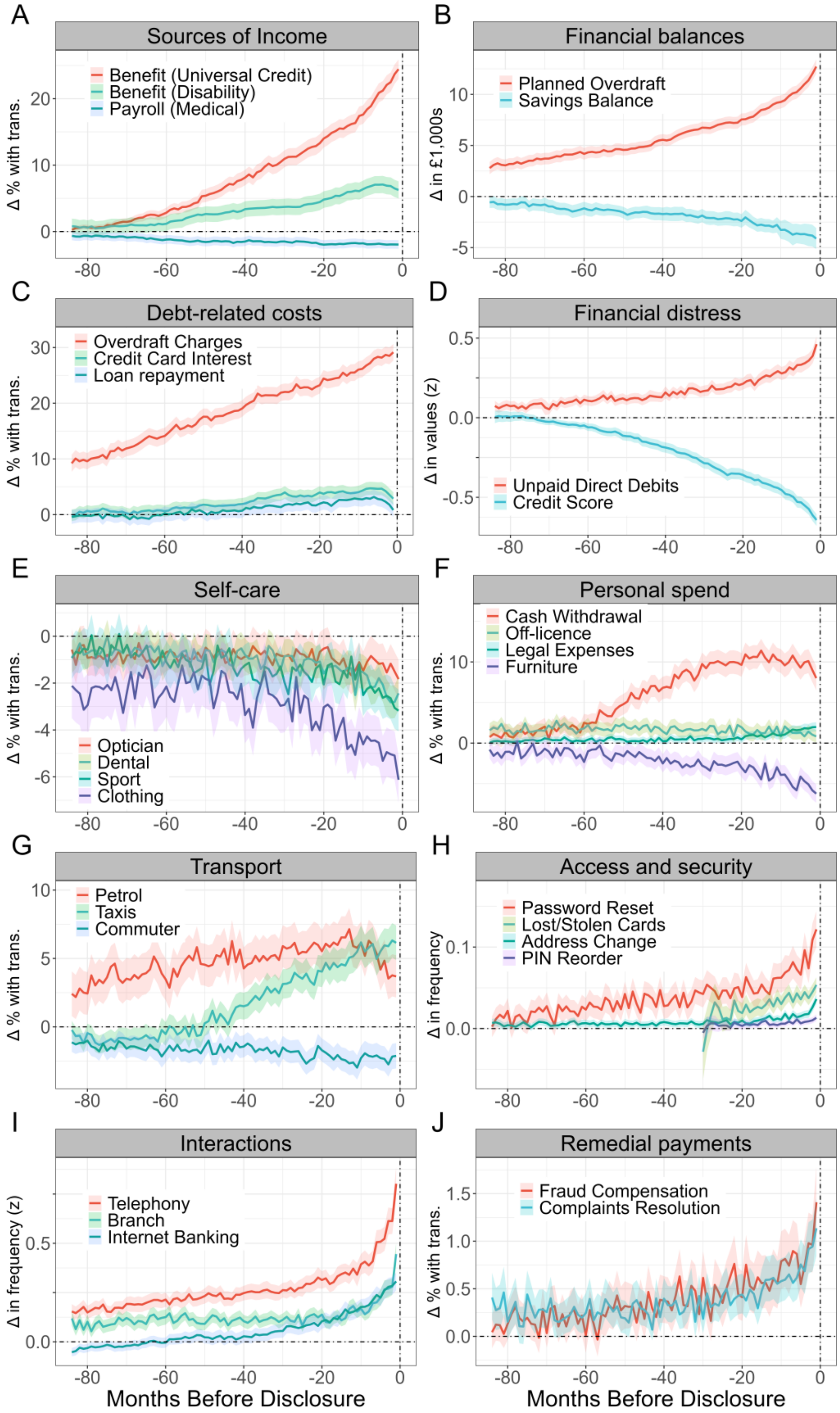

Figure 1. Monthly VS-Control Group differences in selected financial indicators (primary matched sample). Line plots showing monthly VS-Control group differences (vertical axis). The horizontal axis shows the number of months relative to disclosure date (month = 0). Shading denotes 95% CI. Sample sizes: VS (N =5,428), Control (N =15,602). Data on Lost/Stolen cards and PIN reorder are only available from June, 2022. **Panel A. Sources of Income**. VS-Control difference in monthly group proportion with transactions. Exact (transactional) variables used: Benefits/Disability Living Allowance/Other, Benefits/Universal Credit/Other, Payroll/Medical/NHS. **Panel B. Financial balances**. VS-Control difference in monthly group averages (measured in thousand pounds). Exact (non-transactional) variables used: Planned Overdraft, Savings Balance. **Panel C. Debt-related costs.**

VS-Control difference in monthly group proportion with transactions. Exact variables used: Credit Card Interest (derived transactional), Financial Services/Loans/Other (transactional), Overdraft charges (derived transactional). **Panel D. Financial distress.** VS-Control difference in scaled monthly group averages (measured in monthly credit scores, and monthly counts of missed direct debits). Exact variables used: Credit score (non-transactional), Unpaid direct debit (derived transactional). **Panel E. Self-care.** VS-Control difference in monthly group proportion with transactions. Exact (transactional) variables used: Retail/Clothing/Women, Services/Health/Dental, Services/Health/Optician, Hobbies & Interests/Sport/Other. **Panel F. Personal spend.** VS-Control difference in monthly group proportion with transactions. Exact variables used: ATM Withdrawal (derived transactional), Retail/Furniture/Other (transactional), Services/Professional/Legal (transactional), Retail/Food & Drink/Alcohol (transactional). **Panel G. Transport.** VS-Control difference in monthly group proportion with transactions. Exact (transactional) variables used: Travel/Commuter/Other, Motor/Petrol/Other, Travel/Taxis/Other. **Panel H. Access and security.** VS-Control difference in monthly frequency. Exact (non-transactional) variables used: Address Change, Lost/Stolen card, Password Reset, PIN Reorder. **Panel I. Interactions.** VS-Control difference in monthly scaled frequency. Exact (non-transactional) variables used: Branch visits, Internet Banking logins, Telephone visits. **Panel J.** Remedial payments. VS-Control difference in monthly group proportion with transactions. Exact (transactional) variables used: Financial Services/Fraud/Write Off, Financial Services/Other/Complaints Resolution.

Should further data and analysis allow you to address these criticisms, we would be happy to consider a revised manuscript (unless something similar has been accepted at Nature or appeared elsewhere in the meantime). We hope to receive your revised paper within four to six months. If you cannot complete the required revisions within this time frame, please let us know when you would anticipate being able to submit a revised manuscript.

Any revised manuscript should conform to our format instructions and publication policies (see here). We also strongly suggest that your revised manuscript has tracked changes, which is increasingly requested by referees to aid in their re-review.

Thank you for this consideration. We enclose the revised manuscript with tracked changes.

The journal guidance[6] states papers in social science do not normally exceed 8 pages on average, typically containing 5-6 modest display items and running to 4,300 words. Our article contains 2 large display items (Figures 1 and 2) together with one modest-to-large display item (Figure 3). We have therefore limited the text (summary paragraph and body test) to 4,300 words.

We would appreciate your careful attention to the following:

STATISTICS: When revising your manuscript, you should ensure that any statistical analysis used is sound and that it conforms to Nature's guidelines). A collection of articles explaining the basics of statistical analysis and advice on how to best present it can be found here.

REPRODUCIBILITY: All of the checklists provided with the current submission (Reporting summary, and Code and software checklist (if applicable)) should be updated to reflect the revisions made and submitted with the revised manuscript.

DATA AND CODE AVAILABILITY STATEMENTS: All original research manuscripts published in Nature Portfolio journals must include a Data availability statement. This statement must make the conditions of access to the "minimum dataset" that is necessary to interpret, verify and extend the research in the article, transparent to readers. This minimum

dataset may be provided through deposition in public community/discipline-specific repositories, custom proprietary repositories for certain types of datasets, or general repositories like Figshare, Zenodo and Dryad. Providing large datasets in Supplementary Information is strongly discouraged; the preferred approach is to make data available in repositories. More information on Nature Portfolio's reporting standards and preparing your Data availability statement can be found here.

For all studies using custom code or mathematical algorithms that are deemed central to the conclusions, a Code availability statement must be included, indicating whether and how the code or algorithm can be accessed, including any restrictions to access. The Code availability statement should be provided as a separate section after the Data availability statement but before the references. Code should be deposited in a DOI-minting repository such as Zenodo, Gigantum or Code Ocean and cited in the reference list. We encourage you to manage subsequent code versions and to use a license approved by the open source initiative. Additional details can be found here.

METHODS: After the main text figure legends there should be a section entitled "Methods", which provides the full, step-by-step instructions that would allow other researchers to replicate the results. The Methods section will not appear in print but will appear online in the full-text HTML and PDF versions. The Methods section should be written as concisely as possible but should contain all elements necessary to allow interpretation and reproduction of the results. If there are additional references in the Methods section, their numbering should continue from the last reference in the main text, and they should be listed following the Methods section. Specialized methods that require chemical structures, figures or tables, or methods requiring equations, cannot be accommodated in the Methods section of the main text file. If such information is part of the Methods, the entire Methods section must instead be included within a Supplementary Information text file.

ETHICS STATEMENT: For research involving human research participants, the Methods section must include an ethics statement. This statement should provide the name of the committee that approved the study; confirm that the research was performed in accordance with all relevant guidelines and regulations; and confirm that informed consent was obtained from all participants. If the study was granted an exemption from requiring ethics approval, details of the committee granting the exemption must be included.
For research involving human embryos or gametes, or human stem cells in contexts requiring ethical oversight, the Methods section must include an ethics statement. This statement should provide the name of the committee that approved the study and confirm that the research was performed in accordance with all relevant guidelines and regulations. We encourage authors to follow the principles laid out in the 2021 ISSCR Guidelines for Stem Cell Research and Clinical Translation. Where necessary, the ethics statement should also describe the conditions of donation of materials, such as human embryos or gametes, and confirm that informed consent was obtained from all donors of cells or tissues.

EXTENDED DATA: Extended Data do not appear in the print version of the paper but are included online within the full-text HTML and at the end of the online PDF. Extended Data are an integral part of the paper, and only data that directly contribute to the main message should be included. All Extended Data must be referred to in the main text, and their legends should be listed sequentially at the end of the main text, not in the Extended Data files. Extended Data should be assembled into a maximum of 10 A4 size, multi-panelled display items, submitted as individual files in .jpg, .tif or .eps format only. They should be of the same

quality as print figures, but there are important differences in their formatting. More specific instructions are provided here. If you need to describe complex processes, we encourage you to include a schematic of the main finding as part of the Extended Data to aid readers unfamiliar with the immediate discipline.

SUPPLEMENTARY INFORMATION: Supplementary Information (SI) is online-only, peer-reviewed material that is essential background to the study (e.g., large data sets, more complex methods, and calculations), but which is too large or impractical, or of interest only to a few specialists, to justify inclusion in the print version of the paper (see here for further details). While SI should not typically contain data figures (any figures additional to those appearing in the main text should be formatted as Extended Data), we require that the raw, uncropped data for gels be presented as an SI figure (see below). Tables may be included in SI, but only if they are unsuitable for formatting as Extended Data (e.g., tables containing large data sets or raw data tables that are best suited to Excel files). If a manuscript has SI, each discrete item of the SI (e.g., videos, tables) must be referred to at an appropriate point in the main manuscript.

SOURCE DATA (GRAPHS): To increase transparency, we strongly encourage you to provide, in spreadsheet form, the data underlying the graphical representations used in figures. In the case of all experiments presenting data from animal models, this is a requirement and is not optional. This is in addition to our well-established data-deposition policy for specific types of experiments and large datasets. Online readers of the manuscript will be able to access the graphical source data directly from the figure legend. Spreadsheets must be submitted in .xls, .xlsx or .csv formats. One file per figure is permitted. If there is a multi-panelled figure, the source data for each panel should be clearly labeled in the file; alternatively the source data for a figure can be included in multiple, clearly labeled sheets within an Excel file. File sizes of up to 30 MB are permitted, but it is expected that the vast majority of graphical source data files will be considerably smaller than this. When submitting these files with your manuscript, you should select the "Source Data" file type and use the title field in the file description tab to indicate the figure(s) to which the source data pertain.

RAW DATA (GELS): You must provide the original source images for all data obtained by electrophoretic separation (e.g., EMSA, northern/Southern/western blots, etc). The raw images should be assembled into a single .pdf or .tif file (multiple gels on a single page is encouraged). The file should be uploaded as Supplementary Figure 1. The full scanned images must be in uncropped form and contain labeled size/molecular weight markers and loading controls. There should be an accurate indication of how the gels were cropped for the final figure. The figure legends and raw data files should indicate whether controls (such as beta-actin) were run on the same gel as loading controls or on separate gels as sample processing controls (see here). While the data can be displayed in a relatively informal style, there must be a correspondence between each source data image and a specific main text or Extended Data figure. The main text or Extended Data figure legends should refer to the uncropped scans explicitly (e.g., "For gel source data, see Supplementary Figure 1."). For examples, see here or here.

THIRD PARTY RIGHTS: Please identify any content used in your article, whether in the main text, Extended Data or Supplementary Information, that comes from a third party. This could include figures, tables, images, videos or text boxes that are reproductions or adaptations of items that have previously been published elsewhere and/or are owned by a

third party. It also encompasses pictures taken by professional photographers, maps and images downloaded from the internet. You must obtain the right to use each of these items and provide evidence that you have these rights. You will also need to give proper attribution to the copyright holders in your paper. We ask that you fill out a Third party rights table and upload this with your revised manuscript. You can find more information about third-party rights here.

The revised paper should be submitted using the link below:

https://mts-nature.nature.com/cgi-bin/main.plex?el=A1K7G7A3MKWo7J1A9ftdtsu5zKsvmKIrygwW3dGRwZ

***NOTE**: This url links to your confidential home page and therefore to manuscripts you may have submitted or be reviewing for us. If you wish to forward this email to co-authors, please delete the link to your home page first.*

In the meantime, we hope that you will find our referees' comments helpful. Please do not hesitate to contact me if there is anything you would like to discuss.

Yours sincerely,

Sebastián Villamizar Santamaría
Senior Editor
Nature

Referee expertise:

Referee #1: IPV, economic abuse

Referee #2: banking econ, inequality

Referee #3: banking econ, inequality

Referee #4: economic sociology, banking

Referee #5: IPV, economic abuse

Referees' comments:

Referee #1 (Remarks to the Author):

Thank you for the opportunity to review the manuscript "Victim-survivors' banking records reveal the characteristics of financial abuse." It is a privilege to be one of the first to read this critical scholarship.

Summary of key results: This study uses administrative data from a major UK bank to look at patterns in financial behaviors among victim-survivors compared to a matched control group with no abuse disclosure. Findings from this study show that bank records provide a unique opportunity to examine the financial outcomes of victim-survivors and that victim-survivors experience increased debt, depleted savings, more frequent overdrafts, and a range of other financial indicators reflective of intimate partner violence at statistically significantly different rates compared to the control group.

Originality and significance: This study is novel and makes an important contribution to the financial abuse field. Once published, it will be cited frequently.

Data & methodology: The authors use a rigorous strategy for identifying outcomes to focus on, developing a control group, and conducting analyses to look at group differences and longitudinal patterns. Not only is the data quality high, it is also unique. The presentation of findings is thoughtfully done, especially considering the page limits and the amount of data they examine.

Appropriate use of statistics and treatment of uncertainties: Analyses appear appropriate and limitations are documented.

Conclusions: Conclusions are appropriate given the scope of the paper.

Thank you for your kind words.

Suggested improvements: This is very minor, but could "commuting-related expenses" be clarified? I think these may look different across international contexts and it would be helpful to better understand how it was operationalized here.

Commuting-related spend consists of payments to urban public transport companies. The largest merchant in this category is Transport for London (covering London underground and overground, buses and trams), followed by region-specific urban transport companies (predominantly bus and tram operators). At lines 316 in the revised version of the manuscript we have added in parenthesis: *"lower activity in commuting-related spend (**consisting of spend on urban public transport companies e.g., Transport for London**; difference -5.5pp, 95% CI, -6.9 to -4.1;"*.

References: Appropriately credit previous works.

Clarity and context: The manuscript (including abstract, introduction, and conclusion) are clearly written.

Laura Johnson

Referee #1 (Remarks on code availability):

The link did not seem to open properly for me when I clicked it.

Thank you for letting us know. The link should now provide access to the revised code. The previous link has worked for other reviewers, and we hope the new one will work for you as well. If you continue to experience difficulties, we would be happy to provide access via an alternative method.

Referee #2 (Remarks to the Author):

A. *Summary of the key results*

The paper examines retail bank data on customers which have self reported as victims of financial abuse. Using propensity score matching, a control group of similar customers is created using customer details as of 7 years prior to the reporting. The paper then compares trends in the two groups and demonstrates a decline in a number of characteristic outcomes of socioeconomic welfare as well as a set of broad characteristics that appear to characterize financial abuse victims leading up to disclosure that could potentially be used as predictive early warnings for financial institutions.

B. Originality and significance: if not novel, please include reference

The paper reads as reasonably novel and significant to me.

C. Data & methodology: validity of approach, quality of data, quality of presentation

The data quality and presentation seem reasonable to me.

The implementation of the methodology reads as basically correct to me, but I question whether it is appropriate to the stated or intended goals of the paper. The paper reads as having two goals:

1. Predicting the occurrence of financial abuse
2. Demonstrating the socioeconomic impact of financial abuse

If the goal is prediction, which I think is the more important goal as per the introduction, then creating a control sample via propensity score matching followed by monthly outcome comparisons seems inappropriate. It would be much more natural to want to predict the outcome out of the entire population. Some sort of a-priori category sorting may be warranted if the vast majority of eventual victims are from a certain monthly credit turnover bin or geographic deprivation rank, but in the context of prediction, a propensity score match reads as an ill-defined step in prediction. An explicit prediction model would also want to demonstrate the most important characteristics or combination of characteristics for identifying a likely victim from available bank data.

If the goal is demonstrating the socioeconomic impact, then the methodology is okay given the data limitations. However in this case many of the outcomes don't seem relevant to the question of socioeconomic welfare costs.

Thank you for your comments, which the editor also highlighted.

The primary goal of the analysis is to examine how financial abuse is reflected in the experiences of victim-survivors, using banking data. The matched control sample approach was constructed to achieve this goal. This approach provides a statistical counterfactual to the experience of victim-survivors over time, and we agree it is not appropriate for a predictive analytics exercise which might attempt to predict who from a population is at greatest risk of suffering financial abuse. We also agree that that some of the language used in the original manuscript implied causality.

We have therefore revised the main text to clarify that the goal of the paper is descriptive, not predictive and remove references to causal relationships.

Specifically, we have made the following changes to the manuscript:

Abstract:

- *"The ~~effects~~* ***experience*** *of financial abuse, a form of domestic abuse involving control over money and finances, can be severe and long-lasting, yet difficult to document. Current evidence* ***on how financial abuse shapes the everyday experiences*** *of ~~its impact on~~ victim-~~survivors' lives~~* ***survivors*** *is drawn primarily from small-scale, qualitative surveys."* Lines 30-33.
- *"Our results ~~show~~* ***demonstrate*** *how victim-survivors lose financial independence,* ***experience economic instability****, and ~~stability, and provide preliminary evidence that banking data could underpin a data-driven approach to mitigating foreseeable harm through early identification~~ suffer financial distress."* lines 43-44.

Introduction:

- *"However, the potential of financial data for understanding ~~the impact~~* ***victim-survivors experience*** *of financial abuse remains largely untapped."* lines 133-134.
- *"In addition, financial data allows us to follow the same individual over time, capturing ~~the evolving impact of economic abuse on~~* ***how financial abuse is associated with changes in*** *victim-survivors' financial lives."* Lines 146-148.
- *"The matched-design approach ~~controlled~~* ***controls*** *for socio-economic confounds, ~~allowing us to characterize the impact~~* ***, enabling the comparison*** *of ~~financial abuse on~~ victim-survivors' financial wellbeing* ***with controls*** *over time. The outcomes analyzed here span a wide range of behaviors, including financial balances, spending patterns and account management~~,~~****. To our knowledge, this is*** *~~providing~~ the first empirical illustration of* ***the experience of*** *financial ~~abuse's impact~~* ***abuse*** *~~on~~* ***in*** *victim-survivors' everyday lives* ***using banking data****.* Lines 166-172

Results:

- *"To ~~identify~~* ***define*** *the subset of 373 indicators ~~characterizing the effects of~~* ***reflecting differences associated with*** *financial abuse, we first identified"* lines 175-177
- *"~~Decreased spend~~* ***Decreased spending*** *on self beyond basic necessities ~~may reflect the impact of~~* ***aligns with patterns of*** *financial control ~~– either through restriction tactics or exploitation leaving victim-survivors with less money via increased borrowing~~* ***documented in prior studies, including restricted access to funds and financial exploitation, both of which have been linked to reduced financial autonomy among victim-survivors"*** lines 273-276
- *"Higher spending in off-licence stores is consistent with prior evidence showing that victim-survivors may use alcohol as a coping strategy~~, which may even be encouraged by the abuser to increase control~~"*-lines 2812-284

- *"The pattern of increased cash usage ~~might reflect~~ **is consistent with a range of experiences reported in the literature, including** financial exploitation through unauthorized or forced cash withdrawals aiming to deplete the victim-survivor's resources"* lines 307-310
- *"Lower commuting-related spending in the VS group ~~is in line with earlier results showing lower likelihood of employment and could result from sabotaging employment, while increased petrol spend may signal the victim-survivor's attempts to maintain safety and access support which may involve longer journeys. Access to taxis can provide a quick and discreet emergency escape option particularly when shared vehicles are being tracked by the abuser, and an analysis of escape journeys of women found that taxis were the second most common option after transport provided by friends and family.~~ **aligns with earlier findings of lower employment prevalence among victim-survivors. Higher spending on flexible transport options, including cars and taxis, has been reported previously and may reflect the complex, constrained circumstances in which victim-survivors meet everyday needs, including limited alternatives, time pressures and safety considerations."*** lines 319-323
- *"All of these activities ~~may be linked to the abuser taking control of bank accounts or victim-survivors seeking to take back control~~ **reflect changes in account access and management consistent with those reported in situations involving financial abuse, including both shifts in control by an abuser and increased engagement by victim-survivors** post-separation, when it may be safe to do so. Frequent ~~address changes are likely to reflect separation and the victim-survivors' efforts to prevent the abuser from discovering their new location~~ **changes of address align with the residential instability often associated with separation"*** lines 333-337

Discussion

- *"To our knowledge, ~~the present study~~ **this** is the first ~~to draw on~~ **study using** administrative, objective data ~~sources~~ to ~~document the impact of~~ **describe how** financial abuse ~~on~~ **manifests in** victim-survivors' lives"* Lines 458-459
- *"**Observed** changes in cash usage, transport spend**ing**, and account management activity ~~may reflect victim-survivors' efforts to escape their abuser~~ **are consistent with patterns reported around periods of safety-seeking or separation among victim-survivors.**"* Lines 500-502
- *"~~The results presented here, while descriptive, offer preliminary evidence for the feasibility of developing risk indicators that could underpin proactive support strategies and minimize the risk of foreseeable harm.~~"*
- We also added the following limitation: "***our methodological approach does not allow us to establish causality between financial abuse and observed outcomes. For example, there may be two-way relationship between financial abuse and financial distress, or factors jointly driving abuse and distress.***" lines 544-546
- *"Despite these limitations, this study shows that financial data provides a valuable lens to understand the ~~impact~~ **experience** of financial abuse. The results presented in*

*this study indicate that banking data ~~has the potential to underpin the~~ **data could guide future efforts to** develop~~ment of risk~~ **vulnerability** indicators and intervention strategies **designed** to ~~prevent harm and~~ support victim-survivors."* lines 550-554

We also believe the reference in the title to "the characteristics of financial abuse" avoids causal claims which might be construed by using terms such as "impact" or "effects" [of financial abuse].

On the relevance of outcomes, we believe that the broad set of outcomes considered in the paper is relevant for illustrating the experiences associated with financial abuse. Our analysis highlights the multi-dimensional experiences of victim-survivors, encompassing not only financial outcomes such as balances and debts, but also their sources of income (including the receipt of benefits linked to health-related vulnerabilities and reduced likelihood of employment), reduced spending on self-care (consistent with a trade-off between increased financial pressure and wellbeing-related spend), increased private transport spend (potentially reflecting increased time constraints and competing everyday pressures), and increased fraud victimisation (reflecting reduced financial control). A key contribution of our analysis is that these dimensions can be observed and quantified in transaction data, which reflect lived experiences across multiple domains. We have now emphasized the multi-domain nature of these experiences in the introduction at lines 117-120: ***"For the victim, loss of control over their financial life often co-occurs with difficulties across non-financial domains of life, including mental and physical health, the ability to pay for personal goods, and participation in leisure and well-being activities"***

More importantly, the paper should provide some minimal guidance on how to interpret the magnitude of the mean differences.

It seems reasonable that financial abuse would lead to things like overdraft charges, declining consumption, or an increase in unpaid debt. However it is difficult to get a sense of the scale of the problems without comparing them to values from existing research. Alternatively, a comparison to the distribution of outcomes within the sample of all bank customers could provide context to the values. This is important not just for qualifying the overall economic magnitude of any given effect, but in characterizing which specific negative outcomes are the most extreme and therefore most in need of attention by support organizations.

Thank you for this insightful and constructive suggestion. We agree that contextualizing the differences we report is important. To this end, we created two additional samples: a Bank population benchmark sample, comprising of 19,059 randomly selected bank customers, and a Female bank population benchmark sample, comprising of 18,906 randomly selected female customers. We report the 373 outcomes analysed previously for these samples in the year prior to disclosure in Supplementary Table 4. These estimates are directly comparable to the VS and Control means reported in the same period in Supplementary Table 3. More details about sample construction can be found in the Methods (lines 655-669).

To help the reader put the differences in context, when reporting VS-Control differences we also report the population estimate of the mean of the sample of all bank customers, together with the 95% confidence interval around that estimate e.g. at line 224 "*difference -1.8pp, 95% CI, -2.6 to -1.1**; Bank pop. benchmark: 3.9%, 95% CI, 3.6 to 4.2***".

In the discussion, we call out examples associated with financial harm where we observe the largest differences (lines 464-479):

"The findings presented in this paper highlight considerations for measuring the socioeconomic costs of financial abuse. In the year preceding disclosure, compared to the broader bank population (Supplementary Tables 2 and 4), victim-survivors were more likely to experience adverse financial outcomes: the percentage with at least one missed direct debit was 279% higher (63% vs 17%), while those relying on Universal Credit welfare benefit was 421% higher (51% vs 10%). Similarly, compared to the bank population's averages, victim-survivors' credit scores were 20% lower (767 vs 960), and their savings balances were 78% lower (£3,088 vs £14,215).

Victim-survivors' financial experiences vary in severity, with bank disclosures likely reflecting more adverse cases. However, given the magnitude of the harms identified - including damaged credit scores - and the estimated prevalence of financial abuse, these costs are likely to be economically significant. Previous estimates place the annual cost of domestic abuse in the UK at £89 billion (2025 prices), equivalent to ~7% of expected public expenditure in the UK in 2024-25. Owing to data limitations, this estimate excludes costs associated with financial abuse. By quantifying these financial harms, our results could inform extensions of national cost models to capture the socioeconomic burden of domestic abuse more comprehensively."

We thank the reviewer again for this suggestion, which we have incorporated and believe has strengthened the manuscript.

As per the direction of Nature, I have inspected and run the sample code and with small modification it appears to execute as intended, and I don't see any obvious errors with it. Some small suggestions vis-a-vis the code. It's usually best practice to either include the packages in the code repository itself, or to request them from CRAN and PyPi with a specific version so that package updates do not change replicability. The data and output paths should probably also just be specified as a relative reference to the execution directory rather than an absolute path to the host's computer, which would make the code auto-execute without edits. Alternatively, if absolute paths are important, they could be parceled out to an explicit configuration file.

Thank you for this suggestion relating to providing or calling specific versions of the packages. We have revised the code package and made the following changes: 1) the script now uses a path relative to the execution directory and 2) specific versions of packages are automatically installed and loaded (in R we use the renv package used for creating reproducible environments and in Python packages are installed via a requirements.txt).

D. Appropriate use of statistics and treatment of uncertainties

The raw statistical treatment seems basically correct to me, modulo the above comments regarding the methodology.

E. Conclusions: robustness, validity, reliability

The nature of the data creates obvious limitations, but they are acknowledged in the text. Given the limited research into this question, and the preliminary nature of the findings, I don't view this as an issue.

F. Suggested improvements: experiments, data for possible revision

Suggestions for improvement primarily relate to my comments on Methodology (C) and Clarity and context (H). I think the research is interesting and valuable, but that the paper should emphasize whether the goal is pre-preemptive prediction of abuse or quantification of the effects of abuse, and clarify the testing and results as giving more specific context to either one.

Thank you for this suggestion. Please see our responses to C above, in which we clarify that our aim is to describe how financial abuse is reflected in the experiences of victim-survivors, using banking data. Please also see our response to point H below.

G. References: appropriate credit to previous work?

I am not well versed in this area, but the reference read as reasonably complete to me.

H. Clarity and context: lucidity of abstract/summary, appropriateness of abstract, introduction and conclusions

The abstract and introduction are okay, but I had a fair amount of confusion about exactly what was meant by a victim-survivor of financial abuse. A lot of text is dedicated to explaining the problems associated with financial abuse, but I felt unclear as to exactly what is being measured. This is critical because it's the actual identifier of the the treatment and control group. How the central identifier comes to exist should be made clear earlier and more directly in the introduction.

Specifically, how does this self-disclosure come to be? Is it voluntary, or is it elicited by the bank? If it's voluntary, what motivates the victim to self-disclose? If it's elicited by the bank, what prompts the bank to ask about financial abuse? How and why does the bank classify a disclosure as financial abuse and record it as such?

Thank you for this comment, this is an important point that was raised by multiple reviewers. In the revised version of the manuscript, we provide a more detailed description of how victim-survivors come to disclose their experiences to the bank in the introduction (lines 154-161): ***"Victim-survivors self-disclosed their experiences to the bank's specialist financial and domestic abuse team (VS group; N = 5,428). Bank staff are trained to identify potential vulnerabilities, including financial and domestic abuse, during routine interactions (e.g., querying transactions, clarifying charges). When staff judge that a customer may have experienced domestic or financial abuse and might benefit from further support, they offer a referral to the bank's specialist team. With consent from the customer, the specially trained team contacts the customer to understand more about the nature and experience of abuse and, where possible, provide personalized support. The most common forms of support provided are separation of joint accounts, temporary suppression of charges, and signposting to relevant charities."***

Whilst bank staff is trained to identify potential signs of vulnerability, (recorded) disclosure is always voluntary, as per the explicit consent principle of the TEXAS framework[7] widely adopted by financial institutions in the UK. There are several reasons[8] why customers may choose to disclose vulnerability, including, but not limited to, avoiding the need to repeatedly explain distressing experiences to different staff; allowing extra checks on future account

activity (e.g., unusually large transactions) to reduce potential harm; opting out of standard processes that assume full control over finances and might unintentionally cause adverse outcomes (e.g., automated letters); and accessing payment flexibility, such as temporary suppression of charges.

In the UK, banks record customer vulnerability primarily to meet regulatory expectations under the Financial Conduct Authority's (FCA, the UK regulator responsible for supervising banks and ensuring fair treatment of consumers) Consumer Duty[9], which requires firms to identify and take account of vulnerability to ensure fair customer outcomes. Recording this information enables banks to apply proportionate adjustments to standard processes (for example, in communication, fraud monitoring, or collections), and to evidence compliance in the event of complaints or regulatory review. This information is not used to deny access to credit or other financial products or for labelling customers permanently.

Additionally, the structure of the introduction and abstract make it unclear whether the primary takeaway should be one of predicting financial abuse or examining the socioeconomic impact of financial abuse. I understand that the results are meant to be preliminary, but by not committing to one idea or another, or at least cleanly distinguishing the two, it was hard for me as a reader to get a handle on how I should be evaluating the empirical test. It seems from my reading that the primary purpose of the research is to predict abuse from financial data, but the tests lean more towards examining outcomes. Clarifying this would make interpreting the results easier for the reader.

Thank you for this additional request for clarification.

We have modified the abstract and introduction to clarify that the contribution of our paper is to describe how the experience of financial abuse manifests in banking records.
Abstract:

- *"The ~~effects~~* ***experience*** *of financial abuse, a form of domestic abuse involving control over money and finances, can be severe and long-lasting, yet difficult to document. Current evidence* ***on how financial abuse shapes the everyday experiences*** *of ~~its impact on~~ victim-~~survivors' lives~~* ***survivors*** *is drawn primarily from small-scale, qualitative surveys."* Lines 30-33.
- *"Our results ~~show~~* ***demonstrate*** *how victim-survivors lose financial independence,* ***experience economic instability****, and ~~stability, and provide preliminary evidence that banking data could underpin a data-driven approach to mitigating foreseeable harm through early identification~~ suffer financial distress."* lines 43-44.

Introduction:

- *"However, the potential of financial data for understanding ~~the impact~~* ***victim-survivors experience*** *of financial abuse remains largely untapped."* lines 133-134.
- *"In addition, financial data allows us to follow the same individual over time, capturing ~~the evolving impact of economic abuse on~~* ***how financial abuse is associated with changes in*** *victim-survivors' financial lives."* Lines 146-148.
- *"The matched-design approach ~~controlled~~* ***controls*** *for socio-economic confounds, ~~allowing us to characterize the impact~~* ***, enabling the comparison*** *of ~~financial abuse~~*

> ~~*on*~~ *victim-survivors' financial wellbeing* ***with controls*** *over time. The outcomes analyzed here span a wide range of behaviors, including financial balances, spending patterns and account management*~~*,*~~***. To our knowledge, this is*** ~~*providing*~~ *the first empirical illustration of* ***the experience of*** *financial* ~~*abuse's impact*~~ ***abuse*** ~~*on*~~ ***in*** *victim-survivors' everyday lives* ***using banking data****.* Lines 166-172

In addition, we have undertaken the edits described under point C to clarify the contribution of the paper.

Referee #2 (Remarks on code availability):

I have inspected and run the sample code and with small modification it appears to execute as intended, and I don't see any obvious errors with it. Some small suggestions vis-a-vis the code. It's usually best practice to either include the packages in the code repository itself, or to request them from CRAN and PyPi with a specific version so that package updates do not change replicability. The data and output paths should probably also just be specified as a relative reference to the execution directory rather than an absolute path to the host's computer, which would make the code auto-execute without edits. Alternatively, if absolute paths are important, they could be parceled out to an explicit configuration file.

Thank you for this suggestion relating to providing or calling specific versions of the packages. Please see our response to your earlier comment above.

Referee #3 (Remarks to the Author):

Referee Report for "Victim-survivors' banking records reveal the characteristics of financial abuse" submitted to Nature

Summary and general assessment of the paper

This paper studies an interesting question, the possible impact of financial abuse on a range of individual financial and non-financial outcomes. Using data from a major UK retail lending institution, the authors investigate the association between individuals' reported financial abuse and the occurrence of a wide set of outcomes, which range from individual borrowing and expenditures patterns, to credit scores changes, to travel expenses, to reliance on the UK safety net.

This is a policy-relevant issue. While the analysis is potentially interesting and overall presented with care, I have a number of fundamental concerns about the analysis, the methods and the interpretation of the results. As I explain below, because of these relevant limits, in my opinion it is hard to learn sufficiently robust and insightful lessons from the paper.

Comments

1) My first concern regards the possibility of making claims of causal direction based on the descriptive analysis. A reader can be naturally concerned that the indicators of financial distress, impaired (or excess) use of resources, reliance on safety net, etc. are themselves driving conflicts and vulnerability to financial abuse inside households and, hence, causing financial abuse, rather than being induced by financial abuse. The reason we observe an association between credit access (i.e. a low credit score) and financial abuse is that the motivation and incentive to engage in financial abuse inside a household could be more pronounced when an individual is financially weaker inside the household. Similarly, the reliance of an individual on the external safety net could exacerbate or moderate the likelihood that financial abuse occurs.

In a different but related vein, there could be other factors jointly driving a situation of distress and financial abuse, for example.

For all these reasons, while it is interesting to observe associations and descriptive patterns, it's hard to make a step further claiming causality. The analysis is instead presented with the goal of reaching conclusions about the impact of financial abuse on individual outcomes.

We thank the reviewer for raising this important point, which was also highlighted by the editor. We agree that causal direction cannot be inferred from the analysis presented, as the design of the study (matched control group approach) does not allow us to distinguish between financial conditions that precede, co-occur with, or follow financial abuse. These limitations reflect the potential for reverse causality and for unobserved factors to influence both financial distress and the occurrence of abuse.

Our analysis does match the victim-survivor group to a control group comparable on observables seven years prior to disclosure of the abuse and the two groups are very similar in outcomes at that point in time (see Supplementary Table 2 and Supplementary Figures 4-6). As you note, there could, however, be important unobservable factors jointly driving both

exposure to financial abuse and the outcomes we observe. There is not available a research design that could create randomised or as-good-as randomised variation in exposure to financial abuse.

To clarify that the aim is to describe victim-survivors' experiences rather than to estimate the impact of financial abuse, we have made the following changes to the manuscript:
Abstract:

- *"The ~~effects~~* ***experience*** *of financial abuse, a form of domestic abuse involving control over money and finances, can be severe and long-lasting, yet difficult to document. Current evidence* ***on how financial abuse shapes the everyday experiences*** *of ~~its impact on~~ victim-~~survivors' lives~~* ***survivors*** *is drawn primarily from small-scale, qualitative surveys."* Lines 30-33.
- *"Our results ~~show~~* ***demonstrate*** *how victim-survivors lose financial independence,* ***experience economic instability****, and ~~stability, and provide preliminary evidence that banking data could underpin a data-driven approach to mitigating foreseeable harm through early identification~~ suffer financial distress."* lines 43-44.

Introduction:

- *"However, the potential of financial data for understanding ~~the impact~~* ***victim-survivors experience*** *of financial abuse remains largely untapped."* lines 133-134.
- *"In addition, financial data allows us to follow the same individual over time, capturing ~~the evolving impact of economic abuse on~~* ***how financial abuse is associated with changes in*** *victim-survivors' financial lives."* Lines 146-148.
- *"The matched-design approach ~~controlled~~* ***controls*** *for socio-economic confounds, ~~allowing us to characterize the impact~~* ***, enabling the comparison*** *of ~~financial abuse on~~ victim-survivors' financial wellbeing* ***with controls*** *over time. The outcomes analyzed here span a wide range of behaviors, including financial balances, spending patterns and account management~~,~~****. To our knowledge, this is*** *~~providing~~ the first empirical illustration of* ***the experience of*** *financial ~~abuse's impact~~* ***abuse*** *~~on~~* ***in*** *victim-survivors' everyday lives* ***using banking data****.* Lines 166-172

Results:

- *"To ~~identify~~* ***define*** *the subset of 373 indicators ~~characterizing the effects of~~* ***reflecting differences associated with*** *financial abuse, we first identified"* lines 175-177
- *"~~Decreased spend~~* ***Decreased spending*** *on self beyond basic necessities ~~may reflect the impact of~~* ***aligns with patterns of*** *financial control ~~– either through restriction tactics or exploitation leaving victim-survivors with less money via increased borrowing~~* ***documented in prior studies, including restricted access to funds and financial exploitation, both of which have been linked to reduced financial autonomy among victim-survivors"*** lines 273-276
- *"Higher spending in off-licence stores is consistent with prior evidence showing that victim-survivors may use alcohol as a coping strategy~~, which may even be encouraged by the abuser to increase control~~"*-lines 2812-284

- *"The pattern of increased cash usage ~~might reflect~~* ***is consistent with a range of experiences reported in the literature, including*** *financial exploitation through unauthorized or forced cash withdrawals aiming to deplete the victim-survivor's resources"* lines 307-310
- *"Lower commuting-related spending in the VS group ~~is in line with earlier results showing lower likelihood of employment and could result from sabotaging employment, while increased petrol spend may signal the victim-survivor's attempts to maintain safety and access support- which may involve longer journeys. Access to taxis can provide a quick and discreet emergency escape option particularly when shared vehicles are being tracked by the abuser, and an analysis of escape journeys of women found that taxis were the second most common option after transport provided by friends and family.~~* ***aligns with earlier findings of lower employment prevalence among victim-survivors. Higher spending on flexible transport options, including cars and taxis, has been reported previously and may reflect the complex, constrained circumstances in which victim-survivors meet everyday needs, including limited alternatives, time pressures and safety considerations."*** lines 319-323
- *"All of these activities ~~may be linked to the abuser taking control of bank accounts or victim-survivors seeking to take back control~~* ***reflect changes in account access and management consistent with those reported in situations involving financial abuse, including both shifts in control by an abuser and increased engagement by victim-survivors*** *post-separation, when it may be safe to do so. Frequent ~~address changes are likely to reflect separation and the victim-survivors' efforts to prevent the abuser from discovering their new location~~* ***changes of address align with the residential instability often associated with separation"*** lines 333-337

Discussion

- *"To our knowledge, ~~the present study~~* ***this*** *is the first ~~to draw on~~* ***study using*** *administrative, objective data ~~sources~~ to ~~document the impact of~~* ***describe how*** *financial abuse ~~on~~* ***manifests in*** *victim-survivors' lives"* Lines 458-459
- *"**Observed** changes in cash usage, transport spend**ing**, and account management activity ~~may reflect victim-survivors' efforts to escape their abuser~~* ***are consistent with patterns reported around periods of safety-seeking or separation among victim-survivors.*** *"* Lines 500-502
- *"~~The results presented here, while descriptive, offer preliminary evidence for the feasibility of developing risk indicators that could underpin proactive support strategies and minimize the risk of foreseeable harm.~~"*
- We also added the following limitation: "***our methodological approach does not allow us to establish causality between financial abuse and observed outcomes. For example, there may be two-way relationship between financial abuse and financial distress, or factors jointly driving abuse and distress.***" lines 544-546
- *"Despite these limitations, this study shows that financial data provides a valuable lens to understand the ~~impact~~* ***experience*** *of financial abuse. The results presented in*

this study indicate that banking data ~~has the potential to underpin the~~ ***data could guide future efforts to*** *develop~~ment of risk~~* ***vulnerability*** *indicators and intervention strategies* ***designed*** *to ~~prevent harm and~~ support victim-survivors."* lines 550-554

2) In many cases the outcomes can be driven by other forms of physical and psychological abuse occurring at the same time as the financial abuse that is the object of interest here. I could not see an attempt to really isolate financial abuse from other forms of abuse, and the reader is then left wondering what is happening in the background. Indeed, in the presentation of the analysis the authors stress how various forms of abuse very often occur simultaneously.

Moreover, in various cases the explanations offered for the effects appear to indeed relate to other forms of abuse, rather than specifically to financial abuse. It would be important to control as accurately as possible for the possible simultaneous occurrence of other forms of abuse. A change in the use of some type of transportation, or in the reliance on borrowing, can be driven by physical and psychological abuse occurring at the same time as financial abuse, and the resulting need of individuals to resort to external care and support, or to finance medical expenses.

We thank the reviewer for raising this important point on the interrelationship between financial abuse and other forms of abuse. We agree that financial abuse frequently co-occurs with other forms of abuse, including physical and psychological abuse and that the outcomes we observe are often interrelated with these broader experiences. This co-occurrence is not incidental but central to the concept of coercive control, which is widely understood as a pattern of behaviours through which control is exercised across multiple domains of life rather than as isolated, separable acts. For example, a survey[5] for 4,009 adults in the UK found that 89% of women who experienced economic abuse have also experienced at least one other form of abuse (emotional, sexual or physical). For this reason, attempting to fully disentangle financial abuse from other concurrent forms of abuse presents substantial conceptual and empirical challenges.

We also agree with your contention that the patterns of financial transactions we analyse imply the experiences of victim-survivors are not just financial (i.e. increased debt), but extend to other domains of life, for example, reduced likelihood of employment, reduced spending on self-care, and increased private transport spend. We interpret these financial patterns not as solely being related to financial abuse, but as financial manifestations of a victim-survivor's wider lived experience. Our goal is to document the multi-domain experiences that are associated with disclosures of financial abuse as they appear in real-world banking data.

We have revised the manuscript to position financial abuse within the wider context of coercive control, and to highlight the co-occurrence of different types of abuse, as well as the multidimensional nature of victim-survivors' experience:

- *"**While research on domestic abuse has mostly focused on physical violence, many scholars stress that violence must be understood within the wider context of coercive control, which can also encompass psychological, sexual, economic and financial abuse**."* Lines 101-103

- ***“Financial abuse is often intertwined with other aspects of coercive control (e.g., emotional, sexual or physical abuse)[5], reflecting a broader constellation of harms that may include stress, injury, reduced work capacity, social isolation and poorer financial wellbeing.”*** Lines 109-112
- ***“For the victim, loss of control over their financial life often co-occurs with difficulties across non-financial domains of life, including mental and physical health, the ability to pay for personal goods, and participation in leisure and well-being activities.”*** Lines 117-120

3) I liked the richness of the outcomes considered. At the same time, it's hard to make sense of all the possible effects. In fact, some of the explanations offered are somewhat stretched, and refer somewhat loosely to previous studies. In light of this and also of point 2 above, my suggestion would be to focus the attention on a more limited set of outcomes for which the mechanisms of influence of financial abuse can be clearer and that (point 2) are more tightly related to financial abuse specifically.

We appreciate the reviewer’s recognition of the richness of the outcomes analyzed.

We acknowledge the reviewer’s concern that some of the explanations provided may appear stretched. To more explicitly position our findings in the context of prior research on victim-survivors’ experiences, and remove references to causal pathways, we have made the following changes to the manuscript:

- *“~~Decreased spend~~* ***Decreased spending*** *on self beyond basic necessities ~~may reflect the impact of~~* ***aligns with patterns of*** *financial control ~~– either through restriction tactics or exploitation leaving victim-survivors with less money via increased borrowing~~* ***documented in prior studies, including restricted access to funds and financial exploitation, both of which have been linked to reduced financial autonomy among victim-survivors”*** lines 273-276
- *“Higher spending in off-licence stores is consistent with prior evidence showing that victim-survivors may use alcohol as a coping strategy~~, which may even be encouraged by the abuser to increase control~~”*-lines 282-284
- *“The pattern of increased cash usage ~~might reflect~~* ***is consistent with a range of experiences reported in the literature, including*** *financial exploitation through unauthorized or forced cash withdrawals aiming to deplete the victim-survivor’s resources”* lines 307-312
- *“Lower commuting-related spending in the VS group ~~is in line with earlier results showing lower likelihood of employment and could result from sabotaging employment, while increased petrol spend may signal the victim-survivor’s attempts to maintain safety and access support, which may involve longer journeys. Access to taxis can provide a quick and discreet emergency escape option particularly when shared vehicles are being tracked by the abuser, and an analysis of escape journeys of women found that taxis were the second most common option after transport provided by friends and family.~~* ***aligns with earlier findings of lower employment prevalence among victim-survivors. Higher spending on flexible transport options, including***

cars and taxis, has been reported previously and may reflect the complex, constrained circumstances in which victim-survivors meet everyday needs, including limited alternatives, time pressures and safety considerations.” lines 319-323

- *“All of these activities ~~may be linked to the abuser taking control of bank accounts or victim-survivors seeking to take back control~~* ***reflect changes in account access and management consistent with those reported in situations involving financial abuse, including both shifts in control by an abuser and increased engagement by victim-survivors*** *post-separation, when it may be safe to do so. Frequent ~~address changes are likely to reflect separation and the victim-survivors' efforts to prevent the abuser from discovering their new location~~* ***changes of address align with the residential instability often associated with separation”*** lines 333-337
- *“**Observed** changes in cash usage, transport spend**ing**, and account management activity ~~may reflect victim-survivors' efforts to escape their abuser~~* ***are consistent with patterns reported around periods of safety-seeking or separation among victim-survivors.*** *”* Lines 500-502

To further probe the robustness of the findings, we have conducted two additional analyses, first by creating a distress-matched sample (N=5,183 victim-survivors; N=13,430 controls) to isolate patterns associated with abuse from general financial distress, and second by utilizing a relationship-end matched sample (N=1,149 victim-survivors; N=3,236 controls) to examine whether the observed trends are specific to the pre-separation period. While the results largely replicate the findings reported in the primary matched sample, where we find differences they provide additional valuable insights into the experiences of victim-survivors (lines 367-416):

- ***“Results from the distress-matched sample (Supplementary Figure 2, Supplementary Table 5) show that a close match is achieved for financial-distress related variables (Panels B-D). The VS group show distinctive patterns compared with the distress-matched control sample, replicating the main comparison for variables including benefit receipt, cash withdrawals, account access and security, and bank interactions.***
- ***The VS-Control difference observed in disability benefit receipt (difference, 14.3pp, 95% CI 13 to 15.7) is almost double of that observed in the primary matched sample (difference 7.9pp, 95% CI, 6.6 to 9.3). This amplified difference highlights health-related vulnerabilities as a potentially important pathway into financial distress among victim-survivors, beyond that observed in financially distressed women without victim-survivor status. In the UK, it is estimated that disabled adults - defined as those living with a long-standing illness, condition, or impairment - are 2.6 times more likely to experience domestic abuse compared to non-disabled adults.***
- ***No evidence for VS-Control difference in optician spend was found (difference -0.5pp, 95% CI, -2 to 1), and estimated differences were smaller for dental (difference -3.4pp, 95% CI, -4.9 to -1.9 vs difference -8.8pp, 95% CI, -10.2 to -7.4 in the primary matched sample), and furniture spend (difference -4.5, 95% CI, -6 to -***

2.9 vs difference -8pp, 95% CI, -9.5 to -6.5 in the primary matched sample). Attenuated differences in self-care spend suggest that the experience of victim-survivors of financial abuse in these dimensions are not distinctive from women in financial distress without a victim-survivor status.

- *Interestingly, in contrast to results from the primary matched sample, no strong evidence of VS-Control differences in the distress-matched sample were found for petrol (difference 0.8pp, 95% CI, -0.5 to 2 vs difference 5pp, 95% CI, 3.8 to 6.3 in the primary matched sample) and taxi spend (difference 2.5pp, 95% CI, 0.9 to 4.1 vs difference 12.3pp, 95% CI, 10.8 to13.9 in the primary matched sample). This suggests that previously observed differences in these spending categories are not distinct from those seen in women who experience financial distress but not financial abuse. Such financial distress-related travel pressures may include irregular income, reduced flexibility, and time constraints.*
- *Finally, the distress-matched results reveal distinctive bank interaction patterns for victim-survivors. The direction of the VS-Control difference was reversed for internet banking logins (difference -3.4, 95% CI, -5.3 to -1.5 vs difference 10.1, 95% CI, 8.3 to 11.9 in the primary matched sample). This suggests that, although increased internet banking logins are associated with financial distress, victim-survivors tend to rely more on human banking channels. This may reflect barriers to accessing internet banking, or the greater complexity and sensitivity of their needs, which are less easily addressed through digital channels.*
- *Results from the relationship-end matched sample (Supplementary Figure 3, Supplementary Table 6) were consistent with those observed in the primary matched sample, although estimated VS-Control differences were generally smaller and several estimates were centered near zero, reflecting reduced statistical power given the smaller sample size.*
- *Specifically, in the relationship-end matched sample, no evidence for VS-Control difference was found for payroll (difference -1.1pp, 95% CI, -2.6 to 0.5 vs difference -1.8pp, 95% CI, -2.6 to -1.1 in the primary matched sample), optician (difference -2.3pp, 95% CI, -5.3 to 0.7 vs difference -2.9pp, 95% CI, -4.3 to -1.5 in the primary matched sample), and sport (difference 1.3pp, 95% CI, -2 to 4.7 vs difference -5.3pp, 95% CI, -6.9 to -3.8 in the primary matched sample) spend.*
- *Observed VS-Control differences were larger for cash withdrawal (difference 7pp, 95% CI, 5.4 to 8.5 vs difference 4pp, 95% CI, 3.3 to 4.7 in the primary matched sample), and petrol spend (difference 10.1pp, 95% CI, 7.4 to 12.7 vs difference 5pp, 95% CI, 3.8 to 6.3 in the primary matched sample). The larger differences in cash withdrawals and petrol spending around relationship end compared to disclosure date may reflect practical demands associated with separation, including relocation or increased travel."*

We have carefully considered the suggestion to focus on a more limited set of outcomes to provide a clearer link to financial abuse. However, for the reasons explained in point 2, we believe that narrowing the scope would risk omitting the multi-domain reality that characterizes victim-survivors' lived experience of financial abuse and coercive control.

4) It would be important to discuss and address much more carefully the possible issues of sample bias. The sample is based on data from a banking institution and, more specifically, a large retail bank. A reader can then wonder to what extent clients of this large retail bank are representative of the population (this problem is briefly acknowledged in the conclusions).

We thank the reviewer for raising this point. In Supplementary Table 1, we provide comparisons of the characteristics of the Lloyds Banking Group (LBG) population with the UK population (using UK population data[3] provided by the Office for National Statistics[4]; ONS). These tables compare the two populations by age, gender, region and income. They demonstrate that the LBG population is representative of the UK population, closely matching population distributions for each variable. This is as expected, given that Lloyds bank is one of the UK's largest retail bank with 28m customers, serving approximately 51% of the UK adult population. We have added a sentence at lines 151-152 of the revised version of the paper "*The retail bank serves approximately 51% of the UK adult population, and is representative of the population by age, gender, region and income (see Supplementary Table 1)"*. More details about the samples used for the comparison can be found in the Methods at lines 738-747.

An issue of sample selection can also stem from the permanence in the sample. The borrowers who continue to maintain an account rather than exiting the sample or switching to a different bank can be different, for example more or less resilient to financial abuse. This could lead to underestimate or overestimate the association between financial abuse and individual outcomes.

To examine the impact of the filtering criteria applied, we compared the characteristics of the VS candidate sample (N = 5,444) with those who were filtered out (N = 4,284) when applying criteria (1)-(4) at lines 566-570 in the Methods. This comparison focuses exclusively on characteristics unaffected by account activity: age, credit score, and deprivation rank. Other metrics, such as income, are less accurately measured for inactive bank customers who did not meet out transaction criteria, and therefore the comparison would not be informative. These results are shown in Supplementary Table 11.

First, we compared the mean age at the start of the individual-specific period. We found that compared to VS individuals included in the final sample, excluded VS candidates were on average 5 years younger at the start of the individual-specific period (difference 4.9 years, 95% CI, 4.5 to 5.2), reflecting the minimum age threshold (18) imposed at the start of the individual-specific time period.

Second, we compared the mean difference in credit scores and deprivation rank between the first and last year of the individual-specific analysis period. The decline in average credit scores between the first and last year of the individual-specific period was slightly larger amongst included VS individuals compared to excluded VS candidates (difference -13.3 points, 95% CI, -24.3 to -2.5). In contrast, we found no evidence for differences in deprivation rank change between the start and the end of the analysis period (difference 296 ranks, 95% CI, -37 to 636) by inclusion status.

Overall, while the included VS sample is slightly older than excluded candidates, we find no evidence suggesting that permanence in the sample is systematically associated with differential changes in credit scores or deprivation rank. This analysis is included in the Methods at lines 765-769.

5) Among the various outcomes, I was relatively more intrigued by the financial outcomes, as they might be somewhat more closely connected to financial abuse specifically rather than to other forms of abuse. However, I was somewhat confused by the interpretation of the results for financial outcomes. It appears that borrowers more exposed to financial abuse tend to borrow more but also that they use more cash. Moreover, borrowing and, plausibly, borrowing capacity rise but credit scores tend to decline. Are these interpreted as supply side or demand side effects in the credit market? In most cases there is an attempt to provide a demand-driven rationale, but the actual extension of loans (e.g., credit conditions, costs, rationing) will also likely reflect the status of the individual borrowers. So I would expect quite complex interactions between supply side and demand side effects.

We thank the reviewer for raising these points. On the first point, we believe that the tendency of victim-survivors to on average borrow more and also use more cash as not inconsistent with one another. Additional borrowing, such as more use of bank overdrafts, credit cards and personal loans, could be accessed by the victim-survivor as cash via ATM withdrawals either using their debit card or credit card.

On the second point, we agree that our narrative in the first version of the paper alluded to demand-side effects whereby victim-survivors are more likely to demand credit and debt. We agree that there could have been a mechanism where, for various reasons, victim-survivors may not have shown more demand compared to the control group, but instead were more successful in obtaining credit due to greater access (credit supply). We have been able to look into this issue directly using information on credit applications. We compare application rates and access rates for the matched victim-survivor and control groups, with results shown in Supplementary Figure 1. Results show that victim-survivors have higher application rates for credit cards and loans, but do not have higher success rates. For other financial products there are no differences in application and success rates between the groups.

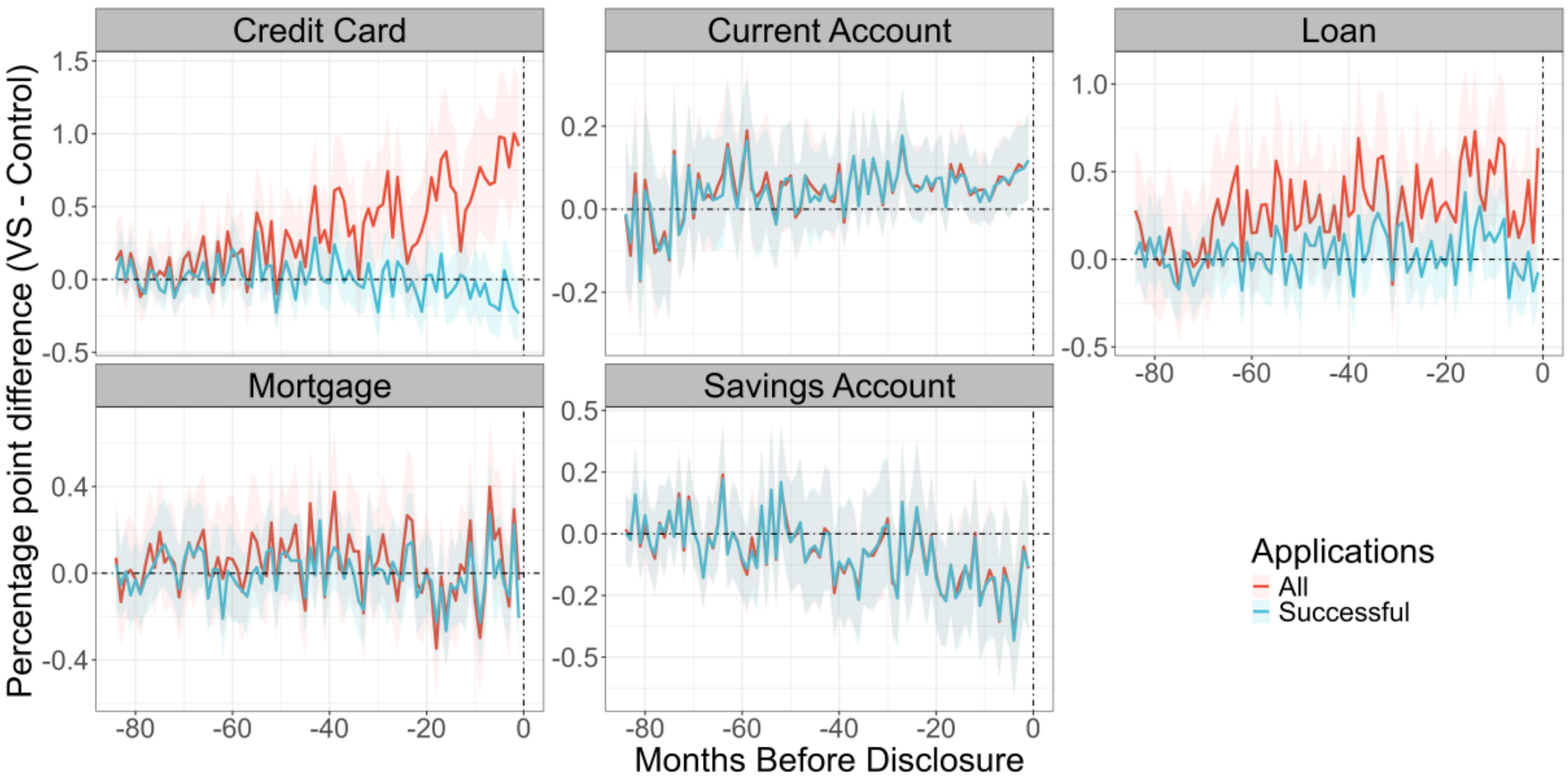

Supplementary Figure 1 Victim-survivor and Control financial product applications (primary matched sample). VS-Control percentage point differences in the number of total, and successful financial product applications (credit card, current account, loan, mortgage, savings account) in the 7 year period prior to disclosure month. Results show that victim-survivors have higher application rates for credit cards and loans, but do not have higher success rates. There are no differences in application and success rates between the groups for other financial products. For methodological details, see "Financial product applications and application outcomes".

We conclude that this provides evidence against a hypothesis a supply-side explanation for our results whereby victim-survivors are more successful in accessing credit. We have added at lines 244-246: ***"Additional analysis shows that compared to Control, the VS group increases the number of applications submitted for credit (credit cards and personal loans) over time, with no corresponding difference in success rates, indicating that higher debt levels are at least partly a result of higher demand for credit (Supplementary Figure 1)."***

6) It would be interesting to see a more detailed discussion about the generalizability of the results beyond the specific setting considered here. Is there any concern that the structure of families in the UK might affect the link between financial abuse and outcomes? Further analysis of the characteristics of the UK financial system would also be useful, as this structure can affect the link between financial abuse and financial outcomes.

We thank the reviewer for this important comment regarding the generalizability of the findings reported. We agree that both family structures and financial system characteristics can profoundly shape how financial abuse manifests and how it translates into observed financial outcomes.

With respect to family structure, the UK context is characterised by relatively high rates of cohabitation[10], legal protections that differ by marital status, and comparatively permissive norms around separation. These features may influence both the prevalence and consequences of financial abuse, and imply that some mechanisms documented here, particularly around post-separation financial adjustment, may differ in settings where separation is socially penalised or legal protections are weaker.

With respect to the financial system, the UK has high levels of bank account penetration[11], individualised retail banking, and widespread use of digital payments, which facilitate the observation of financial behaviours in transaction data. While rates of financial abuse are comparable across the UK and other high-income countries such as the United States[12], the extent to which our findings generalise to contexts with different banking infrastructures or where economic control operates primarily through unpaid or informal labour is likely to be more limited.

We have revised the Discussion to make these contextual considerations explicit and to clarify that our findings are most directly applicable to similar institutional and cultural settings, while highlighting the need for further work in other contexts:

"Our study has several limitations. ***First, the victim-survivor group, comprising of women in the UK with bank accounts who disclosed their victim-survivor status to the bank, and have met our sample selection criteria is not necessarily representative of the average victim-survivor, especially if disclosure is associated with experiencing significant financial distress. The findings reported here are most directly applicable to contexts with high levels of financial inclusion and individualized banking systems, such as the UK and may not generalize to international contexts, where financial abuse is shaped by different social and cultural norms, nor to settings where gendered and unpaid labor constitute central forms of economic control. Further work is needed to understand the extent to which the results reported in this specific group are generalizable to other populations."***
Lines 522-531

7) What are the possible policy implications of the observed associations, including the

possible estimates of the costs of financial abuse for the society at large? What could a financial institution or a policymaker do in order to break down the observed links? I think it would be interesting to at least give a sense of the implications for policymakers and the social costs of the observed mechanisms.

We recognize the importance of discussing the implications for estimating the societal cost of financial abuse. To this end, we created two additional samples: a Bank population benchmark sample, comprising of 19,059 randomly selected bank customers, and a Female bank population benchmark sample, comprising of 18,906 randomly selected female customers. We report the 373 outcomes analysed previously for these samples in the year prior to disclosure in Supplementary Table 4. These estimates are directly comparable to the VS and Control means reported in the same period in Supplementary Table 3. More details about sample construction can be found in the Methods (lines 655-669).

To help the reader put the differences in context, when reporting VS-Control differences we also report the population estimate of the mean of the sample of all bank customers, together with the 95% confidence interval around that estimate e.g. at line 224 "*difference -1.8pp, 95% CI, -2.6 to -1.1**; Bank pop. benchmark: 3.9%, 95% CI, 3.6 to 4.2***".

In the discussion, we call out examples associated with financial harm where we observe the largest differences (lines 464-479):

"The findings presented in this paper highlight considerations for measuring the socioeconomic costs of financial abuse. In the year preceding disclosure, compared to the broader bank population (Supplementary Tables 2 and 4), victim-survivors were more likely to experience adverse financial outcomes: the percentage with at least one missed direct debit was 279% higher (63% vs 17%), while those relying on Universal Credit welfare benefit was 421% higher (51% vs 10%). Similarly, compared to the bank population's averages, victim-survivors' credit scores were 20% lower (767 vs 960), and their savings balances were 78% lower (£3,088 vs £14,215).

Victim-survivors' financial experiences vary in severity, with bank disclosures likely reflecting more adverse cases. However, given the magnitude of the harms identified - including damaged credit scores - and the estimated prevalence of financial abuse, these costs are likely to be economically significant. Previous estimates place the annual cost of domestic abuse in the UK at £89 billion (2025 prices), equivalent to ~7% of expected public expenditure in the UK in 2024-25. Owing to data limitations, this estimate excludes costs associated with financial abuse. By quantifying these financial harms, our results could inform extensions of national cost models to capture the socioeconomic burden of domestic abuse more comprehensively."

In addition, we discuss further potential policy implications in the discussion:

"The findings presented in this paper also have implications for the policy discourse on how to improve support for victim-survivors. For example, estimated group differences in average credit scores could inform the design of credit rating system reforms, aiming to support victim-survivors regain financial independence and stability. Subsequently, estimated differences in selected financial indicators reported here could be used as a benchmark for assessing victim-survivors' financial wellbeing in the context of such reforms." Lines 480-498

8) The results are sufficiently well presented and described. The abstract and introduction of

the paper are sufficiently clear. I have no particular concerns on these fronts. Perhaps, I would suggest highlighting the nature of the data used and their limits earlier in the introduction, and with more detail. Moreover, the paper makes reference to the previous literature in a clear way.

We thank you for your kind words. Providing greater clarity about the VS sample selection earlier on in the paper was requested by multiple reviewers.

In the revised version of the manuscript, we provide a more detailed description of how victim-survivors come to disclose their experiences to the bank in the introduction (lines 152-161): ***"Victim-survivors self-disclosed their experiences to the bank's specialist financial and domestic abuse team (VS group; N = 5,428). Bank staff are trained to identify potential vulnerabilities, including financial and domestic abuse, during routine interactions (e.g., querying transactions, clarifying charges). When staff judge that a customer may have experienced domestic or financial abuse and might benefit from further support, they offer a referral to the bank's specialist team. With consent from the customer, the specially trained team contacts the customer to understand more about the nature and experience of abuse and, where possible, provide personalized support. The most common forms of support provided are separation of joint accounts, temporary suppression of charges, and signposting to relevant charities."***

Referee #3 (Remarks on code availability):
The code files appear to be in good order and the instructions well written.

Thank you.

Referee #4 (Remarks to the Author):

A. Summary of the Key Results

This paper seeks to demonstrate that financial data can be used to construct risk indicators for financial abuse—an issue that accompanies domestic abuse in approximately 95% of cases.

Drawing on data provided by one of the United Kingdom's largest financial institutions (Lloyds Banking Group), the authors compare the financial behavior of a general sample with that of a cohort of women who have self-identified as victim-survivors of financial abuse, over a seven-year period. From an initial set of more than 400 indicators, 171 variables showing statistically significant differences were retained for analysis.

The results reveal significant disparities between the two groups across multiple domains: higher levels of debt and overdraft use among victim-survivors, a decline in savings, distinctive spending and cash withdrawal patterns, and increased interactions with banking services.

The paper concludes by arguing that banking data can serve as a valuable tool for the prevention of domestic abuse, as key behavioral differences can be systematically observed. Such data could be used not only to detect financial abuse but also to inform policy interventions designed to help victim-survivors regain financial stability—for instance, through improved credit scoring methodologies.

B. Originality and Significance

The topic addressed in this article is of considerable importance. It highlights the financial dimension of domestic violence—crucial both for identifying early warning signs and for understanding the economic dynamics that entrap women in abusive relationships, as well as the long-term economic consequences they face.

A further contribution of the paper lies in demonstrating that banking data represent an invaluable resource for detecting situations of risk.

While the notion that banks should engage with the issue of financial abuse is not entirely new—see, for example, Ayesha Scott's Financial Abuse in a Banking Context: Why and How Financial Institutions Can Respond (Journal of Business Ethics, 2023)—Scott's work adopts an ethical perspective and relies primarily on qualitative data.

The originality of this research lies in its use of quantitative data and in its effort to design empirical indicators. Moreover, thanks to the longitudinal character of the banking data, the authors are able to reconstruct seven years of financial history for the participants, which allows for a temporally grounded analysis.

We thank you for your kind words. We found Scott's research highly relevant to the focus of this work and have included it as a reference regarding the responsibilities of financial institutions in supporting victim-survivors.

C. Data and Methodology: Validity of Approach, Data Quality, and Presentation

A central methodological issue concerns the data on victim-survivors, which originate from within the bank itself. Although the authors acknowledge limitations regarding their sample, the implications of these limitations for the validity of the findings are not sufficiently examined.

In particular, the first limitation—that only women who contacted the bank's Specialist Domestic and Financial Abuse Team are included—deserves closer scrutiny. It would be essential to assess the representativeness of this group: what proportion of the bank's female clientele does it represent, and how does that compare to the estimated prevalence of financial abuse in the wider population? Are there demographic, geographic, or socioeconomic specificities within the sample?

We thank the reviewer for raising this important point regarding the representativeness of the victim-survivor sample.

In the calendar year 2024, 10,165 female bank customers self-disclosed being a victim-survivor of domestic abuse via the process described above. This count only includes individuals who provided explicit consent for their vulnerability to be recorded. Overall, Lloyds Banking Group has 7.8m active female customers, meaning that the proportion of women disclosing to the bank is approximately 0.13%.

Survey estimates designed to capture victim-survivors across the full spectrum of severity report a substantially higher prevalence, around 9.1% over a comparable period[13]. It is reasonable to expect that women disclosing to the bank represent the more severe or extreme end of experiences, which explains much of the difference between survey-based and bank-based prevalence.

For additional context, domestic abuse crime statistics for England and Wales provide another point of reference. In the year ending March 2025, the UK Crown Prosecution Service charged 53,919 suspects[13] with domestic abuse. While gender breakdowns are not reported, assuming 94% of perpetrators are male[14], this corresponds to approximately 50,684 male offenders, or a 0.21% offending rate among men (or 0.11% in the overall population[3]). Although this is not a direct comparison, since victimisation rates in the bank data are being compared to criminal charges, it suggests that the prevalence of self-disclosure to the bank is broadly consistent with other proxies for the prevalence of severe domestic abuse in the population.

We assessed the representativeness of the VS sample by comparing the distribution of key demographic characteristics - age group, region of residence, and disability status - with corresponding estimates from a nationally representative survey conducted by the Office for National Statistics (ONS)[4]. Comparisons were restricted to victim-survivors residing in England and Wales to align with the geographic coverage of the survey benchmarks, which together comprise ~89% of the UK population. Age in the LBG sample was calculated as of December 2024. Variables were selected based on the availability of comparable measures across data sources. Supplementary Table 10 presents the percentage distribution of these characteristics for the VS sample restricted to England and Wales (N = 4,875) alongside the corresponding ONS estimates. The LBG sample includes women aged over 18 years at the start of the 7-year analysis period; consequently, the youngest participant was aged 25 years in 2024. In the LBG sample, disability is inferred from disability benefit receipt.

ONS-LBG proportion differences in the 16-24 age groups are by design of the LBG sample. Aside this, compared with ONS estimates, the LBG sample included a higher proportion of victim-survivors aged 35–44 years and a slightly lower proportion aged ≥55 years. Regional distributions and the estimated proportion with a disability were similar across samples.

Given the centrality of this issue, a more detailed description of the Domestic and Financial Abuse Team should be integrated into the main text, not confined to the methodological appendix. How do potential victims become aware of this service? What forms of assistance does it provide? Are individuals encouraged to contact it in cases of domestic abuse even when financial abuse is not explicitly present?

We thank you for raising these important points.

Multiple reviewers requested greater clarity around the construction of the VS sample. In the revised version of the manuscript, we provide a more detailed description of how victim-survivors come to disclose their experiences to the bank in the introduction (lines 152-161): ***"Victim-survivors self-disclosed their experiences to the bank's specialist financial and domestic abuse team (VS group; N = 5,428). Bank staff are trained to identify potential vulnerabilities, including financial and domestic abuse, during routine interactions (e.g., querying transactions, clarifying charges). When staff judge that a customer may have experienced domestic or financial abuse and might benefit from further support, they offer a referral to the bank's specialist team. With consent from the customer, the specially trained team contacts the customer to understand more about the nature and experience of abuse and, where possible, provide personalized support. The most common forms of support provided are separation of joint accounts, temporary suppression of charges, and signposting to relevant charities."***

Victim-survivors are told about this specialist team via frontline staff if they judge that the customer would benefit from further support. The most common forms of assistance are separation of joint accounts, temporary suppression of charges, and signposting to relevant charities. Referrals are made based on the impact on the customer's circumstances, so individuals experiencing a single form of coercive control, such as physical abuse, may also access personalized support from the specialist team.

A major concern—one acknowledged but not fully explored by the authors—is the potential selection bias whereby only individuals experiencing severe financial consequences are likely to contact this service, thereby skewing the sample.

Thank you for making this important point. We agree that the findings presented in the manuscript suggest that in many cases, financial distress is frequently intertwined with the criteria for entering our sample. In addition to the sensitivity analysis conducted to identify the VS-Control differences associated with financial distress (explained further below), we have undertaken further analysis to examine whether the VS sample contains only individuals experiencing severe financial consequences. To this end, we compared the average credit scores of the VS group during the year prior to disclosure against a control group of 18,906 randomly selected female customers (Female bank population benchmark) from the same period. Details on the construction of this sample can be found in the Methods section (lines 655-669), while Supplementary Figure 7 shows the comparison.

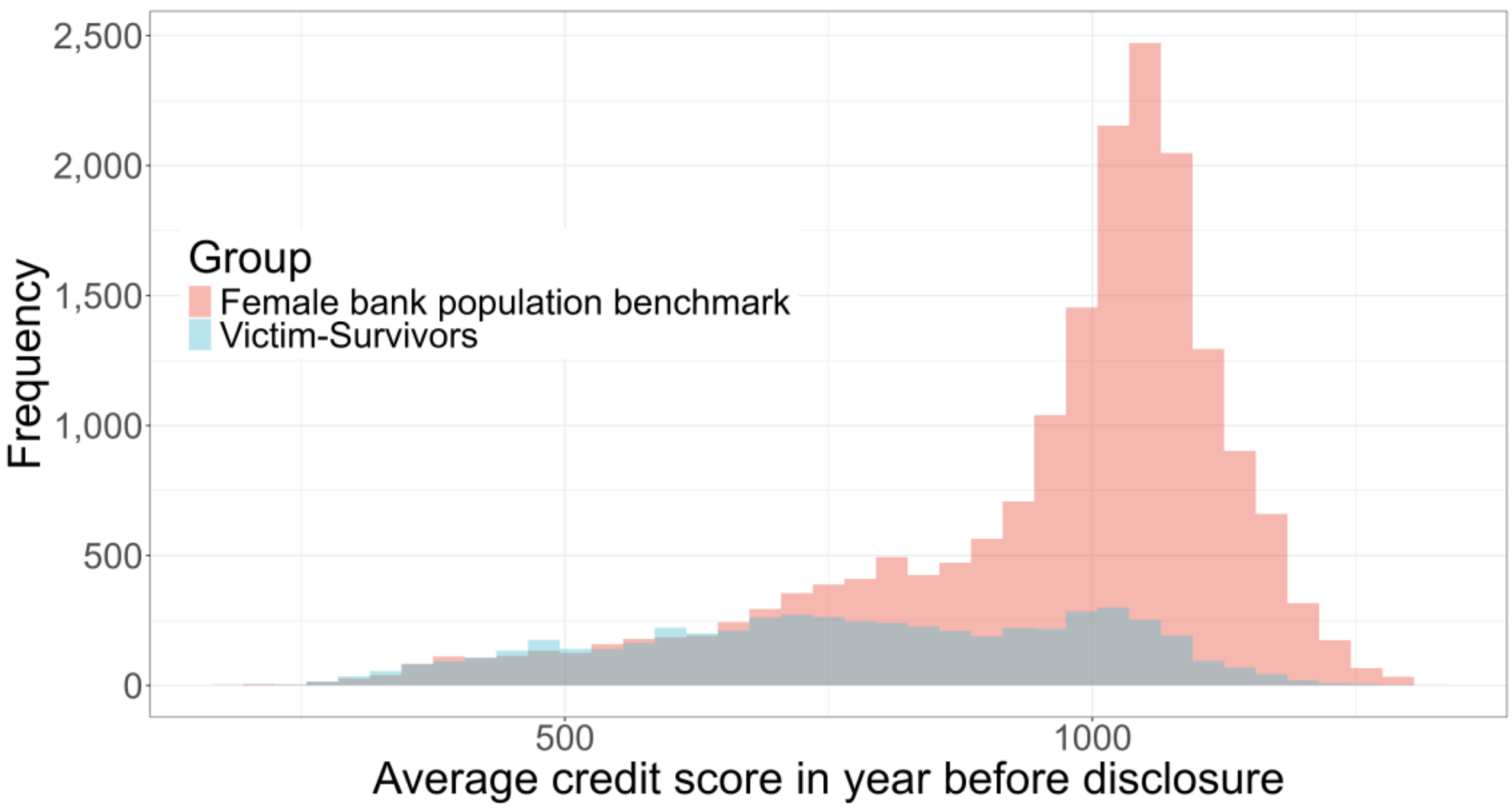

Supplementary Figure 7. Victim-survivor and Female bank population credit score distributions in the year prior to disclosure. The figure shows that while on average, credit scores are significantly lower in the VS group (as expected), we see a wide distribution spanning the entire interval of Control credit score values, suggesting that that the VS sample does not only include individuals in severe financial distress. For methodological details, see "Measuring the degree of self-selection by financial distress".

This reveals two patterns. First, as expected, the average credit score among victim-survivors is lower than that among the Female bank population benchmark group. Second, the plot illustrates that the victim-survivor group does not only contain individuals with severely low credit scores, but instead contains individuals with credit scores across the distribution of the Female bank population benchmark group. Overall, 35% of individuals in the victim-survivor group have credit scores that sit above the 25th percentile of credit score (887) for the Female bank population benchmark group. We agree with the reviewer comment that one might be concerned in particular if the victim-survivor group were heaped at very low credit scores and/or the plot illustrated a clear bi-modality between the two groups. We are reassured by the overlapping distributions that, while the credit score for victim-survivors is lower than that for the Female bank population benchmark group, our victim-survivor sample is not only limited to individuals experiencing severe financial situations.

Furthermore, the analysis would benefit from considering whether, in some cases, financial distress may have preceded the abusive relationship and contributed to victims' vulnerability, or conversely, whether financial hardship triggered recognition of the abuse and disclosure to the bank. Another limitation concerns the use of the disclosure date as a pivotal temporal marker. This date likely encompasses a variety of situations: some women may have long been separated and are seeking to rebuild financial stability, while others may still be cohabiting with their abuser and under ongoing coercive control. Their financial behaviors are therefore not directly comparable.

On the first point, as highlighted by multiple reviewers, the original version of the manuscript has given the impression that our analysis allows us to describe causal patterns in the data. This is not the case, and in the revised manuscript, we clarify that the primary goal of the analysis is to examine how financial abuse is reflected in the experiences of victim-survivors, using banking data. Findings from the descriptive analysis suggest that financial distress is often closely linked to the disclosure decision, but our methodological approach does not allow us to rule out that financial distress may also be a risk factor in victimization.

In response to the comment regarding alternative temporal markers, we have been able to re-interrogate the data and replicate the main results for a sample of victim-survivors where we were able to extract a temporal marker for when the relationship ended (lines 359-366):

"Second, we created a smaller relationship-end matched sample that included victim-survivors where information on relationship end date was available (N = 1,149) and corresponding matched controls (N = 3,236; 21% of the primary matched control sample). By comparing VS-Control differences at relationship end, rather than disclosure date, we can examine the extent to which the patterns reported are specific to the pre-separation period. Aside from defining the individual analysis period using the relationship end date, we followed the same matching procedure as in the primary sample. Supplementary Table 2 presents descriptive statistics for the VS and Control groups."

Lines 634-654 in the Methods explain our approach in more detail:

"To create the relationship-end date samples, we extracted relationship-end date information from the VS candidate sample. Applying the same criteria as described above, we retrieved notes associated with 5,303 victim-survivors. During appointments, with the customer's consent, bank staff records key details of each case in the form of notes. The richness of the information recorded vary by note, but some includes temporal markers, most commonly the relationship end date. We identified 3,559 notes that contained words indicative of a relationship end date. These records either included month names (e.g., "jan") or any the following search words: fled, split, ended, ago, left. We then manually reviewed these notes and extracted relationship end dates with month-level precision. For example, "relationship ended 3 years ago" was not accurate enough, but "fled 2 weeks ago" would be included with a relationship end date two weeks prior to disclosure. We retrieved the latest known contact date with the abuser at the time of the disclosure, and only if it was within our analysis period (after January 2019). Therefore, relationship end could be missing if it was not mentioned in the note, was too long ago, was not specific enough or if the victim-survivor was still in the relationship at the time of disclosure. The manual review resulted in a sample with 1,189 VS sample candidates. On average, relationships ended 7.7 months (s.d. 9.3) prior to disclosure date.
The matching variables were the same as those used in the primary matched sample, the only difference being that the individual-specific analysis period was now defined by the relationship-end date as opposed to disclosure date. The propensity matching process resulted in a final sample of 1,149 VS and 3,236 Control individuals. Supplementary Table 9 shows the output of the propensity score matching"

Using this sample, we replicated the main analysis with results shown in Supplementary Figure 3. We report the following in the Results (lines 401-416):

"Results from the relationship-end matched sample (Supplementary Figure 3, Supplementary Table 6) were consistent with those observed in the primary matched sample, although estimated VS-Control differences were generally smaller and several estimates were centered near zero, reflecting reduced statistical power given the smaller sample size.
Specifically, in the relationship-end matched sample, no evidence for VS-Control difference was found for payroll (difference -1.1pp, 95% CI, -2.6 to 0.5 vs difference -1.8pp, 95% CI, -2.6 to -1.1 in the primary matched sample), optician (difference -2.3pp, 95% CI, -5.3 to 0.7 vs difference -2.9pp, 95% CI, -4.3 to -1.5 in the primary matched

sample), and sport (difference 1.3pp, 95% CI, -2 to 4.7 vs difference -5.3pp, 95% CI, -6.9 to -3.8 in the primary matched sample) spend.
Observed VS-Control differences were larger for cash withdrawal (difference 7pp, 95% CI, 5.4 to 8.5 vs difference 4pp, 95% CI, 3.3 to 4.7 in the primary matched sample), and petrol spend (difference 10.1pp, 95% CI, 7.4 to 12.7 vs difference 5pp, 95% CI, 3.8 to 6.3 in the primary matched sample). The larger differences in cash withdrawals and petrol spending around relationship end compared to disclosure date may reflect practical demands associated with separation, including relocation or increased travel."

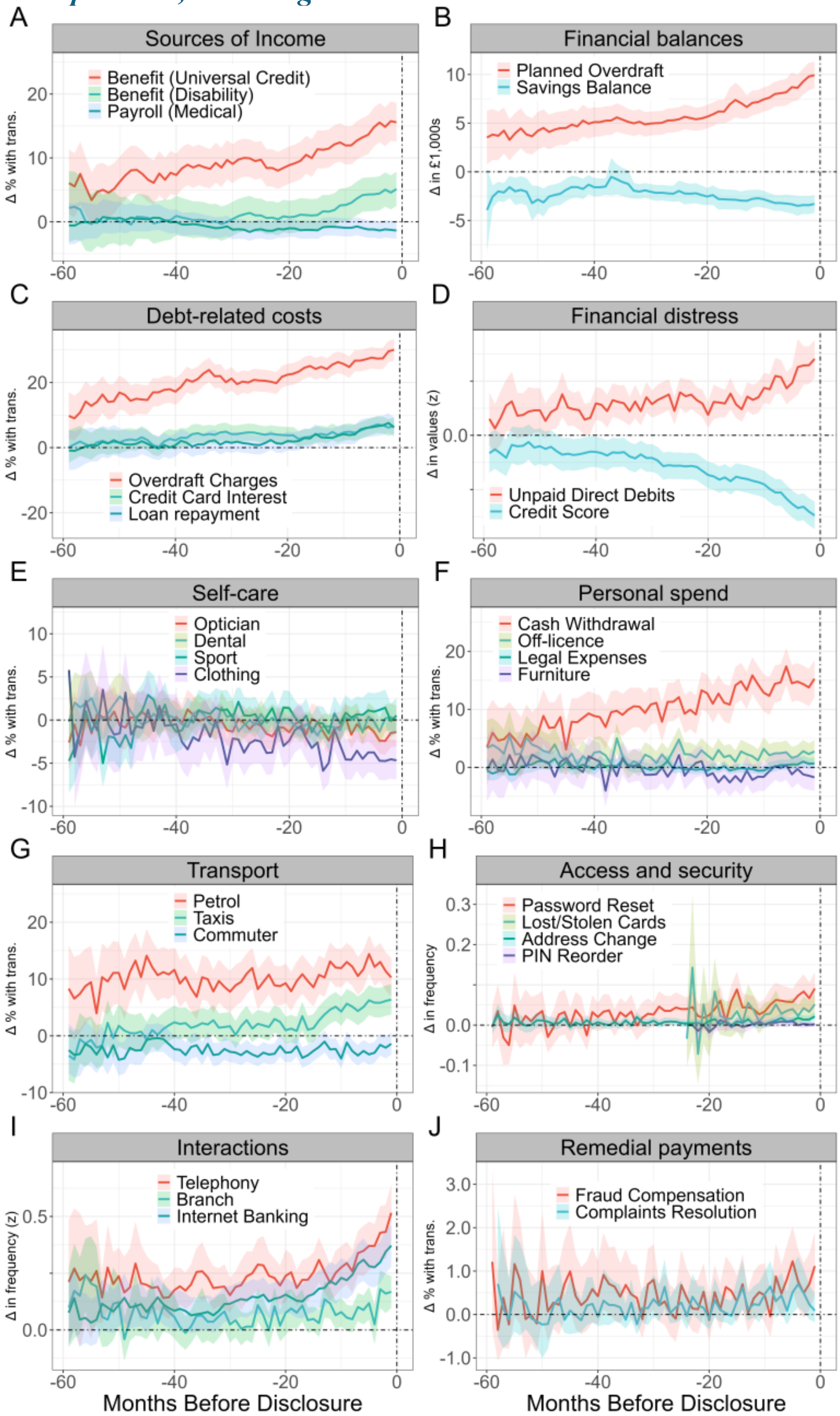

Supplementary Figure 3. Monthly VS-Control Group differences in selected financial indicators (relationship-end matched sample). Line plots showing monthly VS-Control group differences

(vertical axis). The horizontal axis shows the number of months relative to relationship end date (month = 0). Shading denotes 95% CI. Sample sizes: VS (N =1,149), Control (N =3,236). Data on Lost/Stolen cards and PIN reorder are only available from January, 2023. **Panel A. Sources of Income**. VS-Control difference in monthly group proportion with transactions. Exact (transactional) variables used: Benefits/Disability Living Allowance/Other, Benefits/Universal Credit/Other, Payroll/Medical/NHS. **Panel B. Financial balances**. VS-Control difference in monthly group averages (measured in thousand pounds). Exact (non-transactional) variables used: Planned Overdraft, Savings Balance. **Panel C. Debt-related costs.** VS-Control difference in monthly group proportion with transactions. Exact variables used: Credit Card Interest (derived transactional), Financial Services/Loans/Other (transactional), Overdraft charges (derived transactional). **Panel D. Financial distress.** VS-Control difference in scaled monthly group averages (measured in monthly credit scores, and monthly counts of missed direct debits). Exact variables used: Credit score (non-transactional), Unpaid direct debit (derived transactional). **Panel E. Self-care.** VS-Control difference in monthly group proportion with transactions. Exact (transactional) variables used: Retail/Clothing/Women, Services/Health/Dental, Services/Health/Optician, Hobbies & Interests/Sport/Other. **Panel F. Personal spend.** VS-Control difference in monthly group proportion with transactions. Exact variables used: ATM Withdrawal (derived transactional), Retail/Furniture/Other (transactional), Services/Professional/Legal (transactional), Retail/Food & Drink/Alcohol (transactional). **Panel G. Transport.** VS-Control difference in monthly group proportion with transactions. Exact (transactional) variables used: Travel/Commuter/Other, Motor/Petrol/Other, Travel/Taxis/Other. **Panel H. Access and security.** VS-Control difference in monthly frequency. Exact (non-transactional) variables used: Address Change, Lost/Stolen card, Password Reset, PIN Reorder. **Panel I. Interactions.** VS-Control difference in monthly scaled frequency. Exact (non-transactional) variables used: Branch visits, Internet Banking logins, Telephone visits. **Panel J.** Remedial payments. VS-Control difference in monthly group proportion with transactions. Exact (transactional) variables used: Financial Services/Fraud/Write Off, Financial Services/Other/Complaints Resolution.

Therefore, we conclude that despite the difference in sample sizes, the vast majority of VS-Control differences reported in the main analysis (primary matched sample) are still present when we use relationship end date as a temporal marker instead of disclosure date.

Finally, the treatment of banking data appears to assume that each participant holds a single account with Lloyds. Yet many may hold multiple accounts or loans with other institutions. These factors—central to financial life—are not accounted for in the analysis.

We thank the reviewer for raising this point. The analysis includes all accounts held with Lloyds Banking Group (LBG; for example, where individuals hold two checking accounts; or checking accounts, credit cards and other products). Whilst LBG has a relationship with over half the UK population, survey data suggest that an estimated 63% of UK adults are multi-banked[15], and therefore we agree that individuals are likely to hold accounts elsewhere, which are not observed by us.

Our methodological approach was developed with this caveat in mind. By imposing a strict transactional activity criterion for an extended period (up to 7 years), we aimed to ensure that individuals included in our final sample are those who most likely use LBG as their primary bank. While this does not preclude individuals in the sample from holding accounts with other institutions, we can be reasonably confident that our data captures the majority of their financial activity.

We also anticipate that this data gap most likely leads us to underestimate the financial distress effects of financial abuse, as victim-survivors' accounts held elsewhere may also show worsening financial balances, debt-related costs and financial distress. It seems unlikely that victim-survivors would experience financial distress with their accounts held by the bank

who data we use, and yet concurrently experience improving financial situations in accounts held elsewhere.

Some further reassurance on this issue is provided by analysis of credit score. The credit score used by the bank is sourced from a credit reference agency, which holds data on all accounts held by the individual (whether at the bank or at other banks / financial services providers). The deterioration in the credit score of victim-survivors relative to the control group indicates that the victim-survivors experience an overall worse financial situation over time.

Finally, to further assess whether our sample construction could bias the findings, an important concern given that we only observe activity within LBG, we conducted an additional analysis comparing the characteristics of victim-survivors by inclusion status. This comparison, described at lines 765–769 in the Methods and reported in Supplementary Table 11, shows that while the included VS sample is slightly older than excluded candidates, we find no evidence that inclusion (and thus sustained activity with LBG) is systematically associated with differential changes in credit scores or deprivation rank. This provides further reassurance that our results are unlikely to be driven by selection into the observed banking data.

In the revised manuscript, we acknowledge this in the Limitations (lines 537-541):

"First, the victim-survivor group, comprising of women in the UK with bank accounts who disclosed their victim-survivor status to the bank, and have met our sample selection criteria is not necessarily representative of the average victim-survivor, especially if disclosure is associated with experiencing significant financial distress. (...) Due to data availability restrictions, our analysis only looks at a maximum 7-year window ***and draws on data from one bank.*** *Since domestic abuse can span decades****, impacting all bank accounts held by the individual,*** *this timeframe* ***and data view*** *may ~~not fully capture the long-term financial markers of~~* ***reflect all of an individual's financial patterns associated with financial abuse.****"*

At lines 255-257 we have included the point that credit scores capture the overall financial position of the individual: "***Credit score measures individual creditworthiness using data from all bank accounts and financial products, including those beyond the bank used in our analysis, and summarizes the increasing financial strain experienced by the VS group relative to Control."***

D. Appropriate Use of Statistics and Treatment of Uncertainties

The study's approach—testing a large number of indicators independently—is useful as an exploratory step. However, the authors refrain from identifying broader behavioral patterns or developmental stages that could be operationalized by banks.

Each variable is tested separately, and the paper does not propose an analytical model linking these dimensions, which limits the robustness and interpretability of the findings. A cluster or typology analysis could have revealed recurrent combinations of behaviors among victim-survivors, potentially forming the basis for automated detection systems.

As it stands, this remains an initial stage of inquiry. There is an implicit suggestion that the study could directly yield predictive indicators, whereas substantial further work would be

required to achieve this goal. Moreover, no correlation analyses are performed; as a result, the explanations of observed behaviors remain tentative and largely hypothetical.

We thank you for raising this issue, which was also noted by Reviewer 2 and the editor. We agree that earlier wording may have implied that the study could directly yield predictive indicators, which was not our intention.

The primary aim of the analysis is descriptive: to examine how financial abuse is reflected in victim-survivors' experiences over time using banking data and a matched control design. This approach provides a statistical counterfactual and is not intended for predictive modelling or risk identification at the population level.

While typology or cluster analyses that jointly consider variables represent a valuable next step, our focus here is on the longitudinal trajectories of individual indicators across a seven-year period. Establishing these independent dynamics provides a necessary empirical foundation for future work developing behavioural profiles or predictive frameworks.

We have revised the manuscript to clarify this scope and remove language implying causal or predictive claims.

Abstract:

- *"The ~~effects~~* ***experience*** *of financial abuse, a form of domestic abuse involving control over money and finances, can be severe and long-lasting, yet difficult to document. Current evidence* ***on how financial abuse shapes the everyday experiences*** *of ~~its impact on~~ victim-~~survivors' lives~~* ***survivors*** *is drawn primarily from small-scale, qualitative surveys."* Lines 30-33.
- *"Our results ~~show~~* ***demonstrate*** *how victim-survivors lose financial independence,* ***experience economic instability****, and ~~stability, and provide preliminary evidence that banking data could underpin a data-driven approach to mitigating foreseeable harm through early identification~~ suffer financial distress."* lines 43-44.

Introduction:

- *"However, the potential of financial data for understanding ~~the impact~~* ***victim-survivors experience*** *of financial abuse remains largely untapped."* lines 133-134.
- *"In addition, financial data allows us to follow the same individual over time, capturing ~~the evolving impact of economic abuse on~~* ***how financial abuse is associated with changes in*** *victim-survivors' financial lives."* Lines 146-148.
- *"The matched-design approach ~~controlled~~* ***controls*** *for socio-economic confounds, ~~allowing us to characterize the impact~~* ***, enabling the comparison*** *of ~~financial abuse on~~ victim-survivors' financial wellbeing* ***with controls*** *over time. The outcomes analyzed here span a wide range of behaviors, including financial balances, spending patterns and account management~~,~~****. To our knowledge, this is*** *~~providing~~ the first empirical illustration of* ***the experience of*** *financial ~~abuse's impact~~* ***abuse*** *~~on~~* ***in*** *victim-survivors' everyday lives* ***using banking data****.* Lines 166-172

Results:

- *"To ~~identify~~* ***define*** *the subset of 373 indicators ~~characterizing the effects of~~* ***reflecting differences associated with*** *financial abuse, we first identified"* lines 175-177
- *"~~Decreased spend~~* ***Decreased spending*** *on self beyond basic necessities ~~may reflect the impact of~~* ***aligns with patterns of*** *financial control ~~– either through restriction tactics or exploitation leaving victim-survivors with less money via increased borrowing~~* ***documented in prior studies, including restricted access to funds and financial exploitation, both of which have been linked to reduced financial autonomy among victim-survivors"*** lines 273-276
- *"Higher spending in off-licence stores is consistent with prior evidence showing that victim-survivors may use alcohol as a coping strategy~~, which may even be encouraged by the abuser to increase control~~"*-lines 2812-284
- *"The pattern of increased cash usage ~~might reflect~~* ***is consistent with a range of experiences reported in the literature, including*** *financial exploitation through unauthorized or forced cash withdrawals aiming to deplete the victim-survivor's resources"* lines 307-310
- *"Lower commuting-related spending in the VS group ~~is in line with earlier results showing lower likelihood of employment and could result from sabotaging employment, while increased petrol spend may signal the victim-survivor's attempts to maintain safety and access support which may involve longer journeys. Access to taxis can provide a quick and discreet emergency escape option particularly when shared vehicles are being tracked by the abuser, and an analysis of escape journeys of women found that taxis were the second most common option after transport provided by friends and family.~~* ***aligns with earlier findings of lower employment prevalence among victim-survivors. Higher spending on flexible transport options, including cars and taxis, has been reported previously and may reflect the complex, constrained circumstances in which victim-survivors meet everyday needs, including limited alternatives, time pressures and safety considerations."*** lines 319-323
- *"All of these activities ~~may be linked to the abuser taking control of bank accounts or victim-survivors seeking to take back control~~* ***reflect changes in account access and management consistent with those reported in situations involving financial abuse, including both shifts in control by an abuser and increased engagement by victim-survivors*** *post-separation, when it may be safe to do so. Frequent ~~address changes are likely to reflect separation and the victim-survivors' efforts to prevent the abuser from discovering their new location~~* ***changes of address align with the residential instability often associated with separation"*** lines 333-337

Discussion

- *"To our knowledge, ~~the present study~~* ***this*** *is the first ~~to draw on~~* ***study using*** *administrative, objective data ~~sources~~ to ~~document the impact of~~* ***describe how*** *financial abuse ~~on~~* ***manifests in*** *victim-survivors' lives"* Lines 458-459
- *"**Observed** changes in cash usage, transport spend**ing**, and account management activity ~~may reflect victim-survivors' efforts to escape their abuser~~* ***are consistent***

with patterns reported around periods of safety-seeking or separation among victim-survivors. *"* Lines 500-502

- *"~~The results presented here, while descriptive, offer preliminary evidence for the feasibility of developing risk indicators that could underpin proactive support strategies and minimize the risk of foreseeable harm.~~"*
- We also added the following limitation: "***our methodological approach does not allow us to establish causality between financial abuse and observed outcomes. For example, there may be two-way relationship between financial abuse and financial distress, or factors jointly driving abuse and distress.***" lines 544-546
- *"Despite these limitations, this study shows that financial data provides a valuable lens to understand the ~~impact~~* ***experience*** *of financial abuse. The results presented in this study indicate that banking data ~~has the potential to underpin the~~* ***data could guide future efforts to*** *develop~~ment of risk~~* ***vulnerability*** *indicators and intervention strategies* ***designed*** *to ~~prevent harm and~~ support victim-survivors."* lines 550-554

We also highlight the importance of identifying the most effective set of indicators as the next step:

- *"Further research should identify the most effective set of indicators, evaluate their predictive validity, and explore whether data coverage could be improved by combining data from multiple financial institutions."* Lines 506-508

E. Conclusions: Robustness, Validity, and Reliability

The article's conclusions are appropriately cautious: it emphasizes that banking data hold promise for addressing violence against women and should be more systematically leveraged in the future. These conclusions are robust, albeit modest in scope.

Beyond these general claims, however, the paper also presents more specific results whose reliability is weaker, due to the methodological limitations outlined above.

F. Suggested Improvements: Additional Data and Possible Revisions

To strengthen the findings, it would be valuable to include a comparison group of women experiencing financial hardship—manifested in high debt levels and reduced savings—but without exposure to domestic or financial abuse. This would help isolate the behaviors specifically associated with financial abuse.

Thank you for this insightful and constructive suggestion. We agree that distinguishing between VS-Control differences associated with financial distress and those more specifically associated with the experience of financial abuse is an important extension of the analysis.

To this end, we have conducted an additional analysis where we compare the VS group to Control individuals who experience similar levels of financial distress at the time of disclosure (lines 356-358): ***"First, we created a distress-matched sample, pairing victim-survivors (N = 5,183) with controls (N = 13,430) who experienced similar levels of financial distress in the year before disclosure. This allows us to separate patterns associated with abuse from those related to financial distress."***

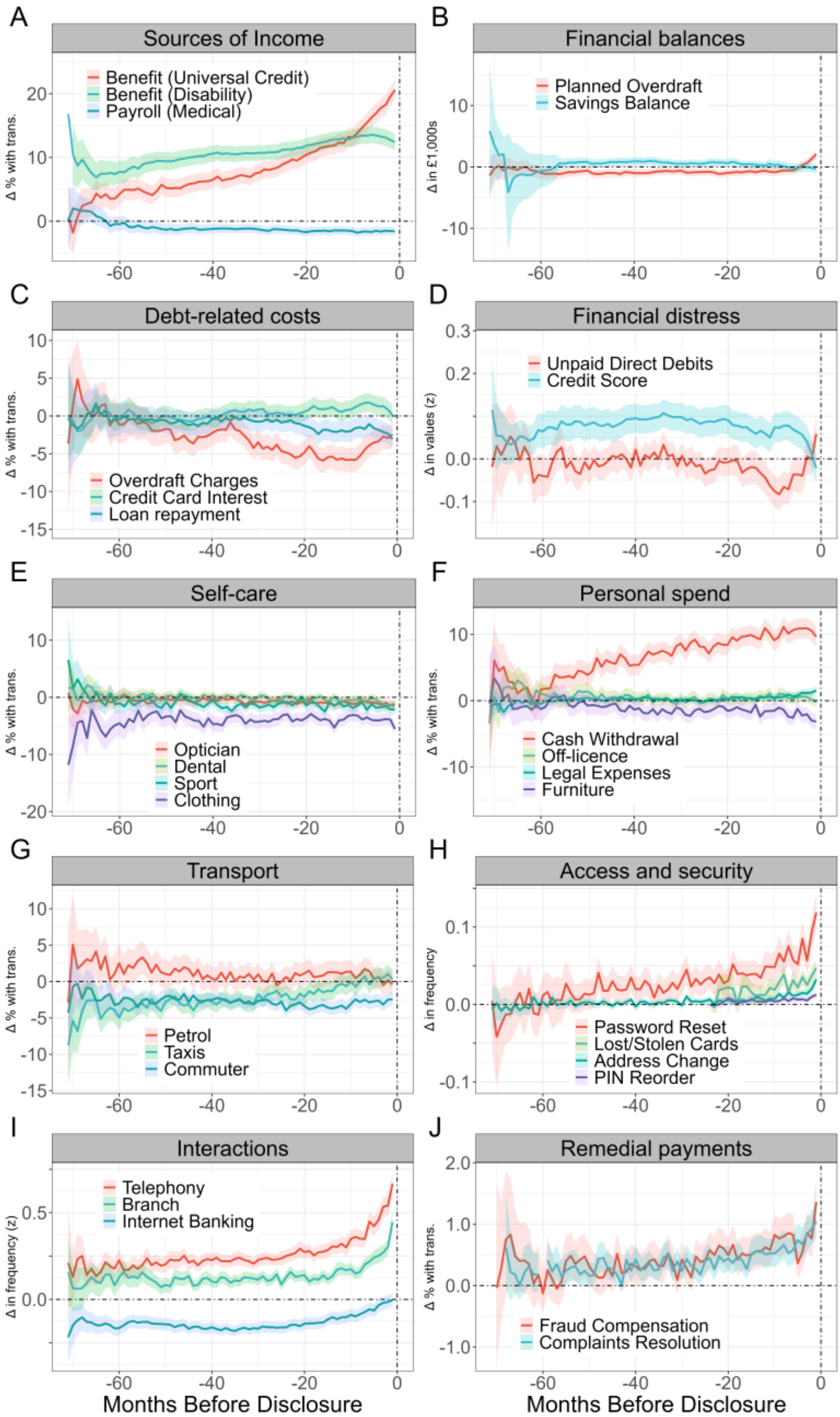
A
Sources of Income
Benefit (Universal Credit)
Benefit (Disability)
Payroll (Medical)
Δ % with trans.
B
Financial balances
Planned Overdraft
Savings Balance
Δ in £1,000s
C
Debt-related costs
Overdraft Charges
Credit Card Interest
Loan repayment
Δ % with trans.
D
Financial distress
Unpaid Direct Debits
Credit Score
Δ in values (z)
E
Self-care
Optician
Dental
Sport
Clothing
Δ % with trans.
F
Personal spend
Cash Withdrawal
Off-licence
Legal Expenses
Furniture
Δ % with trans.
G
Transport
Petrol
Taxis
Commuter
Δ % with trans.
H
Access and security
Password Reset
Lost/Stolen Cards
Address Change
PIN Reorder
Δ in frequency
I
Interactions
Telephony
Branch
Internet Banking
Δ in frequency (z)
J
Remedial payments
Fraud Compensation
Complaints Resolution
Δ % with trans.
Months Before Disclosure
Months Before Disclosure

Supplementary Figure 2. Monthly VS-Control Group differences in selected financial indicators (distress-matched sample). Line plots showing monthly VS-Control group differences (vertical axis). The horizontal axis shows the number of months relative to disclosure date (month = 0). Shading denotes 95% CI. Sample sizes: VS (N =5,183), Control (N =13,430). Data on Lost/Stolen cards and PIN reorder are only available from January, 2023. **Panel A. Sources of Income**. VS-Control difference in monthly group proportion with transactions. Exact (transactional) variables used: Benefits/Disability Living Allowance/Other, Benefits/Universal Credit/Other, Payroll/Medical/NHS. **Panel B. Financial balances**. VS-Control difference in monthly group averages (measured in thousand pounds). Exact (non-transactional) variables used: Planned Overdraft, Savings Balance. **Panel C. Debt-related costs.** VS-Control difference in monthly group proportion with transactions. Exact variables used: Credit Card Interest (derived transactional), Financial Services/Loans/Other (transactional), Overdraft charges (derived transactional). **Panel D. Financial distress.** VS-Control difference in scaled monthly group averages (measured in monthly credit scores, and monthly counts of missed direct debits). Exact variables used: Credit score (non-transactional), Unpaid direct debit (derived transactional). **Panel E. Self-care.** VS-Control difference in monthly group proportion with transactions. Exact (transactional) variables used: Retail/Clothing/Women, Services/Health/Dental, Services/Health/Optician, Hobbies & Interests/Sport/Other. **Panel F. Personal spend.** VS-Control difference in monthly group proportion with transactions. Exact variables used: ATM Withdrawal (derived transactional), Retail/Furniture/Other (transactional), Services/Professional/Legal (transactional), Retail/Food & Drink/Alcohol (transactional). **Panel G. Transport.** VS-Control difference in monthly group proportion with transactions. Exact (transactional) variables used: Travel/Commuter/Other, Motor/Petrol/Other, Travel/Taxis/Other. **Panel H. Access and security.** VS-Control difference in monthly frequency. Exact (non-transactional) variables used: Address Change, Lost/Stolen card, Password Reset, PIN Reorder. **Panel I. Interactions.** VS-Control difference in monthly scaled frequency. Exact (non-transactional) variables used: Branch visits, Internet Banking logins, Telephone visits. **Panel J.** Remedial payments. VS-Control difference in monthly group proportion with transactions. Exact (transactional) variables used: Financial Services/Fraud/Write Off, Financial Services/Other/Complaints Resolution.

We report the Results at lines 367-400: ***"Results from the distress-matched sample (Supplementary Figure 2, Supplementary Table 5) show that a close match is achieved for financial-distress related variables (Panels B-D). The VS group show distinctive patterns compared with the distress-matched control sample, replicating the main comparison for variables including benefit receipt, cash withdrawals, account access and security, and bank interactions.***

The VS-Control difference observed in disability benefit receipt (difference, 14.3pp, 95% CI 13 to 15.7) is almost double of that observed in the primary matched sample (difference 7.9pp, 95% CI, 6.6 to 9.3). This amplified difference highlights health-related vulnerabilities as a potentially important pathway into financial distress among victim-survivors, beyond that observed in financially distressed women without victim-survivor status. In the UK, it is estimated that disabled adults - defined as those living with a long-standing illness, condition, or impairment - are 2.6 times more likely to experience domestic abuse compared to non-disabled adults.

No evidence for VS-Control difference in optician spend was found (difference -0.5pp, 95% CI, -2 to 1), and estimated differences were smaller for dental (difference -3.4pp, 95% CI, -4.9 to -1.9 vs difference -8.8pp, 95% CI, -10.2 to -7.4 in the primary matched sample), and furniture spend (difference -4.5, 95% CI, -6 to -2.9 vs difference -8pp, 95% CI, -9.5 to -6.5 in the primary matched sample). Attenuated differences in self-care spend suggest that the experience of victim-survivors of financial abuse in these dimensions are not distinctive from women in financial distress without a victim-survivor status.

Interestingly, in contrast to results from the primary matched sample, no strong evidence of VS-Control differences in the distress-matched sample were found for petrol (difference 0.8pp, 95% CI, -0.5 to 2 vs difference 5pp, 95% CI, 3.8 to 6.3 in the primary matched sample) and taxi spend (difference 2.5pp, 95% CI, 0.9 to 4.1 vs difference 12.3pp, 95% CI, 10.8 to13.9 in the primary matched sample). This suggests that previously observed differences in these spending categories are not distinct from those seen in women who experience financial distress but not financial abuse. Such financial distress-related travel pressures may include irregular income, reduced flexibility, and time constraints.

Finally, the distress-matched results reveal distinctive bank interaction patterns for victim-survivors. The direction of the VS-Control difference was reversed for internet banking logins (difference -3.4, 95% CI, -5.3 to -1.5 vs difference 10.1, 95% CI, 8.3 to 11.9 in the primary matched sample). This suggests that, although increased internet banking logins are associated with financial distress, victim-survivors tend to rely more on human banking channels. This may reflect barriers to accessing internet banking, or the greater complexity and sensitivity of their needs, which are less easily addressed through digital channels."

Details on the construction of this sample are provided in the Methods (lines 614-633) "***Distress-matched and relationship-end matched samples. We applied the same filtering criteria as described above to create the distress-matched and relationship end date samples. Due to changes in the bank's data retention policy, at the time these analyses were conducted (January 2026), transaction histories used in the below analyses were restricted to 7 years. The analysis periods range between January 2019 and December 2024. Matching in the following analyses was performed using nearest-neighbor matching with a 3:1 treatment-to-control ratio, a caliper of 0.01, and without replacement.***
We created the distress-matched VS (N = 5,387) and control candidate (N = 76,083) samples by applying the same filtering criteria as described above. To create a matched sample, we used variables capturing financial distress and matched the two samples at the end of the analysis period (12 months prior to disclosure date), to create a control sample that experienced similar levels of financial distress as the VS sample in the period preceding disclosure. We used the following matching variables: year and month of the individual-specific analysis period, age, median monthly credit turnover, median monthly number of transactions, deprivation rank of the geography where the individual lived, average credit score, credit score 12 months prior to disclosure, average monthly number of rejected direct debits and overdraft charges, average loan, savings, and planned overdraft balances, binary flags for the following: savings, loan accounts, usage of planned overdraft, unpaid direct debit and overdraft charges.
The propensity matching process resulted in a final sample of 5,183 VS and 13,430 Control individuals. Supplementary Table 8 shows the output of the propensity score matching."

Taken together, this new analysis allows us to better understand which behaviours are specifically associated with the experience of financial abuse as opposed to financial distress. We thank the reviewer again for this suggestion, we believe that the additional analysis has strengthened the manuscript.

In addition, alternative temporal markers beyond the "disclosure date" should be explored to identify key stages in financial exploitation and to test their regularity.

Please see our new analysis of temporal markers and response to your earlier point on this, above.

Finally, the question of causality warrants deeper investigation. Qualitative research, particularly life-history interviews, would be indispensable for understanding the differentiated financial behaviors highlighted in the article and the motivations underlying them.

We thank the reviewer for this thoughtful suggestion. We agree that qualitative research, including life-history interviews, are invaluable for understanding the motivations and lived experiences underlying the financial behaviours highlighted in this study.

However, the present analysis is intentionally quantitative and based on administrative banking data, which necessarily limits inference about individual motivations and causal pathways. As we noted in our response to the earlier point relating to predictive indicators, in the revised version of the manuscript we clarify that our goal is to describe victim-survivors experiences of financial abuse using banking data to highlight that the observed differences are descriptive and associative in nature.

We now explicitly acknowledge the need for complementary qualitative research to explore these mechanisms in greater depth, and we view such work as an important direction for future research beyond the scope of the current study (discussion, lines 544-549):

"Third, our methodological approach does not allow us to establish causality between financial abuse and observed outcomes. For example, there may be two-way relationship between financial abuse and financial distress, or factors jointly driving abuse and distress. In addition, administrative data does not allow us to observe individual motivations or decision-making processes underlying these patterns; qualitative research, such as life-history interviews, would be valuable for exploring these mechanisms in greater depth."

At a minimum, the article suggests that financial distress may stem from two sources—first, the direct exploitation by an abusive partner, and second, the costly and complex processes involved in leaving the abusive situation. Distinguishing between these mechanisms is crucial for designing appropriate support measures.

We agree that distinguishing between financial distress arising from direct financial exploitation and distress associated with the processes of leaving an abusive relationship would be highly valuable for informing targeted support measures. However, as noted in our responses above and reflected in revisions to the manuscript, the present study is not able to establish causal mechanisms or to disentangle these pathways using administrative banking data alone.

We note, however, that in the relationship-end date analysis we observe broadly similar levels and patterns of financial distress in the period preceding relationship end and in the period preceding disclosure to the bank, which occurs after relationship end. This suggests that the observed financial distress is not confined to the post-separation phase or to disclosure-related processes alone.

While this does not permit identification of specific causal pathways, it supports the interpretation that financial distress is present across multiple stages of the abusive relationship and its dissolution.

G. References: Adequate Acknowledgment of Previous Work

The article could engage more extensively with existing qualitative research on financial abuse, including for instance the work of Sundari Anitha, E. Branigan, and S. Singh. Moreover, while the study focuses on the UK context, it would benefit from greater international perspective. Relevant research has been conducted in other countries, such as Australia and India, and should be cited to situate the contribution within the broader field.

Thank you for these suggestions. We have now included the work of Sundari Anitha and S. Singh, and added this important point to the limitations (lines 522-531):
"Our study has several limitations. First, the victim-survivor group, comprising of women in the UK with bank accounts who disclosed their victim-survivor status to the bank, and have met our sample selection criteria is not necessarily representative of the average victim-survivor, especially if disclosure is associated with experiencing significant financial distress. The findings reported here are most directly applicable to contexts with high levels of financial inclusion and individualized banking systems, such as the UK and may not generalize to international contexts, where financial abuse is shaped by different social and cultural norms[57], nor to settings where gendered and unpaid labor constitute central forms of economic control[58]. Further work is needed to understand the extent to which the results reported in this specific group are generalizable to other populations."

57. Singh, S. Domestic Economic Abuse: The Violence of Money. (Routledge, London, 2021). doi:10.4324/9781003178606.
58. Anitha, S. Understanding Economic Abuse Through an Intersectional Lens: Financial Abuse, Control, and Exploitation of Women's Productive and Reproductive Labor. Violence Women 25, 1854–1877 (2019).

H. Clarity and Context
The manuscript is well-written, and the abstract, introduction, and conclusion accurately reflect the content and objectives of the study. Overall, the paper is clearly structured and accessible, even when dealing with complex methodological issues.

Thank you.

Referee #5 (Remarks to the Author):

Using a matched case-control design, this study examined the evolution of a set of financial behavioral indicators (e.g. financial balances, spending patterns, account management) derived from banking data in the seven years prior to disclosure of financial abuse to bank personnel.

Compared to participants in the control group, victim-survivors had depleting savings and increasing debts over the seven years before reporting financial abuse to the bank, leading to missed payments and reduced credit scores. Participants in the victim-survivor group also showed relatively reduced expenditure on items related to self-care, transportation, and legal costs, increased cash withdrawals, more frequently changed addresses and passwords, and a higher likelihood of claiming welfare benefits. The authors conclude that results demonstrate how victim-survivors lose financial independence and stability. They also point out that banking data represents a fruitful new avenue for early identification of financial abuse.

I believe that the results presented in this manuscript will be of wide interest to an interdisciplinary group of scholars of intimate partner violence generally and economic abuse in particular. Exploring financial abuse through banking data is a creative and ambitious idea and the data set is unique in allowing for the investigation of survivors' evolving finances through objectively inferred data collected in a large sample. The authors controlled for socio-economic confounds, enabling a sharp comparison between the control and comparison group; and they were able to analyze a very large number of financial variables, creating a broad and deep picture of survivors' finances over time. As far as I know, this is the first quantitative and objective study of financial abuse's impact on survivors' everyday lives and represents an important leap in the study of this topic. The manuscript itself is well-written and the data is, for the most part, well presented.

Thank you for your kind words.

Nonetheless, the manuscript could be improved, and here I offer several suggestions for doing so, most notably related to the questions of causality and disclosure process. (Note that none of these suggestions touch on the statistical analyses used, as this is not my area of expertise.)

Abstract:

The abstract is clear and direct. However, as I will elaborate below, the study design does not allow for causal inferences and this should be made crystal clear upfront in the abstract.

We thank you for raising this issue, which was also raised by multiple reviewers and the editor in their letter. We agree that the language used in the previous version of the manuscript implied causality that was not supported by the data and methods used.

In response, we have clarified the goal of our analysis and removed suggestions of causal links that we cannot evidence.

Abstract:

- *"The ~~effects~~ **experience** of financial abuse, a form of domestic abuse involving control over money and finances, can be severe and long-lasting, yet difficult to document.*

Current evidence ***on how financial abuse shapes the everyday experiences*** *of ~~its impact on~~ victim-~~survivors' lives~~* ***survivors*** *is drawn primarily from small-scale, qualitative surveys."* Lines 30-33.

- *"Our results ~~show~~* ***demonstrate*** *how victim-survivors lose financial independence,* ***experience economic instability****, and ~~stability, and provide preliminary evidence that banking data could underpin a data-driven approach to mitigating foreseeable harm through early identification~~ suffer financial distress."* lines 43-44.

Introduction:

- *"However, the potential of financial data for understanding ~~the impact~~* ***victim-survivors experience*** *of financial abuse remains largely untapped."* lines 133-134.
- *"In addition, financial data allows us to follow the same individual over time, capturing ~~the evolving impact of economic abuse on~~* ***how financial abuse is associated with changes in*** *victim-survivors' financial lives."* Lines 146-148.
- *"The matched-design approach ~~controlled~~* ***controls*** *for socio-economic confounds, ~~allowing us to characterize the impact~~* ***, enabling the comparison*** *of ~~financial abuse on~~ victim-survivors' financial wellbeing* ***with controls*** *over time. The outcomes analyzed here span a wide range of behaviors, including financial balances, spending patterns and account management~~,~~****. To our knowledge, this is*** *~~providing~~ the first empirical illustration of* ***the experience of*** *financial ~~abuse's impact~~* ***abuse*** *~~on~~* ***in*** *victim-survivors' everyday lives* ***using banking data****.* Lines 166-172

Results:

- *"To ~~identify~~* ***define*** *the subset of 373 indicators ~~characterizing the effects of~~* ***reflecting differences associated with*** *financial abuse, we first identified"* lines 175-177
- *"~~Decreased spend~~* ***Decreased spending*** *on self beyond basic necessities ~~may reflect the impact of~~* ***aligns with patterns of*** *financial control ~~– either through restriction tactics or exploitation leaving victim-survivors with less money via increased borrowing~~* ***documented in prior studies, including restricted access to funds and financial exploitation, both of which have been linked to reduced financial autonomy among victim-survivors"*** lines 273-276
- *"Higher spending in off-licence stores is consistent with prior evidence showing that victim-survivors may use alcohol as a coping strategy~~, which may even be encouraged by the abuser to increase control~~"*-lines 2812-284
- *"The pattern of increased cash usage ~~might reflect~~* ***is consistent with a range of experiences reported in the literature, including*** *financial exploitation through unauthorized or forced cash withdrawals aiming to deplete the victim-survivor's resources"* lines 307-310
- *"Lower commuting-related spending in the VS group ~~is in line with earlier results showing lower likelihood of employment and could result from sabotaging employment, while increased petrol spend may signal the victim-survivor's attempts to maintain safety and access support which may involve longer journeys. Access to~~*

~~*taxis can provide a quick and discreet emergency escape option particularly when shared vehicles are being tracked by the abuser, and an analysis of escape journeys of women found that taxis were the second most common option after transport provided by friends and family.*~~ ***aligns with earlier findings of lower employment prevalence among victim-survivors. Higher spending on flexible transport options, including cars and taxis, has been reported previously and may reflect the complex, constrained circumstances in which victim-survivors meet everyday needs, including limited alternatives, time pressures and safety considerations."*** lines 319-323

- *"All of these activities* ~~*may be linked to the abuser taking control of bank accounts or victim-survivors seeking to take back control*~~ ***reflect changes in account access and management consistent with those reported in situations involving financial abuse, including both shifts in control by an abuser and increased engagement by victim-survivors*** *post-separation, when it may be safe to do so. Frequent* ~~*address changes are likely to reflect separation and the victim-survivors' efforts to prevent the abuser from discovering their new location*~~ ***changes of address align with the residential instability often associated with separation"*** lines 333-337

Discussion

- *"To our knowledge,* ~~*the present study*~~ ***this*** *is the first* ~~*to draw on*~~ ***study using*** *administrative, objective data* ~~*sources*~~ *to* ~~*document the impact of*~~ ***describe how*** *financial abuse* ~~*on*~~ ***manifests in*** *victim-survivors' lives"* Lines 458-459
- *"**Observed** changes in cash usage, transport spend**ing**, and account management activity* ~~*may reflect victim-survivors' efforts to escape their abuser*~~ ***are consistent with patterns reported around periods of safety-seeking or separation among victim-survivors.****"* Lines 500-502
- *"*~~*The results presented here, while descriptive, offer preliminary evidence for the feasibility of developing risk indicators that could underpin proactive support strategies and minimize the risk of foreseeable harm.*~~*"*
- We also added the following limitation: "***our methodological approach does not allow us to establish causality between financial abuse and observed outcomes. For example, there may be two-way relationship between financial abuse and financial distress, or factors jointly driving abuse and distress.***" lines 544-546
- *"Despite these limitations, this study shows that financial data provides a valuable lens to understand the* ~~*impact*~~ ***experience*** *of financial abuse. The results presented in this study indicate that banking data* ~~*has the potential to underpin the*~~ ***data could guide future efforts to*** *develop*~~*ment of risk*~~ ***vulnerability*** *indicators and intervention strategies* ***designed*** *to* ~~*prevent harm and*~~ *support victim-survivors."* lines 550-554

Introduction/Methods:

The authors provide appropriate citations throughout this section but I would suggest adding some of the key references in this area, including the following:

Stylianou, A. M. (2018). Economic abuse within intimate partner violence: A review of the literature.
Violence & Victims, 33(6), 983–1001.

Johnson, L., Chen, Y., Stylianou, A., & Arnold, A. (2022). Examining the impact of economic abuse on survivors of intimate partner violence: A scoping review. BMC Public Health, 22, 1262.
Postmus, J. L., Hoge, G. L., Breckenridge, J., Sharp-Jeffs, N., & Chung, D. (2020). Economic abuse as an invisible form of domestic violence: A multicountry review. Trauma, Violence, & Abuse, 21(2), 261–283.

Adams AE, Littwin AK, Javorka M. (2020). The frequency, nature, and effects of coerced debta among a national sample of women seeking help for intimate partner violence. Violence Against Women, 26(11):1324-1342.

Line 38: "Financial abuse is a subcategory of economic abuse…." It would be useful to define and elaborate a bit on economic abuse as well as on how economic abuse often fits within a broader pattern of coercive control. This would allow the reader to understand financial abuse in a more comprehensive way.

Thank you for these constructive suggestions. We have added these references to the revised version of the paper, and we now position financial abuse within the wider context of coercive control. This was a valuable suggestion, as it highlights the interconnected nature of different forms of domestic abuse and the ways in which economic and financial control are embedded within broader patterns of coercion that shape multiple aspects of victim-survivors' lives.

Introduction:

- ***"While research on domestic abuse has mostly focused on physical violence[12], many scholars stress that violence must be understood within the wider context of coercive control[13,14], which can also encompass psychological, sexual, economic and financial abuse. The terms 'economic' and 'financial' abuse are often used interchangeably in the literature, but while economic abuse is broadly defined as control over economic resources, financial abuse specifically focuses on control over money and finances[15]."*** Lines 101-106
- ***"For the victim, loss of control over their financial life often co-occurs with difficulties across non-financial domains of life, including mental and physical health, the ability to pay for personal goods, and participation in leisure and well-being activities[19,20]."*** Lines 117-120
- ***"Previous studies based on interviews and small-scale surveys reveal that even years after being subjected to abuse, victim-survivors often remain burdened by substantial debt, damaged credit scores[1], and the less visible loss of financial skills and confidence[2], all of which hinder regaining financial independence and stability[3]."*** Lines 121-124

1. Adams, A. E., Littwin, A. K. & Javorka, M. The Frequency, Nature, and Effects of Coerced Debt Among a National Sample of Women Seeking Help for Intimate Partner Violence. Violence Women ***26****, 1324–1342 (2020).*
12. Postmus, J. L., Hoge, G. L., Breckenridge, J., Sharp-Jeffs, N. & Chung, D. Economic Abuse as an Invisible Form of Domestic Violence: A Multicountry Review. Trauma Violence Abuse ***21****, 261–283 (2020).*
19. Johnson, L., Chen, Y., Stylianou, A. & Arnold, A. Examining the impact of economic abuse on survivors of intimate partner violence: a scoping review. BMC Public Health ***22****, 1014 (2022).*
20. Stylianou, A. M. Economic Abuse Within Intimate Partner Violence: A Review of the Literature. Violence Vict. ***33****, 3–22 (2018).*

Line 80: Here, the authors introduce the idea of survivor disclosures. Although they describe the disclosure process in the Methods section, without a fuller description here, I was confused as I continued to read.

Also, in this first description of disclosure, it would be useful to make explicit that disclosure recipients did not document length of time since the start of the abuse. This should also be named in the Limitations section.

We thank you for raising this important point which was highlighted by multiple reviewers. In the revised version of the manuscript, we provide a more detailed description of how victim-survivors come to disclose their experiences to the bank in the introduction (lines 152-161): *"****Victim-survivors self-disclosed their experiences to the bank's specialist financial and domestic abuse team (VS group; N = 5,428). Bank staff are trained to identify potential vulnerabilities, including financial and domestic abuse, during routine interactions (e.g., querying transactions, clarifying charges). When staff judge that a customer may have experienced domestic or financial abuse and might benefit from further support, they offer a referral to the bank's specialist team. With consent from the customer, the specially trained team contacts the customer to understand more about the nature and experience of abuse and, where possible, provide personalized support. The most common forms of support provided are separation of joint accounts, temporary suppression of charges, and signposting to relevant charities.****"*

In the revised version, we also acknowledge the limitation around information on the length of abuse (lines 537-543):

"*(..)Due to data availability restrictions, our analysis only looks at a maximum 7-year window* ***and draws on data from one bank.*** *Since domestic abuse can span decades****, impacting all bank accounts held by the individual,*** *this timeframe* ***and data view*** *may ~~not fully capture the long-term financial markers of~~* ***reflect all of an individual's financial patterns associated with financial abuse. In addition, disclosure records do not include information on the duration of abuse prior to disclosure, which limits our ability to relate observed financial behaviors to the duration or timing of abuse."***

Whilst we do not have information on the start of the abuse, we have been able to re-interrogate the data and replicate the main results for a sample of victim-survivors where we were able to extract a temporal marker for when the relationship ended (lines 359-366):

"Second, we created a smaller relationship-end matched sample that included victim-survivors where information on relationship end date was available (N = 1,149) and corresponding matched controls (N = 3,236; 21% of the primary matched control sample). By comparing VS-Control differences at relationship end, rather than disclosure date, we can examine the extent to which the patterns reported are specific to the pre-separation period. Aside from defining the individual analysis period using the relationship end date, we followed the same matching procedure as in the primary sample. Supplementary Table 2 presents descriptive statistics for the VS and Control groups."

Lines 634-654 in the Methods explain our approach in more detail:

"To create the relationship-end date samples, we extracted relationship-end date information from the VS candidate sample. Applying the same criteria as described above, we retrieved notes associated with 5,303 victim-survivors. During appointments, with the customer's consent, bank staff records key details of each case in the form of notes. The richness of the information recorded vary by note, but some includes temporal markers, most commonly the relationship end date. We identified 3,559 notes that contained words indicative of a relationship end date. These records either included month names (e.g., "jan") or any the following search words: fled, split, ended, ago, left. We then manually reviewed these notes and extracted relationship end dates with month-level precision. For example, "relationship ended 3 years ago" was not accurate enough, but "fled 2 weeks ago" would be included with a relationship end date two weeks prior to disclosure. We retrieved the latest known contact date with the abuser at the time of the disclosure, and only if it was within our analysis period (after January 2019). Therefore, relationship end could be missing if it was not mentioned in the note, was too long ago, was not specific enough or if the victim-survivor was still in the relationship at the time of disclosure. The manual review resulted in a sample with 1,189 VS sample candidates. On average, relationships ended 7.7 months (s.d. 9.3) prior to disclosure date.
The matching variables were the same as those used in the primary matched sample, the only difference being that the individual-specific analysis period was now defined by the relationship-end date as opposed to disclosure date. The propensity matching process resulted in a final sample of 1,149 VS and 3,236 Control individuals. Supplementary Table 9 shows the output of the propensity score matching"

Using this sample, we replicated the main analysis with results shown in Supplementary Figure 3. We report the following in the Results (lines 401-416):

"Results from the relationship-end matched sample (Supplementary Figure 3, Supplementary Table 6) were consistent with those observed in the primary matched sample, although estimated VS-Control differences were generally smaller and several estimates were centered near zero, reflecting reduced statistical power given the smaller sample size.

Specifically, in the relationship-end matched sample, no evidence for VS-Control difference was found for payroll (difference -1.1pp, 95% CI, -2.6 to 0.5 vs difference -1.8pp, 95% CI, -2.6 to -1.1 in the primary matched sample), optician (difference -2.3pp, 95% CI, -5.3 to 0.7 vs difference -2.9pp, 95% CI, -4.3 to -1.5 in the primary matched sample), and sport (difference 1.3pp, 95% CI, -2 to 4.7 vs difference -5.3pp, 95% CI, -6.9 to -3.8 in the primary matched sample) spend.

Observed VS-Control differences were larger for cash withdrawal (difference 7pp, 95% CI, 5.4 to 8.5 vs difference 4pp, 95% CI, 3.3 to 4.7 in the primary matched sample), and petrol spend (difference 10.1pp, 95% CI, 7.4 to 12.7 vs difference 5pp, 95% CI, 3.8 to 6.3 in the primary matched sample). The larger differences in cash withdrawals and petrol spending around relationship end compared to disclosure date may reflect practical demands associated with separation, including relocation or increased travel."

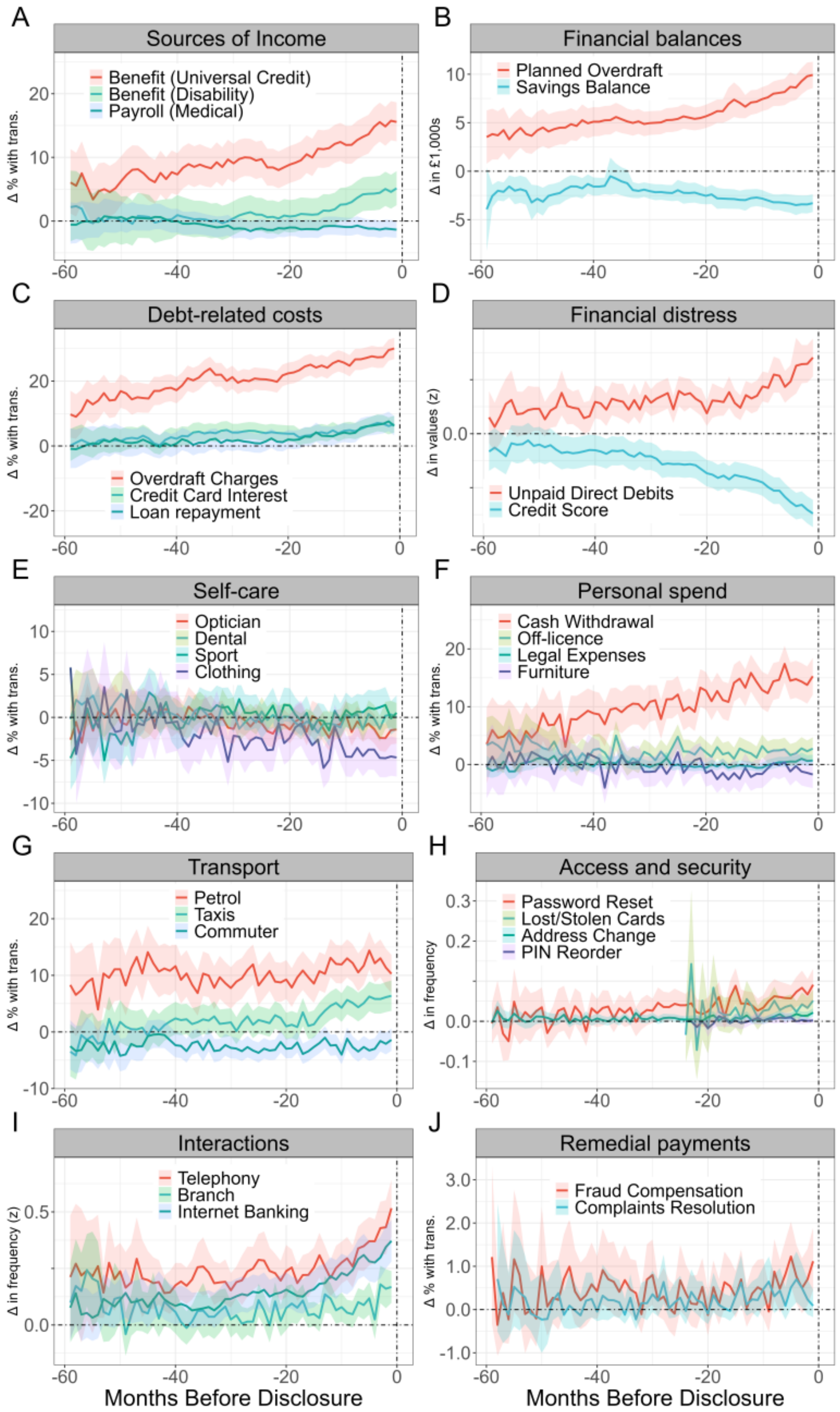

Supplementary Figure 3. Monthly VS-Control Group differences in selected financial indicators (relationship-end matched sample). Line plots showing monthly VS-Control group differences (vertical axis). The horizontal axis shows the number of months relative to relationship end date

(month = 0). Shading denotes 95% CI. Sample sizes: VS (N =1,149), Control (N =3,236). Data on Lost/Stolen cards and PIN reorder are only available from January, 2023. **Panel A. Sources of Income**. VS-Control difference in monthly group proportion with transactions. Exact (transactional) variables used: Benefits/Disability Living Allowance/Other, Benefits/Universal Credit/Other, Payroll/Medical/NHS. **Panel B. Financial balances**. VS-Control difference in monthly group averages (measured in thousand pounds). Exact (non-transactional) variables used: Planned Overdraft, Savings Balance. **Panel C. Debt-related costs.** VS-Control difference in monthly group proportion with transactions. Exact variables used: Credit Card Interest (derived transactional), Financial Services/Loans/Other (transactional), Overdraft charges (derived transactional). **Panel D. Financial distress.** VS-Control difference in scaled monthly group averages (measured in monthly credit scores, and monthly counts of missed direct debits). Exact variables used: Credit score (non-transactional), Unpaid direct debit (derived transactional). **Panel E. Self-care.** VS-Control difference in monthly group proportion with transactions. Exact (transactional) variables used: Retail/Clothing/Women, Services/Health/Dental, Services/Health/Optician, Hobbies & Interests/Sport/Other. **Panel F. Personal spend.** VS-Control difference in monthly group proportion with transactions. Exact variables used: ATM Withdrawal (derived transactional), Retail/Furniture/Other (transactional), Services/Professional/Legal (transactional), Retail/Food & Drink/Alcohol (transactional). **Panel G. Transport.** VS-Control difference in monthly group proportion with transactions. Exact (transactional) variables used: Travel/Commuter/Other, Motor/Petrol/Other, Travel/Taxis/Other. **Panel H. Access and security.** VS-Control difference in monthly frequency. Exact (non-transactional) variables used: Address Change, Lost/Stolen card, Password Reset, PIN Reorder. **Panel I. Interactions.** VS-Control difference in monthly scaled frequency. Exact (non-transactional) variables used: Branch visits, Internet Banking logins, Telephone visits. **Panel J.** Remedial payments. VS-Control difference in monthly group proportion with transactions. Exact (transactional) variables used: Financial Services/Fraud/Write Off, Financial Services/Other/Complaints Resolution.

Therefore, we conclude that despite the difference in sample sizes, the vast majority of VS-Control differences reported in the main analysis (primary matched sample) are still present when we use relationship end date as a temporal marker instead of disclosure date.

In the Methods section, Line 284, the authors provide more detailed information on the disclosure process, but this information, in turn, raised concerns that overlap with the question of causality, described next: In my reading, it sounds like participants disclosed the financial abuse in the hopes of receiving some sort of support. How might a potential reward for disclosure affect who is in the victim-survivor group? At the margin, does financial insecurity raise the likelihood of reporting abuse to the bank? Is it possible, for example, that only those with the most severe financial abuse reported it? (Conversely, it is possible that those with higher abuse were more afraid to disclose the abuse). These interpretations should be discussed more deeply in the Limitations section.

We thank the reviewer for raising these important points. While we agree that financial distress may influence the likelihood of disclosure and, therefore, inclusion in the victim-survivor sample, disclosure typically occurs through routine customer-bank interactions (for example, queries about transactions), rather than being driven by the expectation of a specific form of support. Moreover, accessing payment flexibility (such as temporary suppression of charges) is only one of several reasons a customer may choose to disclose their vulnerability. Other, common motivations[8] include reducing the need to repeatedly recount distressing experiences to different staff, enabling additional monitoring of account activity (for example, checks on unusually large transactions), and opting out of standard banking processes that assume full financial control and may otherwise lead to unintended harm (such as automated correspondence).

We have undertaken further analysis to examine whether the VS sample contains only individuals experiencing severe financial consequences. To this end, we compared the average credit scores of the VS group during the year prior to disclosure against a control group of 18,906 randomly selected female customers (Female bank population benchmark) from the same period. Details on the construction of this sample can be found in the Methods section (lines 770-774), while Supplementary Figure 7 shows the comparison.

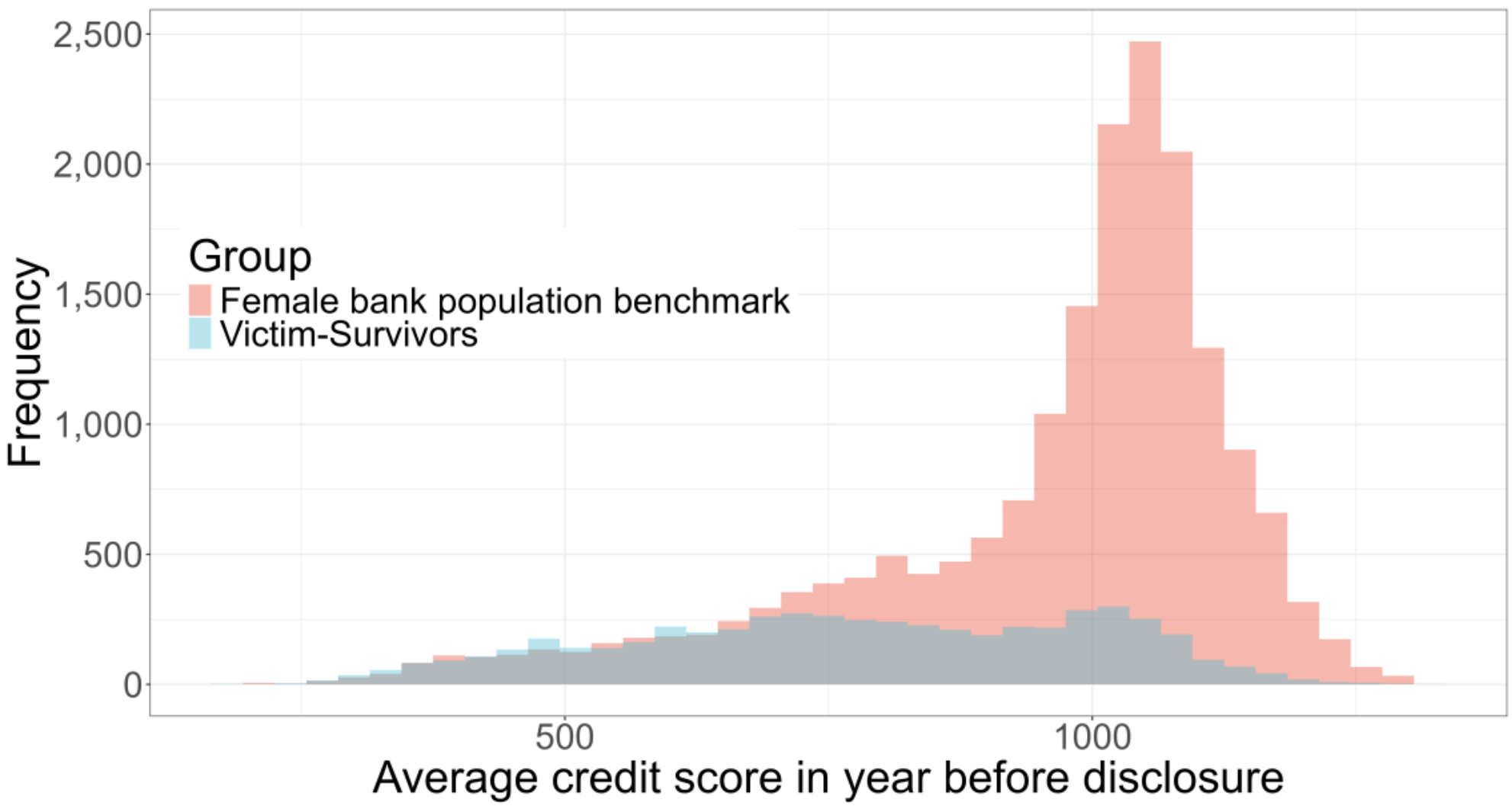

Supplementary Figure 7. Victim-survivor and Female bank population credit score distributions in the year prior to disclosure. The figure shows that while on average, credit scores are significantly lower in the VS group (as expected), we see a wide distribution spanning the entire interval of Control credit score values, suggesting that that the VS sample does not only include individuals in severe financial distress. For methodological details, see "Measuring the degree of self-selection by financial distress".

This reveals two patterns. First, as expected, the average credit score among victim-survivors is lower than that among the Female bank population benchmark group. Second, the plot illustrates that the victim-survivor group does not only contain individuals with severely low credit scores, but instead contains individuals with credit scores across the distribution of the Female bank population benchmark group. Overall, 35% of individuals in the victim-survivor group have credit scores that sit above the 25th percentile of credit score (887) for the Female bank population benchmark group. We agree with the reviewer comment that one might be concerned in particular if the victim-survivor group were heaped at very low credit scores and/or the plot illustrated a clear bi-modality between the two groups. We are reassured by the overlapping distributions that, while the credit score for victim-survivors is lower than that for the Female bank population benchmark group, our victim-survivor sample is not only limited to individuals experiencing severe financial situations.

We acknowledge this in the limitations (lines 522-525):

"First, the victim-survivor group, comprising of women in the UK with bank accounts who disclosed their victim-survivor status to the bank, and have met our sample selection criteria is not necessarily representative of the average victim-survivor, especially if disclosure is associated with experiencing significant financial distress. "

As part of the revision, in addition to the VS sample description in the Introduction, we also make it clearer that we rely on disclosures:

"Our analysis focuses on self-disclosing victim-survivors with ongoing banking relationships, allowing us to track outcomes over time." Lines 164-165

"Given estimated levels of underreporting of financial abuse ***and our reliance on self-disclosure****, the control group may also include victim-survivors who did not disclose."* Lines 532-533

Line 83: This is the first place the authors discuss their use of a retrospective design to examine the "impact of financial abuse on victim-survivors' financial wellbeing over time." As I began to note in the above comment about the disclosure process, here and elsewhere, the authors imply a causality they cannot establish empirically. Their retrospective design allows for various interpretations of the findings. For example, bad financial luck or poor financial decision-making can set in motion a woman's entry into a relationship where she has relatively little power simply out of financial desperation, setting the stage for abuse. In this interpretation, it is the financial downturn that predicts the abuse, not the other way around. In other words, the authors sometimes make leaps in interpreting their findings and should offer clear alternative explanations, probably in the Limitations section.

Thank you for this comment, which was emphasized by multiple reviewers and the editor in their letter. The primary goal of the analysis is to examine how financial abuse is reflected in the experiences of victim-survivors, using banking data. The matched control sample approach was constructed to achieve this goal. This approach provides a statistical counterfactual to the experience of victim-survivors over time, and we agree it is not appropriate for inferring casual relationships.

Please see our response to your first point, in which we provide a summary of revisions aimed at clarifying the goal of the analysis and removing causal language throughout the manuscript.

Figures:

Figure 1: I like these panels as a way to visualize complex data. It might be useful to add labels or units along the vertical axis for easier comprehension

Thank you for raising this point. We have now added y axis labels to Figure 1 to clarify measurement units and support the interpretation of the patterns depicted.

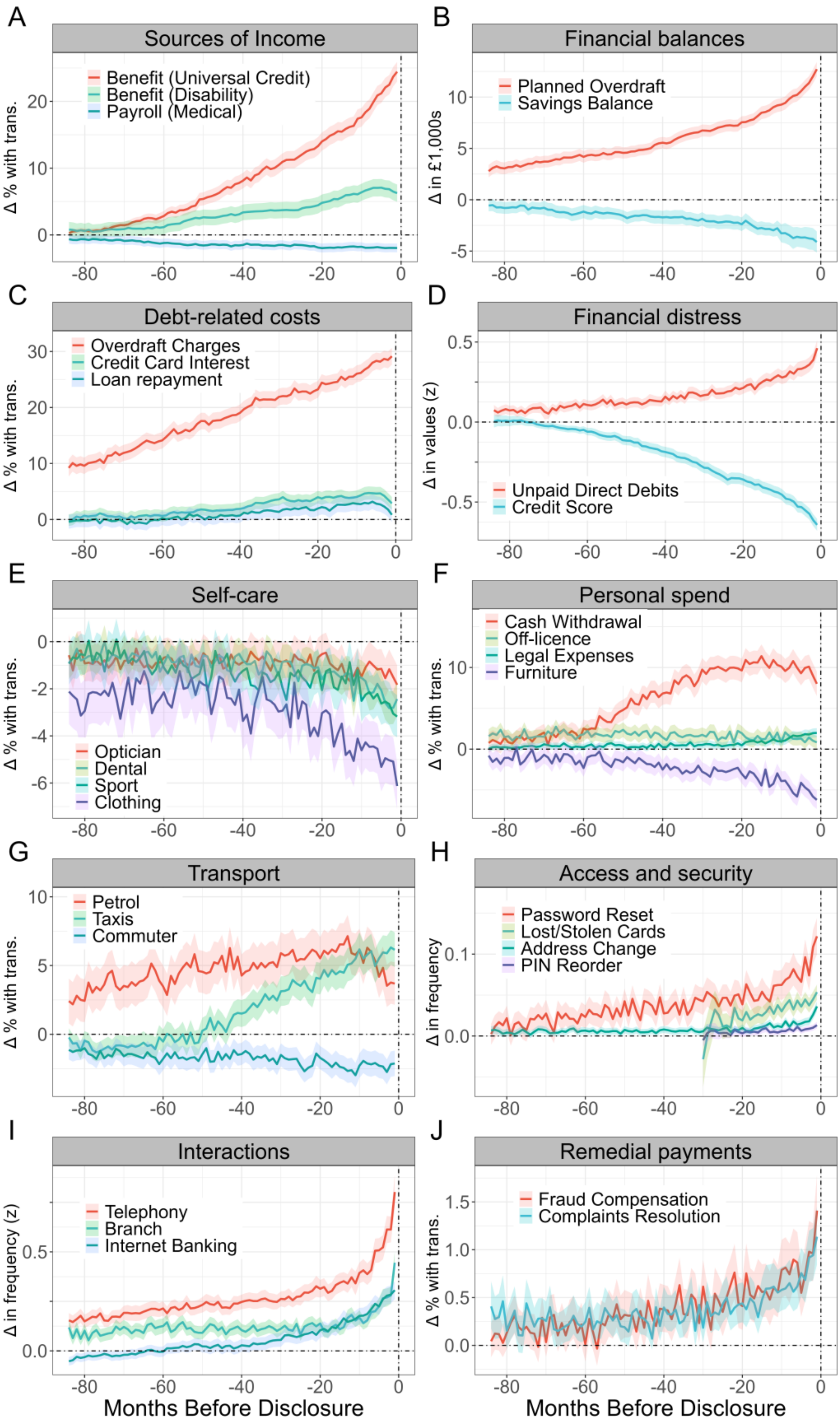

A
Sources of Income
Benefit (Universal Credit)
Benefit (Disability)
Payroll (Medical)
Δ % with trans.
B
Financial balances
Planned Overdraft
Savings Balance
Δ in £1,000s
C
Debt-related costs
Overdraft Charges
Credit Card Interest
Loan repayment
Δ % with trans.
D
Financial distress
Unpaid Direct Debits
Credit Score
Δ in values (z)
E
Self-care
Optician
Dental
Sport
Clothing
Δ % with trans.
F
Personal spend
Cash Withdrawal
Off-licence
Legal Expenses
Furniture
Δ % with trans.
G
Transport
Petrol
Taxis
Commuter
Δ % with trans.
H
Access and security
Password Reset
Lost/Stolen Cards
Address Change
PIN Reorder
Δ in frequency
I
Interactions
Telephony
Branch
Internet Banking
Δ in frequency (z)
J
Remedial payments
Fraud Compensation
Complaints Resolution
Δ % with trans.
Months Before Disclosure
Months Before Disclosure

Figure 1. Monthly VS-Control Group differences in selected financial indicators (primary matched sample). Line plots showing monthly VS-Control group differences (vertical axis). The horizontal axis shows the number of months relative to disclosure date (month = 0). Shading denotes 95% CI. Sample sizes: VS (N =5,428), Control (N =15,602). Data on Lost/Stolen cards and PIN reorder are only available from June, 2022. **Panel A. Sources of Income**. VS-Control difference in monthly group proportion with transactions. Exact (transactional) variables used: Benefits/Disability Living Allowance/Other, Benefits/Universal Credit/Other, Payroll/Medical/NHS. **Panel B. Financial balances**. VS-Control difference in monthly group averages (measured in thousand pounds). Exact (non-transactional) variables used: Planned Overdraft, Savings Balance. **Panel C. Debt-related costs.** VS-Control difference in monthly group proportion with transactions. Exact variables used: Credit Card Interest (derived transactional), Financial Services/Loans/Other (transactional), Overdraft charges (derived transactional). **Panel D. Financial distress.** VS-Control difference in scaled monthly group averages (measured in monthly credit scores, and monthly counts of missed direct debits). Exact variables used: Credit score (non-transactional), Unpaid direct debit (derived transactional). **Panel E. Self-care.** VS-Control difference in monthly group proportion with transactions. Exact (transactional) variables used: Retail/Clothing/Women, Services/Health/Dental, Services/Health/Optician, Hobbies & Interests/Sport/Other. **Panel F. Personal spend.** VS-Control difference in monthly group proportion with transactions. Exact variables used: ATM Withdrawal (derived transactional), Retail/Furniture/Other (transactional), Services/Professional/Legal (transactional), Retail/Food & Drink/Alcohol (transactional). **Panel G. Transport.** VS-Control difference in monthly group proportion with transactions. Exact (transactional) variables used: Travel/Commuter/Other, Motor/Petrol/Other, Travel/Taxis/Other. **Panel H. Access and security.** VS-Control difference in monthly frequency. Exact (non-transactional) variables used: Address Change, Lost/Stolen card, Password Reset, PIN Reorder. **Panel I. Interactions.** VS-Control difference in monthly scaled frequency. Exact (non-transactional) variables used: Branch visits, Internet Banking logins, Telephone visits. **Panel J.** Remedial payments. VS-Control difference in monthly group proportion with transactions. Exact (transactional) variables used: Financial Services/Fraud/Write Off, Financial Services/Other/Complaints Resolution.

Figure 3: It would be easier to compare Panels A and B if the categories along the vertical axis were in the same order.

We thank the reviewer for this suggestion and agree that consistent ordering can facilitate direct visual comparison across panels. However, in this case, Panels A and B are intentionally ordered to reflect the ranking of variables within each perspective (onset and size of differences), highlighting how the ranking of different behaviours differs between the two views. We felt it was important to retain the panel-specific ordering to illustrate differences in relative ranking across perspectives rather than impose a single ordering, which would make it difficult for the reader to infer ranking for (at least) one of the categories.

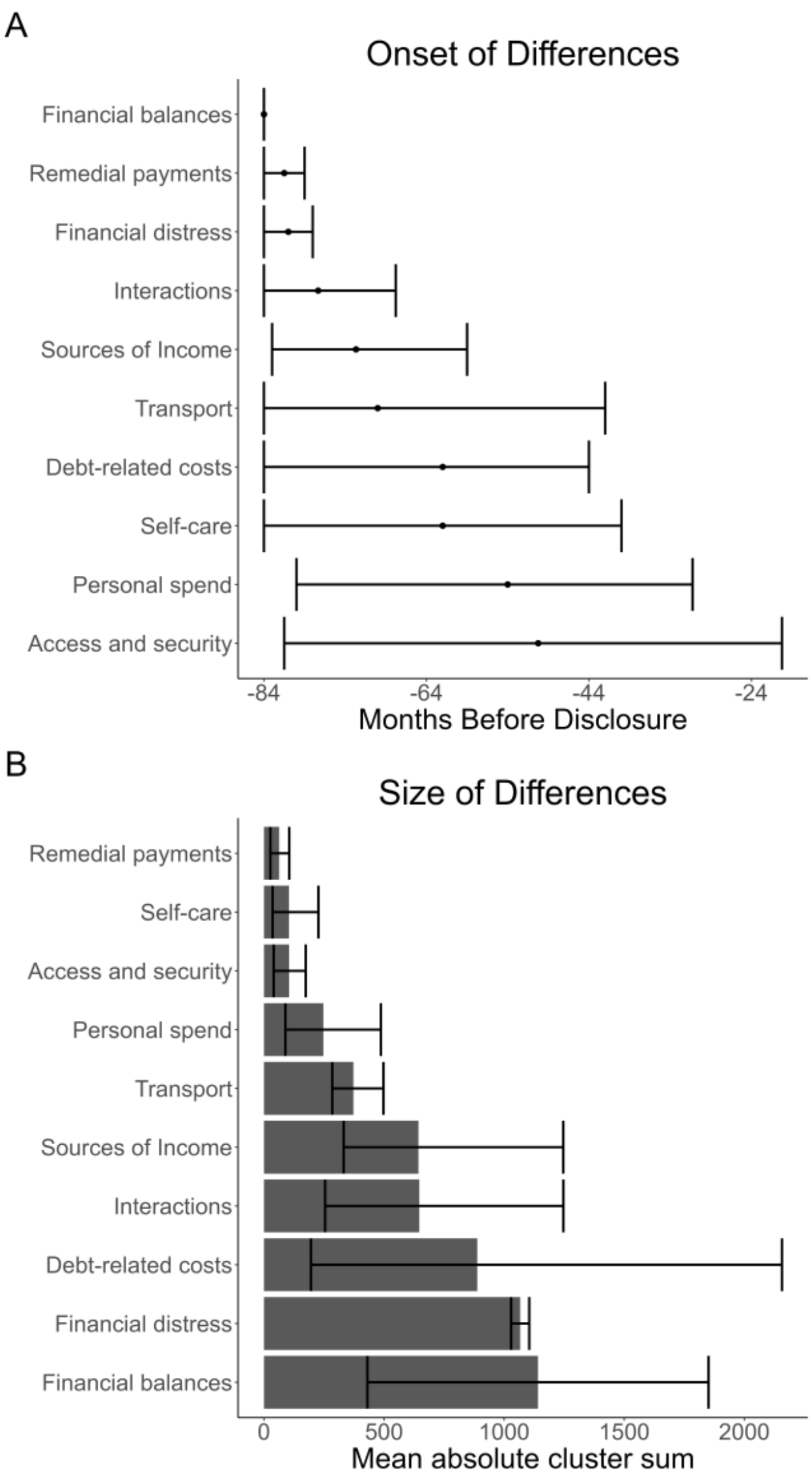

Figure 3. Aggregated temporal clustering results for selected variables (primary matched sample). Point and bar plots showing clustering results aggregated by financial indicator group shown on Figure 1 (N=30 indicators used). **Panel A** shows the average earliest significant cluster start point by financial indicator group and the associated 95% CIs. **Panel B** shows the average cluster sum by financial indicator group and the associated 95% CIs.

Discussion:

The limitations section lays out well some of the challenges in interpreting the results of the study. I think these should be elaborated – particularly the two I have focused on in this review - so that the authors have a chance to explore alternative interpretations of the data more fully.

Thank you for this feedback. Regarding the use of causal language, please refer to our response to your first point, where we detail revisions aimed at clarifying the study's objectives and removing causal terminology throughout the manuscript. Regarding the disclosure process, please see our response to your third point, where we provide more details on the construction of the victim-survivor sample and the possible self-selection by financial distress.

Referee #5 (Remarks on code availability):

References

1. Disability and crime, UK - Office for National Statistics. https://www.ons.gov.uk/peoplepopulationandcommunity/healthandsocialcare/disability/bulletins/disabilityandcrimeuk/2019.
2. Urban Transport | She Drives Change. https://shedriveschange.worldbank.org/urban-transport?utm_source=chatgpt.com.
3. Population estimates for the UK, England, Wales, Scotland and Northern Ireland - Office for National Statistics. https://www.ons.gov.uk/peoplepopulationandcommunity/populationandmigration/populationestimates/bulletins/annualmidyearpopulationestimates/mid2024.
4. Home - Office for National Statistics. https://www.ons.gov.uk/.
5. Ellie Butt. *Know Economic Abuse*. https://refuge.org.uk/wp-content/uploads/2020/10/Know-Economic-Abuse-Report-2020.pdf (2020).
6. Formatting guide | Nature. https://www.nature.com/nature/for-authors/formatting-guide.
7. Surviving Economic Abuse. *TEXAS Framework: For Victim-Survivors of Economic Abuse*. https://survivingeconomicabuse.org/wp-content/uploads/2021/02/TEXAS-one-page-FINAL.pdf (2021).
8. How banks can help - Surviving Economic Abuse. https://survivingeconomicabuse.org/i-need-help/banking/how-banks-can-help/.
9. Financial Conduct Authority. *FG21/1: Guidance for Firms on the Fair Treatment of Vulnerable Customers*. https://www.fca.org.uk/publication/finalised-guidance/fg21-1.pdf.
10. Families and households in the UK - Office for National Statistics. https://www.ons.gov.uk/peoplepopulationandcommunity/birthsdeathsandmarriages/families/bulletins/familiesandhouseholds/2024.

11. Transactional banking. *UK Finance* https://www.ukfinance.org.uk/our-expertise/personal-banking/transactional-banking.
12. Surviving Economic Abuse. *Annex 1: Prevalence of Economic Abuse Globally*. https://survivingeconomicabuse.org/wp-content/uploads/2022/11/SEA_Economic-Abuse-A-Global-Perspective_Annex.pdf.
13. Domestic abuse in England and Wales overview - Office for National Statistics. https://www.ons.gov.uk/peoplepopulationandcommunity/crimeandjustice/bulletins/domesticabuseinenglandandwalesoverview/november2025.
14. Domestic abuse is a gendered crime. *Women's Aid* https://womensaid.org.uk/information-support/what-is-domestic-abuse/domestic-abuse-is-a-gendered-crime/.
15. UK customers more likely to switch banks than European peers. https://www.thebanker.com/content/7227b62d-0fa2-548d-9ae5-1e25f70a2230.
16. Singh, S. *Domestic Economic Abuse: The Violence of Money*. (Routledge, London, 2021). doi:10.4324/9781003178606.
17. Anitha, S. Understanding Economic Abuse Through an Intersectional Lens: Financial Abuse, Control, and Exploitation of Women's Productive and Reproductive Labor. *Violence Women* **25**, 1854–1877 (2019).
18. Postmus, J. L., Hoge, G. L., Breckenridge, J., Sharp-Jeffs, N. & Chung, D. Economic Abuse as an Invisible Form of Domestic Violence: A Multicountry Review. *Trauma Violence Abuse* **21**, 261–283 (2020).
19. Stark, E. *Coercive Control: How Men Entrap Women in Personal Life*. (Oxford University Press, 2023). doi:10.1093/oso/9780197639986.001.0001.
20. Domestic Abuse Intervention Programs. The Duluth Model. https://www.theduluthmodel.org/wheels/ (2017).

21. Sharp-Jeffs, N. *Money Matters : Research into the Extent and Nature of Financial Abuse within Intimate Relationships in the UK*. https://repository.londonmet.ac.uk/1481/ (2015).

22. Johnson, L., Chen, Y., Stylianou, A. & Arnold, A. Examining the impact of economic abuse on survivors of intimate partner violence: a scoping review. *BMC Public Health* **22**, 1014 (2022).

23. Stylianou, A. M. Economic Abuse Within Intimate Partner Violence: A Review of the Literature. *Violence Vict.* **33**, 3–22 (2018).

24. Adams, A. E., Littwin, A. K. & Javorka, M. The Frequency, Nature, and Effects of Coerced Debt Among a National Sample of Women Seeking Help for Intimate Partner Violence. *Violence Women* **26**, 1324–1342 (2020).

25. New data shows victim-survivors of economic abuse are left with long-lasting harm. *Surviving Economic Abuse* https://survivingeconomicabuse.org/news/new-data-shows-victim-survivors-of-economic-abuse-are-left-with-long-lasting-harm/ (2022).

Revision of Nature manuscript 2025-09-23824

"Victim-survivors' banking records reveal the characteristics of financial abuse"

Thank you for offering us the opportunity to undertake a further revision of this paper for publication in Nature. Please see the decision letter and reviewer comments on the first revision enclosed below in italics, with our responses shown in plain type. Please also find enclosed our revised manuscript with tracked changes and line numbers added, which we reference below.

Editor Letter

Dear Dr Trendl,

Your manuscript, "Victim-survivors' banking records reveal the characteristics of financial abuse", has now been seen by 5 referees. You will see from their comments below that while they find your work of interest, some important points are raised. We are interested in the possibility of publishing your study in Nature, but would like to consider your response to these concerns in the form of a revised manuscript before we make a final decision on publication.

In addition to addressing the remaining reviewer requests and comments, we would like you to revise your manuscript taking into account the following points:

-Ethics. The bank has granted permission to use the data, but the details about this process are unclear. Please add more information regarding the bank's ethical committee and approval process, including if the consent requested included publication of the findings beyond usage for the study.

This research was approved by the bank's internal data privacy and risk assessment body, which reviews proposed uses of customer data for compliance with applicable data protection, privacy, and operational risk requirements. The research was conducted in the context of the bank's customer vulnerability and support initiatives. As part of this review, the publication of aggregated research findings beyond internal analytical use was specifically assessed and approved. The processing of customer data for this research was conducted under the bank's legitimate interests basis for processing with the aim to improve customer support. Customers were informed through the bank's privacy notice at account opening that their personal data may be used for behavioural segmentation to better understand customer needs, and that aggregated research findings and insights may be shared beyond the bank.

We have added more information regarding the bank's ethical committee and approval process. Please see the revised text at lines 451-460 in Methods:

"Prior to analysis commencing, approval was obtained following review by the bank's Privacy Risk and Impact Assessment (PRIA) governance body, which is independent of the project team. The review was conducted by a panel of specialists in data ethics, privacy, impact assessment, and UK GDPR compliance, including data protection officers. The PRIA application for this project was submitted in March 2024. Approval was granted in August 2024 following a review by a data protection officer. The body approved use of the data in line with the project proposal for the publication of research entering the public

domain. Customers were informed through the bank's privacy notice[59] at account opening that their personal data may be used for behavioral segmentation to better understand customer needs, and that aggregated research findings and insights may be shared beyond the bank."

-Data availability. It currently states that it would be available upon reasonable request. We avoid this language to avoid unclear messaging, so please add the specific conditions through which it would seem a reasonable request. Some examples could be for research purposes only, for projects with IRB approval, etc. Would, for example, a minimal set of anonymized data be possible to upload? It could also be an aggregate dataset, a capsule dataset, or other forms of data that would allow for reproducibility of the findings.

The individual-level data used in this study are proprietary to the bank and constitute personal data subject to UK GDPR. Public release is not permitted due to confidentiality, contractual, and regulatory constraints. Any data access requests would be subject to approval by Lloyds Banking Group under its internal data governance, legal, and regulatory review processes.

To support reproducibility despite these restrictions, in the accompanying code, we provide R and Python code that produces a model-derived synthetic individual-level dataset that reproduces the aggregate statistical properties of the original dataset. Users can choose what percentage of the original dataset they would like use for the synthetic data generation. We propose this more flexible approach rather than providing a static synthetic dataset to avoid storing a large pre-generated dataset, and enable users to generate synthetic data at different scales for replication and robustness checks. If preferred, we can additionally provide a static, pre-generated synthetic dataset produced using the same procedure.

Below we show Figure 1 produced from the original data (left) and the replication obtained via running the accompanying code (using 25% of the original data size; right):

Original figure

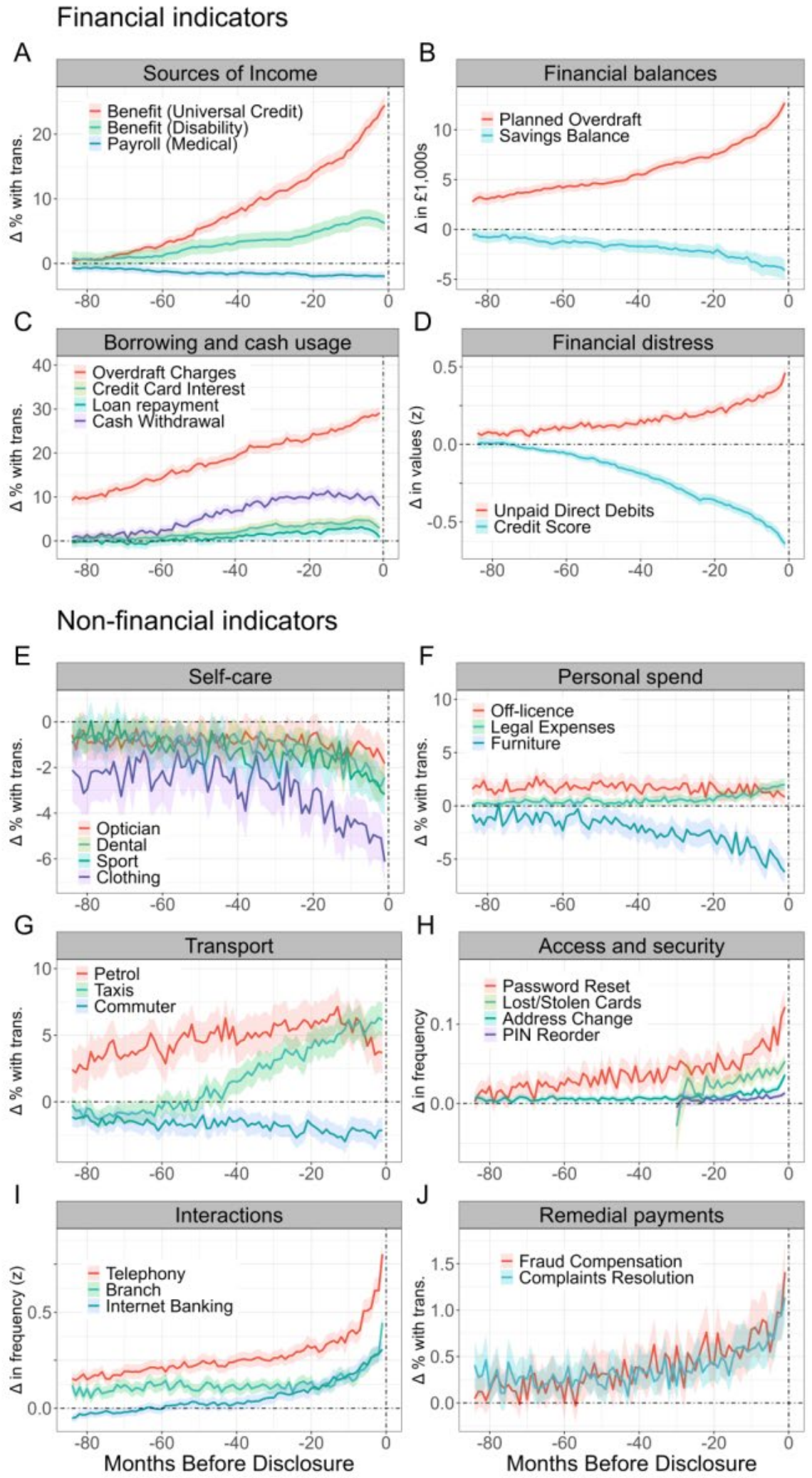

Replicated figure

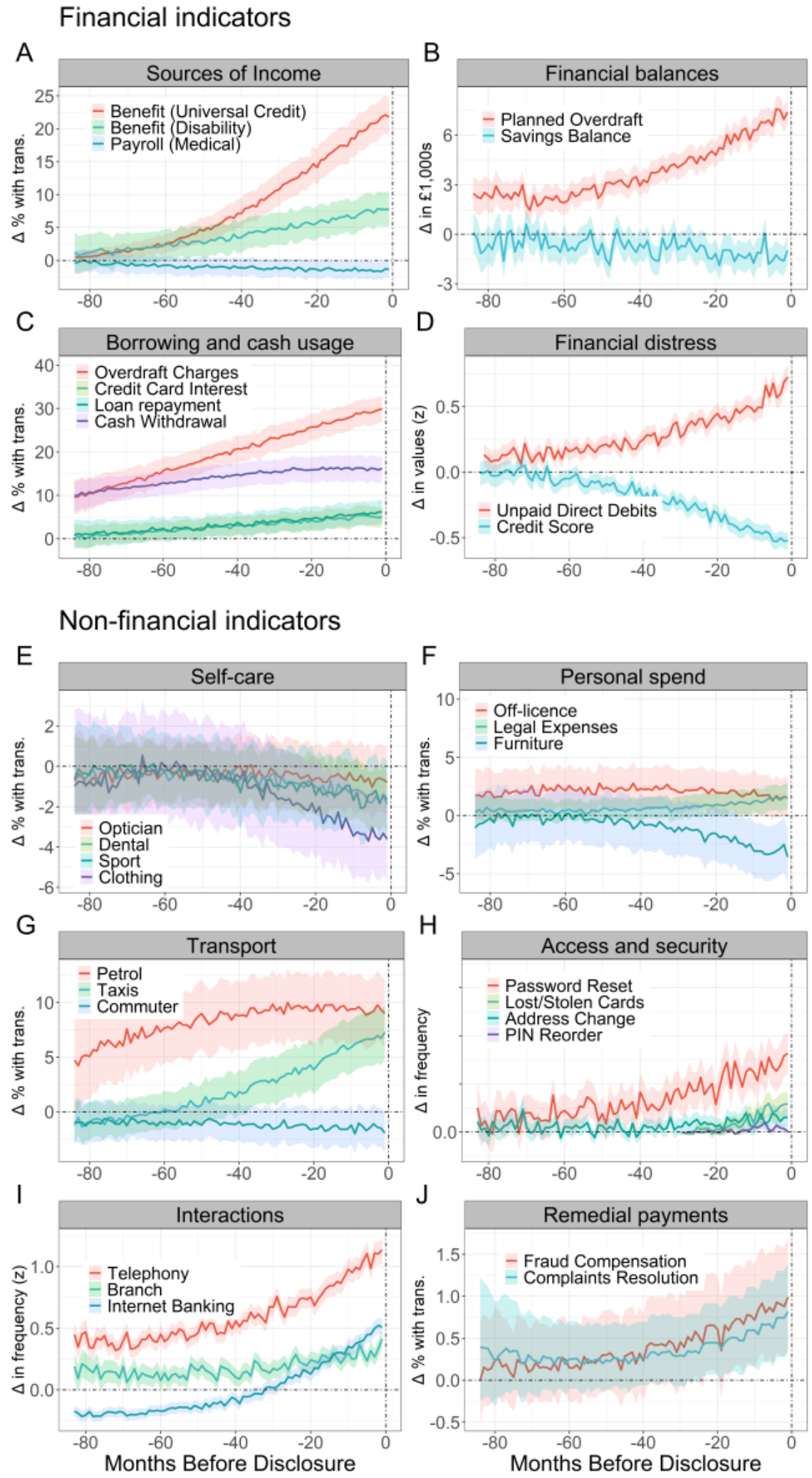

We have revised the Data Availability statement (lines 778-784) accordingly:
"Access to the raw data is restricted owing to confidentiality, contractual, and regulatory obligations associated with customer banking records. Any access to the underlying data is subject to approval in line with Lloyds Banking Group's internal governance, data privacy and security processes. To facilitate reproducibility despite this restriction, the accompanying code generates a model-derived synthetic dataset reproducing the aggregate statistical properties of the original dataset used in the study. The code that generates this dataset and reproduces the main results of the study is available here."

-Code availability. Similar to the previous point, we would need details on what would make it a reasonable request or you would need to provide elements of the code or pseudocode, etc., to ensure reproducibility.

We have updated the Code Availability statement to clarify the scope of the SQL, R, and Python code and the restriction on SQL code access. The R and Python code implement the full analytical workflow and generate the synthetic dataset used in all analyses presented in the paper, ensuring the pipeline is fully reproducible without access to confidential raw data.

We have also updated the README and pseudocode to document the data generation steps and clustering analysis that produce Figures 2 and 3.

The revised Code Availability statement (lines 786-796) reads:

"Data were extracted from the bank's internal secure systems using Teradata SQL Assistant (v.16.20.0.12); the SQL code used for data extraction is commercially sensitive and therefore not publicly available. Any access to the underlying code is subject to approval in line with Lloyds Banking Group's internal governance and security processes. The analysis presented in the study was conducted using R (4.3.0) and Python (3.12.2). The full analytical workflow of this analysis is provided in the accompanying code, which generates a model-derived synthetic dataset that preserves the aggregate statistical properties of the original data and replicates the analysis steps used to obtain the main results presented in the paper."

You will also need to make some editorial changes to your paper so that it is as brief as possible and complies with our Guide to Authors. We also strongly suggest that your revised manuscript has tracked changes, which is increasingly requested by referees to aid in their re-review.

Our revised manuscript shows tracked changes using the MS Word tracked change function.

We would appreciate your careful attention to the following:

STATISTICS: When revising your manuscript, you should ensure that any statistical analysis used is sound and that it conforms to Nature's guidelines). A collection of articles explaining the basics of statistical analysis and advice on how to best present it can be found here.

Statistics are reported in conformity with Nature's guidelines.

REPRODUCIBILITY: All of the checklists provided with the current submission (Reporting summary, and Code and software checklist (if applicable)) should be updated to reflect the revisions made and submitted with the revised manuscript.

Updated checklists have been uploaded with this latest submission.

*LENGTH: In print, biological sciences papers do not normally exceed 8 **OR 6** pages on average; the final print length, however, is at the editor's discretion. The typical length of an 8-page article with 5 modest (quarter-page) display items is 4300 words. **OR The typical length of a 6-page article with 4 modest display items (figures and tables) is 2500 words (summary paragraph plus body text).** If a composite figure (with multiple panels) must occupy at least half a page in order for all the elements to be visible, the text length may need to be reduced accordingly to accommodate such figures. Essential but technical details can be moved into the Methods or Supplementary Information (see below).*

Our article has two large display items (Figures 1 and 2) and one modest display item (Figure 3), which we judge equates to 5 modest display items. Excluding abstract and figure legends, the article contains 4,283 words. We suggest that if space-saving reduction is required, Figure 2 could be moved to the supplementary items.

TITLE: Titles cannot exceed 75 characters (including spaces); they must not contain punctuation.

We have slightly adjusted the title by removing "the". The new title is 75 characters exactly including spaces.

SUMMARY PARAGRAPH: All Nature papers begin with a fully referenced paragraph, typically no longer than 200 words, aimed at readers in other disciplines. This paragraph starts with a 2- to 3-sentence, basic introduction to the field; continues with a 1-sentence statement of the main findings starting 'Here we show' or an equivalent phrase; and finally, concludes with 2 to 3 sentences putting the main findings into general context so it is clear how the results described in the paper have moved the field forward. A downloadable, annotated example is available here. In some cases it may be necessary to exceed this limit modestly in order to explain complex material for readers in other fields. The extra length, however, is for introduction and context, and not for additional technical information.

The summary paragraph is 193 words and follows the suggested structure.

MAIN TEXT: If further introductory material is necessary, the main text can begin with up to 500 words of introduction expanding on the background to the work (some overlap with the summary is acceptable), before proceeding to a concise, focused account of the findings, and ending with 1 or 2 short paragraphs of discussion. Sections are separated with subheadings (up to 40 characters including spaces) to aid navigation.

The first four paragraphs of the introduction provide background, running to 431 words. The fifth paragraph, opening "*While financial abuse leaves no physical marks, it creates clear, measurable traces in financial records*", introduces the data and focused account of the findings. We can provide additional subheadings to break up this section into subsection if required.

REFERENCES: As a guideline, most papers should include no more than 50 main text references; additional references can be cited in (and listed after) the Methods section, as detailed below.

We have reduced the number of references cited in the main text to 50.

FIGURE LEGENDS: These should be listed sequentially after the main text references and not in the figure files; they should not exceed 300 words each. Each legend should begin with a brief title for the whole figure and continue with a short description of each panel and the symbols used. Each figure legend should contain, for each panel where relevant, the following information:
** the exact sample size (n) for each experimental group/condition, given as a number, not a range;*
** a description of the sample collection allowing the reader to understand whether the samples represent technical or biological replicates (including how many animals, litters, cultures, etc);*
** a statement of how many times the experiment shown was replicated;*
** definitions of statistical methods and measures:*
** very common tests (e.g., t-test, simple Chi-square tests, Wilcoxon and Mann-Whitney tests) can be identified by name only, but more complex techniques should be described in the Methods;*

** whether tests are one-sided or two-sided;*
** whether there are adjustments for multiple comparisons;*
** the statistical test results (e.g., P values);*
** the definition of 'center values' as median or average;*
** the definition of error bars as s.d. or s.e.m.*
Any descriptions too long for the figure legend should be included in the Methods section; see here for further explanation.

We have limited the figure legends to 300 words and placed these after the main references.

METHODS: After the main text figure legends there should be a section entitled "Methods", which provides the full, step-by-step instructions that would allow other researchers to replicate the results. The Methods section will not appear in print but will appear online in the full-text HTML and PDF versions. The Methods section should be written as concisely as possible but should contain all elements necessary to allow interpretation and reproduction of the results. If there are additional references in the Methods section, their numbering should continue from the last reference in the main text, and they should be listed following the Methods section. Specialized methods that require chemical structures, figures or tables, or methods requiring equations, cannot be accommodated in the Methods section of the main text file. If such information is part of the Methods, the entire Methods section must instead be included within a Supplementary Information text file.

The methods section enclosed conforms with these requirements.

ETHICS STATEMENT: For research involving human research participants, the Methods section must include an ethics statement. This statement should provide the name of the committee that approved the study; confirm that the research was performed in accordance with all relevant guidelines and regulations; and confirm that informed consent was obtained from all participants. If the study was granted an exemption from requiring ethics approval, details of the committee granting the exemption must be included.

We have revised the ethics statement to meet these requirements.

MAIN TEXT STATEMENTS: Several statements (which will not appear in print but will appear online in the full-text HTML and PDF) are required after the Methods, and before the Extended Data legends. First, there should be an Acknowledgements section, listing grant/financial support. Next, we require a detailed Author Contribution statement; the specific contributions of each author, particularly in terms of which authors performed which specific experiments, must be listed. This is followed by a Competing Interest statement. Financial or non-financial interests should be noted here, as well as any patents; patent information should include at a minimum what is covered by the patent and who submitted the patent application. Finally, an Additional Information statement should include information regarding reprints and permissions and name the author(s) to whom correspondence and requests for materials should be addressed. For details of "end note" style and an example see here.

Main text statements are enclosed.

DATA AND CODE AVAILABILITY STATEMENTS: All original research manuscripts published in Nature Portfolio journals must include a Data availability statement (DAS). This statement must make the conditions of access to the "minimum dataset" that is necessary to

interpret, verify and extend the research in the article, transparent to readers. This minimum dataset may be provided through deposition in public community/discipline-specific repositories, custom proprietary repositories for certain types of datasets, or general repositories like Figshare, Zenodo and Dryad. Providing large datasets in supplementary information is strongly discouraged and the preferred approach is to make data available in repositories. More information on Nature Portfolio's reporting standards and preparing your Data availability statement can be found here.

For all studies using custom code or mathematical algorithms that are deemed central to the conclusions, a Code availability statement (CAS) must be included, indicating whether and how the code or algorithm can be accessed, including any restrictions to access. the CAS should be provided as a separate section after the DAS but before the references. Code should be deposited in a DOI-minting repository such as Zenodo, Gigantum or Code Ocean and cited in the reference list. We encourage you to manage subsequent code versions and to use a license approved by the open source initiative. Additional details can be found here.

Wherever possible the data used in the paper should be placed into a public data repository or presented as Supplementary Information. If data can only be shared on request, please explain why in the DAS and in the cover letter. The DAS must list which data are included (e.g. by figure panels and data types) and mention any restrictions on availability. If a dataset generated or analysed during the study is publicly available and has a unique DOI, please include it in the reference list and cite the dataset in the Methods. Accession numbers for any newly determined sequences, structures, microarray or zoobank data; project IDs for MG-RAST data; accession numbers for X-ray crystallographic coordinates and structure factor files; or comparable NMR or cryoEM data, should be included only in the DAS.

Please see our in-line responses to points raised by the editor, above. We have amended the manuscript with an updated ethics statement, data availability statement and code availability statement.

DISPLAY ITEMS: We ask that you take stock of all the data that have been generated throughout the review process and ensure that only the data most central to the conclusions are presented in the main text figures. Although figures can be presented within the text file during the review process, they must be removed from the final text file and uploaded as separate individual files. Figures should be comprehensible to readers in other or related disciplines and assist their understanding of the paper. Figures should be as small and simple as is compatible with clarity. Main text figures (but ***not*** *Extended Data) must be provided in an editable format. Acceptable formats include .ai, .cmx, .cdr, .doc, .eps, .pdf, .ppt, .ps, .psd, .svg and .xls; unacceptable formats include .cvs, .gif, .jpg, .png and .tif. All panels of a figure should be logically connected; each panel of a multipart figure should be sized so that the whole figure can be reduced by the same amount and reproduced on the printed page at the smallest size at which essential details are visible. For guidance, Nature's standard figure sizes are 9, 12, or 18 cm wide; the maximum permitted height of a figure is 17 cm. All panels of figures must be on a single page and assembled into a rectangular shape for publication; any essential alignments (parts horizontal, vertical, spacings of stereo pairs, etc) should be indicated. Nature requests that authors of accepted manuscripts contribute towards the total cost of reproduction of colour figures in print. You will be charged a fee for the print reproduction of colour figures (see here for cost information). Tables should be prepared using the Table menu in Microsoft Word.*

We have reviewed the data generated and the items appearing in the manuscript are only those most central to the results.
FIGURE FORMATTING: Lettering in all figures (e.g., labelling of axes) should be in uniform, sans-serif font, in lower-case type, and large enough to permit substantial reduction for publication (the minimum font size permitted is 5 pt). Separate parts of a figure are labelled a, b, etc. Units have a single space between the number and the unit, and follow SI nomenclature or the nomenclature common to a particular field. Thousands are separated by commas (1,000). Unusual units or abbreviations are defined in the legend.

Figure formatting is in line with these requirements.

IMAGE PRESENTATION: Data figures are integrated into the main paper online. An overview of the key features of this presentation may be found here. We strongly encourage you to consult the guidelines. A discussion of our standards regarding how images should be prepared and presented can be found here.

Figures included in the manuscript follow these guidelines.

EXTENDED DATA: Extended Data do not appear in print but are included online within the full-text HTML and integrated in the downloadable PDF. Extended Data are an integral part of the paper, and only data that directly contribute to the main message should be included. All Extended Data must be referred to in the main text, and their legends should be listed sequentially at the end of the main text, not in the Extended Data files. The Extended Data should be assembled into a maximum of 10 A4 size, multi-panelled display items, submitted as individual files in .jpg, .tif or .eps format only. They should be of the same quality as print figures, but there are important differences in their formatting. More specific instructions can be found here. We encourage authors who are describing complex processes to include a schematic of the main finding as part of the Extended Data to aid readers unfamiliar with the immediate discipline.

Our manuscript is not accompanied by extended data.

SUPPLEMENTARY INFORMATION: Supplementary Information (SI) is online-only, peer-reviewed material that is essential background to the study (e.g., large data sets, more complex methods, and calculations), but which is too large or impractical, or of interest only to a few specialists, to justify inclusion in the print version of the paper (see here for further details). While SI should not typically contain data figures (any figures additional to those appearing in the main text should be formatted as Extended Data), we require that the raw, uncropped data for gels be presented as an SI figure (see below). Tables may be included in SI, but only if they are unsuitable for formatting as Extended Data (e.g., tables containing large data sets or raw data tables that are best suited to Excel files). If a manuscript has SI, each discrete item of the SI (e.g., videos, tables) must be referred to at an appropriate point in the main manuscript. We ask that you provide a word file entitled "SI Guide", containing a cover page with manuscript title and author information; a table of contents (preferably with page numbers); and then any SI text, notes, figures, and titles and legends for any separate SI files. Additional information can be found here.

Please pay careful attention to the formatting of the SI because it is not subedited. After the paper has been accepted, SI files can only be amended for critical changes to the scientific content, not for style.

Supplementary items are included in the manuscript, formatted in line with these requirements.

SOURCE DATA (GRAPHS): To increase transparency, we strongly encourage you to provide, in spreadsheet form, the data underlying the graphical representations used in the figures. In the case of all experiments presenting data from animal models, this is a requirement and is not optional. This is in addition to our well-established data-deposition policy for specific types of experiments and large datasets. Online readers of the manuscript will be able to access the graphical source data directly from the figure legend. Spreadsheets can only be submitted in .xls, .xlsx or .csv formats. One file per figure is permitted. If there is a multi-panelled figure, the source data for each panel should be clearly labeled in the file; alternatively the source data for a figure can be included in multiple, clearly labeled sheets within an Excel file. File sizes of up to 30 MB are permitted, but it is expected that the vast majority of graphical source data files will be considerably smaller than this. When submitting these files with your manuscript, you should select the "Source Data" file type and use the title field in the file description tab to indicate the figure(s) to which the source data pertain.

As stated in the Data Availability statement, the individual-level data underlying the results presented in the paper are not publicly available. To support reproducibility despite these restrictions, we provide code that generates a model-derived synthetic individual-level dataset that reproduces the aggregate statistical properties required for the analyses. This synthetic dataset is generated within the replication pipeline and is used to execute all reported analyses and produce the figures and tables in the manuscript.

Rather than distributing a standalone synthetic dataset, we adopted this approach to enable reproducibility while avoiding the need to store or share large volumes of synthetic individual-level data. It also allows users to generate datasets at different scales for replication and robustness checks. If preferred, we can additionally provide a static, pre-generated synthetic dataset produced using the same procedure.

THIRD PARTY RIGHTS: Please identify any content used in your article, whether in the main text, extended data or supplementary information, that comes from a third party. This could include figures, tables, images, videos or text boxes that are reproductions or adaptations of items that have previously been published elsewhere and/or are owned by a third party. It also encompasses pictures taken by professional photographers, maps and images downloaded from the internet. You must obtain the right to use each of these items and provide evidence that you have these rights for your paper. You will also need to give proper attribution to the copyright holders in your paper. We ask that you fill out a Third party rights table and upload this with your manuscript. You can find more information about third party rights here.

There is no third party content in the article.

ORCID: As part of our efforts in improving transparency in authorship, we now request that all authors identified as 'corresponding author' create and link their Open Researcher and Contributor Identifier (ORCID) with their account on Nature's manuscript tracking system prior to acceptance. ORCID helps the scientific community achieve unambiguous attribution of all scholarly contributions. More information and instructions on how to associate your

ORCID and MTS accounts can be found here. If you have any issues attaching an ORCID identifier to your MTS account, please contact the Platform Support Helpdesk.

The corresponding author has linked their ORCID on the manuscript submission system.

When revising your paper:

** Include a point-by-point response to our referees and to any editorial suggestions;*

** Complete the Reporting summary;*

** Complete the Code and software checklist (if applicable);*

** Ensure it complies with our format requirements.*

The revision can be submitted using the following link:

https://mts-nature.nature.com/cgi-bin/main.plex?el=A3K2BMf5A4MKWo1J5A9ftdTRt7FfBjz7FeFybH2dbBAZ

***NOTE**: This url links to your confidential home page and therefore to manuscripts you may have submitted or be reviewing for us. If you wish to forward this email to co-authors, please delete the link to your home page first.*

All correspondence should contain your Nature reference number in the subject line.

We hope to receive your revised paper within four weeks. If you cannot send it within this time, we will still be happy to reconsider your paper at a later date as long as nothing similar has been accepted for publication at Nature or published elsewhere in the meantime.

We look forward to hearing from you soon.

Yours sincerely,

Sebastián Villamizar Santamaría
Senior Editor
Nature

Referee expertise:

Referee #1: IPV, economic abuse

Referee #2: banking economics, inequality

Referee #3: banking economics, inequality

Referee #4: economic sociology, banking

Referee #5: IPV, economic abuse

Referees' comments:

***Referee #1** (Remarks to the Author):*

Thank you for the opportunity to review the revised draft of this manuscript, focused on victim-survivors banking records. As I noted in my original review, this study provides a unique and valuable insight into survivors' financial abuse experiences viewed through their banking patterns.

I think the authors did an excellent job of addressing reviewer feedback. While this type of statistical analyses is not my expertise, the the findings and implications are clear.

The authors removed causal language, instead emphasizing associations found within the data. The addition of the sensitivity analysis, particularly by repeating the analysis with the distress-matched sample, helps to isolate the behaviors associated with intimate partner violence survivorship, as the authors note in their response.

With regard to the comment "seemingly conflicting finding that victim-survivors simultaneously increase their borrowing and experience declining credit scores" – I appreciate that the authors used credit application data to provide further context. However, these findings also seem reasonable to me since increasing borrowing does have the potential to decrease credit score.

In my reading of the revised draft, it occurred to me that the limitations section might be the first time that the manuscript really explicitly states the gender of victim-survivors in the sample ("First, the victim-survivor group, comprising of women in the UK with bank accounts…") Because of this, the use of the female bank population benchmark also seemed to be missing context. Perhaps the gender of the sample could be noted more clearly earlier in the paper. Also, was this a specific sampling choice or did only women utilize the financial and domestic abuse team?

We thank the reviewer for their kind words. We agree that the gender of victim-survivors should be clarified earlier in the manuscript. This restriction was made owing to the fact that the vast majority of the victim-survivor sample (87%) was women. Restricting the analysis to women therefore improved statistical robustness and interpretability by avoiding inference based on a comparatively small male subgroup.

We made the following revisions to more clearly reflect this restriction:

Abstract (lines 17-18)

- *"…group of **female** victim-survivors of financial abuse…"*

Introduction (line 87)

- *"**Female** victim-survivors self-disclosed their experiences to the bank's specialist financial and domestic abuse team…"*

I appreciate the authors' decision to include both financial and non-financial outcomes and agree they are relevant to providing context to the various domains impacted by abuse.

Overall, the discussion of study limitations is much more robust.

Referee #1 (Remarks on code availability):

I was able to access the code, however, I did not attempt to open it because I do not use R.

We would like to thank the reviewer for their comments the revised version of our paper.

***Referee #2** (Remarks to the Author):*

The authors have been responsive to my concerns. The reframing of the paper from a predictive exercise to a descriptive characterization of financial abuse in banking data resolves the methodological tension I identified in Round 1 between the stated goals and the PSM design. The bank population benchmarks now provide the effect-magnitude context I requested, and the disclosure process is adequately explained. Code reproducibility improvements have been implemented. I am satisfied that my comments have been addressed.

Malcolm Wardlaw

We would like to thank the reviewer for their comments on the revised version of our paper.

***Referee #3** (Remarks to the Author):*

I appreciated the revision and I would like to thank the authors for their work. While the paper cannot establish causality, the authors now make clear the scope of this work and more cautiously refer to associations. This is useful. I also appreciated the careful work in documenting the external validity of the analysis and in defending the representativeness of the sample.

I have one remaining comment that bundles together two comments in my previous report. The authors argue that the various forms of abuse occur more or less at the same time, so it is indeed very difficult to separate the specific effects of financial abuse. At the same time, in the revised version they keep on focusing on the same very wide range of outcomes, with no particular priority in their discussion in the text. Given the difficulty of properly controlling for forms of non-financial abuse, I find this strategy of presenting such a wide range of outcomes not particularly convincing. It can be natural for a reader to tie, say, the use of

particular types of transportation to forms of physical or psychological abuse, rather than primarily to financial abuse. In the absence of a clear distinction among the effects of various types of abuse, presenting on the same level such a wide range of outcomes can weaken the message and raise a reader's concerns about omitted variables issues.

It's of course up to the Editor's judgement, but my recommendation is then (in a minor revision) to focus more on financial and economic outcomes that can be more closely linked to financial abuse and reduce the emphasis on outcomes that are far away from financial abuse. Such additional outcomes could be presented and discussed in a somewhat shorter way in the text, as additional outcomes, with related figures given less priority. This would also allow to better clarify the comments on more salient outcomes and instead shorten the comments on outcomes and mechanisms that are less clearly related to financial abuse and that currently appear to be disconnected from the core of the paper.

Referee #3 (Remarks on code availability):

The code appears to be in good order.

We would like to thank the reviewer for their comments on the revised version of our paper. We agree that some outcomes are plausibly more closely aligned to financial abuse and some outcomes may be affected by other factors, such as the correlates of financial abuse.

At the same time, we believe an important contribution of the paper is demonstrating how banking data can capture a broad spectrum of experiences associated with victim-survivors' financial lives, including outcomes extending beyond narrowly defined financial indicators. To address the reviewer's concern while preserving this broader perspective, we have revised the presentation and discussion of the results to more clearly distinguish between financial and non-financial indicators.

Specifically, Figure 1 has been reorganized so that the top four panels present financial indicators and the bottom six panels present non-financial indicators. Cash withdrawals have been regrouped with debt-related indicators under the revised heading "Borrowing and cash usage". The corresponding Results section has also been restructured into separate subsections titled "Financial indicators" and "Non-financial indicators".

We hope this revised structure improves clarity regarding which findings are most directly connected to financial abuse while retaining the broader set of outcomes that illustrate the breadth of insight available from banking data. If preferred by the editor, we would also be willing to move selected non-financial indicators from the main text to the Supplementary Information.

Referee #4 *(Remarks to the Author):*

The authors have carried out a thorough and rigorous revision in this second version and have addressed the comments I previously raised, in particular regarding the representativeness of the sample, the possibility of constructing a cluster of variables, and the comparison with individuals experiencing financial distress who are not victims of violence. The inclusion of the suggested references should also be noted.

I am therefore in favor of the publication of this article in its current form, and I would like to

reiterate the importance of the topic and the originality of the approach, which I hope will inspire further research.

Jeanne Lazarus

We would like to thank the reviewer for their comments on the revised version of our paper.

***Referee #5** (Remarks to the Author):*

Thank you for the opportunity to review the revised version of this important manuscript. I believe that the authors did a tremendous job responding to the many concerns expressed by the five reviewers. They have certainly addressed my own suggestions.

The only thing I want to suggest now is that the authors review the manuscript one more time for language that implies causation. For example, this revised line still implies causality with the word "shapes." Current evidence on how financial abuse shapes the everyday experiences of its impact on victim-survivors' lives survivors is drawn primarily from small-scale, qualitative surveys." It would be good to shift to words like "is related to" here and possibly elsewhere.

Otherwise, I think this will make a terrific contribution.

Best, Lisa Goodman

Referee #5 (Remarks on code availability):

We would like to thank the reviewer for their comments on the revised version of our paper.

In light of the suggestion from the reviewer, we have amended the manuscript as follows:

Abstract (lines 15-16)

- *"Current evidence on ~~how financial abuse shapes~~ the everyday experiences of victim-survivors **in the context of financial abuse** is drawn primarily from small-scale, qualitative surveys"*

Revision of Nature manuscript 2025-09-23824

"Victim-survivors' banking records reveal characteristics of financial abuse"

Editor Letter

Dear Dr Trendl,

Your manuscript, "Victim-survivors' banking records reveal characteristics of financial abuse", has now been seen by our referees, and in the light of their advice I am delighted to say that we can in principle offer to publish it. First, however, we would like you to revise your paper to address the points made by the referees, and to make some editorial changes to your paper so that it is as brief as possible and complies with our Guide to Authors. No peer reviewed data should be removed altogether when making these changes.

Thank you for the in-principle offer to publish our manuscript. We are delighted to receive this decision and appreciate the constructive feedback from you and the reviewers. Please see the decision letter and reviewer comments enclosed below in italics, with our responses shown in plain type.

1-Please address the remaining referee comments regarding the code.

We thank the editor and reviewer for these helpful comments. We have updated the code repository to address all remaining code-related suggestions. This included incorporating the additional statistical reporting requested during review, revising the Figure 2 visualization to display individual data points rather than summary bars alone, improving the reproducibility documentation and execution pipeline through the inclusion of setup.sh and run_analysis.sh, and implementing the additional changes detailed in our point-by-point responses below.

2-Statistics: Please add direct comparisons to your quantitative descriptive results, i.e. where you say the amounts and how they are different, please add statistics to show whether these differences are statistically significant or not. When you do this, please be sure that all statistical details are reported in full (name of the test, test statistic, sample size/degrees of freedom, p value, effect size, when appropriate, etc., in the Results, etc.). Please also be sure to check the test assumptions and to correct for multiple comparisons, when necessary. If you do get non-significant results, please do not interpret them unless you add statistical support for the null (e.g. equivalence test or Bayes factors) and throughout the manuscript. If you have this information in a figure legend, please add it in the Main text and vice versa.

We thank the Editor for this suggestion. We have revised the Results and Methods to provide more complete statistical reporting throughout the manuscript. Specifically, we have added the statistical test used for each comparison, the corresponding test statistic and P value, applied Benjamini–Hochberg false discovery rate correction where appropriate, clarified sample sizes where these differed across analyses, and ensured that statistical details are reported consistently across the main text, tables, and figure legends.

Because significance was assessed using permutation-based inference (see lines 539-552), *P* values were obtained empirically from permutation distributions rather than theoretical parametric distributions. Consequently, degrees of freedom are not applicable and are therefore not reported. The temporal clustering analyses already controlled the family-wise error rate using a permutation-based maximum-cluster procedure (see lines 553-565 of the

revised manuscript), while the outcome-level analyses now additionally incorporate false discovery rate correction (see lines 549-551 of the revised manuscript).

We continue to report the observed group differences together with their 95% confidence intervals as the primary measures of effect size because they provide directly interpretable estimates on the natural scale of the outcomes.

Lastly, we have revised the manuscript to avoid interpreting non-significant findings as evidence of no effect. Specifically, we now report that we found no evidence of group differences in successful credit card or loan applications, rather than stating that the groups did not differ (lines 150-152 of the revised manuscript).

3- Please reduce the length of the title to 75 characters (with spaces) or less, so that it fits on two lines in the final layout. We suggest a version of "Financial abuse is associated with a subsequent loss of financial independence."

Thank you for your comment. We believe the current title, "Victim-survivors' banking records reveal characteristics of financial abuse", is 75 characters (including spaces) and therefore meets the requested limit.

4-Please reduce subheadings to 40 characters (with spaces) or less.

Thank you for your comment. We have shortened the subheadings to meet the 40-character limit. Specifically, "Replications for alternative temporal markers and comparison groups" has been revised to "Alternative Comparison Groups", and "Temporal pattern of group differences (primary matched sample)" has been revised to "Temporal Patterns of Differences".

5-Please create a separate reference list for any methods references, making sure that the numbering continues from the main text references.

We have split the references into Main text and Methods references.

6-Please remove the main figures from the article file and re-supply them individually in an acceptable format such as EPS, AI, PS, PDF, PPT, PSD or XLS (for graphs) with editable vector files.

We have removed the main figures from the article file and resupplied them as separate PDF files.

7-Please provide a supplementary information guide as a separate word document.

We have added a separate supplementary information guide SI_guide.docx.

8-The funding information has been merged with the acknowledgment section; please provide it as a separate section.

The funding information has been moved from the Acknowledgements to a separate Funding information section, as requested.

9-You have more than one account on EJP. Please contact Nature Manuscripts (naturemanuscripts@nature.com) to confirm all accounts which belong to you, and your primary email address.

We have contacted Nature Manuscripts to confirm all EJP accounts associated with the corresponding author and to identify the preferred primary email address, as requested.

10-We have noticed that not all corresponding authors of the manuscript are ORCID linked. As part of our efforts to improve transparency in authorship, we now request that all authors identified as 'corresponding author' create and link their Open Researcher and Contributor Identifier (ORCID) with their account on the Manuscript Tracking System (MTS) prior to acceptance. For more information please visit http://www.springernature.com/orcid. If you have an alternative account which is ORCID linked, please contact Calla Smith-Triglia (calla.smith-triglia@us.nature.com).

The corresponding author has their ORCID linked to their Nature account.

11-Please remove the supplementary figures and tables from the article file and re-supply them individually in EPS, JPEG or TIF format.

We have removed the supplementary figures and tables from the article file and resupplied the figures as separate JPEG files.

12-Please ensure that the text size in all figures is at least 5 pt Arial.

Thank you for your comment. We believe that all figures already met this criterion, with the possible exception of Figure 2, which includes the names of 116 financial indicators. To improve readability, we have simplified the financial indicator names as part of this revision. If you consider it necessary, we would also be happy to provide an alternative version of Figure 2 in which the figure is split into two panels arranged horizontally to allow for larger text.

13-Ethics: Please clarify how many specialists were involved and how they were chosen Also, please confirm whether customers could opt out, or if having their data used this way was required to open an account?

We have updated the ethics statement in the article to clarify these points:
"Prior to analysis commencing, approval was obtained following review by the bank's Privacy Risk and Impact Assessment (PRIA) governance body. Following an initial review by a Data Privacy Manager and a Data Privacy Risk Specialist, the application underwent an additional review by a Group Data Protection Officer, in accordance with the bank's governance procedures for projects involving data relating to vulnerable customers. The reviewers were assigned according to the bank's standard PRIA governance procedures and were independent of the project team. The PRIA application for this project was submitted in March 2024, and approval was granted in August 2024. The body approved use of the data in line with the project proposal for the publication of research entering the public domain. Customers were informed through the bank's privacy notice at account opening that their personal data may be used for behavioral segmentation to better understand customer needs, and that aggregated research findings and insights may be shared beyond the bank. Although the bank's data privacy notice covers the use of customer data for research purposes, customers retained their existing rights to request the erasure of, or object to the processing of, their personal data, and use of their data for research was not a requirement of holding an account."

14-Code and Data Accessibility Statements: Please provide more information about what people need to do to obtain access. As you say it's subject to approval, does this mean that

people can request approval? To you or the bank? Also, please specify the link rather than use a hyperlink.

We have updated the Code and Data Accessibility Statements to clarify that access requests should be directed to the corresponding author. We have replaced the hyperlinks with the full link.

For any Supplementary Figures, please check and confirm that:
** If data is presented as bar charts, individual data points are shown using overlaid dot plots.*
** The n number (i.e. the sample size used to derive statistics) is provided and defined as a precise value (not a range), using the wording "n=X samples/cells/independent experiments" etc. where applicable.*
** Any chart axis, error bars, scale bars, symbols and colour scales are defined.*
** Any statistical tests used for data analysis are specified and exact p-values are provided either on the figures themselves, in the legend or in the Source Data file.*
** Wherever representative data such as micrographs are shown, the legend indicates how many times the experiment was repeated with the same results.*

Thank you for your comment. We have revised the supplementary figures to ensure they comply with these requirements. In particular, we have updated Supplementary Figure 3 to display individual data points instead of bar charts. We have also reviewed all supplementary figures and legends to ensure that axes, symbols, statistical tests, sample sizes, and other relevant information are clearly defined where applicable.

TRANSPARENT PEER REVIEW: Nature uses a transparent peer review system for original research manuscripts. We encourage increased transparency in peer review by publishing the referee comments and the authors' rebuttal letters. Such peer review material is made available as a supplementary peer review file.

Please note: We allow redactions to authors' rebuttal and referee comments in the interest of confidentiality. If you are concerned about the release of confidential data, please let us know specifically what information you would like to have removed. We cannot incorporate redactions for any other reasons. Referee names will be published in the peer review files if the referee signed the comments to authors, or if the referee explicitly agrees to release their name. For more information, please refer to our website.

***ORCID--IMPORTANT**: All authors identified as 'corresponding author' on the manuscript must have an ORCID associated with their Nature account before submitting the final version of the manuscript. While non-corresponding authors do not have to link their ORCIDs, they are encouraged to do so. Please note that it will NOT be possible to add/modify ORCIDs at the proof stage. Thus, if they wish to have their ORCID added to the paper they must follow the above procedure prior to acceptance. If you have any issues attaching an ORCID identifier to your Nature account, please contact the Platform Support Helpdesk.*

We have ORCID identifiers for all co-authors. However, these ORCIDs do not appear in the Manuscript Tracking System. Please let us know if any further action is required on our part to ensure that the co-authors are correctly linked to their Springer Nature accounts and that their ORCID identifiers are correctly associated with this submission before acceptance.

In order to avoid delays with publication of your manuscript, please read the guidelines below carefully before resubmission of your manuscript.

STATISTICS: When revising your manuscript, you should ensure that any statistical analysis used is sound and that it conforms to our guidelines. A collection of articles explaining the basics of statistical analysis and advice on how to best present it can be found here.

REPRODUCIBILITY: To ensure that the quality and transparency of methods and statistical reporting (as discussed here) are sound before the paper is published, we have reviewed your Reporting summary editorially. I have attached two documents: one listing specific issues related to your manuscript and one containing an annotated version of the Reporting summary. Please ensure that, as well as the more general points below, the points highlighted in the attached documents are addressed in full, both on these forms and within the manuscript. Both forms should be uploaded as a "Related Manuscript" file type. The Reporting summary will be published with your paper.

LENGTH: In print, biological sciences papers do not normally exceed 8 pages on average; the final print length, however, is at the editor's discretion. The typical length of an 8-page article with 5 modest (quarter-page) display items is 4300 words. If a composite figure (with multiple panels) must occupy at least half a page in order for all the elements to be visible, the text length may need to be reduced accordingly to accommodate such figures. Essential but technical details can be moved into the Methods or Supplementary Information (see below).

In this case, we feel the current length of the paper is appropriate, so no further shortening is necessary; you should not significantly add to the text when revising.

TITLE: Titles cannot exceed 75 characters (including spaces); they must not contain punctuation. See above for a suggestion to be modified.

AUTHOR NAMES USING NON-ROMAN CHARACTERS: We can support presentation of author names using non-Roman characters in the HTML version of the published article. Currently supported scripts include Arabic, Chinese, Cyrillic, Devanagari, Greek, Hebrew, Hangul, Japanese and Persian. If this is relevant to your authors, please include the author names in parentheses after the Roman-character spelling (see an example here). You will be asked to verify the rendering is correct at the proof stage.

SUMMARY PARAGRAPH: Papers start with a fully referenced, bold paragraph, ideally of about 200 words, aimed at readers in other disciplines. Numbers, abbreviations, acronyms or measurements should be avoided unless essential. The summary paragraph consists of 2 to 3 sentences of basic-level introduction to the field; a brief account of the background and rationale of the work; a statement of the main conclusions (introduced by the phrase 'Here we show' or its equivalent); and a conclusion of 2 to 3 sentences putting the main findings into general context so it is clear how the results described in the paper have moved the field forward. A downloadable, annotated example is available here.

MAIN TEXT: If further introductory material is necessary, the main text can begin with up to 500 words of introduction expanding on the background to the work (some overlap with the summary is acceptable), before proceeding to a concise, focused account of the findings, and ending with 1 or 2 short paragraphs of discussion. Sections are separated with subheadings

(up to 40 characters including spaces) to aid navigation.

REFERENCES: As a guideline, most papers should include no more than 50 main text references; all additional references can be cited in (and listed after) the Methods section, as detailed below.

FIGURE LEGENDS: These should be listed sequentially after the main text references and not in the figure files. Each legend should begin with a brief title for the whole figure and continue with a short description of each panel and the symbols used. Legends should not exceed 300 words each. Each figure legend should contain, for each panel where relevant, the following information:
** the exact sample size (n) for each experimental group/condition, given as a number, not a range;*
** a description of the sample collection allowing the reader to understand whether the samples represent technical or biological replicates (including how many animals, litters, cultures, etc);*
** a statement of how many times the experiment shown was replicated;*
** definitions of statistical methods and measures:*
** very common tests (e.g. t-test, simple Chi-square tests, Wilcoxon and Mann-Whitney tests) can be identified by name only, but more complex techniques should be described in the Methods;*
** whether tests are one-sided or two-sided;*
** whether there are adjustments for multiple comparisons;*
** the statistical test results (e.g., P values);*
** the definition of 'center values' as median or average;*
** the definition of error bars as s.d. or s.e.m.*
Descriptions that are too long for the figure legend should be included in the Methods section.

METHODS: The Methods section, which provides the full, step-by-step instructions that would allow other researchers to replicate the results, is included after the main text figure legends. The Methods section will not appear in print but will appear online in the full-text HTML and PDF versions. The Methods section should be written as concisely as possible but should contain all elements necessary to allow interpretation and reproduction of the results. If there are additional references (in the Methods section, Supplementary Information, etc), their numbering should continue from the last entry in the main text reference list, and they should be listed following the Methods section. Specialized methods that require chemical structures, figures, or tables cannot be accommodated in the Methods section of the main text file. If such information is part of the Methods, the entire Methods section must instead be included within a Supplementary Information text file.

ETHICS STATEMENT: For research involving human research participants, the Methods section must include an ethics statement. This statement should provide the name of the committee that approved the study; confirm that the research was performed in accordance with all relevant guidelines and regulations; and confirm that informed consent was obtained from all participants. If the study was granted an exemption from requiring ethics approval, details of the committee granting the exemption must be included.

MAIN TEXT STATEMENTS: Several statements (which will not appear in print but will appear online in the full-text HTML and PDF) are required after the Methods (and

additional references, if present). First, there should be an Acknowledgements section. Second, any relevant funding should be declared in a separate Funding statement; our funding statement guidelines can be found here. Third, we require a detailed Author Contribution statement; the specific contributions of each author, particularly in terms of which authors performed which specific experiments, must be listed. Fourth, a Competing Interest statement noting financial and non-financial interests, as well as any patents, must be provided; patent information should include at a minimum the patent number, what is covered by the patent, and who submitted the patent application. Finally, an Additional Information statement should include information regarding reprints and permissions and name the author(s) to whom correspondence and requests for materials should be addressed. Formatting details and an example are available here.

DATA AND CODE AVAILABILITY STATEMENTS: Any manuscript reporting original research must include a Data Availability statement that makes transparent to the reader the conditions of access to the "minimum dataset" that is necessary to interpret, verify and extend the research in the article. This minimum dataset may be provided through deposition in public community/discipline-specific repositories, custom proprietary repositories (for certain types of datasets), or general repositories like Figshare, Zenodo and Dryad. We strongly discourage providing large datasets in Supplementary Information; the preferred approach is to make data available in repositories. More information on Nature Portfolio's reporting standards and guidance on preparing your Data Availability statement can be found here.

For all studies using custom code or mathematical algorithms that are deemed central to the conclusions, a Code Availability statement must be included, indicating whether and how the code or algorithm can be accessed, including any restrictions to access. The Code Availability statement is listed as a separate section after the Data Availability statement but before any additional references. Code should be deposited in a DOI-minting repository such as Zenodo, Gigantum or Code Ocean and cited in the reference list. Authors are encouraged to manage subsequent code versions and to use a license approved by the open source initiative. Additional details can be found here.

DISPLAY ITEMS: We suggest that you take stock of all data that have been generated throughout the review process and ensure that only the data most central to the conclusions are presented in the main text figures. Any figures included within the main text file during the review process must be removed from the final main text file and uploaded as separate, individual files; they will be integrated into the main paper in print and online. An overview of the key features of this presentation may be found here.

Figures should be comprehensible to readers in other disciplines and assist in understanding of the paper. Main text figures (but ***not*** *Extended Data) must be provided in production-quality versions in an editable format (i.e., .ai, .cmx, .cdr, .doc, .eps, .pdf, .ppt, .ps, .psd, .svg and .xls); we cannot accept figures in .cvs, .gif, .jpg, .png and .tif formats. We highly encourage you to consult our artwork guidelines. They should be as small and simple as is compatible with clarity. All panels of a figure should be logically connected and assembled on a single page in a rectangular shape; any essential alignments (parts horizontal, vertical, spacings, etc) should be indicated. Each panel of a multipart figure should be sized so that the whole figure can be proportionally reduced and reproduced on the printed page at the smallest size at which essential details are visible. Nature's standard figure sizes are either 9 or 18 cm wide; the maximum permitted height is 17 cm. Panels should be arranged to fit*

these widths while minimizing excess space around the panels. Tables should be prepared using the Table menu in Word. As we must be able to edit the figures so that they conform to our house style, the submission of files that are incorrectly formatted, flattened, or of insufficient resolution may delay final acceptance of your manuscript.

THIRD PARTY RIGHTS: You must provide proof that you have secured permission to use any third party materials that appear in any part of your manuscript, including Extended Data and Supplementary Information. Please fill out a Third party rights table, and upload this with the final version of your manuscript. Third party materials include any figures, tables, images, videos or text boxes that are reproductions or adaptations of items that have previously been published elsewhere and/or are owned by a third party. This includes pictures taken by professional photographers, maps and images downloaded from the internet. You will need to obtain the right to use each of these items before your paper can be accepted for publication. You will also need to give proper attribution to the copyright holders in your paper. Please ensure you upload any necessary grants of rights alongside the final version of your manuscript. More information is available on our Rights and permissions page. Failure to obtain the appropriate rights and to supply a completed third party rights table will delay the publication of your article. The editorial assistant (cc'd) can help with any questions.

COVER ARTWORK: We welcome submissions of artwork for consideration for our cover. More information can be found in our guide for cover artwork. The file name(s) should include the manuscript reference number and be labelled as a cover suggestion; a short description is also preferred. Illustrations should be selected more for their aesthetic appeal than for their scientific content. We cannot promise that your suggestions will be selected for the cover, as competition is intense.

IMAGE INTEGRITY: We strongly advise that you go carefully through all the data (including Extended Data and Supplementary Information) to ensure there are no accidental image/data duplications, other image manipulations or data errors. Such issues generally require correction after publication. Any image provided for publication, either in print or online (including Extended Data and Supplemental Information), may be subject to a quality control process to check for image integrity and manipulation. A discussion of our standards regarding how images should be prepared and presented can be found here.

EXTENDED DATA: Extended Data do not appear in print but are included online within the full-text HTML and integrated in the downloadable PDF. Extended Data are an integral part of the paper, and only data that directly contribute to the main message should be included. All Extended Data must be referred to in the main text, and their legends should be listed sequentially at the end of the main text file, not in the Extended Data files. Extended Data should be assembled into a maximum of 10 A4 size, multi-panelled display items. They must be supplied as individual files in .jpg, .tif or .eps format ***only****. They should be of the same quality as the main figures, but there are important differences in their formatting. More specific instructions are provided here. If you need to describe a complex process, we encourage you to add a schematic of the main finding as part of the Extended Data to aid readers unfamiliar with the immediate discipline.*

SUPPLEMENTARY INFORMATION: Supplementary Information (SI) is online-only, peer-reviewed material that is essential background to the study (e.g., large data sets, more complex methods, and calculations), but which is too large or impractical, or of interest only

to a few specialists, to justify inclusion in the print version of the paper (see here for further details). While SI should not typically contain data figures (any figures additional to those appearing in the main text should be formatted as Extended Data), we require that the raw, uncropped data for gels be presented as an SI figure (see below). Tables may be included in SI, but only if they are unsuitable for formatting as Extended Data (e.g., tables containing large data sets that cannot fit a single page or raw data tables that are best suited to Excel files). If a manuscript has SI, each discrete SI item (e.g., videos, tables) must be referred to at an appropriate point in the main text file. You must also provide a Word file entitled "SI Guide", containing a cover page with manuscript title and author information; a table of contents (preferably with page numbers); and then any SI text, notes, figures, and titles and legends for any separate SI files; for additional information see here.

We recommend that you pay careful attention to the formatting of the SI because it is not subedited. After the paper has been accepted, SI files can only be amended for critical changes to the scientific content, not for style.

SOURCE DATA (GRAPHS): To increase transparency, we strongly encourage you to provide, in spreadsheet form, the data underlying the graphical representations used in figures. For all experiments presenting data from animal models, this is a requirement and is not optional. This is in addition to our well-established data-deposition policy for specific types of experiments and large datasets. Online readers of the manuscript will be able to access the graphical source data directly from the figure legend. Spreadsheets must be submitted in .xls, .xlsx or .csv formats. One file per figure is permitted. If there is a multi-panelled figure, the source data for each panel should be clearly labeled in the file; alternatively the source data for a figure can be included in multiple, clearly labeled sheets within an Excel file. File sizes of up to 30 MB are permitted, but it is expected that the vast majority of graphical source data files will be considerably smaller than this. When submitting these files with your manuscript, you should select the "Source Data" file type and use the title field in the file description tab to indicate the figure(s) to which the source data pertain. Source data should not be provided as Extended Data.

We will not send your revised paper for further review. If the revised paper is in our format (as detailed above), in accessible style and of appropriate length, we shall begin the acceptance process.

In order to accept your paper, we require the following electronic files:

** A cover letter describing your response to any editorial comments and detailing any format changes during revision, particularly if the overall length is affected. Please include a final word count of the manuscript within your covering letter*

** A point-by-point response (preferably in Word) to any remaining issues raised by our referees.*

** The final version of your text as a Word document. Word Equation Editor/MathType should be used only for formulae that cannot be produced using normal text or symbol font. If this is not possible, the manuscript can be supplied as a single plain vanilla TeX or LaTeX file that includes all references and abbreviations, with no special formatting, as well as a PDF version that is uploaded as a 'related manuscript file'.*

** Production-quality versions of all figures (see above).*

** The final version of the Extended Data.*

** The final version of any Supplementary Information, presented as one file (ideally a PDF) if feasible, as well as a separate SI Guide.*

** Source Data, if appropriate.*

** For optimal quality videos we encourage H.264 encoding and a standard aspect ratio of 16:9 (4:3 is second best), without compression.*

** Completed and signed copies of the following **three (or four) forms**, uploaded as a "Related Manuscript File" file type:*

1) Biology editorial checklist;
2) Reporting summary;
3) Third party rights table;
4) Code and software submission checklist (if applicable).

Nature has now transitioned to a unified Rights Collection system which will allow our author services team to quickly and easily collect the rights and permissions required to publish your work. Once your paper is accepted, you will receive an email in approximately 10 business days providing you with a link to complete the grant of rights. If you choose to publish Open Access, our author services team will also be in touch at that time regarding any additional information that may be required to arrange payment for your article. If you have any questions please contact asjournals@springernature.com.

***You may need to take specific actions to achieve compliance with funder and institutional open access mandates.** If your research is supported by a funder that requires immediate open access (e.g. according to Plan S principles) then you should select the gold OA route, and we will direct you to the compliant route where possible. If you select the subscription publication route our standard licensing terms will need to be accepted, including our self-archiving policies. Those standard licensing terms will supersede any other terms that you or any third party may assert apply to any version of the manuscript.*

We hope to hear from you within two weeks; please let us know if the process may take longer.

Yours sincerely,

Sebastián Villamizar Santamaría
Senior Editor
Nature

Referees' comments:

Referee #2 (Remarks to the Author):

===
===========
FIELD 1 — "Remarks on Code Availability" (transmitted to authors)
===
===========

I reviewed the code and supporting materials deposited in the OSF repository associated with this submission, and I ran the analysis pipeline end-to-end on the simulated data. The data underlying the paper is confidential banking data and cannot be shared, which is appropriate; the authors instead provide a self-contained pipeline that generates simulated data preserving the relevant statistical properties (means and standard deviations) of the original, together with the parameter files needed to do so. The analysis is therefore fully demonstrable without access to the confidential source data.

The renv environment restored cleanly, the dependencies installed without incident, and all five steps executed in the documented order and ran to completion. They produce the full set of outputs mapped in the README: Figure 1 from the main analysis, the propensity-score balance tables and figures (Supplementary Tables 7-9, Figures 4-6), the temporal-clustering results, and Figures 2-3. The propensity-score matching achieves good covariate balance on the simulated sample, with all post-match standardized mean differences falling below roughly 0.06. I am satisfied that the analysis is reproducible from the materials provided. Because the demonstration runs on simulated data and at reduced permutation and subsample settings, the figures reproduce the structure and qualitative behavior of the published results rather than their exact magnitudes, which is the appropriate and expected standard for a study built on confidential data.

The code is clearly organized and well documented. The five-step structure is logical, the README maps each output to its corresponding manuscript figure or table, and pseudocode.md gives a readable account of the analytical logic. I note in particular that the two concerns I raised in the first round have been resolved: the absolute file paths have been removed in favor of paths relative to the execution directory, and the R package environment is now fully version-locked via renv.lock. Both changes materially improve reproducibility.

I have a small number of minor packaging issues that the authors should correct, none of which affects the validity of the analysis:

1. The README "Quick Start" instructs reviewers to run setup.sh and then run_analysis.sh, but neither script is present in the repository; only the individual step scripts are included. The authors should either add the two wrapper scripts or update the README to point solely to the step-by-step instructions.

We thank the reviewer for their thorough review of the code, for testing its reproducibility on macOS, and for providing template shell scripts. We adopted the reviewer's suggestion and have included setup.sh and run_analysis.sh in the repository. These scripts automate environment setup and execution of the complete analysis pipeline, and the README has been updated accordingly.

2. The README refers to the final visualization script as step_5_visualise_temporal_pattern.R, but the file in the repository is named

step_5_temporal_pattern_viz.R. The names should be reconciled so the documented sequence can be run as written.

Thank you for identifying this inconsistency. We have updated the README and pseudocode documentation so that all references use the correct filename (step_5_temporal_pattern_viz.R), ensuring that the documented execution sequence matches the files provided in the repository.

3. The simulated demonstration dataset distributed with the submission (sample_data_2_pct.csv.gz) does not appear to be read by any script in the pipeline, which generates its own simulated data internally from the parameter files in inputs/. The authors may wish to either wire this file into the demo or clarify its intended role to avoid confusion.

We thank the reviewer for highlighting this point. The file sample_data_2_pct.csv.gz was included at the request of the handling editor as a standalone demonstration dataset. It is not used by the reproducibility pipeline, which instead generates simulated data internally from the distributional parameters provided in the inputs/ directory. We have clarified the role of this file in the README to avoid confusion.

4. A compiled Python artifact (__pycache__/functions.cpython-313.pyc, built under Python 3.13) is included although the stated requirement is Python 3.10 or later. This is harmless but should be removed from the distribution.

We thank the reviewer for identifying this issue. The compiled Python cache file has been removed from the repository, and only the source code required to reproduce the analyses is now included in the distribution.

5. The R environment is pinned via renv.lock, but the Python dependencies in requirements.txt are specified only with lower bounds (>=). Because the clustering step imports a non-public function from MNE (mne.stats.cluster_level._find_clusters), which is more likely than the public API to change across releases, I would encourage pinning the Python dependencies to exact versions as well to protect long-term replicability.

We thank the reviewer for this helpful suggestion. We have updated the Python environment specification by pinning the versions of the core analysis packages (mne and glmtools), including mne, which provides the non-public clustering function used in the analysis. The remaining scientific Python packages retain minimum version requirements to preserve compatibility across supported Python releases while ensuring reproducibility of the analysis pipeline.

6. A global seed is set, but several stages draw fresh stochastic samples during execution, so the demonstration is not fully deterministic run-to-run (for example, the number of significant clusters varies slightly between executions). This does not affect the validity of the demonstration, but seeding each stochastic stage would make the example exactly reproducible and is a small, worthwhile addition.

We thank the reviewer for this suggestion. We have revised the analysis pipeline so that stochastic operations are controlled by a single fixed random seed in the R workflow (step_1_setup.R) and a single fixed random seed in the Python clustering analysis (step_4_clustering.ipynb). This ensures that repeated executions of the demonstration pipeline produce identical results while maintaining a simple and transparent seeding

strategy.

Overall, the data availability statement is appropriate and the code is in good order. Having run the pipeline to completion and confirmed it reproduces the documented outputs, I am satisfied that the reproducibility concerns from the previous round have been adequately addressed. The remaining items above are minor packaging fixes that can be handled at the copy-editing stage.

Referee #2 (Remarks on code availability):

Regarding the execution shell scripts setup.sh, and run_analysis.sh. I have written a working version of them in trying to assemble the code flow. This worked for me on MacOS GNU bash, version 3.2.57.

```
#!/usr/bin/env bash
#
# setup.sh
#
# Restores the locked R environment (renv) and builds a Python virtual
#
# Usage:
# bash setup.sh
#
# Notes:
# - The R restore can be slow the first time: renv builds pinned package
#
set -euo pipefail
cd "$(dirname "$0")"

# --- R environment ------------------------------------------------------
command -v Rscript >/dev/null 2>&1 || { echo "ERROR: Rscript not found on PATH." >&2;
exit 1; }
echo ">>> Restoring R environment via renv (this may take a while on first run)..."
Rscript -e 'if (!requireNamespace("renv", quietly = TRUE)) install.packages("renv", repos =
"https://cloud.r-project.org"); renv::restore(prompt = FALSE)'

# --- Python environment -------------------------------------------------
echo ">>> Building Python environment from requirements.txt..."
if command -v uv >/dev/null 2>&1; then
uv venv .venv
uv pip install --python .venv -r requirements.txt
else
command -v python3 >/dev/null 2>&1 || { echo "ERROR: python3 not found on PATH."
>&2; exit 1; }
python3 -m venv .venv
# shellcheck disable=SC1091
source .venv/bin/activate
python -m pip install --upgrade pip
```

```
python -m pip install -r requirements.txt
deactivate
fi

echo ">>> Setup complete. Python venv is at ./.venv"
echo ">>> Next: bash run_analysis.sh"
```

```
#!/usr/bin/env bash
#
# run_analysis.sh — execute the full analysis pipeline in order.
#
# Runs steps 1–5 against the simulated data and writes all figures and
# tables
#
# Usage:
# bash run_analysis.sh
#
set -euo pipefail

# Always run from the directory this script lives in, so the scripts'
# relative paths (inputs/, helpers/, outputs/) resolve correctly.
cd "$(dirname "$0")"

# Required tooling.
command -v Rscript >/dev/null 2>&1 || { echo "ERROR: Rscript not found on PATH." >&2; exit 1; }
command -v jupyter >/dev/null 2>&1 || { echo "ERROR: jupyter not found on PATH." >&2; exit 1; }

run_r () {
echo ">>> [$1/5] Rscript $2"
Rscript "$2"
}

echo "=== Pipeline start: $(date) ==="

run_r 1 step_1_setup.R
run_r 2 step_2_psm.R
run_r 3 step_3_main_analysis.R

echo ">>> [4/5] jupyter nbconvert --execute step_4_clustering.ipynb"
jupyter nbconvert --to notebook --execute --inplace --ExecutePreprocessor.timeout=-1 step_4_clustering.ipynb

run_r 5 step_5_temporal_pattern_viz.R

echo "=== Pipeline complete: $(date) ==="
echo "Outputs written to: $(pwd)/outputs/"
```

```

*Referee #3 (Remarks to the Author):*

*The authors have substantially satisfied my recommendations. Thank you for your careful work*

*Referee #3 (Remarks on code availability):*

*The code looks satisfactory to me*
```
